# Reagent-dictated site selectivity in intermolecular aliphatic C–H functionalizations using nitrogen-centered radicals†
†Electronic supplementary information (ESI) available: Experimental details and characterization data. CCDC 1553660. For ESI and crystallographic data in CIF or other electronic format see DOI: 10.1039/c8sc01756e


**DOI:** 10.1039/c8sc01756e

**Published:** 2018-05-14

**Authors:** Anthony M. Carestia, Davide Ravelli, Erik J. Alexanian

**Affiliations:** a Department of Chemistry , The University of North Carolina at Chapel Hill , Chapel Hill , North Carolina 27599 , USA . Email: eja@email.unc.edu; b PhotoGreen Lab , Department of Chemistry , University of Pavia , Viale Taramelli 12 , 27100 Pavia , Italy . Email: davide.ravelli@unipv.it

## Abstract

The site selectivities of intermolecular, aliphatic C–H bond functionalizations are central to the value of these transformations. While the scope of these reactions continues to expand, the site selectivities remain largely dictated by the inherent reactivity of the substrate C–H bonds. Herein, we introduce reagent-dictated site selectivity to intermolecular aliphatic C–H functionalizations using nitrogen-centered amidyl radicals. Simple modifications of the amide lead to high levels of site selectivity in intermolecular C–H functionalizations across a range of simple and complex substrates. DFT calculations demonstrate that the steric demand of the reacting nitrogen-centered radical is heavily affected by the substitution pattern of the starting amide. Optimization of transition state structures consistently indicated higher reagent-dictated steric selectivities using more hindered amides, consistent with experimental results.

## Abstract 2

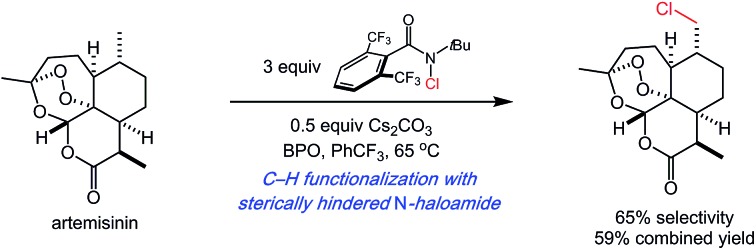
Intermolecular aliphatic C–H functionalizations using nitrogen-centered radicals with reagent-dictated site selectivities are described, including reactions targeting primary C–H bonds across a range of simple and complex substrates.

## Introduction

Recent advances in intermolecular aliphatic C–H functionalization have provided unique tools for the synthesis and late-stage derivatization of complex molecules.^1^ The site selectivity of these reactions is critical to their application owing to the number of different C–H bonds present in many substrates. The intrinsic reactivity of the substrate C–H bonds often dictates the site selectivity of a given functionalization.^2^ Among the factors influencing the relative reactivity of C–H bonds are steric accessibility, electron richness, and participation in hyperconjugation. Sole reliance on substrate-dictated selectivity limits the scope of intermolecular C–H functionalization, as many substrates contain C–H bonds with similar reactivity, thus affording poor discrimination between sites. Moreover, functionalizations at less inherently reactive C–H bonds are impossible without additional elements of control.

Reagents or catalysts that override inherent substrate-dictated site selectivity hold significant promise in expanding the capabilities of intermolecular C–H functionalization. Noteworthy advances in this area have been achieved *via* the use of modified ligand architectures in catalytic C–H functionalizations proceeding *via* high-valent metal oxo or metal carbenoid intermediates.^3^ Protein engineering has also led to enzymatic aliphatic C–H functionalizations with altered regioselectivities.^4^

**Fig. 1:**
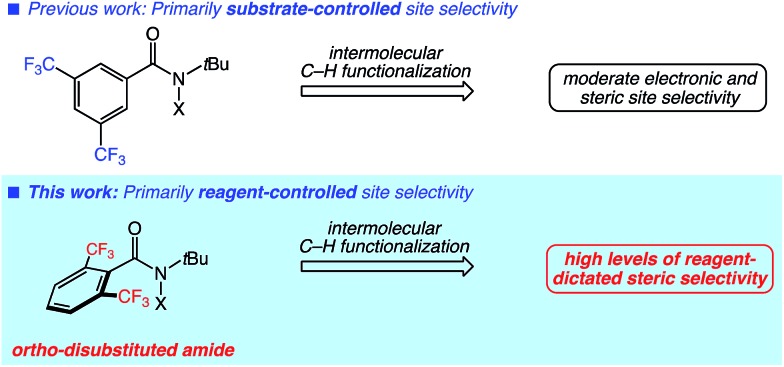
Substrate *versus* reagent control in site-selective, intermolecular aliphatic C–H functionalizations involving nitrogen-centered radicals.

We have previously developed practical systems for aliphatic C–H functionalization using readily accessed *N*-halo- and *N*-xanthylamides.^5^ In those studies, moderate site selectivities were achieved primarily through substrate control. We anticipated that the highly modular nature of our reagents would offer unique opportunities in introducing reagent-dictated site selectivity (Fig. 1). Herein, we report our studies towards this goal using an easily prepared, modified amide to provide high levels of site selectivity in C–H functionalizations of diverse substrates. Computational studies of both the amidyl radical structures and representative C–H abstraction transition states were performed to shed light on the unique, reagent-dictated site selectivities observed.

## Results and discussion

We commenced our studies with the functionalization of *trans* decalin, a benchmark substrate used in studies of intermolecular, aliphatic C–H functionalizations.^6^ The difficulty of achieving steric discrimination between the methylene sites is evident upon surveying the site selectivities of several previously reported functionalizations (Table 1, entries 1–4). For example, iron-catalyzed oxidations of *trans* decalin with bulky tetradentate ligands poorly discriminated between these sites (entries 1 and 2).6*b* Manganese-catalyzed C–H chlorination using a hindered tetramesitylporphyrin (TMP) system achieved good selectivity (4.0 : 1), although tertiary functionalization was also observed (entry 3).6*c* The C–H chlorination using our reported *N*-chloroamide **3** proceeded with no selectivity between the methylene sites (entry 4).5*b*

**Table 1:** Site selectivity of the functionalization of *trans* decalin comparing catalytic systems and *N*-chloroamides^*a*^ ^,^^*b*^

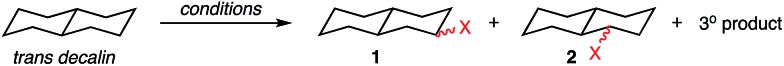					
Entry	Functionalization system	Isomer distribution (%)			**1**/**2**
		**1**	**2**	3° pdt	
1	(*R*,*R*′)-[Fe(OTf)_2_(pdp)], H_2_O_2_ (X = ketone)	46	40	13	1.2
2	(*S*,*S*′)-[Fe(OTf)_2_(^tips^mcp)], H_2_O_2_ (X = ketone)	65	34	1	1.9
3	Mn(TMP)Cl, NaOCl	76	19	5	4.0
4	*N*-chloroamide **3**	50	50	<1	1.0
5	*N*-chloroamide **4**	66	34	<1	2.0
6	*N*-chloroamide **5**	65	35	<1	1.9
7	*N*-chloroamide **6**	87	13	<1	6.5
8^*c*^	*N*-chloroamide **6** (76% combined yield)	88 (2.5 : 1 dr)	12 (1 : 1 dr)	<1	7.2
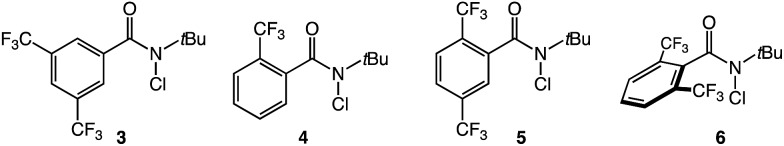					

^*a*^Reactions in entries 1–3 as reported in ref. 6b and c. Reactions of the *N*-chloroamides were performed in PhCF_3_ at 65 °C using 10 mol% benzoyl peroxide as initiator with 5 equiv. of substrate, 0.5 equiv. of Cs_2_CO_3_, and 1 equiv. *N*-chloroamide. Yields and selectivities of the methylene functionalizations were determined by ^1^H NMR analysis, and those of the tertiary products were determined by GC with dodecane as the internal standard.

^*b*^X = Cl unless otherwise noted.

^*c*^Reaction performed using 1 equiv. of substrate.

We hypothesized that increasing the steric demand of the *N*-chloroamide would offer improved site selectivities. We thus evaluated a set of *N*-chloroamides containing *ortho* trifluoromethyl substitution of the benzamide, which are readily available on scale (>10 grams). Reagents **4** and **5** containing a single *ortho* substituent increased the selectivity for the less hindered, distal methylene site, although the ratio of **1** : **2** remained close to 2.0 : 1 (entries 5 and 6). We next sought to prepare the highly hindered 2,6-bistrifluoromethyl reagent **6**, which was obtained *via* addition of the aryl Grignard to *t*Bu isocyanate.^7^ The C–H chlorination of *trans* decalin with **6** proceeded with higher site selectivity than the other *N*-chloroamides or metal-catalyzed systems, with a 6.5 : 1 ratio of products (entry 7). Performing the reaction with 1 equiv. of substrate provided the products in a 7.2 : 1 ratio in 76% combined yield (entry 8). To the best of our knowledge, this is the most site-selective secondary C–H functionalization of *trans* decalin known using substrate as limiting reagent.^8^–^10^

**Fig. 2:**
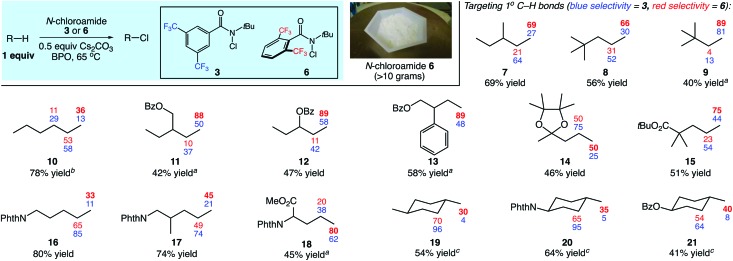
Reagent-dictated site selectivities (%) in C–H chlorinations of diverse substrates. Yields provided are for reactions using reagent **6**. Reactions were performed in PhCF_3_ at 65 °C using (2 × 10 mol%) benzoyl peroxide as initiator with 1 equiv. of substrate, 0.5 equiv. of Cs_2_CO_3_, and 1–1.5 equiv. *N*-chloroamide. Yields and selectivities were determined by GC with dodecane as an internal standard or ^1^H NMR analysis. See ESI† for further details regarding the distribution of minor products. ^*a*^3 equiv. of substrate used. ^*b*^5 equiv. of substrate used. ^*c*^Secondary site selectivity includes all secondary chlorination products.

We next evaluated the site selectivities of the C–H chlorination using reagent **6** across a range of substrates. As shown in Fig. 2, reagent **6** is a free-flowing white powder that is readily available on scale. The C–H chlorination of these substrates with *N*-chloroamide **6** also proceeded with unique site selectivities, and notably includes radical functionalizations selective for primary sites. Site selectivities with our first-generation reagent **3** are shown for direct comparison. For example, the chlorination of 3-methylpentane (**7**) with reagent **3** showed a preference for the 2-position (64%) over the 1-position (27%). However, reagent **6** provided a remarkable reagent-dictated switch in site selectivity, favoring the 1-chloro isomer (69%, 69% combined yield). Free radical species demonstrating such selectivity for the strongest, primary C–H bond over secondary or tertiary C–H bonds in synthetic alkane functionalizations are unknown to the best of our knowledge.^11^

Our efforts continued with the C–H chlorination of diverse compounds (Fig. 2). The chlorination of 2,2-dimethylpentane (**8**) and 2,2-dimethylbutane (**9**) demonstrate that fully substituted carbon centers enable steric discrimination between C–H sites, with 89% primary C–H selectivity in the reaction of substrate **9** with *N*-chloroamide **6**. There was an increase in the level of primary functionalization of *n*-hexane (**10**) with reagent **6**, however the 2-position remained the preferred site of functionalization.

The presence of electron-withdrawing functionality disfavors reaction at proximal methylene sites and increased the site selectivity for the primary positions to 88% and 89% for substrates **11** and **12**, respectively. The functionalization of 2-phenylbutyl benzoate (**13**) remarkably demonstrated a high selectivity for the primary position (89%) even in the presence of a benzylic C–H site. The fully substituted carbon centers in acetal **14** and ester **15** lead to a clear reagent-dictated change in selectivity between reagents **3** and **6**; the primary C–H chlorination product is favored with up to 75% selectivity with ester substrate **15**.

Phthalimide-protected amines undergo intermolecular C–H functionalizations using amidyl radicals with high levels of electronically-dictated site selectivities.^5^ We surveyed several substrates containing this electron-withdrawing group. With the lack of major steric discrimination between the C–H sites, the functionalization of substrate **16** with reagent **6** only increased the level of primary functionalization to a modest 33%. In the case of substrate **17** containing chain branching, a higher level of primary functionalization was observed (45%). The shorter aliphatic chain in the norleucine derivative **18** electronically disfavored methylene functionalization, and the use of hindered *N*-chloroamide **6** resulted in good primary selectivity (80%).

The reagent-dictated site selectivity provided by *N*-chloroamide **6** is also evident in reactions with cyclic substrates **19–21**. Each of these structures contain eight methylene and either three or six methyl C–H bonds for chlorination. In all cases C–H chlorination with *N*-chloroamide **3** was highly methylene selective. Switching to reagent **6** for the chlorination of these substrates significantly increased the levels of methyl group functionalization. This unique selectivity profile enabled the 1° C–H functionalizations of methyl groups in complex substrates (*vide infra*).

**Fig. 3:**
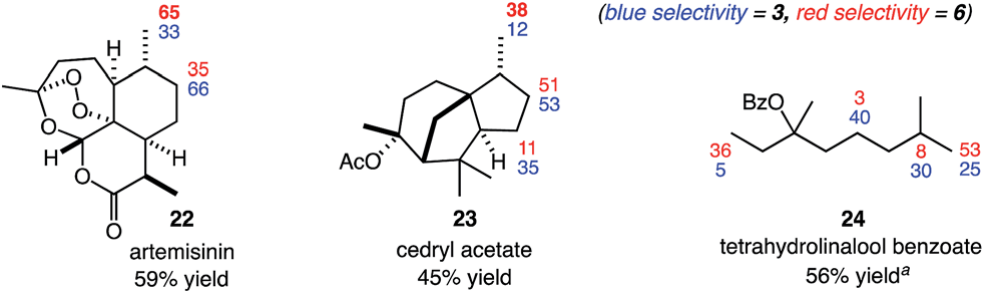
C–H chlorinations of complex natural products. See the ESI† for reaction details. Yields and selectivities were determined by GC with dodecane as an internal standard or ^1^H NMR analysis. ^*a*^3 equiv. of substrate used.

We next sought to capitalize on the unique selectivity profile of reagent **6** in the aliphatic C–H functionalization of complex natural products (Fig. 3). The late-stage functionalization of the antimalarial artemisinin has been targeted in several studies.3b,6e,^12^,^13^ Most commonly, the reactive site is the methine of the methyl-bearing carbon in the cyclohexyl ring. Recently, groundbreaking studies from the White group (catalyst-dictated selectivity)3*b* and the Fasan group (enzyme-dictated selectivity)^13^ have achieved transformations capable of overcoming this reactivity profile.

We questioned whether the use of reagent **6** would enable C–H functionalization of the methyl group of the cyclohexyl ring. To date, only enzymatic catalysts have successfully hydroxylated this methyl site.^13^ In the event, chlorination with *N*-chloroamide **6** provided the desired product with moderate site selectivity (1.9 : 1) in 59% combined yield (Fig. 3). The C–H chlorinations of cedryl acetate and tetrahydrolinalool benzoate also demonstrated the ability of reagent **6** to target primary C–H sites. Switching from reagent **3** to reagent **6** brings about a significant increase in methyl functionalization, most notably with 89% primary selectivity in the functionalization of **24**.

**Fig. 4:**
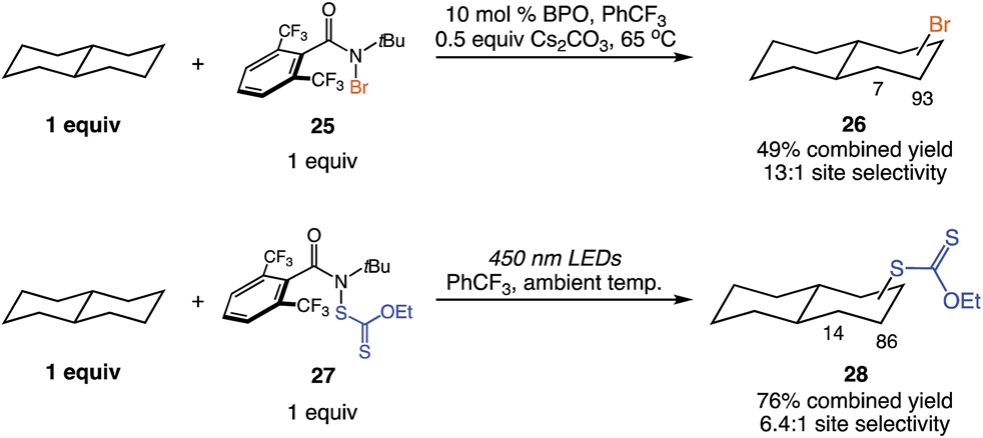
Similar reagent-dictated site selectivities are observed in C–H brominations and xanthylations. Combined yields and site selectivities determined by ^1^H NMR analysis.

In prior work, we have developed protocols for C–H bromination and xanthylation using functionalized amides.5a,c As these transformations are likewise synthetically valuable,^14^ we sought to demonstrate that the reagent-dictated selectivity herein was transferrable to those reactions. We prepared the *N*-bromo and *N*-xanthylamides **25** and **27** to address this question. The C–H functionalizations of *trans* decalin with these reagents provided similar methylene site selectivities and reaction efficiencies as compared to the chlorination (Fig. 4).^15^ Thus, tuning the amidyl radical responsible for dictating the site selectivity of the C–H abstraction is a general approach to introducing reagent control to this class of C–H functionalizations.

**Fig. 5:**
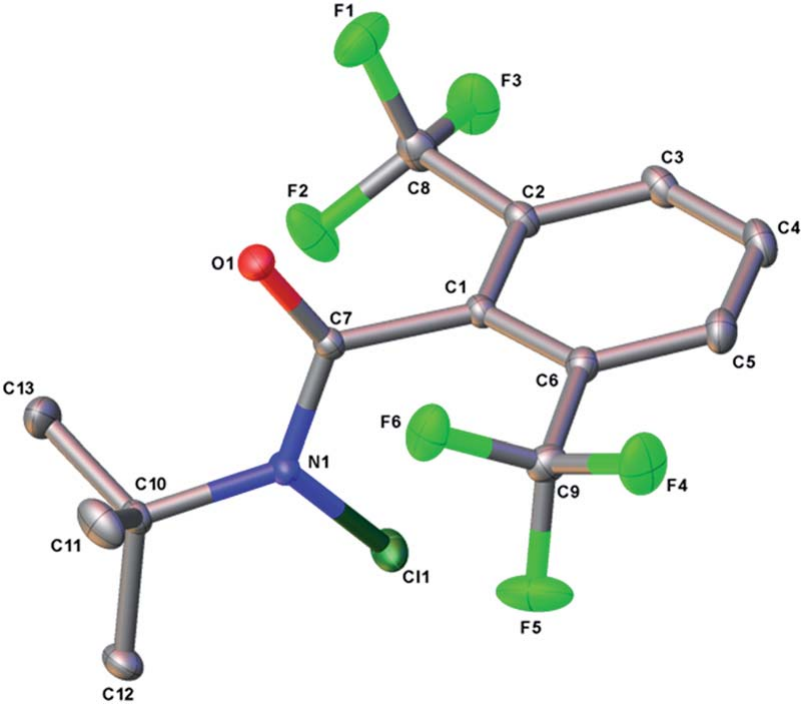
OLEX2 structure of *N*-chloroamide **6**.

The structure of the *N*-chloroamide **6** provides some insight into the basis for increased site selectivity (Fig. 5).^16^ Non-bonded interactions between the *ortho* trifluoromethyl groups and the amide bond dictate a conformation where the aromatic plane and the amide are nearly orthogonal. This is clear from the C_6_C_1_C_7_O_1_ dihedral angle, which is 96.6°. The *ortho* substituents positioned above and below the plane of the amide are expected to shield the nitrogen atom and the resulting amidyl radical, leading to an increased selectivity for sterically less hindered C–H sites. This unique feature of reagent **6** is not present in *N*-chloroamides **3**–**5**, which have more planar structures.

In order to gain further insight regarding the observed site selectivities, we performed computational studies of the structures of the amidyl radicals involved, as well as the hydrogen atom abstractions of select substrates. The relevant transition states of the H-atom abstractions were optimized, and the associated energy changes were evaluated. A DFT approach was used, with the ωB97XD functional, standard 6-31G(d,p) basis set, and Gaussian16 software (see ESI† for details). Optimizations were initially carried out *in vacuo*, then solvent effects were included through single-point calculations at the same level of theory with the SMD model and adopting the standard parameters for CH_2_Cl_2_ (a computationally convenient alternative to PhCF_3_,^17^ the solvent used in this work).

**Fig. 6:**
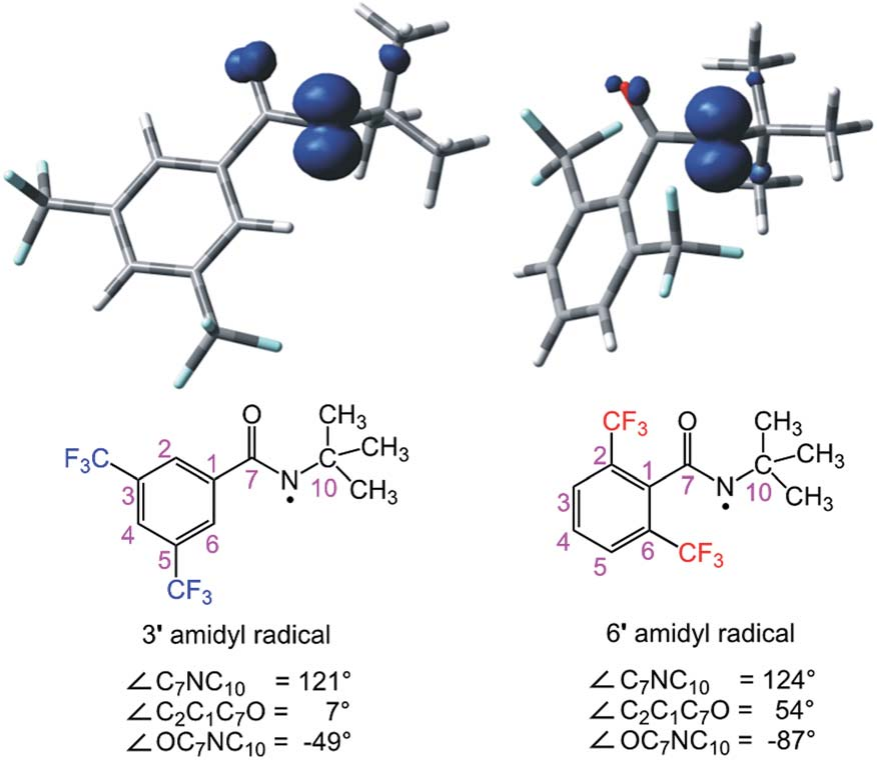
Optimized structures of amidyl radicals **3′** and **6′**, including their spin density plots and some relevant parameters.

The calculated structures of amidyl radicals **3′** and **6′** are shown in Fig. 6, along with their spin density plots and some relevant parameters. Both species are essentially *N*-centered radicals; the nitrogen atoms have sp^2^-like hybridization, and the unpaired electron lies in a pure p orbital perpendicular to the plane consisting of the substituents attached to the nitrogen atom. The presence of the *tert*-butyl group on the nitrogen atom is known to impart a twisted structure to amidyl radicals, decreasing the conjugative delocalization of the unpaired electron from N to O.^18^ This is also apparent in the case of amidyl radicals **3′** and **6′**, where the OC_7_NC_10_ dihedral angles markedly deviate from zero. The environments around the radical centers are also significantly affected by the substitution patterns of the phenyl ring, and are markedly different for the two amidyl radicals, however. For example, the aromatic ring of **3′** is fully conjugated with the amide carbonyl, and they lie in nearly the same plane (only a 7° deviation is observed). On the other hand, the aromatic ring of **6′** is out of the carbonyl plane (54° dihedral angle) owing to the steric hindrance of the two *ortho* CF_3_ substituents. As a result, several hydrogen atoms of the *tert*-butyl group are within a <3 Å distance of the fluorine atoms in **6′**, consistent with a highly congested chemical environment around the radical center.

**Fig. 7:**
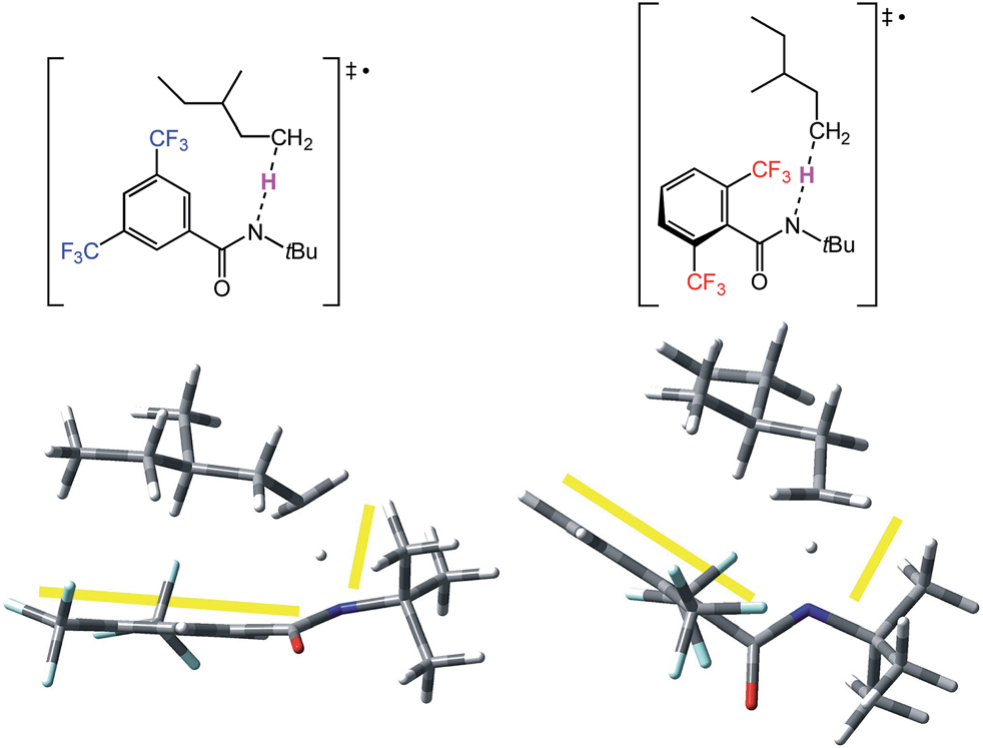
Optimized structures of the TS for the reactive primary C–H abstraction of 3-methylpentane by **3′** (left) and **6′** (right).

We next modeled the transition states (TS) of the H-atom abstractions by **3′** and **6′** involving select substrates, with the goal of identifying important factors that account for the observed site selectivities. We studied a diverse set of cyclic and acyclic substrates, including *trans* decalin, 3-methylpentane (**7**) and 3-pentyl acetate–a model for the benzoate substrate **12**. Representative TS for the abstractions are shown in Fig. 7 for the reactive primary C–H bonds of 3-methylpentane (**7**) involving amidyl radicals **3′** (left) and **6′** (right). The reactive site is much more crowded in the case of **6′** (Fig. 7, right), as highlighted by the yellow lines in the TS forming a smaller angle than in the case for **3′** (Fig. 7, left). The primary C–H bond of 3-methylpentane approaches amidyl radical **6′** through a relatively tight pocket. On the other hand, the substrate engages with the nearly flat conformation of **3′** from the top. It is worth noting that the two amidyl radicals retain the geometric parameters from Fig. 6 in the TS.

The Gibbs free energy barriers (Δ*G*^‡^) and changes (Δ*G*) for the C–H abstractions evaluated are listed in Table 2. As expected, all C–H abstractions occur with an overall energy gain (negative Δ*G* values), regardless of the substrate, position, or amidyl radical considered. Comparisons of the energy barriers involved indicate different trends for the two reagents, however. In particular, the last column of Table 2 reports ΔΔ*G*^‡^ values quantifying the energetic difference between the two amidyl radicals for reaction at a given C–H site. For example, amidyl radical **6′** is more reactive than **3′** in the abstraction at the methylene C–H site distal from the ring fusion of *trans* decalin, as indicated by the negative ΔΔ*G*^‡^ value (for the observed experimental results see Table 1). On the other hand, virtually no difference is observed at the proximal methylene site, with a small ΔΔ*G*^‡^ of +0.35 kcal mol^–1^.

**Table 2:** Calculated parameters for hydrogen atom abstractions by amidyl radicals **3′** and **6′** from selected substrates (Sub–H)^*a*^

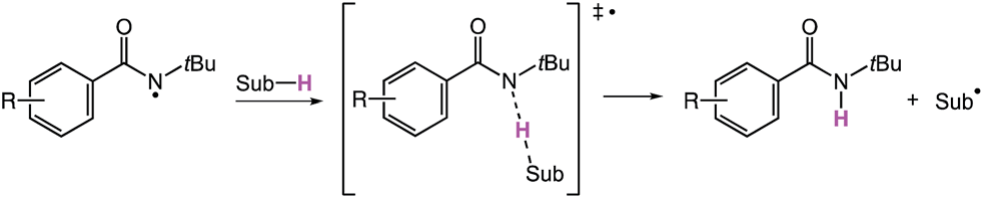					
Functionalization site	**3′** (*meta* substituted)		**6′** (*ortho* substituted)		ΔΔ*G*^‡^^*b*^
	Δ*G*^‡^	Δ*G*	Δ*G*^‡^	Δ*G*	
***trans* decalin** ^*c*^					
Distal	11.81	–8.46	9.56	–13.50	–2.25
Proximal	9.07	–9.21	9.42	–11.96	+0.35

**3-Methylpentane (**7**)**					
Primary	13.52	–5.84	8.72	–10.04	–4.80
Secondary	10.04	–10.48	10.15	–13.89	+0.11

**3-Pentyl acetate**					
Primary	12.46	–6.28	10.38	–9.57	–2.08
Secondary	11.54	–8.22	11.24	–12.29	–0.30

^*a*^Energy values expressed in kcal mol^–1^ at the SMD(CH_2_Cl_2_)-ωB97XD/6-31G(d,p) level of theory have been reported (see Table S41 in the ESI for details).

^*b*^Value obtained by taking the difference of Δ*G*^‡^(**6′**) – Δ*G*^‡^(**3′**). A negative value indicates a lower energy barrier for **6′** than for **3′**.

^*c*^The functionalizations of the axial and equatorial C–H bonds have been considered, but only the parameters of the most stable TS have been reported (see Table S41).

In the case of acyclic substrates, amidyl radical **6′** is consistently more reactive than **3′** at primary C–H sites, while little difference is observed at secondary positions. Notably, C–H abstraction at the reactive primary position of 3-methylpentane (**7**) shows the largest (in absolute terms) calculated ΔΔ*G*^‡^ value (–4.80 kcal mol^–1^), which is consistent with experimental results; functionalization at this position involves the largest selectivity change upon switching from **3′** to **6′** (27% to 69%, see Fig. 2). Overall, our computational findings are in good agreement with the experimental results, as reagent **6** consistently provided higher selectivities for the least hindered positions of substrates–either primary or distal—throughout our studies.

## Conclusions

In conclusion, we have developed an approach to introduce reagent-dictated site selectivity to aliphatic C–H functionalizations using tuned nitrogen-centered radicals. The use of hindered *N*-functionalized amides has led to C–H functionalization of diverse substrates that are among the most site-selective intermolecular C–H transformations known. This strategy has also enabled the first radical-mediated aliphatic C–H functionalizations targeting primary C–H bonds across a wide range of simple and complex substrates. Computational studies revealed the different chemical environments of the reacting amidyl radicals, and established a direct correlation between the steric hindrance at the reacting site and the preference for functionalization of the least hindered substrate C–H bonds. Future studies will continue to develop unique reagents that offer increased levels of site selectivity in intermolecular C–H functionalization for applications in chemical synthesis.

## Conflicts of interest

There are no conflicts to declare.

## Supplementary Material

Supplementary information

Crystal structure data
