# Supplementary material for: Reagent-dictated site selectivity in intermolecular aliphatic C–H functionalizations using nitrogen-centered radicals
Source: Chem Sci. 2018 May 14;9(24):5360–5. doi: 10.1039/c8sc01756e (PMC6009468; doi:10.1039/c8sc01756e)
Supplement: Supplementary file 1 [file SC-009-C8SC01756E-s001.pdf]

Electronic Supplementary Material (ESI) for Chemical Science.
This journal is © The Royal Society of Chemistry 2018

Supporting Information

Reagent-Dictated Site Selectivity in Intermolecular Aliphatic C–H Functionalizations Using Nitrogen-Centered Radicals

Anthony M. Carestia, Davide Ravelli,*‡ and Erik J. Alexanian*†

† Department of Chemistry, University of North Carolina at Chapel Hill, Chapel Hill, NC 27599, United States

‡ PhotoGreen Lab, Department of Chemistry, University of Pavia, Viale Taramelli, 12, 27100, Pavia, Italy

†eja@email.unc.edu

‡davide.ravelli@unipv.it

Table of Contents

General Methods **S1-S2**
Reagent Synthesis **S2-S6**
Substrate Synthesis **S7-S11**
Standard Synthesis **S11-S19**
C-H Functionalizations **S19-S70**
X-Ray Crystallography Data **S71-S79**
Computational Data **S80-S122**
References **S123**
NMR Spectra **S124-S151**

General Methods

Proton and carbon magnetic resonance spectra (^{1}H NMR and ^{13}C NMR) were obtained using a Bruker model AVANCE III 400, 500, or 600 (^{1}H NMR at 400 MHz, 500 MHz 600 MHz and ^{13}C NMR at 126 MHz or 151 MHz) spectrometer with solvent resonance as internal reference (^{1}H NMR: $CDCl_3$ at 7.27 ppm, ^{13}C NMR: $CDCl_3$ at 77.00 ppm). Recycle delay (d1) is consistent for each type of experiment run and is set to 2.00 seconds for all experiments with the exceptions of ^{1}H NMR and ^{19}F NMR which are set to 1.00 seconds and HMBC and HMQC which are set to 1.50 seconds. ^{1}H NMR data is reported as follows: chemical shift, multiplicity (s = singlet, d = doublet, t = triplet, q = quartet, dd = doublet of doublets, ddd = doublet of doublet of doublets, td = triplet of doublets, tdd = triplet of doublet of doublets, qd = quartet of doublets, m = multiplet, br. s. = broad singlet), coupling constants (Hz), and integration. Mass spectra were obtained using either a Thermo LTqFT mass spectrometer with electrospray introduction and external calibration or an Agilent Gas Chromatograph-Mass Spectrometer with a 6850 series GC system and a 5973 Network Mass Selective Detector. GC Spectra were obtained using a Shimadzu GC-2010 gas chromatograph with a Shimadzu AOC-20s Autosampler, and Shimadzu SHRXI-5MS GC column. Flash Chromatography was performed using SiliaFlash P60 silica gel (40-63µm) purchased from Silicycle. Four different GC methods are used. Method 1: Initial temperature of 30.0 °C, ramping at 2.0 °C/min until 75.0 °C, holding for 1.0

min, then ramping at 30.0 °C/min until 250.0 °C, and holding for 2.0 min. Method 2: Initial temperature of 55.0 °C, ramping at 2.0 °C/min until 95.0 °C, then ramping at 30.0 °C/min until 250.0 °C, and holding for 2.0 min. Method 3: Initial temperature of 55.0 °C, ramping at 3.0 °C/min until 120.0 °C, then ramping at 30.0 °C/min until 250.0 °C, and holding for 2.0 min. Method 4: Initial temperature of 55.0 °C, ramping at 15.0 °C/min until 200.0 °C, then ramping at 3.0 °C/min until 250.0 °C, and holding for 2.0 min. Prep HPLC chromatography was done using a Perkin Elmer Flexar HPLC system equipped with Daicel IA and IC columns. Visualization was accomplished with short wave UV light (254 nm), aqueous basic potassium permanganate solution, or ethanolic acidic *p*-anisaldehyde solution followed by heating. Tetrahydrofuran (THF), diethyl ether (Et_2O), dichloromethane (DCM), toluene, acetonitrile (MeCN), and dimethylformamide (DMF) were dried by passage through a column of neutral alumina under nitrogen prior to use. Trifluorotoluene and benzene were dried over 3Å molecular sieves and degassed with argon prior to use. Carbon tetrachloride was distilled prior to use. All other reagents were obtained from commercial sources and used without further purification unless otherwise noted.

Reagent Synthesis

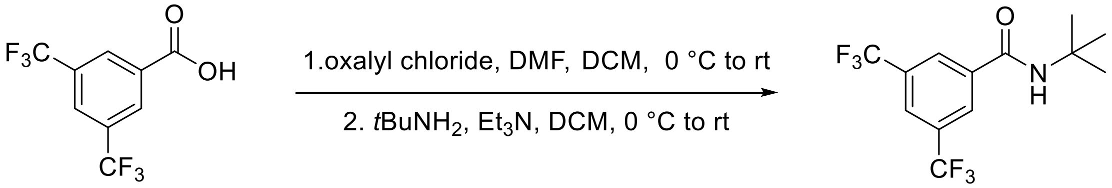

S1

***N*-(*tert*-butyl)-3,5-bis(trifluoromethyl)benzamide (S1)** was prepared according to a literature procedure, and spectral data were in accordance with the literature values.[1]

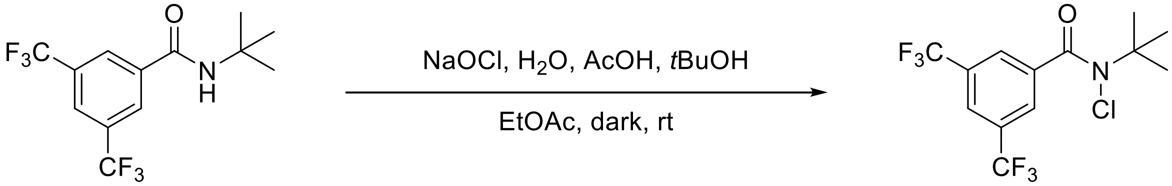

S1 **3**

***N*-(*tert*-butyl)-*N*-chloro-3,5-bis(trifluoromethyl)benzamide (3)** was prepared according to a literature procedure, and spectral data were in accordance with the literature values.[1]

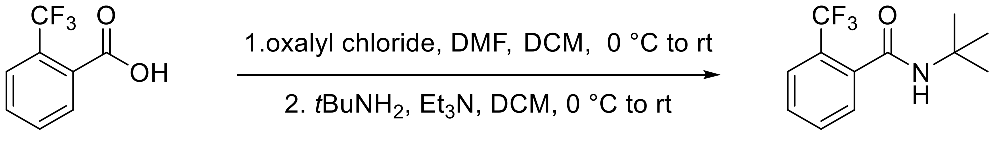

S2 **S3**

***N*-(*tert*-butyl)-2(trifluoromethyl)benzamide (S3):** To a flame dried 250 mL round bottom flask was charged with 2-(trifluoromethyl)benzoic acid (3.00 g, 15.8 mmol) and dissolved in 80.0 mL of DCM and few drops of DMF. The reaction cooled to 0 °C. Then oxalyl chloride (2.20 mL, 12.8 mmol) was added

slowly dropwise. The reaction was allowed to warm to room temperature and stir overnight. The reaction was conventrated *in vacuo* to afford crude acid chloride product. The crude acid chloride was dissolved in 80.0 mL of DCM and cooled to 0 °C. Then *tert*-butylamine (3.30 mL, 31.6 mmol) was added. The reaction was allowed to warm to room temperature and stir overnight. The reaction was washed with 10% sodium hydroxide solution, 1 M hydrochloric acid solution, and brine, dried with $MgSO_4$, and concentrated *in vacuo*. The crude product was purified via column chromatography (10% ethyl acetate in hexanes) to give the product (4.10 g, 99% yield) as a white solid.

^{1}H NMR: (600MHz ,$CDCl_3$) 7.68 - 7.65 (m, 1 H), 7.59 - 7.54 (m, 1 H), 7.54 - 7.47 (m, 2 H), 5.56 (br. s., 1 H), 1.45 (d, *J* = 0.7 Hz, 9 H)
^{13}C NMR: (151MHz ,$CDCl_3$) 167.0, 137.0 (q, *J* = 0.01 Hz), 132.0, 129.4, 128.6, 126.9, 126.1 (q, *J* = 0.03 Hz), 122.8, 52.2, 28.5
^{19}F NMR: (376MHz ,$CDCl_3$) -58.622
HRMS: calculated for $[C_{12}H_{14}F_3NO+H]^+$ = 246.1100, found = 26.1099

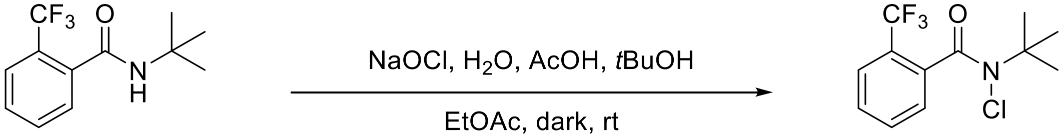

S3 **4**

***N*-(*tert*-butyl)-*N*-chloro-2-(trifluoromethyl)benzamide (4):** To a foil wrapped 1 L round bottom flask was charged with *N*-(*tert*-butyl)-2(trifluoromethyl)benzamide (2.00 g, 8.20 mmol) and *tert*-butanol (5.20 mL, 54.4 mmol) and dissolved in 161 mL ethyl acetate. To a foil wrapped addition funnel was added sodium hypochlorite (94.0 mL, 141 mmol), water (56.0 mL, 3.10 mmol), and glacial acetic acid (37.0 mL, 648 mmol). The contents of the addition funnel were added slowly to the reaction over 1 hour. The reaction was allowed to stir for 4 hours. The reaction was diluted with DCM and quenched with bicarbonate solution. The organic layer was separated and washed with bicarbonate, dried with $MgSO_4$, and concentrated *in vacuo*. The crude product was purified via column chromatography (5% ethyl acetate in hexanes) to give the product (1.28 g, 57% yield) as a white solid.

^{1}H NMR: (600MHz ,$CDCl_3$) 7.67 (d, *J* = 7.7 Hz, 1 H), 7.61 - 7.54 (m, 1 H), 7.52 - 7.46 (m, 1 H), 7.38 (d, *J* = 7.7 Hz, 1 H), 1.60 (s, 9 H)
^{13}C NMR: (151MHz ,$CDCl_3$) 171.6, 136.3 (q, *J* = 0.02 Hz), 131.7, 129.1, 127.0, 126.4 (q, *J* = 0.21 Hz), 126.4 (t, *J* = 0.03 Hz), 123.6 (d, *J* = 1.82 Hz), 64.9, 28.2
^{19}F NMR: (376MHz ,$CDCl_3$) -59.860
HRMS: calculated for $[C_{12}H_{13}ClF_3NO+H]^+$ = 280.0711, found = 280.0709

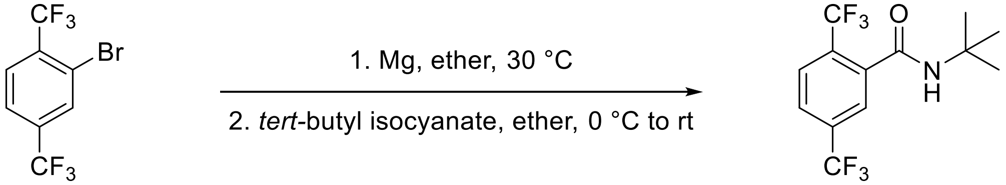

S4

***N*-(*tert*-butyl)-2,5-bis(trifluoromethyl)benzamide (S4):** To a flame dried 100 mL round bottom flask was charged with magnesium turnings (0.310 g, 12.8 mmol) in the glovebox. To the reaction 2.00 mL ether and a few drops of iodine in 1,2-dibromoethane was added to activate the magnesium. Then 2,5-bis(trifluoromethyl)bromobenzene (2.2 mL, 12.8 mmol) was dissolved in 11.0 mL ether and added to the reaction. The reaction was heated to 30 °C. The reaction was allowed to stir until all the magnesium was consumed. The reaction was cooled to 0 °C and 204 mL of ether was added to the reaction. Then *tert*-butyl isocyanate (1.32 mL, 11.6 mmol) was added. The reaction was allowed to warm to room temperature and stir overnight. The reaction was quenched with ammonium chloride solution and extracted with ethyl acetate, dried with $MgSO_4$, and concentrated *in vacuo*. The crude product was purified via column chromatography (10% ethyl acetate in hexanes) to give the product (0.730 g, 20% yield) as a white solid.

^{1}H NMR: (600MHz ,$CDCl_3$) 7.83 - 7.80 (m, 1 H), 7.79 - 7.76 (m, 2 H), 5.60 (br. s., 1 H), 1.47 (s, 9 H)
^{13}C NMR: (151MHz ,$CDCl_3$) 167.0, 137.0 (q, *J* = 0.01 Hz), 132.0, 129.4, 128.6, 126.9, 126.1 (q, *J* = 0.03 Hz), 122.8, 52.2, 28.5
^{19}F NMR: (376MHz ,$CDCl_3$) -59.154, -63.288
HRMS: calculated for $[C_{13}H_{13}F_6NO+H]^+$ = 314.0974, found = 314.0973

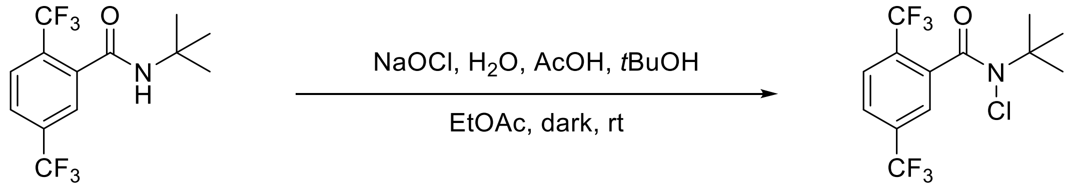

S4 **5**

***N*-(*tert*-butyl)-*N*-chloro-2,5-bis(trifluoromethyl)benzamide (5):** To a foil wrapped 500 mL round bottom flask was charged with *N*-(*tert*-butyl)-2,5-bis(trifluoromethyl)benzamide (600 mg, 1.90 mmol) and *tert*-butanol (1.20 mL, 12.5 mmol) and dissolved in 38.0 mL ethyl acetate. To a foil wrapped addition funnel was added sodium hypochlorite (21.7 mL, 32.6 mmol), water (13.0 mL, 0.720 mmol), and glacial acetic acid (8.60 mL, 151 mmol). The contents of the addition funnel were added slowly to the reaction over 1 hour. The reaction was allowed to stir for 5 hours. The reaction was diluted with DCM and quenched with bicarbonate solution. The organic layer was separated and washed with bicarbonate, dried with $MgSO_4$, and concentrated *in vacuo*. The crude product was purified via column chromatography (5% ethyl acetate in hexanes) to give the product (0.520 g, 79% yield) as a white solid.

^{1}H NMR: (600MHz ,$CDCl_3$) 7.84 - 7.79 (m, 1 H), 7.78 - 7.72 (m, 1 H), 7.63 (s, 1 H), 1.61 (s, 9 H)
^{13}C NMR: (151MHz ,$CDCl_3$) 169.9, 137.2 (q, J = 0.02 Hz), 134.0 (q, *J* = 0.22 Hz), 129.6 (q, *J* = 0.22 Hz), 127.4 (q, *J* = 0.03 Hz), 126.0 (q, *J* = 0.02 Hz), 124.3 (q, *J* = 0.02 Hz), 122.9 (qd, *J* = 1.81, 0.03 Hz), 65.4, 28.2
^{19}F NMR: (376MHz ,$CDCl_3$) -60.342, -63.229
HRMS: calculated for $[C_{13}H_{12}ClF_6NO+H]^+$ = 348.0584, found = 348.0584

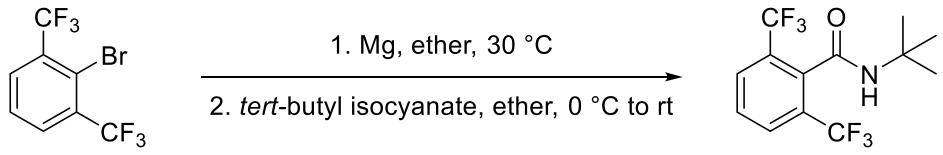

S5

***N*-(*tert*-butyl)-2,6-bis(trifluoromethyl)benzamide (S5):** To a flame dried 1 L round bottom flask was charged with magnesium turnings (1.66 g, 68.3 mmol) in the glovebox. To the reaction 20.0 mL ether and a few drops of iodine in 1,2-dibromoethane was added to activate the magnesium. Then 2,6-bis(trifluoromethyl)bromobenzene (20.0 g, 68.3 mmol) was dissolved in 48.0 mL ether and added to the reaction. The reaction was heated to 30 °C. The reaction was allowed to stir until all the magnesium was consumed. The reaction was cooled to 0 °C and 204 mL of ether was added to the reaction. Then *tert*-butyl isocyanate (7.10 mL, 62.1 mmol) was added. The reaction was allowed to warm to room temperature and stir overnight. The reaction was quenched with ammonium chloride solution and extracted with ethyl acetate, dried with $MgSO_4$, and concentrated *in vacuo*. The crude product was purified via column chromatography (10% ethyl acetate in hexanes) to give the product (18.5 g, 95% yield) as a white solid.

^{1}H NMR: (600MHz ,$CDCl_3$) 7.88 (d, *J* = 8.1 Hz, 2 H), 7.62 (t, *J* = 7.9 Hz, 1 H), 5.53 (br. s., 1 H), 1.44 (s, 9 H)
^{13}C NMR: (151MHz ,$CDCl_3$) 162.8, 135.0 (q, *J* = 0.01 Hz), 129.9 (q, *J* = 0.03 Hz), 129.3, 129.0 (q, *J* = 0.22 Hz), 123.1 (q, *J* = 1.82 Hz), 52.7, 28.1
^{19}F NMR: (376MHz ,$CDCl_3$) -58.658
HRMS: calculated for $[C_{13}H_{13}F_6NO+H]^+$ = 314.0974, found = 314.0973

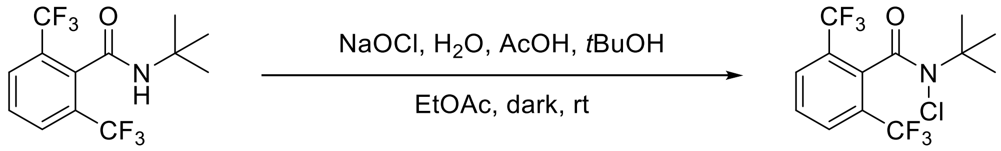

S5 **6**

***N*-(*tert*-butyl)-*N*-chloro-2,6-bis(trifluoromethyl)benzamide (6):** To a foil wrapped 5 L round bottom flask was charged with *N*-(*tert*-butyl)-2,6-bis(trifluoromethyl)benzamide (15.0 g, 47.9 mmol) and *tert*-butanol (30.0 mL, 311.5 mmol) and dissolved in 1.00 L ethyl acetate. To a foil wrapped addition funnel was added sodium hypochlorite (543 mL, 814 mmol), water (324 mL, 18.0 mmol), and glacial acetic acid (216 mL, 3,780 mmol). The contents of the addition funnel were added slowly to the reaction over 2 hours. The reaction was allowed to stir for 8 hours. The reaction was diluted with DCM and quenched with bicarbonate solution. The organic layer was separated and washed with bicarbonate, dried with $MgSO_4$, and concentrated *in vacuo*. The crude product was purified via column chromatography (5% ethyl acetate in hexanes) to give the product (14.4 g, 86% yield) as a white solid.

^{1}H NMR: (600MHz ,$CDCl_3$) 7.89 (d, *J* = 8.1 Hz, 2 H), 7.63 (t, *J* = 8.1 Hz, 1 H), 1.60 (s, 9 H)
^{13}C NMR: (151MHz ,$CDCl_3$) 167.0, 134.4 (q, *J* = 0.02 Hz), 130.1 (q, *J* = 0.03 Hz), 129.2, 127.7 (q, *J* = 0.21 Hz), 123.1 (q, *J* = 1.82 Hz), 65.3, 65.5, 28.1
^{19}F NMR: (376MHz ,$CDCl_3$) -59.708
HRMS: calculated for $[C_{13}H_{12}ClF_6NO+H]^+$ = 348.0584, found = 348.0583

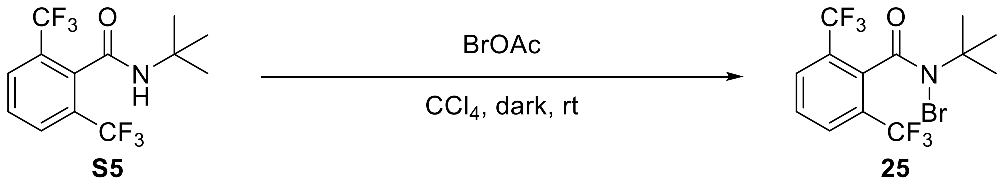

***N*-bromo-*N*-(*tert*-butyl)-2,6-bis(trifluoromethyl)benzamide (25):** To a foil wrapped flame dried 25 mL round bottom flask was charged with *N*-(*tert*-butyl)-2,6-bis(trifluoromethyl)benzamide (200 mg, 0.640 mmol) and acetyl hypobromite solution in carbontetrachloride (4.00 mL, 0.960 mmol) freshly prepared as described by us previously.[2]The reaction was monitored by ^{1}H NMR. The reaction was allowed to stir for 72 hours adding multiple additions of 4.00 mL aliquots of hypobromite solution after 18 hours, 42 hours, and 64 hours. The reaction was concentrated *in vacuo*. After concentrating, starting material was still observed by ^{1}H NMR. The crude product was resubjected to the reaction conditions and stirred for 8 hours. Hypobromite solution was added in 4.00 mL aliquots initially, after 3 hours, and 5 hours. The reaction was concentrated *in vacuo*. The product (179 mg, 71% yield) was obtained as a yellow solid without further purification.

^{1}H NMR: (600MHz ,$CDCl_3$) 7.88 (d, i = 8.1 Hz, 2 H), 7.61 (t, i = 8.1 Hz, 1 H), 1.59 (s, 9 H)
^{13}C NMR: (151MHz ,$CDCl_3$) 68.1, 135.6 (q, *J* = 0.02 Hz), 130.1 (q, *J* = 0.03 Hz), 129.1, 127.7 (q, *J* = 0.21 Hz), 123.1 (q, *J* = 1.82 Hz), 65.5, 29.0
^{19}F NMR: (376MHz ,$CDCl_3$) -58.658, -59.575
HRMS: calculated for $[C_{13}H_{12}BrF_6NO+H]^+$ = 392.0079, found = 392.0079

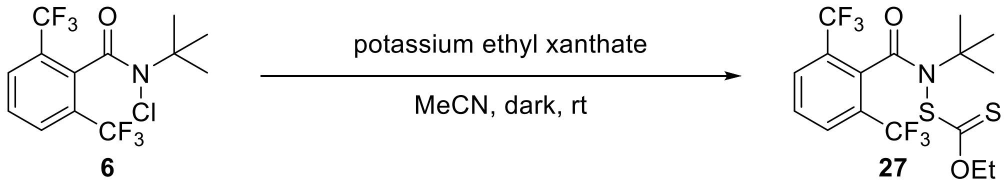

***N*-(*tert*-butyl)-*N*-((ethoxycarbonothioyl)thio)-2,6-bis(trifluoromethyl)benzamide (27):** To a foil wrapped 1 L round bottom flask was charged with potassium ethyl xanthate (1.15 g, 7.20 mmol) and dissolved in 250 mL MeCN. To the reaction a solution of chloroamide **6** (2.50 g, 7.20 mmol) in 100 mL MeCN. The reaction was allowed to stir overnight. The reaction was concentrated *in vacuo*. The crude product was diluted with DCM/ water (1:1). The organic layer was separated and washed with brine and dried with $MgSO_4$, and concentrated *in vacuo*. The crude product was purified via column chromatography (2.5% ethyl acetate in hexanes) to give the product (0.970 g, 31% yield) as a yellow solid.

^{1}H NMR: (600MHz ,$CDCl_3$) 7.87 (d, *J* = 7.7 Hz, 1 H), 7.80 (d, *J* = 8.1 Hz, 1 H), 7.61 (t, *J* = 7.9 Hz, 1 H), 4.79 (qd, *J* = 7.2, 10.6 Hz, 1 H), 4.51 (qd, *J* = 7.2, 10.8 Hz, 1 H), 1.60 (s, 9 H), 1.53 (t, *J* = 7.2 Hz, 3 H)
^{13}C NMR: (151MHz ,$CDCl_3$) 211.8, 168.7, 134.4 (q, *J* = 0.02 Hz), 131.0 (q, *J* = 0.03 Hz), 130.2 (q, *J* = 0.03 Hz), 129.2, 127.0 (quint, *J* = 0.21 Hz), 123.2 (qd, *J* = 1.82, 0.22 Hz), 70.8, 64.4, 64.3, 28.5, 13.5
^{19}F NMR: (376MHz ,$CDCl_3$) -57.517, -59.063
HRMS: calculated for $[C_{16}H_{17}F_6NO_2S_2+H]^+$ = 434.0678, found = 434.0676

Substrate Synthesis

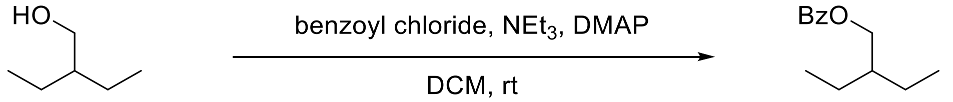

11

2-ethylbutyl benzoate (11): To a flame dried 500 mL round bottom flask was charged with DMAP (0.600 g, 4.90 mmol) and dissolved in 120 mL DCM was added 2-ethylbutanol (3.00 mL, 24.4 mmol) and trimethylamine (5.10 mL, 36.6 mmol). The reaction was cooled to 0 °C. Benzoyl chloride (3.40 mL, 29.3 mmol) was then added dropwise. The reaction was allowed to warm to room temperature and stir overnight. The reaction was quenched with water and extracted with DCM, dried with $MgSO_4$, and concentrated *in vacuo*. The crude product was purified via column chromatography (5% ethyl acetate in hexanes) to give the product (4.64 g, 92% yield) as a clear oil.

^{1}H NMR: (600MHz ,$CDCl_3$) 8.10 - 7.99 (m, 2 H), 7.61 - 7.53 (m, 1 H), 7.49 - 7.39 (m, 2 H), 4.33 (t, *J* = 6.4 Hz, 2 H), 3.44 (t, *J* = 6.8 Hz, 2 H), 2.00 - 1.89 (m, 2 H), 1.86 - 1.76 (m, 2 H), 1.67 - 1.57 (m, 2 H)
^{13}C NMR: (151MHz ,$CDCl_3$) 166.6, 132.9, 130.3, 129.5, 128.3, 64.6, 33.5, 32.3, 27.9, 24.7
HRMS: calculated for $[C_{13}H_{18}O_2+H]^+$ = 207.1380, found = 207.1378

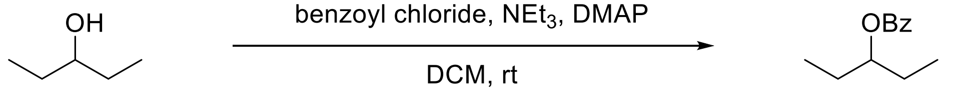

12

3-pentyl benzoate (12): To a flame dried 500 mL round bottom flask was charged with DMAP (0.680 g, 5.50 mmol) and dissolved in 140 mL DCM was added 3-pentanol (3.00 mL, 27.7 mmol) and trimethylamine (5.80 mL, 41.6 mmol). The reaction was cooled to 0 °C. Benzoyl chloride (3.90 mL, 33.3 mmol) was then added dropwise. The reaction was allowed to warm to room temperature and stir overnight. The reaction was quenched with water and extracted with DCM, dried with $MgSO_4$, and concentrated *in vacuo*. The crude product was purified via column chromatography (5% ethyl acetate in hexanes) to give the product (5.17 g, 97% yield) as a yellowish oil.

^{1}H NMR: (600MHz ,$CDCl_3$) 8.12 - 7.98 (m, 2 H), 7.65 - 7.51 (m, 1 H), 7.51 - 7.41 (m, 2 H), 5.02 (t, *J* = 6.2 Hz, 1 H), 1.83 - 1.63 (m, 4 H), 0.96 (s, 6 H)
^{13}C NMR: (151MHz ,$CDCl_3$) 166.4, 132.7, 130.8, 129.5, 128.3, 77.3, 26.5, 9.7
HRMS: calculated $[C_{12}H_{16}O_2+H]^+$ = 193.1223, found = 193.1222

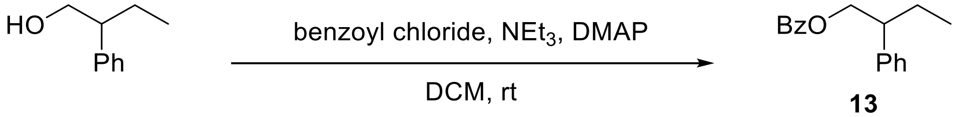

13

2-phenylbutyl benzoate (13): To a flame dried 250 mL round bottom flask was charged with DMAP (0.330 g, 2.70 mmol) and dissolved in 67.0 mL DCM was added 2-phenylbutanol (2.10 mL, 13.4 mmol) and trimethylamine (2.80 mL, 20.1 mmol). The reaction was cooled to 0 °C. Benzoyl chloride (1.90 mL, 16.1

mmol) was then added dropwise. The reaction was allowed to warm to room temperature and stir overnight. The reaction was quenched with water and extracted with DCM, dried with $MgSO_4$, and concentrated *in vacuo*. The crude product was purified via column chromatography (5% ethyl acetate in hexanes) to give the product (3.30 g, 97% yield) as a clear oil.

^{1}H NMR: (600MHz ,$CDCl_3$) 8.01 (d, J = 8.4 Hz, 2 H), 7.59 - 7.53 (m, 1 H), 7.46 - 7.42 (m, 2 H), 7.39 - 7.34 (m, 2 H), 7.32 - 7.25 (m, 3 H), 4.54 - 4.45 (m, 2 H), 3.06 - 2.98 (m, 1 H), 1.99 - 1.90 (m, 1 H), 1.81 - 1.71 (m, 1 H), 0.92 (t, J = 7.3 Hz, 3 H)
^{13}C NMR: (151MHz ,$CDCl_3$) 166.4, 141.8, 132.8, 130.2, 129.5, 128.4, 128.3, 127.9, 126.6, 68.7, 46.8, 25.3, 11.8
HRMS: calculated for $[C_{17}H_{18}O_2+H]^+$ = 255.1380, found = 255.1377

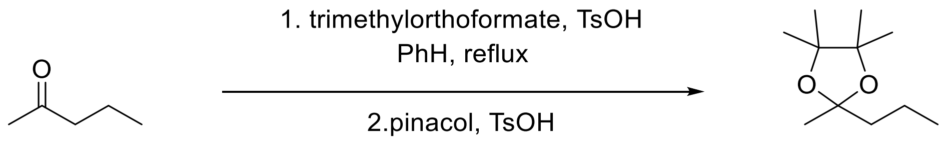

14

2,4,4,5,5-pentamethyl-2-propyl-1,3-dioxolane (14): To a flame dried 100 mL round bottom flask was charged with *p*-toluene sulfonic acid (89.0 mg, 0.470 mmol) and dissolved in 40.0 mL benzene was added trimethylorthoformate (12.0 mL, 113 mmol) and 2-pentanone (5.00 mL, 47.0 mmol). The reaction was heated to reflux and allowed to stir overnight. The reaction was cooled to room temperature, washed with bicarbonate solution, dried with $MgSO_4$, and concentrated *in vacuo*. The crude product was then added to a flame dried 100 mL round bottom flask charged with *p*-toluene sulfonic acid (89.0 mg, 0.470 mmol) and pinacol (2.99 g, 28.2 mmol) dissolved in 40.0 mL chloroform. The reaction was heated to reflux and allowed to stir overnight. The reaction was then neutralized with bicarbonate solution, dried with $MgSO_4$, and concentrated *in vacuo*. The crude product was purified via column chromatography (10% ethyl acetate in hexanes) to give the product (1.89 g, 36% yield) as a clear oil.

^{1}H NMR: (600MHz ,$CDCl_3$) 1.64 - 1.60 (m, 2 H), 1.45 - 1.39 (m, 1 H), 1.38 (s, 3 H), 1.25 (s, 6 H), 1.23 (s, 6 H), 0.92 (t, J = 7.5 Hz, 3 H)
^{13}C NMR: (151MHz ,$CDCl_3$) 107.6, 82.3, 45.4, 26.9, 24.8, 24.7, 18.4, 14.6
HRMS: calculated for $[C_{11}H_{22}O_2+H]^+$ = 187.1693, found = 187.1694

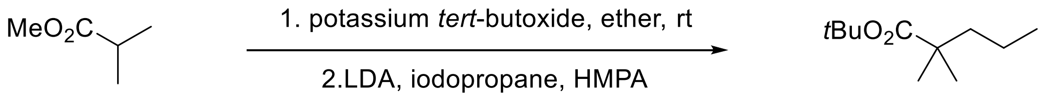

15

***tert*-butyl-2,2-dimethyl pentanoate (15):** To a flame dried 250 mL round bottom flask was charged with potassium *tert*-butoxide (5.90 g, 52.3 mmol) and dissolved in 175 mL ether was added methyl isobutyrate (5.00 mL, 436 mmol). The reaction was allowed to stir for 3 hours. The reaction was filtered through alumina and concentrated *in vacuo*. The crude product was taken on without further purification. A 250 mL round bottom flask was flamed dried and charged with diisopropylamine (2.68 mL, 19.1 mmol) and dissolved in 40.0 mL THF. The reaction was cooled to -78 °C. Then *n*-butyl lithium (7.95 mL, 19.1 mmol)

added dropwise. The reaction was allowed to stir for 30 minutes. Iodopropane (1.77 mL, 18.2 mmol) and HMPA (3.17 mL, 18.2 mL) were added simultaneously and the reaction was allowed to warm to room temperature and stir overnight. The reaction was then neutralized with 1 M HCl solution. The aquoues layer was extracted with ether. The combined organic layers were washed with brine, dried with $MgSO_4$, and concentrated *in vacuo*. The crude product was purified via column chromatography (20% ether in pentane) to give the product (1.49 g, 44% yield) as a yellowish oil.

^{1}H NMR:(600MHz ,$CDCl_3$) 1.46 - 1.43 (m, 2 H), 1.42 (s, 9 H), 1.27 - 1.21 (m, 2 H), 1.10 (s, 6 H), 0.88 (t, J = 7.2 Hz, 3 H)
^{13}C NMR: (151MHz ,$CDCl_3$) 177.5, 79.5, 43.2, 42.7, 28.0, 25.2, 18.2, 14.7
HRMS: calculated for $[C_{11}H_{22}O_2+H]^+$ = 187.1693, found = 187.1692

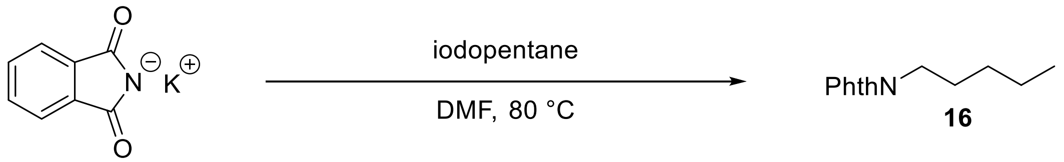

16

***N*-*n*-pentyl phthalimide (16):** was prepared according to a literature procedure, and spectral data were in accordance with the literature values.[2]

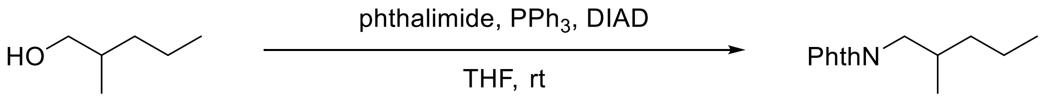

17

***N*-(2-methyl pentyl) phthalimide (17):** was prepared according to a literature procedure, and spectral data were in accordance with the literature values.[1]

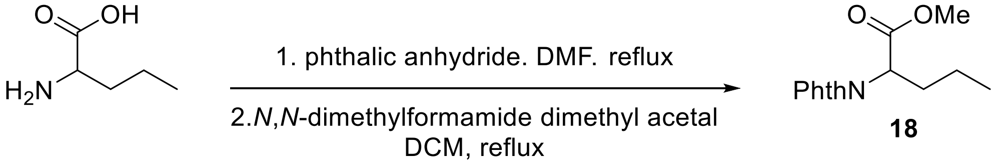

18

***N*-phthaloylnorvaline methyl ester (18):** Prepared via a 2-step procedure. To a flame dried 100 mL round bottom flask was charged with norvaline (1.00 g, 8.50 mmol), phthalic anhydride (1.90 g, 12.8 mmol) and dissolved in 24 mL DMF was heated to reflux and stirred overnight. The reaction was allowed to cool to room temperature. The reaction was diluted with ether, washed with water, and concentrated *in vacuo*. The crude reaction was taken on without further purification. To a flame dried 25 mL round bottom flask was charged *N*- phthaloylnorvaline norvaline (1.00 g, 4.00 mml), dissolved in 13 mL DCM, and was added *N*,*N*-dimethylforamide dimethyl acetal (1.10 mL, 8.00 mmol). The reaction was heated to reflux and allowed to stir overnight. The reaction was allowed to cool to room temperature and was diluted with ether, washed with saturated bicarbonate, washed with saturated ammonium chloride, washed with brine, dried with $MgSO_4$, and concentrated *in vacuo*. The crude product was

purified via column chromatography (20% ethyl acetate in hexanes) to give the product (0.745 g, 71% yield) as a clear oil.

^{1}H NMR: (600MHz ,$CDCl_3$) 7.90 - 7.82 (m, 2 H), 7.77 - 7.70 (m, 2 H), 4.86 (dd, *J* = 4.6, 11.2 Hz, 1 H), 3.72 (s, 3 H), 2.32 - 2.21 (m, 1 H), 2.21 - 2.11 (m, 1 H), 1.38 - 1.26 (m, 2 H), 0.92 (t, *J* = 7.3 Hz, 3 H)
^{13}C NMR: (151MHz ,$CDCl_3$) d = 169.9, 167.7, 134.1, 131.8, 123.6, 52.6, 51.8, 30.6, 19.5, 13.3
HRMS: calculated $[C_{14}H_{15}NO_4+H]^+$ = 262.10738, found = 262.10656

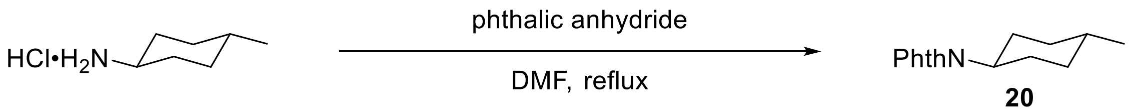

20

***trans*-4-methylcyclohexyl phthalimide (20):** To a flame dried 100 mL round bottom flask was charged with *tran*-4-methylcyclohexylamine hydrochloride (1.50 g, 10.0 mmol), phthalic anhydride (2.20 g, 15.0 mmol) and dissolved in 29 mL DMF was heated to reflux and stirred overnight. The reaction was allowed to cool to room temperature. The reaction was diluted with ether, washed with water, and concentrated *in vacuo*. The crude product was purified via column chromatography (20% ethyl acetate in hexanes) to give the product (1.04 g, 43% yield) as a white solid.

^{1}H NMR: (600MHz ,$CDCl_3$) 7.89 - 7.76 (m, 2 H), 7.73 - 7.63 (m, 2 H), 4.09 (tt, *J* = 3.9, 12.4 Hz, 1 H), 2.27 (dq, *J* = 3.5, 12.8 Hz, 2 H), 1.88 - 1.77 (m, 2 H), 1.77 - 1.64 (m, 2 H), 1.57 - 1.41 (m, 1 H), 1.17 - 0.99 (m, 2 H), 0.93 (d, *J* = 6.6 Hz, 3 H)
^{13}C NMR: (151MHz ,CDCl3) 168.5, 133.7, 132.1, 123.0, 50.7, 34.5, 31.5, 29.5, 22.2
HRMS: calculated $[C_{15}H_{17}NO_2+H]^+$ = 244.13321, found = 244.13244

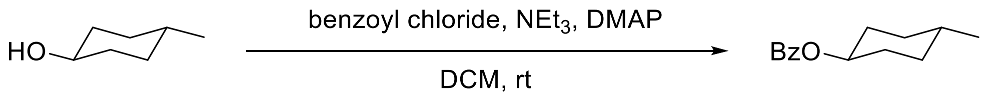

21

***trans*-4-methylcyclohexyl benzoate (21):** To a flame dried 250 mL round bottom flask was charged with DMAP (0.390 g, 3.20 mmol) and dissolved in 80 mL DCM was added *trans*-4-methylcyclohexanol (2.00 mL, 16.0 mmol) and trimethylamine (3.35 mL, 24.0 mmol). The reaction was cooled to 0 °C. Benzoyl chloride (2.25 mL, 19.2 mmol) was then added dropwise. The reaction was allowed to warm to room temperature and stir overnight. The reaction was quenched with water and extracted with DCM, dried with $MgSO_4$, and concentrated *in vacuo*. The crude product was purified via column chromatography (5% ethyl acetate in hexanes) to give the product (3.06 g, 88% yield) as a white solid.

^{1}H NMR: (600MHz ,$CDCl_3$) 8.05 (dd, J = 0.9, 8.3 Hz, 12 H), 7.57 - 7.53 (m, 6 H), 7.46 - 7.41 (m, 12 H), 4.92 (tt, J = 4.4, 11.1 Hz, 6 H), 2.12 - 2.06 (m, 12 H), 1.83 - 1.76 (m, 12 H), 1.56 - 1.47 (m, 13 H), 1.46 - 1.39 (m, 6 H), 1.17 - 1.07 (m, 12 H), 0.93 (d, J = 6.6 Hz, 18 H)
^{13}C NMR: (151MHz ,$CDCl_3$) 166.1, 132.6, 130.9, 129.5, 128.2, 74.0, 33.0, 31.7, 31.7, 21.9
HRMS: calculated for $[C_{14}H_{18}O_2+H]^+$ = 219.13796, found = 219.13797

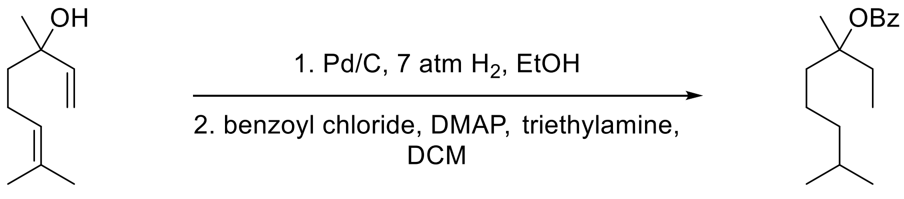

24

tetrahydrolinalool benzoate (24): Prepared via a 2-step procedure. To a flame dried 10 mL Ace Glass pressure tube was charged 10% palladium on carbon (0.180g, 1.70 mmol) 5 mL ethanol, and linalool (3.00 mL, 16.7 mmol). The tube was sealed with a Swagelok gas quick-connect adapter[3] and pressurized with 7 atm of H_2. The reaction was allowed to stir over 3 days, monitored by HNMR, and repressurized multiple times. The crude reaction was diluted with DCM and ran through a celtie plug, concentrated *in vacuo*, diluted with DCM and ran through a silica plug. The reaction was taken on without further purification. To a flame dried 25 mL round bottom flask was charged with DMAP (36.7 mg, 0.300 mmol) and dissolved in 10 mL pyridine was added tetrahydrolinalool (1.00 g, 6.30 mmol). To the reaction benzoyl chloride (2.22 mL, 19.0 mmol) was added dropwise was allowed to stir overnight. The reaction was diluted with hexanes, washed with water, dried with $MgSO_4$, and concentrated *in vacuo*. The crude product was purified via column chromatography (5% ethyl acetate in hexanes) to give the product (1.55 g, 94% yield) as a clear oil.

^{1}H NMR: (500MHz ,CHLOROFORM-d) 8.02 - 7.96 (m, 2 H), 7.56 - 7.49 (m, 1 H), 7.45 - 7.36 (m, 2 H), 2.09 - 1.77 (m, 4 H), 1.60 - 1.49 (m, 4 H), 1.40 - 1.30 (m, 2 H), 1.24 - 1.16 (m, 2 H), 0.93 (t, J = 7.5 Hz, 3 H), 0.87 (d, J = 6.4 Hz, 6 H)
^{13}C NMR: (126MHz ,CHLOROFORM-d) 165.5, 132.5, 132.0, 129.4, 128.2, 85.9, 39.3, 38.2, 31.1, 27.8, 23.4, 22.6, 21.3, 8.1
HRMS: calculated $[C_{17}H_{26}O_2+H]^+$ = 263.20056, found = 263.10990

Standard Synthesis

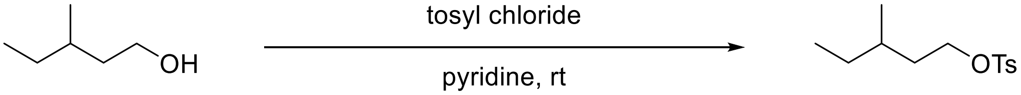

S6

3-methyl pentyl tosylate (S6): To a flame dried 100 mL round bottom flask was charged with tosyl chloride (7.96 g, 41.7 mmol) and dissolved in 21.0 mL pyridine. The reaction was cooled to 0 °C. To the reaction 3-methyl pentyl pentanol (4.00 mL, 32.1 mmol). The reaction was allowed to warm to room temperature and stir overnight. The reaction was quenched with 1 M HCl solution and extracted with ether. The organic layer was washed with saturated $CuSO_4$ solution, saturated bicarbonate solution, brine, dried with $MgSO_4$, and concentrated *in vacuo*. The crude product was purified via column chromatography (5% ethyl acetate in hexanes) to give the product (3.72 g, 45% yield) as a clear oil.

^{1}H NMR: (600MHz ,$CDCl_3$) 7.84 - 7.72 (m, 2 H), 7.35 (d, *J* = 8.1 Hz, 2 H), 4.14 - 3.96 (m, 2 H), 2.45 (s, 3 H), 1.74 - 1.62 (m, 1 H), 1.49 - 1.35 (m, 2 H), 1.32 - 1.20 (m, 1 H), 1.15 - 1.03 (m, 1 H), 0.83 - 0.78 (m, 6 H)

^{13}C NMR: (151MHz ,CDCl$_3$) 144.6, 133.1, 129.8, 127.9, 69.1, 35.2, 30.6, 29.1, 21.6, 18.6, 11.1
HRMS: calculated for $[C_{13}H_{20}O_3S+H]^+$ = 257.1206, found = 257.1318

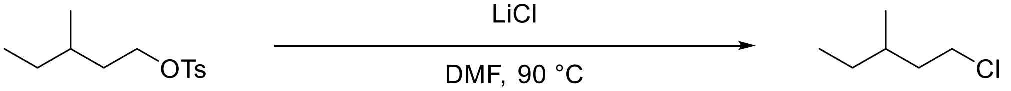

S6 **S7**

1-chloro-3-methylpentane (S7): A flame dried 50 mL round bottom flask was charged with lithium chloride (1.49 g, 35.1 mmol), dissolved in 12.0 mL DMF, and added 3-methylpentyl tosylate (3.00 g, 11.7 mmol). The reaction was heated to 90 °C. The reaction was allowed to stir overnight. The reaction was diluted with ether and washed twice with 1 M HCl solution. The organic layer was dried with $MgSO_4$, and concentrated *in vacuo*. The product (0.210 g, 15% yield) was given as a pale yellow oil.

^1H NMR: (600MHz ,CDCl$_3$) 3.65 - 3.53 (m, 2 H), 1.87 - 1.77 (m, 1 H), 1.66 - 1.55 (m, 2 H), 1.43 - 1.34 (m, 1 H), 1.25 - 1.16 (m, 1 H), 0.95 - 0.86 (m, 6 H)
^{13}C NMR: (151MHz ,CDCl$_3$) 43.4, 39.3, 31.9, 29.1, 18.5, 11.2
HRMS: calculated for $[C_6H_{12}Cl]^-$ = 119.0628, found = 191.0623

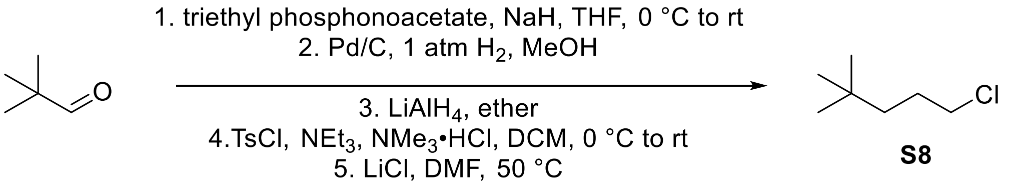

S8

1-chloro-4,4-dimethylpentane (S8): Prepared via a 5-step procedure. To a flame dried 250 mL round bottom flask was charged with sodium hydride (1.56 g, 64.8 mmol) and dissolved in 47.0 mL THF. The reaction was cooled to 0 °C. To the reaction triethyl phosphonoacetate (15.0 mL, 75.6 mmol) was added and allowed to stir for 15 minutes, then pivaldehyde (5.87 mL, 54.0 mmol) was added in 45.0 mL THF. The reaction was allowed to stir overnight and warm to room temperature. The reaction was quenched with saturated ammonium chloride, extracted with ethyl acetate, washed with brine, and dried with $MgSO_4$, and concentrated *in vacuo*. The crude product was taken on without further purification. To a flame dried 100 mL round bottom flask was charged with palladium on carbon (0.480 g, 4.50 mmol) and dissolved in 22.0 mL methanol. To the reaction ethyl (*E*)-4,4-dimethyl-2-pentenoate (7.00 g, 44.8 mmol) was added and allowed to stir overnight. The reaction was quenched with saturated ammonium chloride, extracted with ethyl acetate, washed with brine, and dried with $MgSO_4$, and concentrated *in vacuo*. The crude product was taken on without further purification. To a flame dried 250 mL round bottom was charged lithium aluminum hydride (0.360 g, 9.50 mmol) and dissolved in 38.0 mL ether. The reaction was cooled to 0 °C and added ethyl 4,4-dimethylpentanoate (1.20 g, 7.60 mmol) in 32.0 mL ether. The reaction was allowed to stir overnight. The reaction was quenched with 1.00 mL water, 2.50 mL 15% sodium hydroxide solution, and 2.50 mL water, then filtered through celite, dried with $MgSO_4$, and concentrated *in vacuo*. The crude product was taken on without further purification. To a flame dried 50 mL round bottom was charged tosyl chloride (0.600 g, 5.20 mmol), trimethylamine hydrochloride (49.7 mg, 0.520 mmol), and dissolved in 5.00 mL DCM. The reaction was cooled to 0 °C and added trimethylamine (1.81 mL, 13.0 mmol) and 4,4-dimethylpentanol (0.600 g, 5.20 mmol) in 5.00 mL DCM. The reaction was allowed to stir for 1 hour and quenched with *N,N*-dimethyl-1,3-propanediamine (1.30 mL, 10.4 mmol) and allowed to stir for 15 minutes. The reaction was diluted with water, washed with 1 M HCl, and dried with $MgSO_4$, and

concentrated *in vacuo*. The crude product was taken on without further purification. To a flame dried 25 mL round bottom was charged with 4,4-dimethylpentyl tosylate (1.00 g, 3.70 mmol), dissolved in 7.50 mL DMF, and added lithium chloride (0.470 g, 11.1 mmol). The reaction was heated to 50 °C and allowed to stir overnight. The reaction was diluted with pentane and washed with water. The organic layer was filtered through a silica plug with pentane and concentrated *in vacuo*. The product (79.1 mg, 16% yield) was obtained as a clear oil.

^{1}H NMR: (600MHz ,$CDCl_3$) 3.51 (t, *J* = 6.8 Hz, 2 H), 1.79 - 1.70 (m, 2 H), 1.33 - 1.26 (m, 2 H), 0.89 (s, 9 H)
^{13}C NMR: (151MHz ,$CDCl_3$) 46.1, 41.3, 30.1, 29.3, 28.2
HRMS: calculated for $[C_7H_{14}Cl]^-$ = 133.0790, found = 133.0779

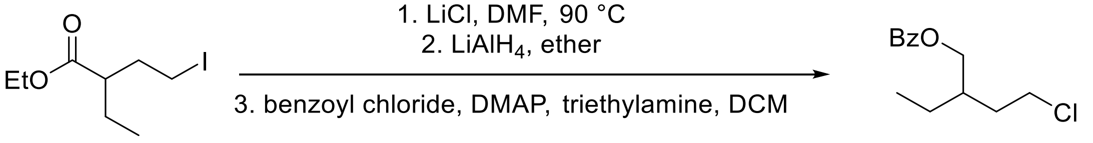

S9 **S10**

4-chloro-2-ethylbutyl benzoate (S10): Prepared via a 3-step procedure. Ethyl 2-ethyl-4-iodobutanoate **S9** was prepared as outlined by Bach.[4] To a flame dried 250 mL round bottom was charged with ethyl 2-ethyl-4-iodobutanoate **S9** (3.00 g, 11.1 mmol), dissolved in 11.0 mL DMF, and added lithium chloride (1.40 g, 33.3 mmol). The reaction was heated to 90 °C and allowed to stir overnight. The reaction was diluted with ether and washed with 1 M HCl, dried with $MgSO_4$, and concentrated *in vacuo*. The crude product was taken on without further purification. To a flame dried 250 mL round bottom was charged lithium aluminum hydride (0.400 g, 10.5 mmol) and dissolved in 42.0 mL ether. The reaction was cooled to 0 °C and added ethyl 4-chloro-2-ethylbutanoate (1.50 g, 8.40 mmol) in 35.0 mL ether. The reaction was allowed to stir overnight. The reaction was quenched with 1.00 mL water, 2.50 mL 15% sodium hydroxide solution, and 2.50 mL water, then filtered through celite, dried with $MgSO_4$, and concentrated *in vacuo*. The crude product was taken on without further purification. To a flame dried 50 mL round bottom flask was charged with DMAP (71.0 mg, 0.600 mmol) and dissolved in 15.0 mL DCM. The reaction was cooled to 0 °C and added triethylamine (0.600 mL, 4.40 mmol), 4-chloro-2-ethylbutanol (0.40 g, 2.90 mmol), and benzoyl chloride (0.410 mL, 3.50 mmol). The reaction was allowed to stir overnight. The reaction was quenched with water, extracted with DCM, dried with $MgSO_4$, and concentrated *in vacuo*. The product (0.590 g, 84% yield) was obtained as a clear oil.

^{1}H NMR: (600MHz ,$CDCl_3$) 8.06 - 8.01 (m, 2 H), 7.60 - 7.55 (m, 1 H), 7.48 - 7.42 (m, 2 H), 3.65 (t, *J* = 7.0 Hz, 2 H), 2.04 - 1.97 (m, 1 H), 1.92 (dq, *J* = 7.3, 14.1 Hz, 2 H), 1.54 - 1.46 (m, 2 H), 0.99 (t, *J* = 7.5 Hz, 3 H)
^{13}C NMR: (151MHz ,$CDCl_3$) 166.6, 133.0, 130.1, 129.5, 128.4, 66.4, 42.9, 36.6, 34.3, 23.7, 11.1
HRMS: calculated for $[C_{13}H_{17}ClO_2+H]^+$ = 241.0990 , found = 241.0989

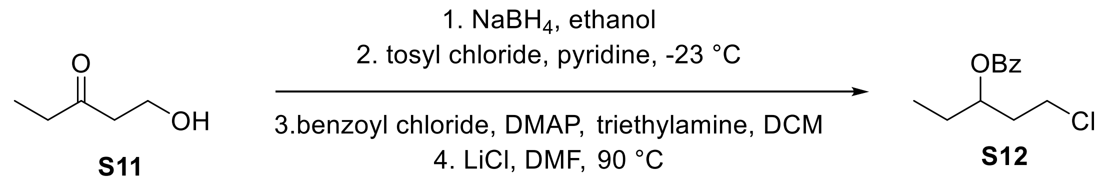

1-chloro-3-pentanyl benzoate (S12): Prepared via a 4-step procedure. 1-hydroxy-3-pentanone **S11** was prepared as outlined by Albizati.[5] To a flame dried 250 mL round bottom flask was charged with 1-hydroxy-3-pentanone **S11** (800.0 mg, 7.80 mmol) and dissolved in 58.0 mL ethanol. The reaction was cooled to 0 °C and added sodium borohydride (178 mg, 4.70 mmol) in multiple portions over 5 minutes and allowed to stir for 3 hours. The reaction was quenched with 1.10 mL acetic acid added dropwise and allowed to stir for 30 minutes. The reaction was diluted with ethyl acetate washed with 5% bicarbonate solution. The aqueous layer was saturated with sodium chloride and extracted with ether, dried with $MgSO_4$, and concentrated *in vacuo*. The crude reaction was taken on without further purification. To a flame dried 50 mL round bottom flask was charged with tosyl chloride (594 mg, 3.12 mmol) and dissolved in 4.40 mL pyridine. The reaction was cooled to -23 °C and added 1,3-pentandiol (400.0 mg, 3.12 mmol) in 4.40 mL pyridine. The reaction was allowed to stir for 45 minutes. The reaction was quenched with cold water and extracted with ether. The organic layer was washed with cold water, washed with cold 1 M HCl, washed again with cold water, and washed with cold saturated bicarbonate solution, dried with $MgSO_4$, and concentrated *in vacuo*. The crude reaction was taken on without further purification. To a flame dried 10 mL round bottom flask was charged with DMAP (11.0 mg, 0.0900 mmol) and dissolved in 2.20 mL DCM. The reaction was cooled to 0 °C and added triethylamine (90.6 µL, 0.650 mmol), 3-hydroxypentyl tosylate (0.110 g, 0.430 mmol), and benzoyl chloride (60.4 µL, 0.520 mmol). The reaction was allowed to stir overnight. The reaction was quenched with water, extracted with DCM, dried with $MgSO_4$, and concentrated *in vacuo*. The crude product was taken on without further purification. To a flame dried 10 mL round bottom was charged with 1-(tosyloxy)-3-pentyl benzoate (84.0 mg, 0.230 mmol), dissolved in 0.500 mL DMF, and added lithium chloride (29.0 mg,0.690 mmol). The reaction was heated to 90 °C and allowed to stir overnight. The reaction was diluted with pentane and washed with water. The organic layer was filtered through a silica plug with pentane and concentrated *in vacuo*. The product (19.3 mg, 37% yield) was obtained as a clear oil.

^{1}H NMR: (600MHz ,$CDCl_3$) 8.07 - 8.03 (m, 2 H), 7.59 - 7.55 (m, 1 H), 7.48 - 7.43 (m, 2 H), 5.28 - 5.22 (m, 1 H), 3.64 - 3.55 (m, 2 H), 2.25 - 2.17 (m, 1 H), 2.16 - 2.09 (m, 1 H), 1.80 - 1.73 (m, 2 H), 0.98 (t, J = 7.3 Hz, 3 H)
^{13}C NMR: (151MHz ,$CDCl_3$) 166.1, 133.0, 130.2, 129.6, 128.4, 73.3, 40.9, 36.8, 27.2, 9.5
HRMS: calculated for $[C_{12}H_{15}ClO_2+H]^+$ = 227.0833, found = 227.0833

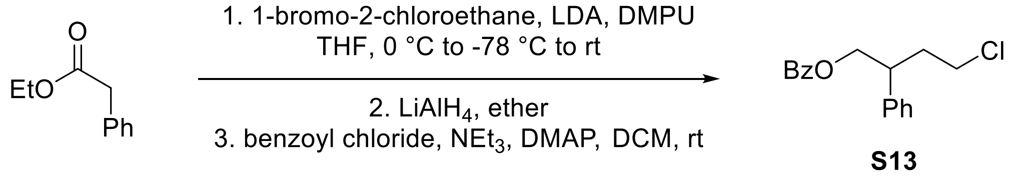

4-chloro-2-phenylbutyl benzoate (S13): Prepared via a 3-step procedure. To a flame dried 1 L round bottom flask was charged with diisopropylamine (2.90 mL, 20.7 mmol) and dissolved in 10.0 mL THF. The

reaction was cooled to 0 °C and added *n*-butyl lithium (9.60 mL, 20.7 mmol). The reaction was allowed to stir for 30 minutes and then cooled to -78 °C and added ethyl-2-phenyl acetate (2.70 mL, 18.8 mmol) in 20.0 mL THF. The reaction was allowed to stir for 30 minutes and added DMPU (2.30 mL, 18.8 mmol) in 20.0 mL THF and 1-bromo-2-chloroethane (3.14 mL, 37.6 mmol) in 40.0 mL THF. The reaction was allowed to warm to room temperature and stir overnight. The reaction was quenched with 1 M HCl extracted with ether. The organic layer was washed with brine, dried with $MgSO_4$, and concentrated *in vacuo*. The crude reaction was filtered through a silica plug and taken on without further purification. To a flame dried 250 mL round bottom flask was charged with lithium aluminum hydride (0.450 g, 11.8 mmol) and dissolved in 47.0 mL ether. The reaction was cooled to 0 °C and added ethyl 4-chloro-2-phenylbutanoate (2.00 g, 9.40 mmol) in 24.0 mL ether. The reaction was allowed to stir overnight. The reaction was quenched with 1.00 mL water, 2.50 mL 15% sodium hydroxide solution, and 2.50 mL water, then filtered through celite, dried with $MgSO_4$, and concentrated *in vacuo*. The crude product was taken on without further purification. To a flame dried 100 mL round bottom was charged DMAP (0.150 g, 1.20 mmol), 4-chloro-2-phenylbutan-1-ol (1.00 g, 5.90 mmol), and dissolved in 30.0 mL DCM. The reaction was cooled to 0 °C and added trimethylamine (1.24 mL, 8.90 mmol) and benzoyl chloride (0.83 mL, 7.10 mmol). The reaction was allowed to stir for 2 days and quenched with water and extracted with DCM. The organic layer was washed with 1 M HCl, and dried with $MgSO_4$, and concentrated *in vacuo*. . The crude product purified via column chromatography (10% ethyl acetate in hexanes) to give the product (0.630 g, 11% yield) as a clear oil.

^{1}H NMR: (600MHz ,$CDCl_3$) 8.01 - 7.96 (m, 2 H), 7.56 (tt, *J* = 1.2, 7.4 Hz, 1 H), 7.47 - 7.42 (m, 2 H), 7.39 - 7.34 (m, 2 H), 7.32 - 7.26 (m, 3 H), 4.52 - 4.44 (m, 2 H), 3.54 (ddd, *J* = 5.0, 6.6, 11.2 Hz, 1 H), 3.42 - 3.32 (m, 2 H), 2.34 (dddd, *J* = 4.8, 6.7, 9.0, 14.0 Hz, 1 H), 2.23 - 2.15 (m, 1 H)
^{13}C NMR: (151MHz ,$CDCl_3$) 166.4, 139.9, 133.0, 130.0, 129.5, 128.8, 128.4, 127.9, 127.3, 68.3, 42.6, 42.2, 35.2
HRMS: calculated for [C17H17ClO2+H]+ = 289.0990 , found = 289.0987

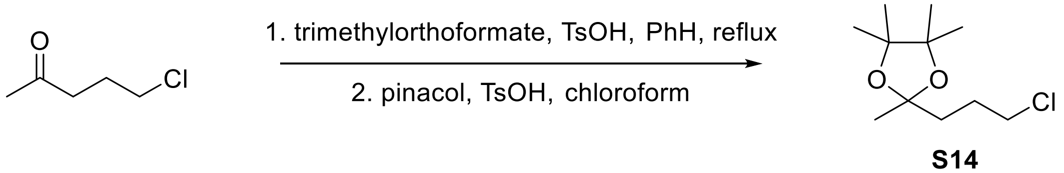

S14

2-(3-chloropropyl)-2,4,4,5,5-pentamethyl-1,3-dioxolane (S14): To a flame dried 100 mL round bottom flask was charged with *p*-toluenesulfonic acid (57.0 mg, 0.300 mmol) and dissolved in 22.0 mL benzene. To the reaction trimethylorthoformate (6.86 mL, 62.7 mmol) and 5-chloro-2-pentanone (3.00 mL, 26.1 mmol) was added and heated to reflux. The reaction was allowed to stir overnight. The reaction was cooled to room temperature and quenched with saturated bicarbonate. The organic layer was dried with $MgSO_4$, and concentrated *in vacuo*. To the crude reaction was added *p*-toluenesulfonic acid (50.0 mg, 0.260 mmol) and pinacol (1.67 g, 15.7 mmol) and dissolved in 31.0 mL chloroform. The reaction was heated to reflux and allowed to stir overnight. The reaction was cooled to room temperature and quenched with saturated bicarbonate. The organic layer was dried in $MgSO_4$ and concentrated *in vacuo*. The crude product purified via column chromatography (10% ethyl acetate in hexanes) to give the product (1.65 g, 29% yield) as a yellow oil.

^{1}H NMR: (600MHz ,$CDCl_3$) 1.64 - 1.60 (m, 2 H), 1.45 - 1.39 (m, 1 H), 1.38 (s, 3 H), 1.25 (s, 6 H), 1.23 (s, 6 H), 0.92 (t, J = 7.5 Hz, 3 H)
^{13}C NMR: (151MHz ,$CDCl_3$) 107.6, 82.3, 45.4, 26.9, 24.8, 24.7, 18.4, 14.6
HRMS: calculated for $[C_{11}H_{21}ClO_2+H]^+$ = 221.1303, found = 221.1304

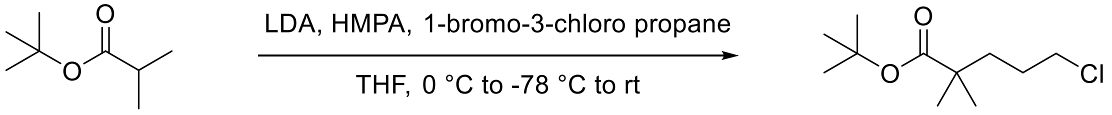

S15

***tert*-butyl 5-chloro-2,2-dimethylpentanoate (S15):** To a flame dried 100 mL round bottom flask was charged with diisopropylamine (1.02 mL, 7.30 mmol) and dissolved in 17.0 mL THF was cooled to 0 °C. To the reaction *n*-butyl lithium (2.90 mL, 7.30 mmol) was added and allowed to stir for 30 minutes. The reaction was cooled to -78 °C and added *tert*-butyl isobutyrate (1.00 g, 6.90 mmol) and was allowed to stir for 30 minutes. To the reaction 1-bromo-3-chloro propane (0.680 mL, 6.90 mmol) and HMPA (1.20 mL, 6.90 mmol) were added. The reaction was allowed to warm to room temperature and stir overnight. The reaction was quenched with 1 M HCl solution and extracted with ether three times. The organic layer was washed with brine, dried with $MgSO_4$, and concentrated *in vacuo*. The crude product was purified via column chromatography (10% ethyl acetate in hexanes) to give the product (0.610 g, 40% yield) as a clear oil.

^{1}H NMR: (600MHz ,$CDCl_3$) 3.51 (t, J = 6.6 Hz, 2 H), 1.76 - 1.69 (m, 2 H), 1.65 - 1.58 (m, 2 H), 1.43 (s, 9 H), 1.13 (s, 6 H)
^{13}C NMR: (151MHz ,$CDCl_3$) 176.8, 80.0, 45.5, 42.3, 37.9, 28.4, 28.0, 25.2
HRMS: calculated for $[C_{11}H_{22}ClO_2+H]^+$ = 221.1303, found = 221.1304

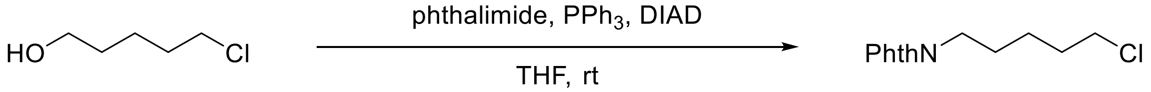

S16

5-chloropentyl phthalimide (S16): To a flame dried 500 mL round bottom was charged with phthalimide (3.78 g, 25.7 mmol), added triphenylphosphine (6.74 g, 25.7 mmol), and dissolved in 171 mL THF. To the reaction was added 5-chloropentanol (2.00 mL, 17.1 mmol) and DIAD (5.05 mL, 25.7 mmol) and allowed to stir overnight. The reaction concentrated *in vacuo*. The crude product was purified via column chromatography (20% ethyl acetate in hexanes) to give the product (3.13 g, 73% yield) as a pale yellow solid.

^{1}H NMR: (600MHz ,CHLOROFORM-d) 7.86 - 7.82 (m, 2 H), 7.73 - 7.69 (m, 2 H), 3.72 - 3.67 (m, 2 H), 3.53 (dt, J = 1.1, 6.6 Hz, 2 H), 1.85 - 1.79 (m, 2 H), 1.74 - 1.68 (m, 2 H), 1.49 (quin, J = 7.7 Hz, 2 H)
^{13}C NMR: (151MHz ,CHLOROFORM-d) 168.4, 133.9, 132.1, 123.2, 44.7, 37.7, 32.0, 27.9, 24.1
HRMS: calculated for $[C_{13}H_{14}ClNO_2+H]^+$ = 252.0786, found = 252.0783

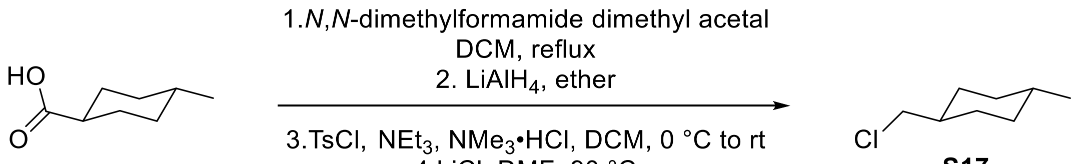

S17

***trans*-1-(chloromethyl)-4-methylcyclohexane (S17):** Prepared via a 4-step procedure. . To a flame dried 500 mL round bottom flask was charged *trans*-4-methylcyclohexane carboxylic acid (6.00 g, 42.2 mml), dissolved in 140 mL DCM, and was added *N*,*N*-dimethylformamide dimethyl acetal (11.6 mL, 84.4 mmol). The reaction was heated to reflux and allowed to stir overnight. The reaction was allowed to cool to room temperature and was diluted with ether, washed with saturated bicarbonate, washed with saturated ammonium chloride, washed with brine, dried with $MgSO_4$, and concentrated *in vacuo*. The crude reaction was taken on without further purification. To a flame dried 1 L round bottom flask was charged with lithium aluminum hydride (2.43 g, 64.0 mmol) and dissolved in 200 mL ether. The reaction was cooled to 0 °C and added *trans*-4-methylcyclohexane carboxylic acid methyl ester (6.6 g, 42.2 mmol) in 250 mL ether. The reaction was allowed to stir overnight. The reaction was quenched with 2.50 mL water, 5.00 mL 15% sodium hydroxide solution, and 7.50 mL water, then filtered through celite, dried with $MgSO_4$, and concentrated *in vacuo*. The crude product was taken on without further purification. To a flame dried 250 mL round bottom was charged tosyl chloride (5.18 g, 46.8 mmol), trimethylamine hydrochloride (0.300 g, 3.10 mmol), and dissolved in 31 mL DCM. The reaction was cooled to 0 °C and added trimethylamine (10.9 mL, 78.0 mmol) and (*trans*-4-methylcyclohexyl) methanol (4.00 g, 31.2 mmol) in 31 mL DCM. The reaction was allowed to stir for 3 hour and quenched with *N,N*-dimethyl-1,3-propanediamine (7.85 mL, 62.4 mmol) and allowed to stir for 15 minutes. The reaction was diluted with water, washed with 1 M HCl, and dried with MgSO4, and concentrated *in vacuo*. The crude product was taken on without further purification. To a flame dried 50 mL round bottom was charged with (*trans*-4-methylcyclohexyl) methanol tosylate (3.00 g, 10.6 mmol), dissolved in 21 mL DMF, and added lithium chloride (1.40 g, 31.9 mmol). The reaction was heated to 90 °C and allowed to stir overnight. The reaction was diluted with pentane and washed with water. The organic layer was filtered through a silica plug with pentane and concentrated *in vacuo*. The product (0.924 g, 59% yield) was obtained as a clear oil.

^{1}H NMR: (600MHz ,$CDCl_3$) 3.38 (d, J = 6.2 Hz, 2 H), 1.88 - 1.82 (m, 2 H), 1.76 - 1.69 (m, 2 H), 1.61 - 1.51 (m, 1 H), 1.36 - 1.25 (m, 1 H), 1.06 - 0.98 (m, 2 H), 0.97 - 0.90 (m, 2 H), 0.89 (d, J = 6.6 Hz, 3 H)
^{13}C NMR: (151MHz ,$CDCl_3$) 51.1, 40.0, 34.5, 32.5, 30.7, 22.5
HRMS: calculated $[C_8H_{15}Cl]^{+\bullet}$ = 146.08568, found = 146.08561

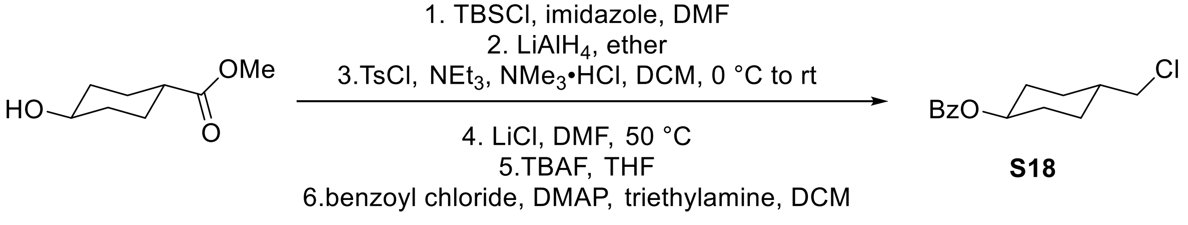

S18

***trans*-4-(chloromethyl)cyclohexyl benzoate (S18):** Prepared via a 6-step procedure. To a flame dried 100 mL round bottom was charged imidazole (3.40 g, 50.6 mmol), *trans*-4-methyl hydroxycyclohexane

carboxylate (4.00 g, 25.3 mmol), and dissolved in 17.0 mL DMF. To the reaction *tert*-butyldimethylsilyl chloride (4.20 g, 27.8 mmol) was added. The reaction was allowed to stir overnight. The reaction was quenched with water, diluted with ether, washed with brine, dried with $MgSO_4$, and concentrated *in vacuo*. The crude product was taken on without further purification. To a flame dried 500 mL round bottom was charged lithium aluminum hydride (1.13 g, 29.8 mmol) and dissolved in 120 mL ether. The reaction was cooled to 0 °C and added *trans*-4-methyl(*tert*-butyldimethylsilylether)cyclohexane carboxylate (6.50 g, 23.9 mmol) in 96.0 mL ether. The reaction was allowed to stir overnight. The reaction was quenched with 1.00 mL water, 2.50 mL 15% sodium hydroxide solution, and 2.50 mL water, then filtered through celite, dried with $MgSO_4$, and concentrated *in vacuo*. The crude product was taken on without further purification. To a flame dried 100 mL round bottom was charged tosyl chloride (3.40 g, 30.8 mmol), trimethylamine hydrochloride (0.200 g, 2.10 mmol), and dissolved in 20.0 mL DCM. The reaction was cooled to 0 °C and added trimethylamine (7.20 mL, 51.3 mmol) and *trans*-4-(*tert*-butyldimethylsilylether)cyclohexyl methanol (5.00 g, 20.5 mmol) in 20.0 mL DCM. The reaction was allowed to stir for 1 hour and quenched with *N,N*-dimethyl-1,3-propanediamine (5.20 mL, 41.0 mmol) and allowed to stir for 15 minutes. The reaction was diluted with water, washed with 1 M HCl, and dried with $MgSO_4$, and concentrated *in vacuo*. The crude product was taken on without further purification. To a flame dried 25 mL round bottom was charged with *trans*-4-(*tert*-butyldimethylsilylether)cyclohexyl methanol tosylate (8.00 g, 20.0 mmol), dissolved in 40.0 mL DMF, and added lithium chloride (2.55 g, 60.0 mmol). The reaction was heated to 90 °C and allowed to stir overnight. The reaction was diluted with ether, washed with water, dried with $MgSO_4$, and concentrated *in vacuo*. The crude product was taken on without further purification. To a flame dried 500 mL round bottom was charged *trans*-4-(chloromethyl) cyclohexyl *tert*-butyldimethylsilylether (4.00 g, 15.2 mmol) and dissolved in 100 mL THF. To the reaction tetrabutyl ammonium fluoride (1.99 g, 7.6 mmol) was added and the reaction was allowed to stir overnight. The reaction was quenched with 1 M HCl and extracted 3 times with ether. The organic layer was washed with water, brine, dried with $MgSO_4$, and concentrated *in vacuo*. The crude product was taken on without further purification. To a flame dried 250 mL round bottom flask was charged with DMAP (0.400 g, 3.40 mmol) and dissolved in 80.0 mL DCM. The reaction was cooled to 0 °C and added triethylamine (3.50 mL, 25.2 mmol), *trans*-4-(chloromethyl) cyclohexanol (2.50 g, 16.8 mmol), and benzoyl chloride (2.40 mL, 20.2 mmol). The reaction was allowed to stir overnight. The reaction was quenched with water, extracted with DCM, dried with $MgSO_4$, and concentrated *in vacuo*. The crude product was purified via column chromatography (10% ethyl acetate in hexanes) to give the product (2.08 g, 49% yield) was obtained as an off white oil.

^{1}H NMR: (600MHz ,$CDCl_3$) 8.11 - 7.99 (m, 11 H), 7.63 - 7.52 (m, 1 H), 7.50 - 7.39 (m, 2 H), 4.94 (tt, J = 4.4, 11.0 Hz, 1 H), 3.44 (d, J = 6.6 Hz, 2 H), 2.21 - 2.14 (m, 2 H), 2.08 - 1.95 (m, 2 H), 1.79 - 1.64 (m, 1 H), 1.57 - 1.49 (m, 2 H), 1.30 - 1.20 (m, 2 H)
^{13}C NMR: (151MHz ,$CDCl_3$) 166.0, 132.8, 130.6, 129.5, 128.3, 73.4, 49.9, 39.2, 31.0, 28.5
HRMS: calculated for $[C_{14}H_{17}ClO_2+H]^+$ = 253.09898, found = 253.09897

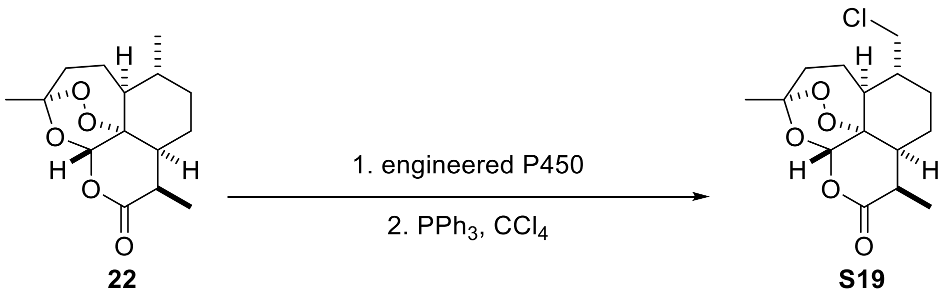

6a-chloro-artemisinin (S19) was prepared by the Fasan group according to a literature procedure. [6]

^{1}H NMR: (600MHz ,$CDCl_3$) 5.84 (s, 1 H), 3.73 - 3.62 (m, 2 H), 3.41 (dq, J = 5.5, 7.2 Hz, 1 H), 2.48 (ddd, J = 4.2, 13.2, 14.9 Hz, 1 H), 2.09 (ddd, J = 2.9, 5.0, 14.9 Hz, 1 H), 2.02 - 1.94 (m, 2 H), 1.90 - 1.77 (m, 3 H), 1.76 - 1.67 (m, 1 H), 1.58 - 1.42 (m, 5 H), 1.28 - 1.19 (m, 4 H), 1.13 (dq, J = 3.5, 13.6 Hz, 1 H)
^{13}C NMR: (151MHz , CDCl3) 171.7, 105.4, 93.5, 79.3, 47.6, 44.5, 44.3, 43.5, 35.6, 32.8, 28.2, 25.1, 24.0, 22.7, 12.5
HRMS: calculated $[C_{15}H_{21}ClO_5+H]^+$ = 317.11503, found = 317.11380

C-H Functionalizations

General Procedure A:

A flame dried, 1 dram vial, charged with a stir bar was brought into the glove where BPO (10 mol %), cesium carbonate (0.5 equiv), $PhCF_3$ (0.5 M with respect to substrate), substrate (1 equiv), and chloroamide (1 or 1.5 equiv) were added. The reaction was capped with a PTFE lined screw cap, sealed with Teflon tape, taken out of the glovebox, heated to 65 °C in a pie block, and stirred for 7 hours. The reaction was brought into the glovebox where BPO (10 mol %) was added. The reaction was capped and resealed with Teflon tape, taken out of the glovebox, heated to 65 °C in a pie block, and stirred for 17 hours. Upon completion, the reaction mixture was diluted with 3 mL of DCM and analyzed by GC using dodecane as an internal standard. The diluted reaction mixture was then filtered over celite and concentrated down *in vacuo* and analyzed by NMR using 15.5 µL 2,5-dimethylfuran or 5.1 µL hexamethyldisiloxane as an internal standard.

General Procedure B:

A flame dried, 1 dram vial, charged with a stir bar was brought into the glove where substrate (3 or 5 equiv), BPO (10 mol %), cesium carbonate (0.5 equiv), $PhCF_3$ (0.5 M with respect to chloroamide), and chloroamide (1.0 equiv) were added. The reaction was capped with a PTFE lined screw cap, sealed with Teflon tape, taken out of the glovebox, heated to 65 °C in a pie block, and stirred for 7 hours. The reaction was brought into the glovebox where BPO (10 mol %) was added. The reaction was capped and resealed with Teflon tape, taken out of the glovebox, heated to 65 °C in a pie block, and stirred for 17 hours. Upon completion, the reaction mixture was diluted with 3 mL of DCM and analyzed by GC using dodecane as an internal standard. The diluted reaction mixture was then filtered over celite and concentrated down *in vacuo* and analyzed by NMR using 15.5 µL 2,5-dimethylfuran or 5.1 µL hexamethyldisiloxane as an internal standard.

General Procedure C:

A flame dried, 1 dram vial was brought into the glove charged with xanthylamide (1.5 equiv), dissolved in $PhCF_3$ (1.0 M with respect to substrate) and added substrate (1 equiv). The reaction was capped with a PTFE lined screw cap, sealed with Teflon tape, taken out of the glovebox, placed over blue LED lights and allowed to react for 24 hours. Upon completion, the reaction mixture was diluted with 3 mL of DCM and analyzed by GC using dodecane as an internal standard. The diluted reaction mixture was then filtered over celite and concentrated down *in vacuo* and analyzed by NMR using 15.5 µL 2,5-dimethylfuran or 5.1 µL hexamethyldisiloxane as an internal standard.

General Procedure D:

A flame dried, 1 dram vial, charged with a stir bar was brought into the glove where substrate (1 equiv), BPO (20 mol %), cesium carbonate (0.5 equiv), $PhCF_3$ (0.5 M with respect to substrate), and chloroamide (2.0 equiv) were added. The reaction was capped with a PTFE lined screw cap, sealed with Teflon tape, taken out of the glovebox, heated to 65 °C in a pie block, and stirred for 7 hours. The reaction was brought into the glovebox where BPO (20 mol %) was added. The reaction was capped and resealed with Teflon tape, taken out of the glovebox, heated to 65 °C in a pie block, and stirred for 16 hours. The reaction was brought into the glovebox where BPO (20 mol %) was added. The reaction was capped and resealed with Teflon tape, taken out of the glovebox, heated to 65 °C in a pie block, and stirred for 8 hours. The reaction was brought into the glovebox where BPO (20 mol %) was added. The reaction was capped and resealed with Teflon tape, taken out of the glovebox, heated to 65 °C in a pie block, and stirred for 17 hours. Upon completion, the reaction mixture was diluted with DCM and filtered over celite and concentrated down *in vacuo* and analyzed by NMR using 15.5 µL 2,5-dimethylfuran or 5.1 µL hexamethyldisiloxane as an internal standard.

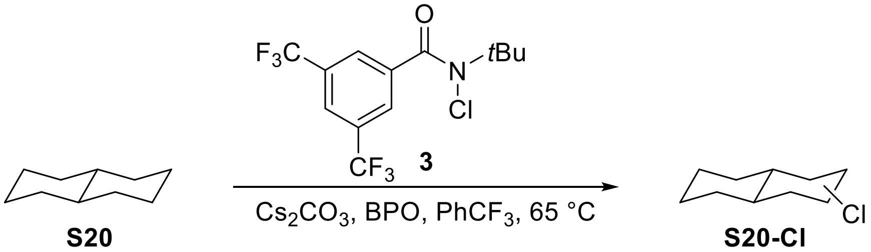

Reaction with *trans*-decalin: Prepared according to General Procedure B with 5 equiv of substrate on 0.144 mmol scale using chloroamide **6** and *trans*-decalin.

Figure S1. ^{1}H NMR of crude reaction of *trans*-decalin with 3.

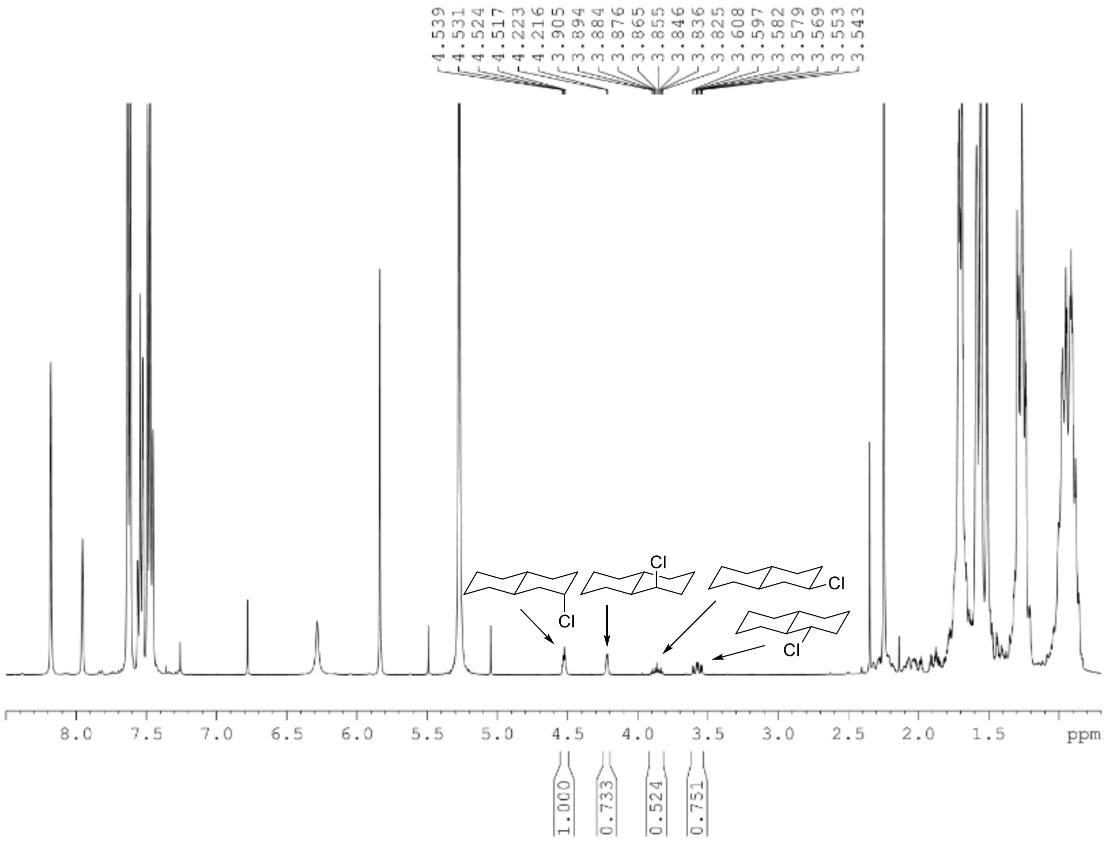

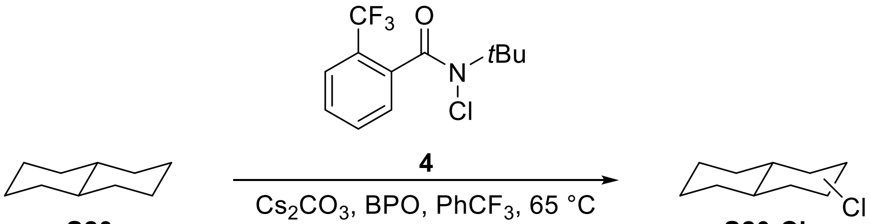

S20 **S20-Cl**

Reaction with *trans*-decalin: Prepared according to General Procedure B with 5 equiv of substrate on 0.144 mmol scale using chloroamide **6** and *trans*-decalin.

Figure S2. ^{1}H NMR of crude reaction of *trans*-decalin with 4.

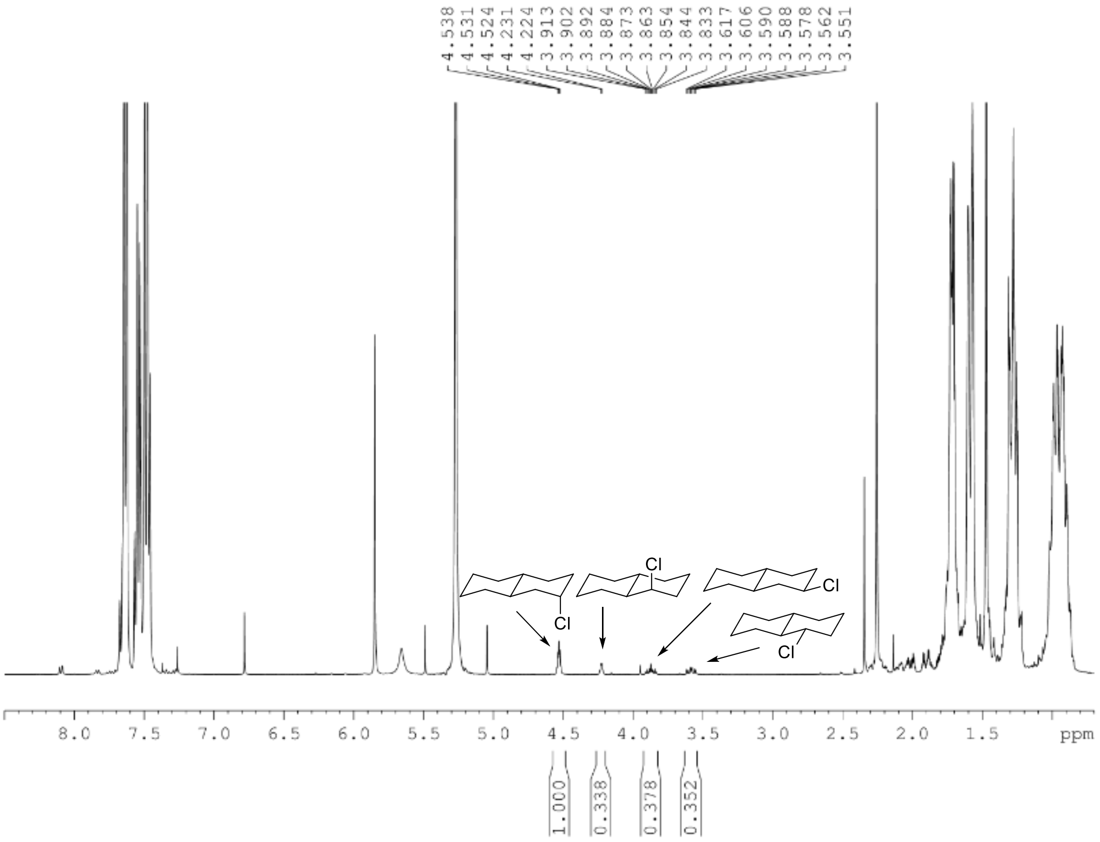

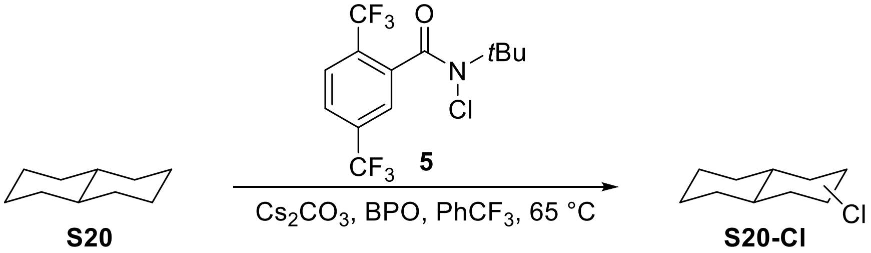

Reaction with *trans*-decalin: Prepared according to General Procedure B with 5 equiv of substrate on 0.144 mmol scale using chloroamide **6** and *trans*-decalin.

Figure S3. ^{1}H NMR of crude reaction of *trans*-decalin with 5.

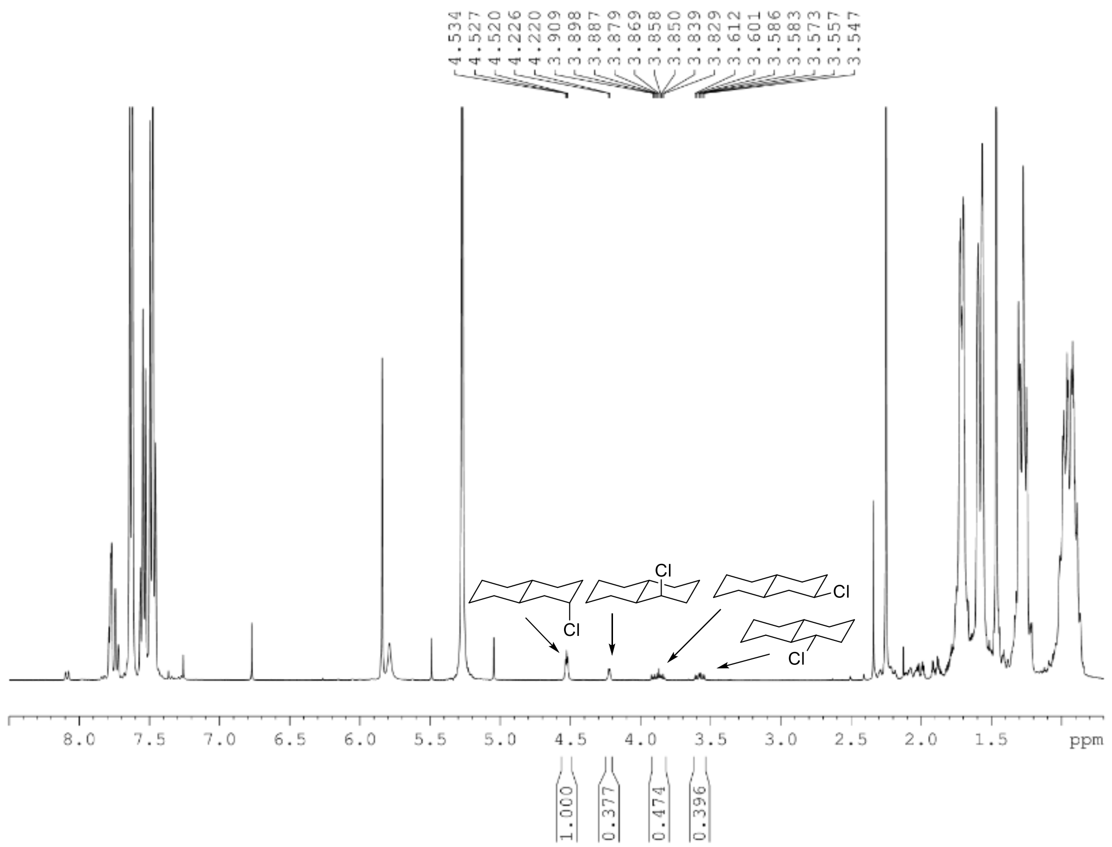

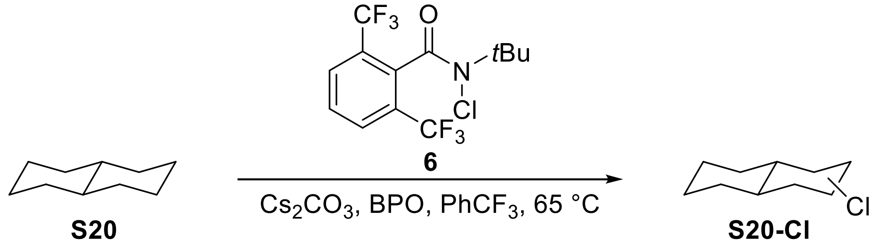

Reaction with *trans*-decalin: Prepared according to General Procedure B with 5 equiv of substrate on 0.144 mmol scale using chloroamide **6** and *trans*-decalin.

Figure S4. ^{1}H NMR of crude reaction of *trans*-decalin with 6.

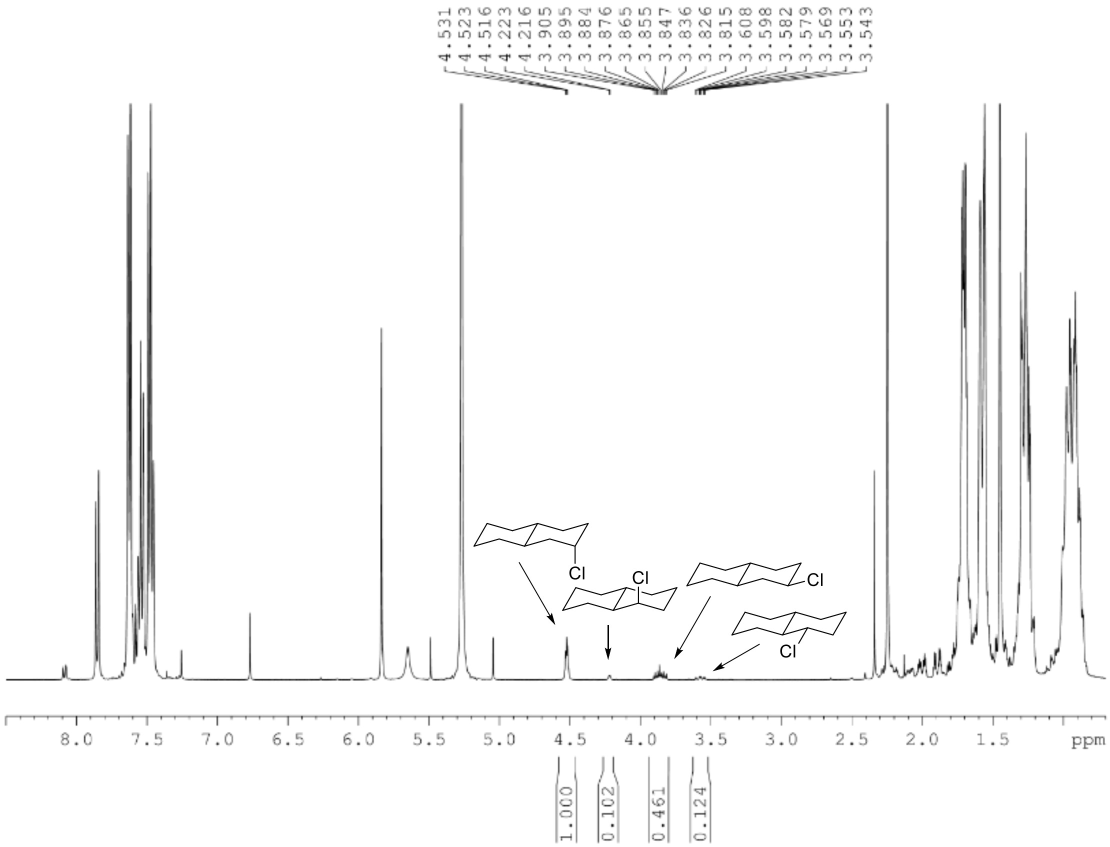

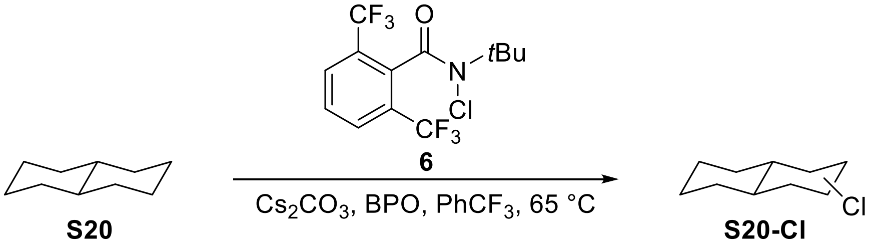

Reaction with *trans*-decalin: Prepared according to General Procedure A with 1 equiv of chloroamide on 0.144 mmol scale using chloroamide **6** and *trans*-decalin giving 76% NMR yield.

Figure S5. ^{1}H NMR of crude reaction of *trans*-decalin with 6.

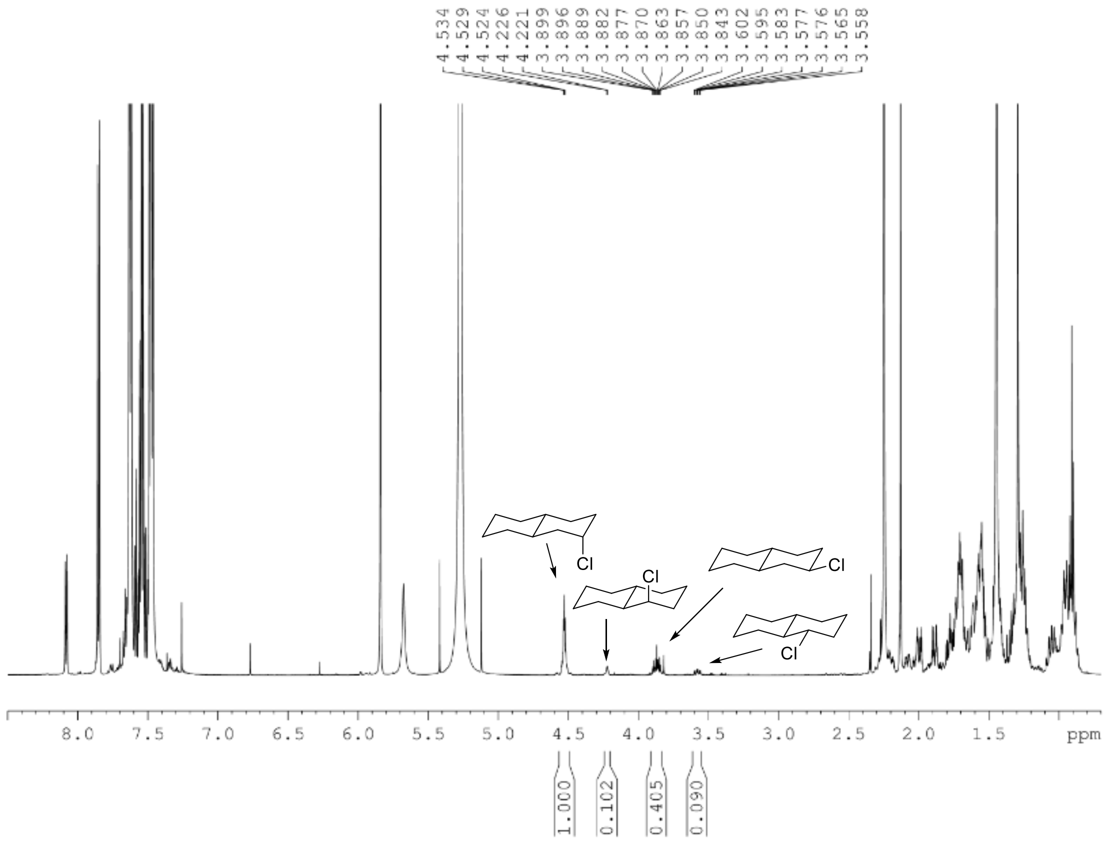

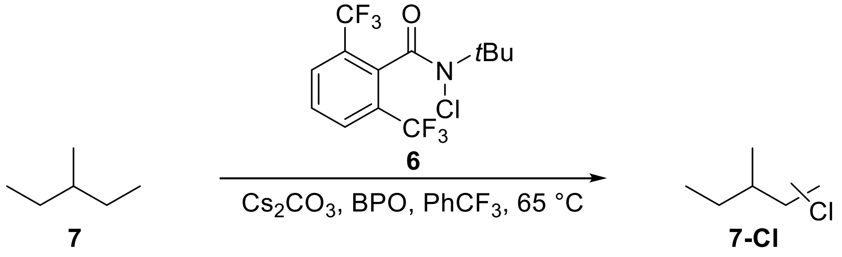

Reaction with 3-methylpentane: Prepared according to General Procedure A with 1.5 equiv of chloroamide on 0.144 mmol scale using chloroamide **6** and 3-methylpentane giving 69% GC yield. GC data obtained using Method 1.

Table S1. GC data for functionalization of 3-methylpentane with 6.

Product	Retention Time	Peak Area
3°	3.730	2.423
ω-1	4.160	11.153
ω-1	4.247	9.931
1°	4.920	7.277
ω	5.023	69.217

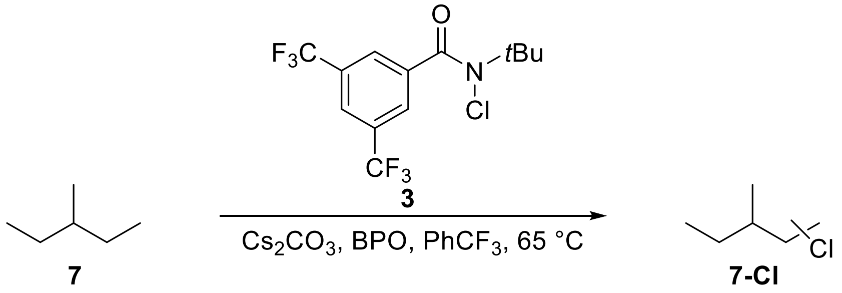

Reaction with 3-methylpentane: Prepared according to General Procedure A with 1.5 equiv of chloroamide on 0.144 mmol scale using chloroamide **3** and 3-methylpentane giving 96% GC yield. GC data obtained using Method 1.

Table S2. GC data for functionalization of 3-methylpentane with 3.

Product	Retention Time	Peak Area
3°	3.727	6.05
ω-1	4.159	32.653
ω-1	4.246	30.938
1°	4.927	3.559
ω	5.029	26.801

Figure S6. Chromatograph of 3-methylpentane functionalizations

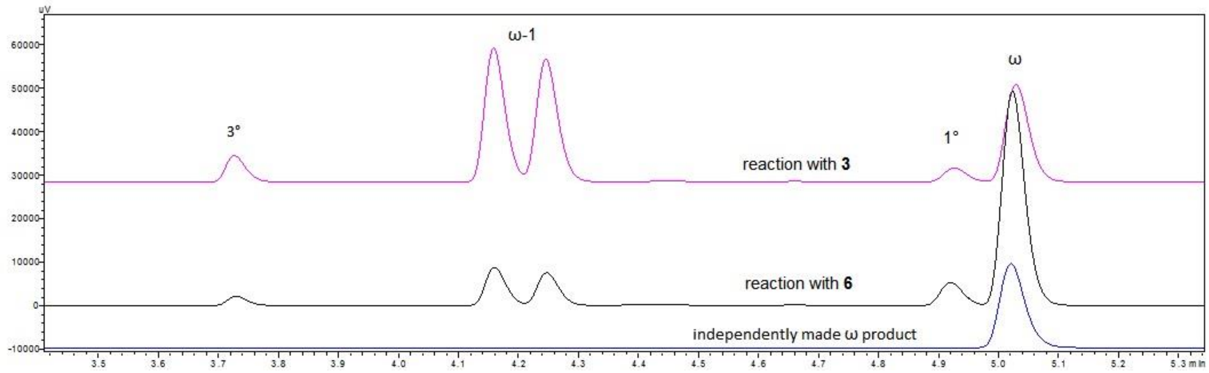

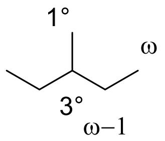

GC assignments supported by ^{1}H NMR spectrum analysis.

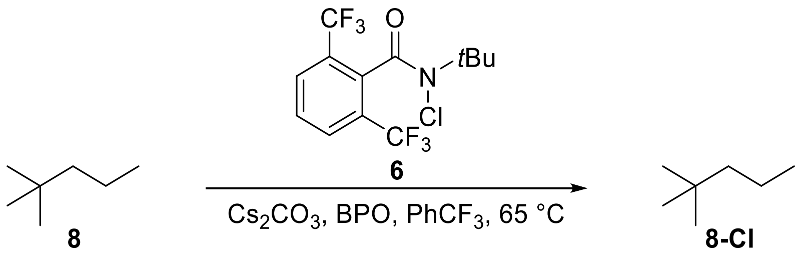

Reaction with 2,2-dimethylpentane: Prepared according to General Procedure A with 1.5 equiv of chloroamide on 0.144 mmol scale using chloroamide **6** and 2,2-dimethylpentane giving 56% GC yield. GC data obtained using Method 1.

Table S3. GC data for functionalization of 2,2-dimethylpentane with 6.

Product	Retention Time	Peak Area
ω-1	4.737	30.953
1°	6.535	3.438
ω	6.922	65.608

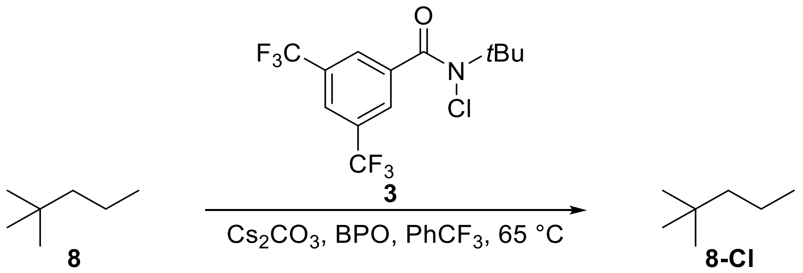

Reaction with 2,2-dimethylpentane: Prepared according to General Procedure A with 1.5 equiv of chloroamide on 0.144 mmol scale using chloroamide **3** and 2,2-dimethylpentane giving 45% GC yield. GC data obtained using Method 1.

Table S4. GC data for functionalization of 2,2-dimethylpentane with 3.

Product	Retention Time	Peak Area
ω-1	4.777	51.718
1°	6.574	18.004
ω	6.965	30.278

Figure S7. Chromatograph of 2,2-dimethylpentane functionalizations.

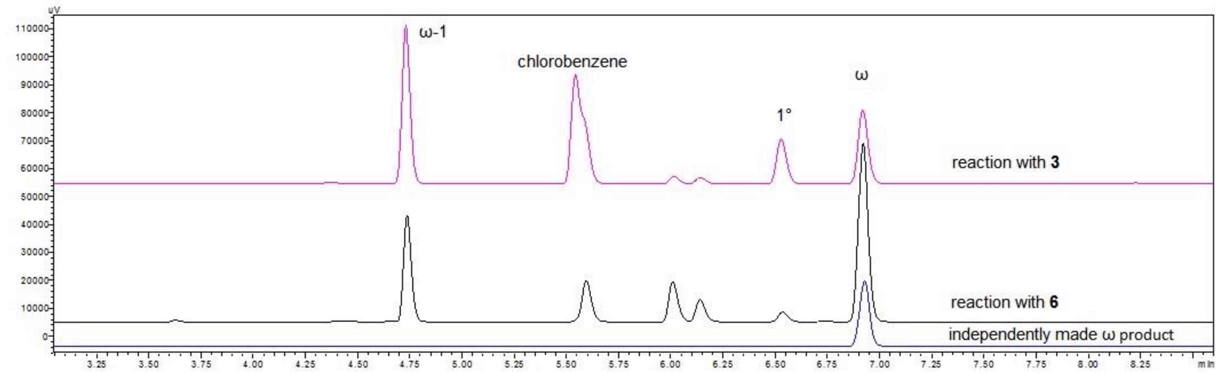

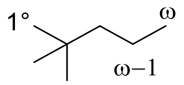

GC assignments supported by ^{1}H NMR spectrum analysis.

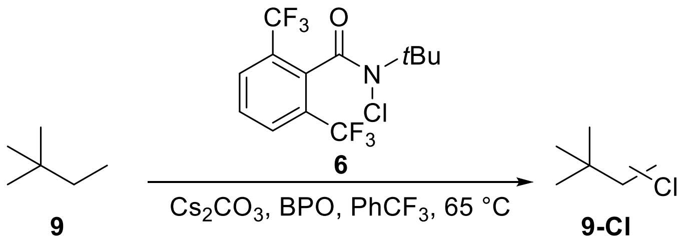

Reaction with 2,2-dimethylbutane: Prepared according to General Procedure B with 3 equiv of substrate on 0.144 mmol scale using chloroamide **6** and 2,2-dimethylbutane giving 40% NMR yield. GC data obtained using Method 1.

Figure S8. ¹H NMR of crude reaction of 2,2-dimethylbutane with 6.

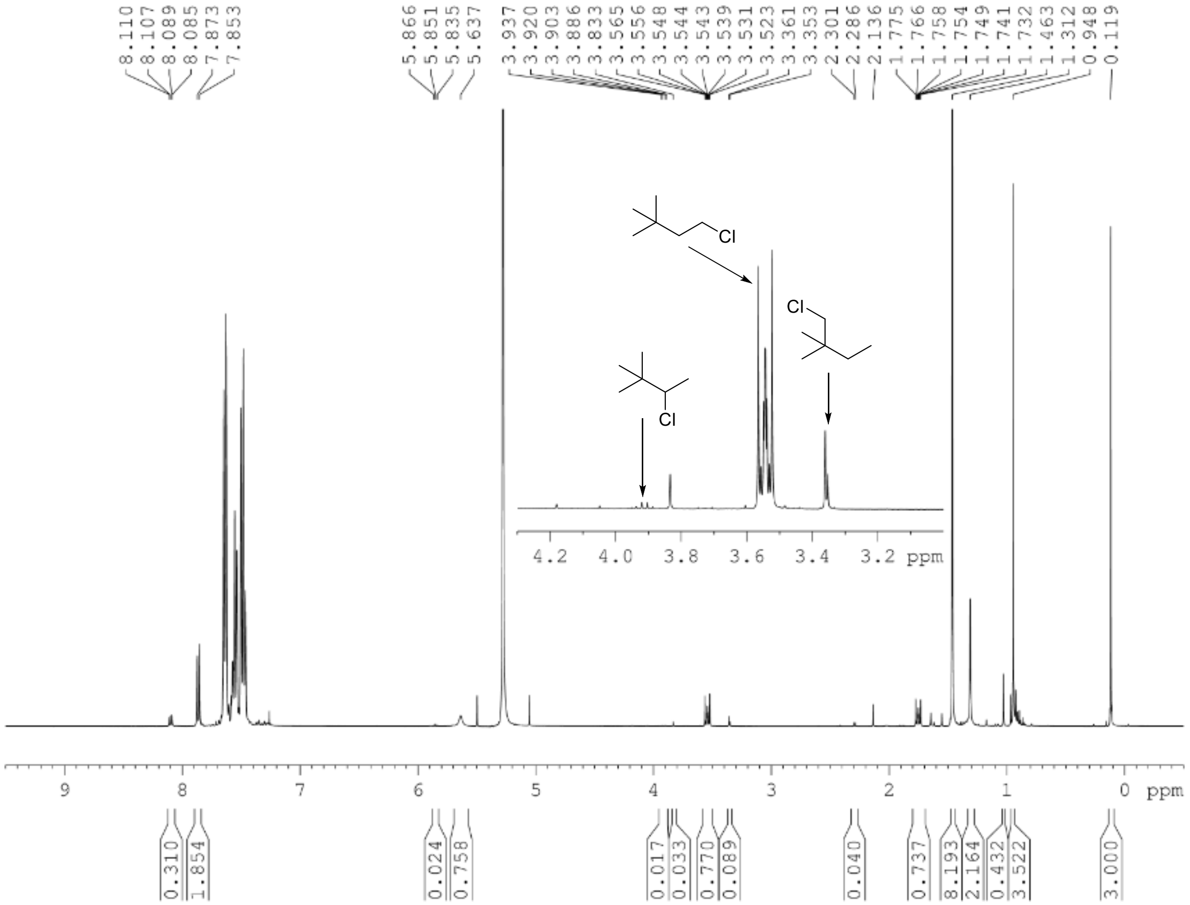

Table S5. GC data for functionalization of 2,2-dimethylbutane with 6.

Product	Retention Time	Peak Area
2°	3.137	3.522
1°	3.606	89.33
t-Bu 1°	3.716	7.148

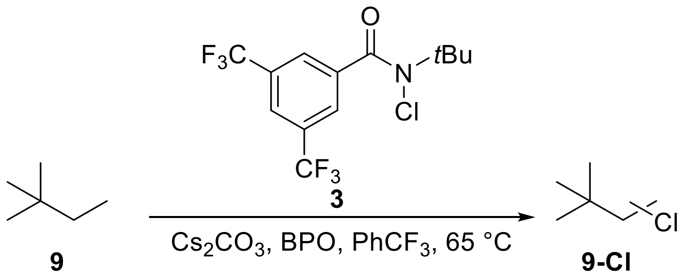

Reaction with 2,2-dimethylbutane: Prepared according to General Procedure B with 3 equiv of substrate on 0.144 mmol scale using chloroamide **3** and 2,2-dimethylbutane giving 39% NMR yield. GC data obtained using Method 1.

Figure S9. ^{1}H NMR of crude reaction of 2,2-dimethylbutane with 3.

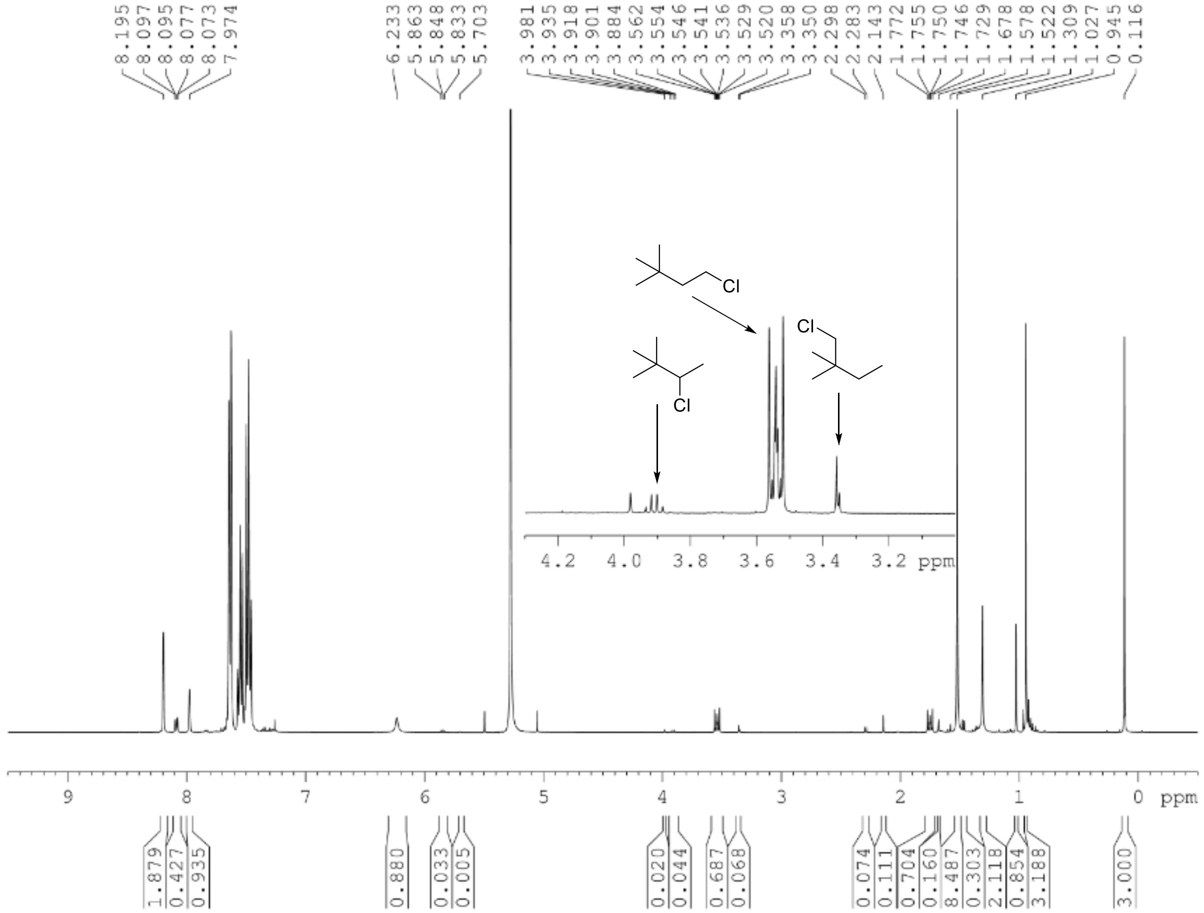

Table S6. GC data for functionalization of 2,2-dimethylbutane with 3.

Product	Retention Time	Peak Area
2°	3.133	13.083
1°	3.604	80.895
t-Bu 1°	3.715	6.022

Figure S10. Chromatograph of 2,2-dimethylbutane functionalizations.

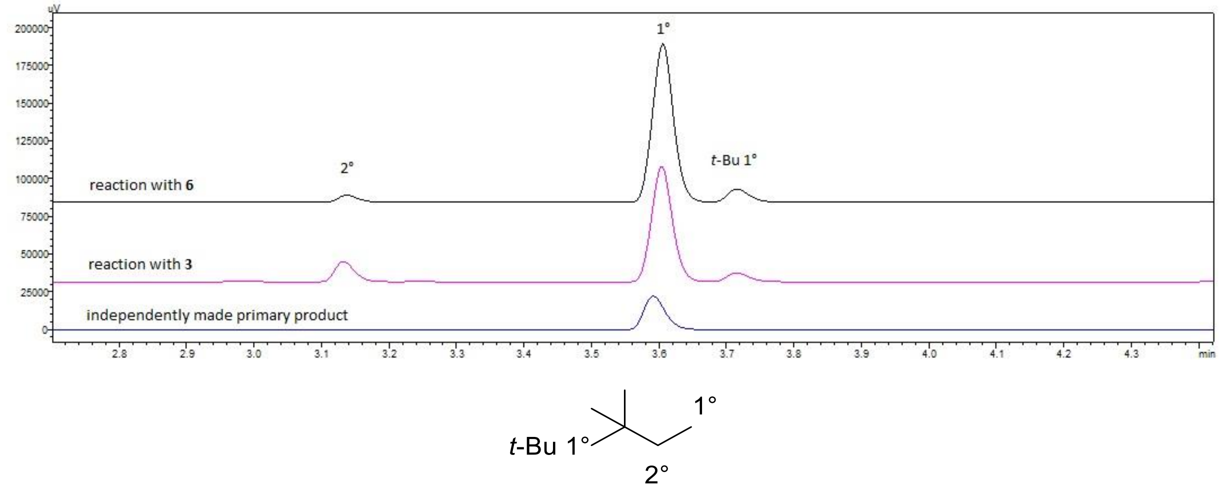

GC assignments supported by ^{1}H NMR spectrum analysis.

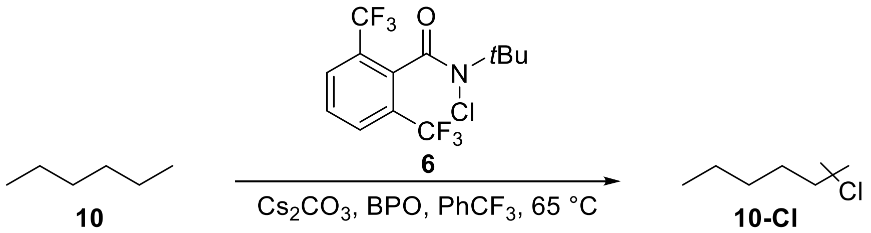

Reaction with *n*-hexane: Prepared according to General Procedure B with 5 equiv of substrate on 0.144 mmol scale using chloroamide **6** and *n*-hexane giving 78% NMR yield. GC data obtained using Method 1.

Table S7. GC data for functionalization of *n*-hexane with 6.

Product	Retention Time	Peak Area
ω-1 & ω-2	4.439	68.354
ω	6.096	31.646

Figure S11. ^{1}H NMR of crude reaction of *n*-hexane with 6.

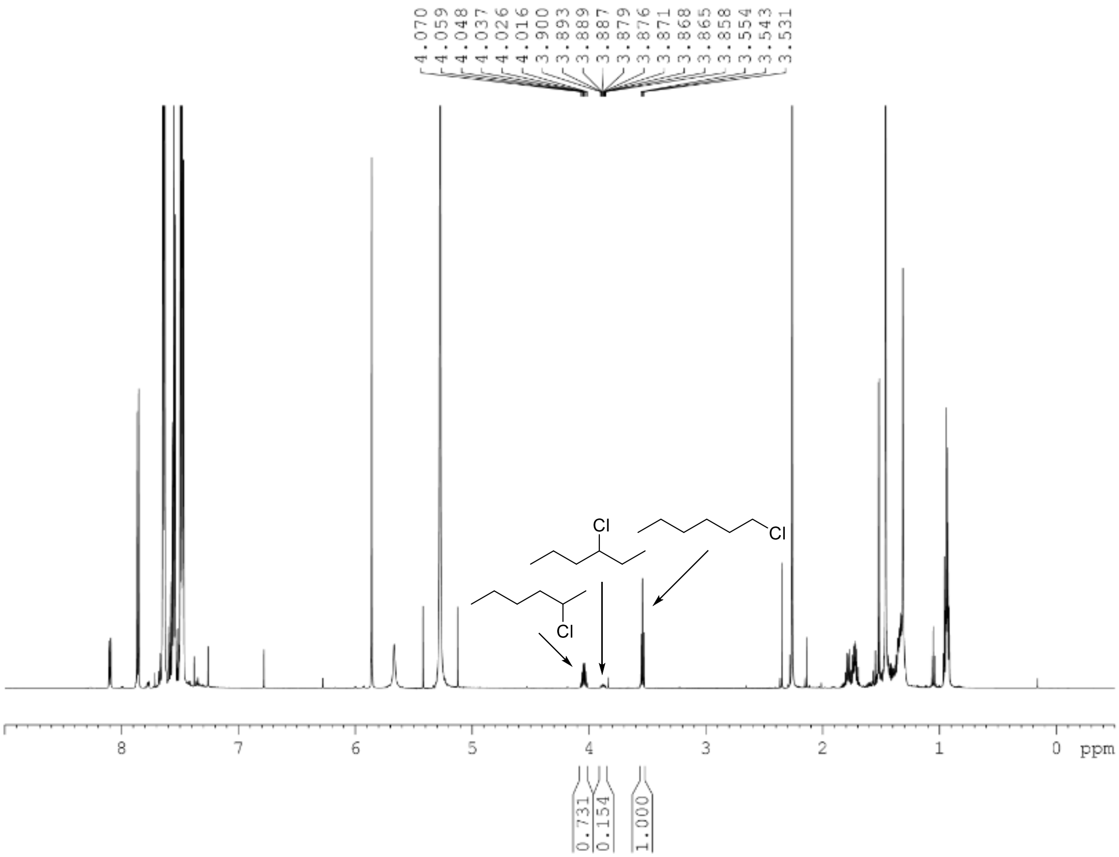

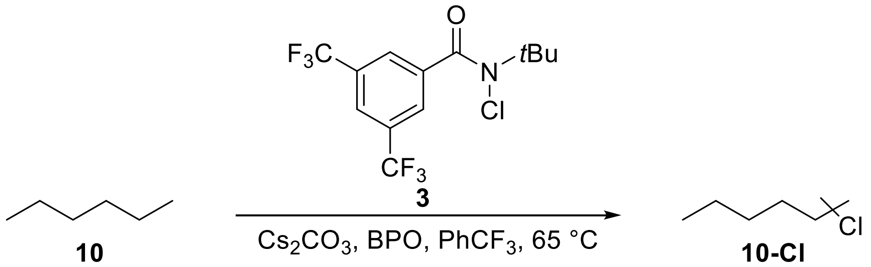

Reaction with *n*-hexane: Prepared according to General Procedure B with 5 equiv of substrate on 0.144 mmol scale using chloroamide **3** and *n*-hexane giving 76% NMR yield. GC data obtained using Method 1.

Table S8. GC data for functionalization of *n*-hexane with 3.

Product	Retention Time	Peak Area
ω-1 & ω-2	4.48	89.313
ω	6.139	10.687

Figure S12. ^{1}H NMR of crude reaction of *n*-hexane with 3.

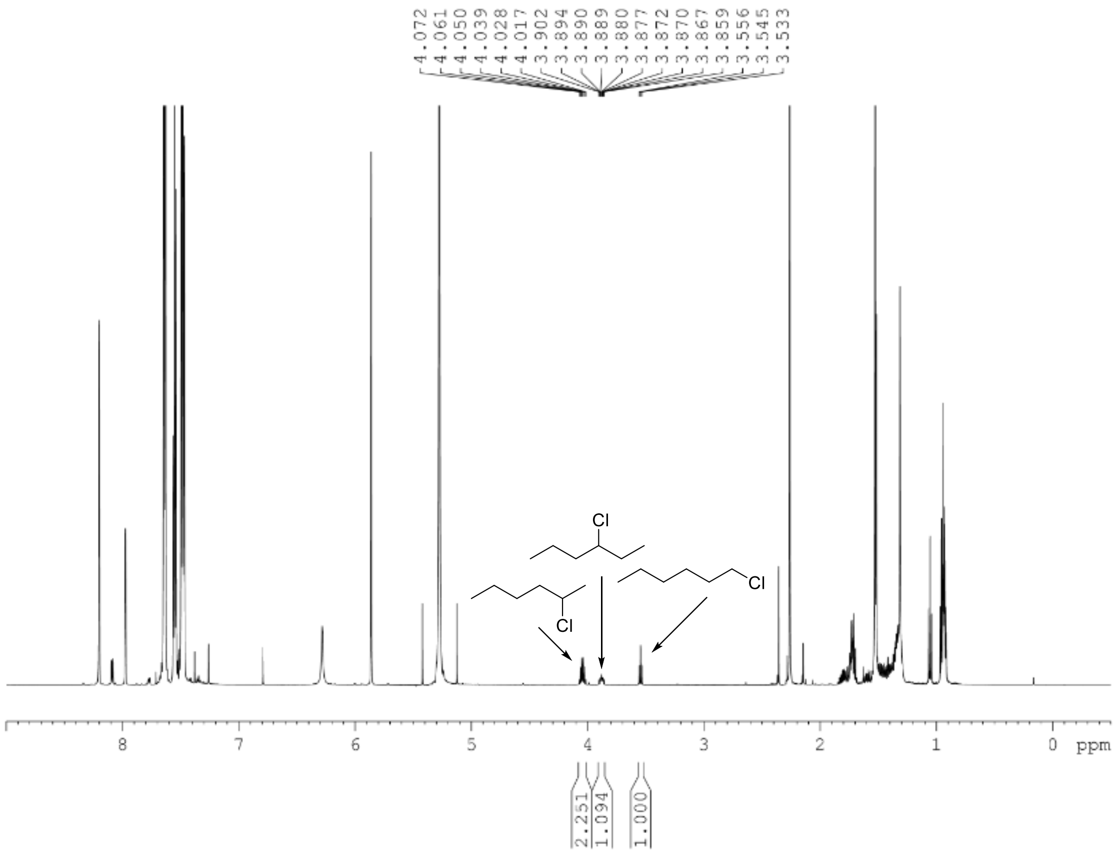

Figure S13. Chromatograph of *n*-hexane functionalizations

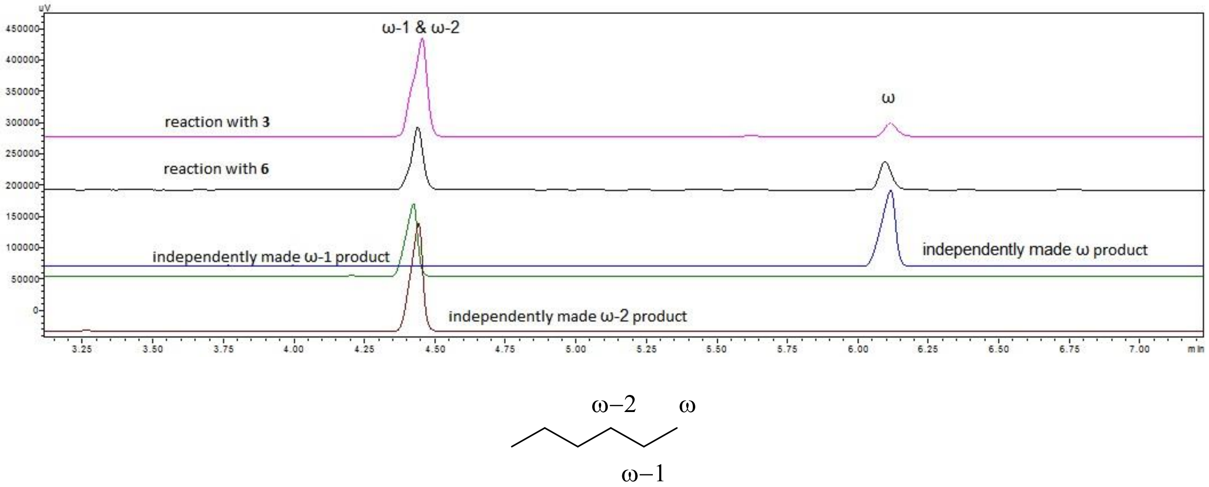

GC assignments supported by ^{1}H NMR spectrum analysis.

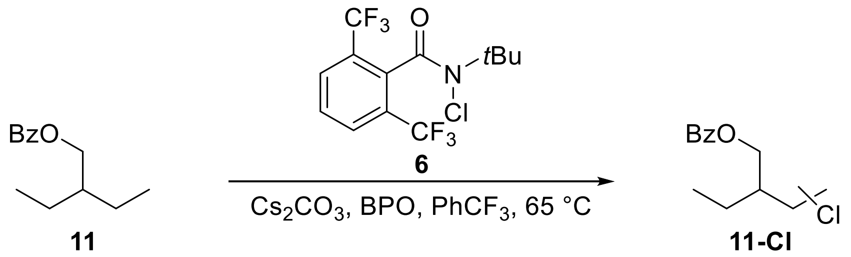

Reaction with 2-ethylbutyl benzoate: Prepared according to General Procedure B with 3 equiv of substrate on 0.144 mmol scale using chloroamide **6** and 2-ethylbutyl benzoate giving 42% GC yield. GC data obtained using Method 4.

Table S9. GC data for functionalization of 2-ethylbutyl benzoate with 6.

Product	Retention Time	Peak Area
3°	9.024	2.243
ω-1	9.635	9.626
ω	10.021	88.132

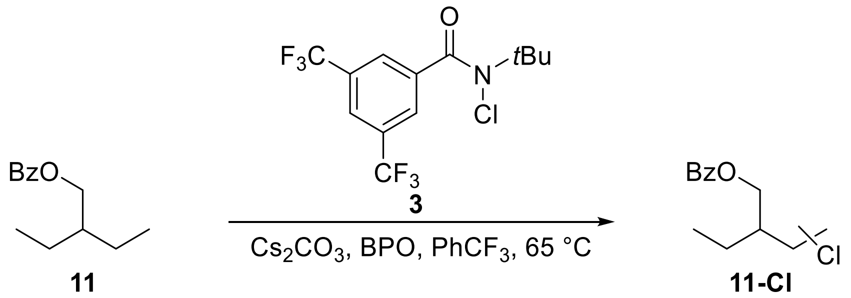

Reaction with 2-ethylbutyl benzoate: Prepared according to General Procedure B with 3 equiv of substrate on 0.144 mmol scale using chloroamide **3** and 2-ethylbutyl benzoate giving 41% GC yield. GC data obtained using Method 4.

Table S10. GC data for functionalization of 2-ethylbutyl benzoate with 3.

Product	Retention Time	Peak Area
3°	8.936	11.983
ω-1	9.547	37.648
ω	9.905	50.368

Figure S14. Chromatograph of 2-ethylbutyl benzoate functionalizations.

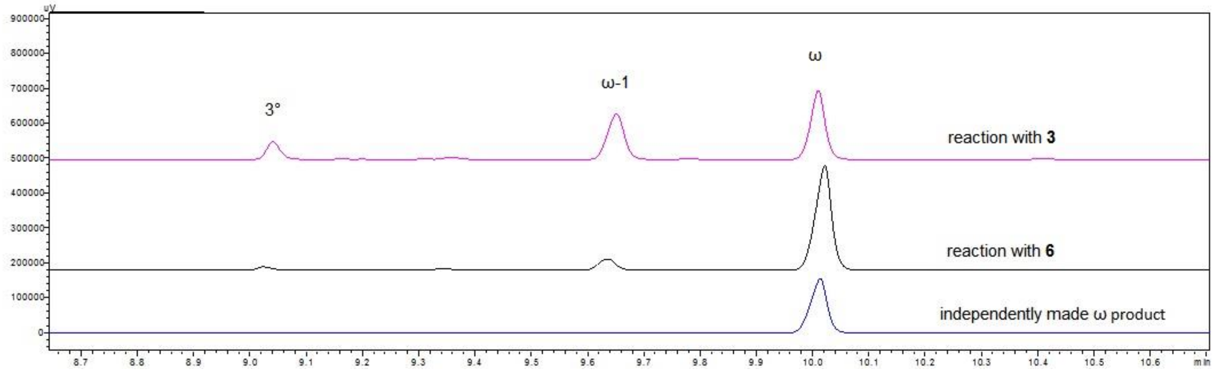

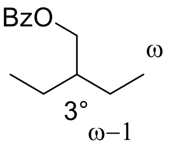

GC assignments supported by ^{1}H NMR spectrum analysis.

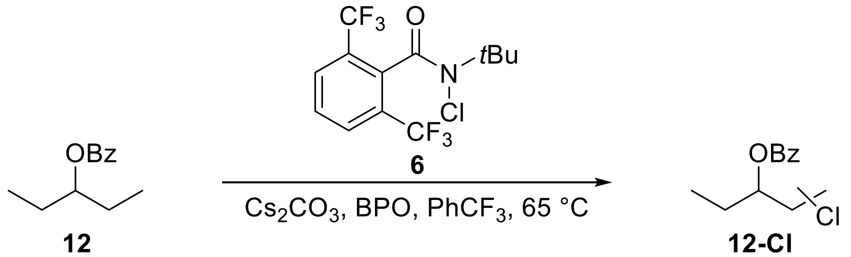

Reaction with 3-pentyl benzoate: Prepared according to General Procedure A with 1.5 equiv of chloroamide on 0.144 mmol scale using chloroamide **6** and 3-pentyl benzoate giving 47% GC yield. GC data obtained using Method 4.

Table S11. GC data for functionalization of 3-pentyl benzoate with 6.

Product	Retention Time	Peak Area
ω-1	8.179	4.92
ω-1	8.222	5.797
ω	8.772	89.282

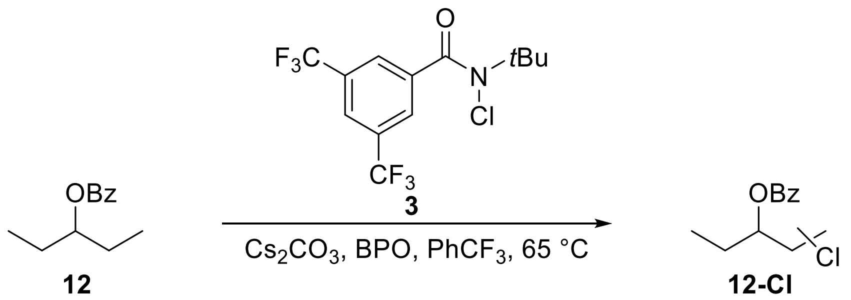

Reaction with 3-pentyl benzoate: Prepared according to General Procedure A with 1.5 equiv of chloroamide on 0.144 mmol scale using chloroamide **3** and 3-pentyl benzoate giving 52% GC yield. GC data obtained using Method 4.

Table S12. GC data for functionalization of 3-pentyl benzoate with 3.

Product	Retention Time	Peak Area
ω-1	8.181	19.220
ω-1	8.224	23.126
ω	8.767	57.654

Figure S15. Chromatograph of 3-pentyl benzoate functionalizations.

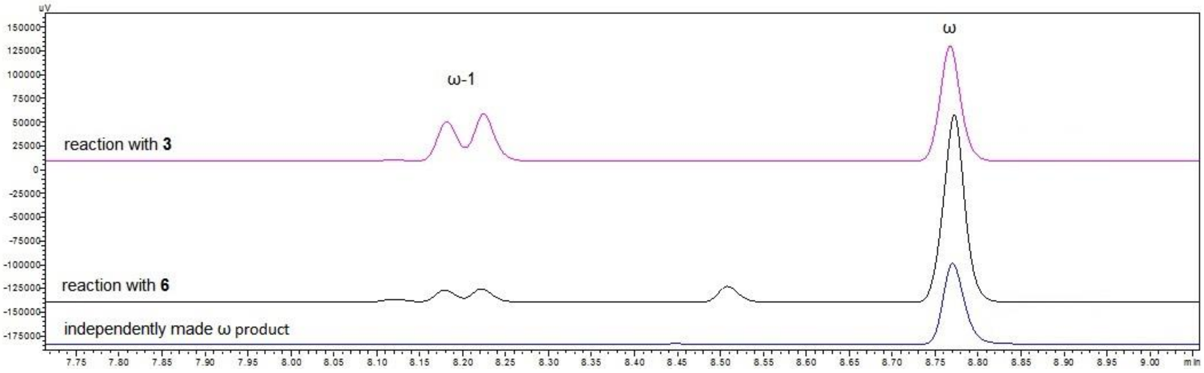

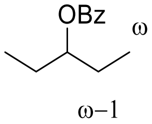

GC assignments supported by ^{1}H NMR spectrum analysis.

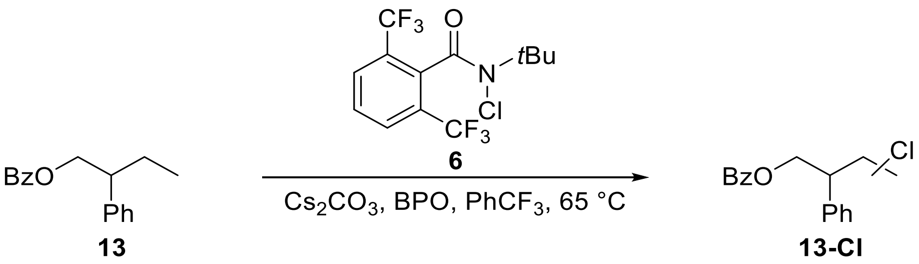

Reaction with 2-phenylbutyl benzoate: Prepared according to General Procedure B with 3 equiv of substrate on 0.144 mmol scale using chloroamide **6** and 2-phenylbutyl benzoate giving 58% GC yield. GC data obtained using Method 4.

Table S13. GC data for functionalization of 2-phenylbutyl benzoate with 6.

Product	Retention Time	Peak Area
minor product	12.595	1.915
minor product	12.937	2.519
minor product	13.878	3.677
minor product	14.227	2.924
ω	15.045	88.967

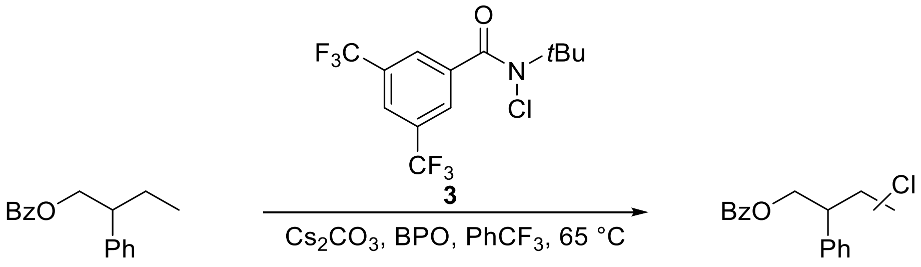

Reaction with 2-phenylbutyl benzoate: Prepared according to General Procedure B with 3 equiv of substrate on 0.144 mmol scale using chloroamide **3** and 2-phenylbutyl benzoate giving 56% GC yield. GC data obtained using Method 4.

Table S14. GC data for functionalization of 2-phenylbutyl benzoate with 3.

Product	Retention Time	Peak Area
minor product	12.596	13.168
minor product	12.942	16.954
minor product	13.881	15.703
minor product	14.23	13.898
ω	15.015	40.277

Figure S16. Chromatograph of 2-phenylbutyl benzoate functionalizations.

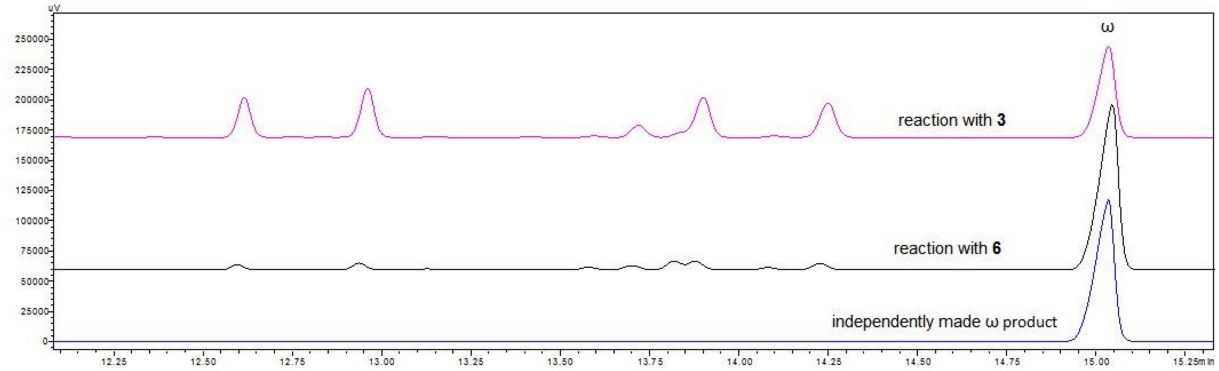

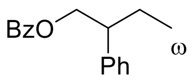

GC assignments supported by ^{1}H NMR spectrum analysis.

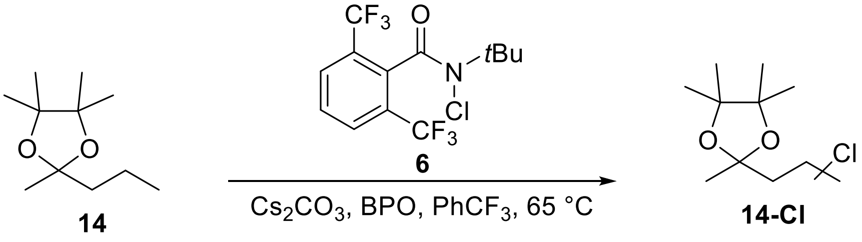

Reaction with 2,4,4,5,5-pentamethyl-2-propyl-1,3-dioxolane: Prepared according to General Procedure A with 1.5 equiv of chloroamide on 0.144 mmol scale using chloroamide **6** and 2,4,4,5,5-pentamethyl-2-propyl-1,3-dioxolane giving 46% GC yield. GC data obtained using Method 3.

Table S15. GC data for functionalization of 2,4,4,5,5-pentamethyl-2-propyl-1,3-dioxolane with 6.

Product	Retention Time	Peak Area
ω-1	15.155	50.335
ω	17.713	49.665

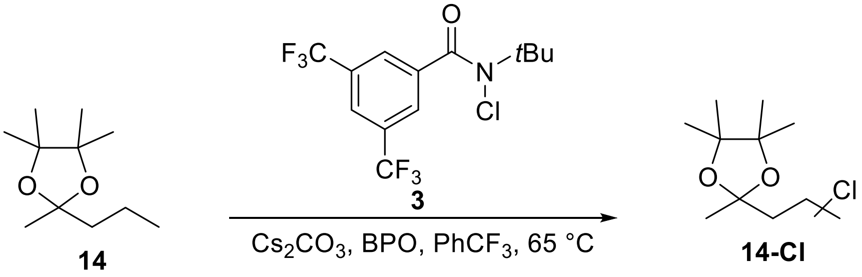

Reaction with 2,4,4,5,5-pentamethyl-2-propyl-1,3-dioxolane: Prepared according to General Procedure A with 1.5 equiv of chloroamide on 0.144 mmol scale using chloroamide **3** and 2,4,4,5,5-pentamethyl-2-propyl-1,3-dioxolane giving 66% GC yield. GC data obtained using Method 3.

Table S16. GC data for functionalization of 2,4,4,5,5-pentamethyl-2-propyl-1,3-dioxolane with 3.

Product	Retention Time	Peak Area
ω-1	15.183	75.404
ω	17.700	24.596

Figure S17. Chromatograph of 2,4,4,5,5-pentamethyl-2-propyl-1,3-dioxolane functionalizations.

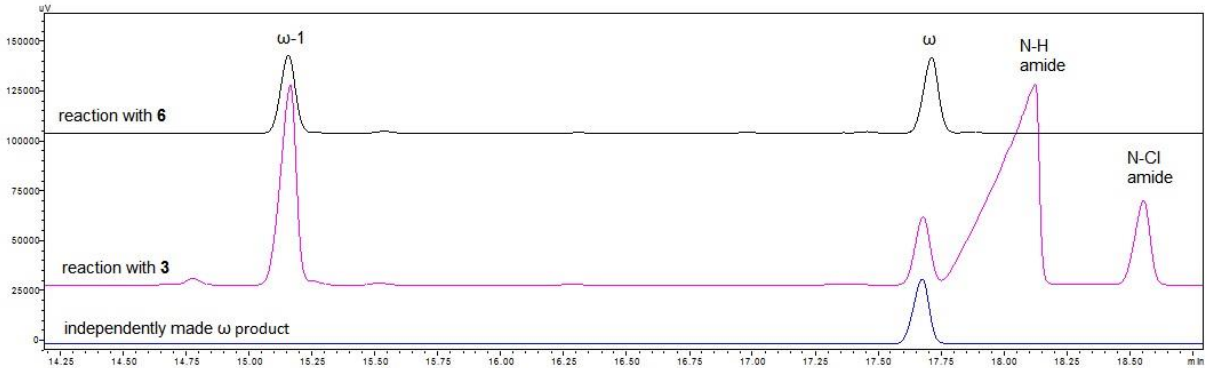

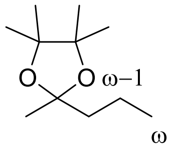

GC assignments supported by ^{1}H NMR spectrum analysis.

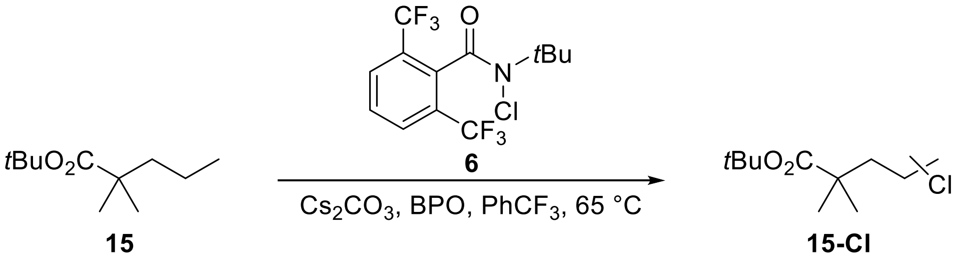

Reaction with *tert*-butyl 2,2-dimethylpentanoate: Prepared according to General Procedure A with 1.5 equiv of chloroamide on 0.144 mmol scale using chloroamide **6** and *tert*-butyl 2,2-dimethylpentanoate giving 51% GC yield. GC data obtained using Method 3.

Table S17. GC data for functionalization of *tert*-butyl 2,2-dimethylpentanoate with 6.

Product	Retention Time	Peak Area
ω-1	12.963	23.062
minor product	13.947	2.299
ω	15.406	74.638

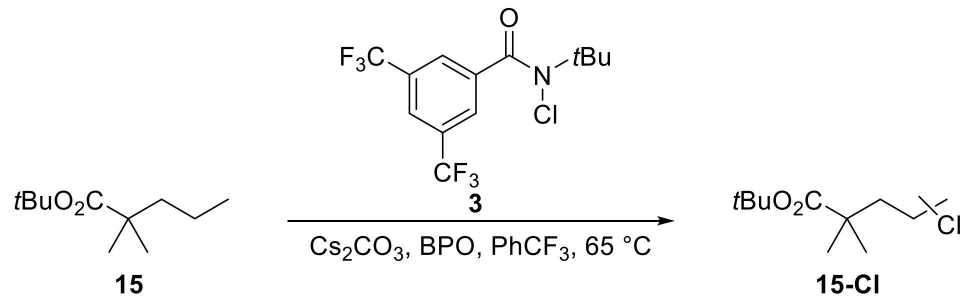

Reaction with *tert*-butyl 2,2-dimethylpentanoate: Prepared according to General Procedure A with 1.5 equiv of chloroamide on 0.144 mmol scale using chloroamide **3** and *tert*-butyl 2,2-dimethylpentanoate giving 55% GC yield. GC data obtained using Method 3.

Table S18. GC data for functionalization of *tert*-butyl 2,2-dimethylpentanoate with 3.

Product	Retention Time	Peak Area
ω-1	12.984	54.378
minor product	13.951	1.56
ω	15.397	44.062

Figure S18. Chromatograph of *tert*-butyl 2,2-dimethylpentanoate functionalizations.

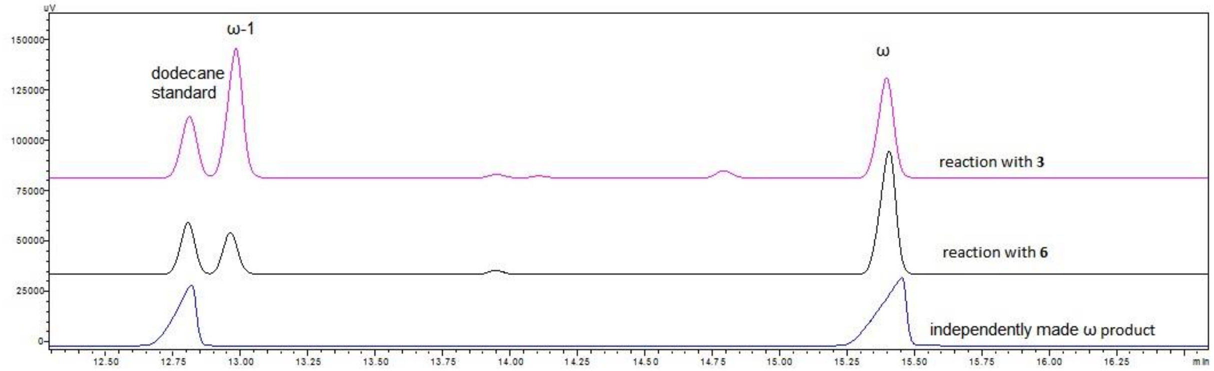

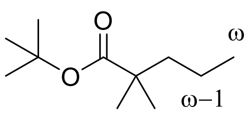

GC assignments supported by ^{1}H NMR spectrum analysis.

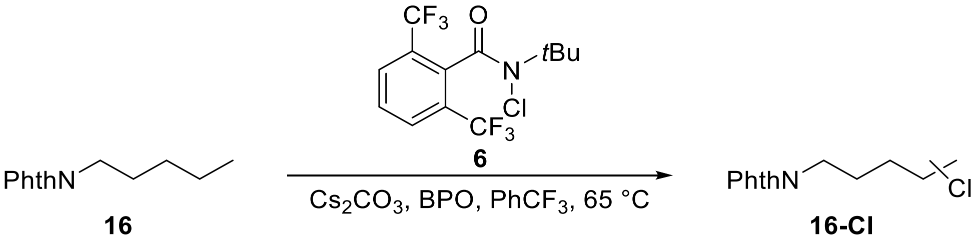

Reaction with *N*-*n*-pentyl phthalimide: Prepared according to General Procedure A with 1.5 equiv of chloroamide on 0.144 mmol scale using chloroamide **6** and n-pentyl phthalimide giving 80% GC yield. GC data obtained using Method 4.

Table S19. GC data for functionalization of *N*-*n*-pentyl phthalimide with 6.

Product	Retention Time	Peak Area
ω-2	11.435	1.490
ω-1	12.028	65.099
ω	12.859	33.411

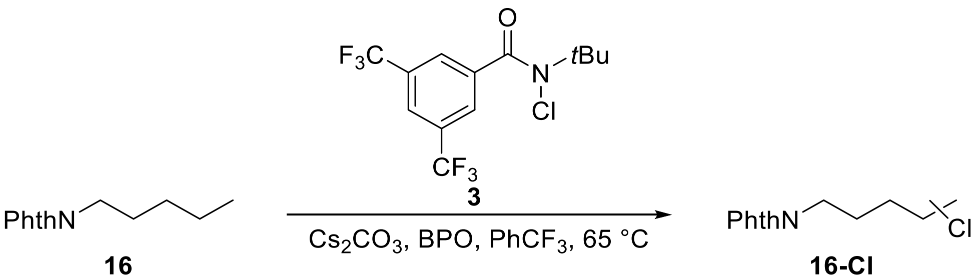

Reaction with *N*-*n*-pentyl phthalimide: Prepared according to General Procedure A with 1.5 equiv of chloroamide on 0.144 mmol scale using chloroamide **3** and n-pentyl phthalimide giving 77% GC yield. GC data obtained using Method 4.

Table S20. GC data for functionalization of *N*-*n*-pentyl phthalimide phthalimide with 3.

Product	Retention Time	Peak Area
ω-2	11.437	4.412
ω-1	12.033	84.488
ω	12.843	11.100

Figure S19. Chromatograph of *N*-*n*-pentyl phthalimide functionalizations

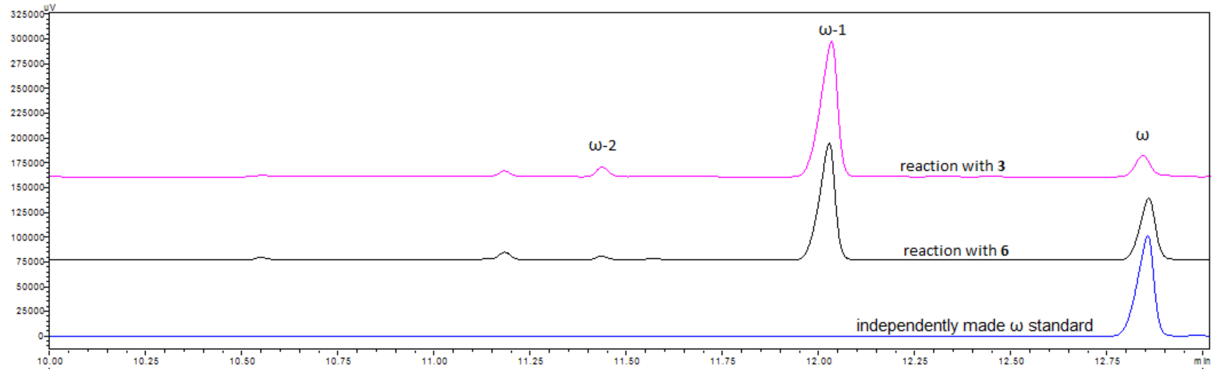

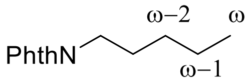

GC assignments made in agreement with previous reports.[1]

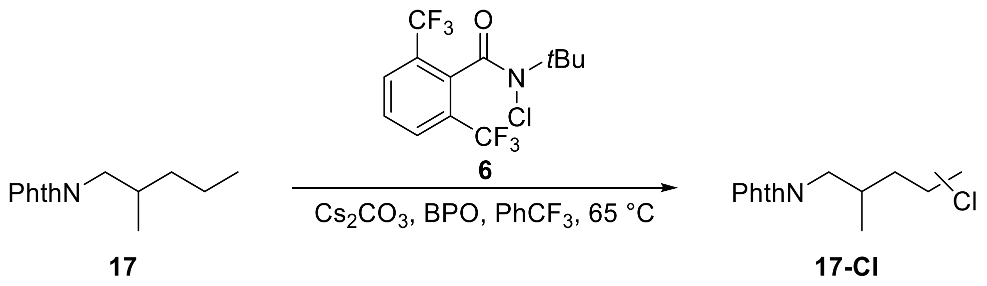

Reaction with *N*-(2-methyl pentyl) phthalimide: Prepared according to General Procedure A with 1.5 equiv of chloroamide on 0.144 mmol scale using chloroamide **6** and *N*-(2-methyl pentyl) phthalimide giving 74% GC yield. GC data obtained using Method 4.

Table S21. GC data for functionalization of *N*-(2-methyl pentyl) phthalimide with 6.

Product	Retention Time	Peak Area
ω-1	12.020	24.059
ω-1	12.155	27.447
ω-2	12.502	0.986
β	12.618	2.660
ω	13.127	44.849

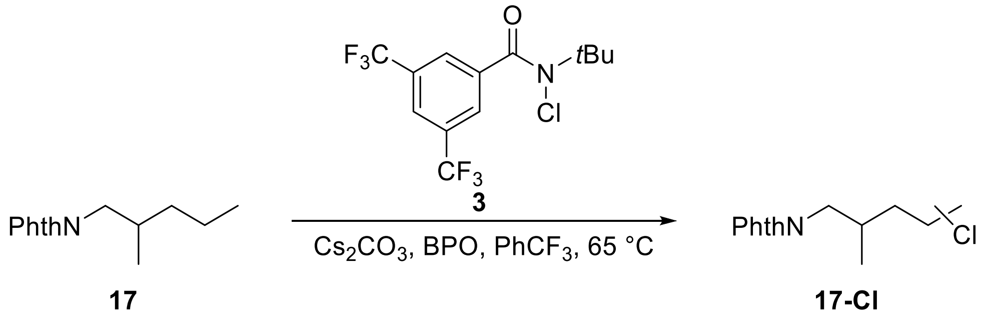

Reaction with *N*-(2-methyl pentyl) phthalimide: Prepared according to General Procedure A with 1.5 equiv of chloroamide on 0.144 mmol scale using chloroamide **3 and** *N*-(2-methyl pentyl) phthalimide giving 74% GC yield. GC data obtained using Method 4.

Table S22. GC data for functionalization of *N*-(2-methyl pentyl) phthalimide with 3.

Product	Retention Time	Peak Area
ω-1	12.035	33.206
ω-1	12.173	40.754
ω-2	12.513	4.170
β	12.626	1.082
ω	13.120	20.787

Figure S20. Chromatograph of *N*-(2-methyl pentyl) phthalimide functionalizations

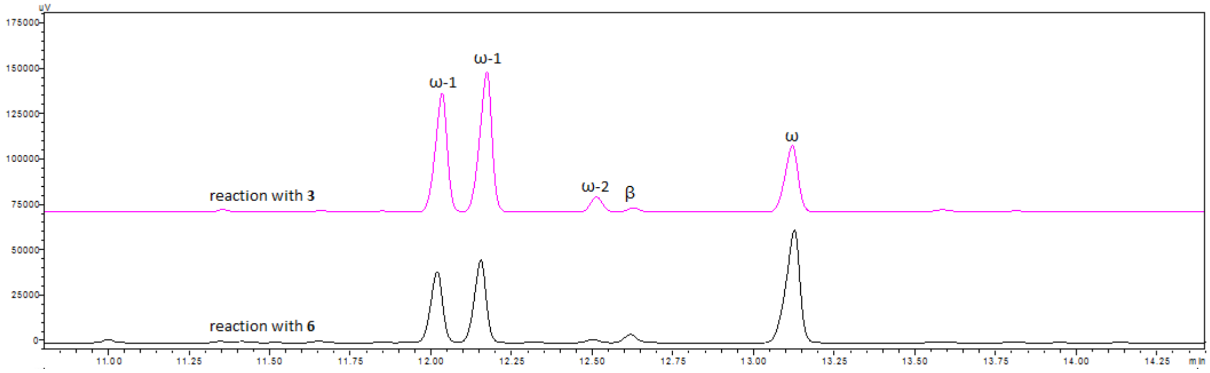

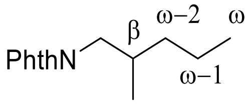

GC assignments made in agreement with previous reports.[1]

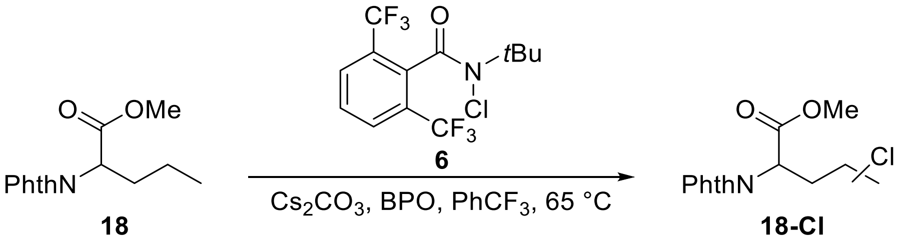

Reaction with *N*-phthaloylnorvaline methyl ester: Prepared according to General Procedure B with 3 equiv of substrate on 0.144 mmol scale using chloroamide **6** and N-phthaloylnorvaline methyl ester giving 45% NMR yield. Chloride products assigned based on analogy to norleucine derivative.[1]

Figure S21. ^{1}H NMR of crude reaction of *N*-phthaloylnorvaline methyl ester with 6.

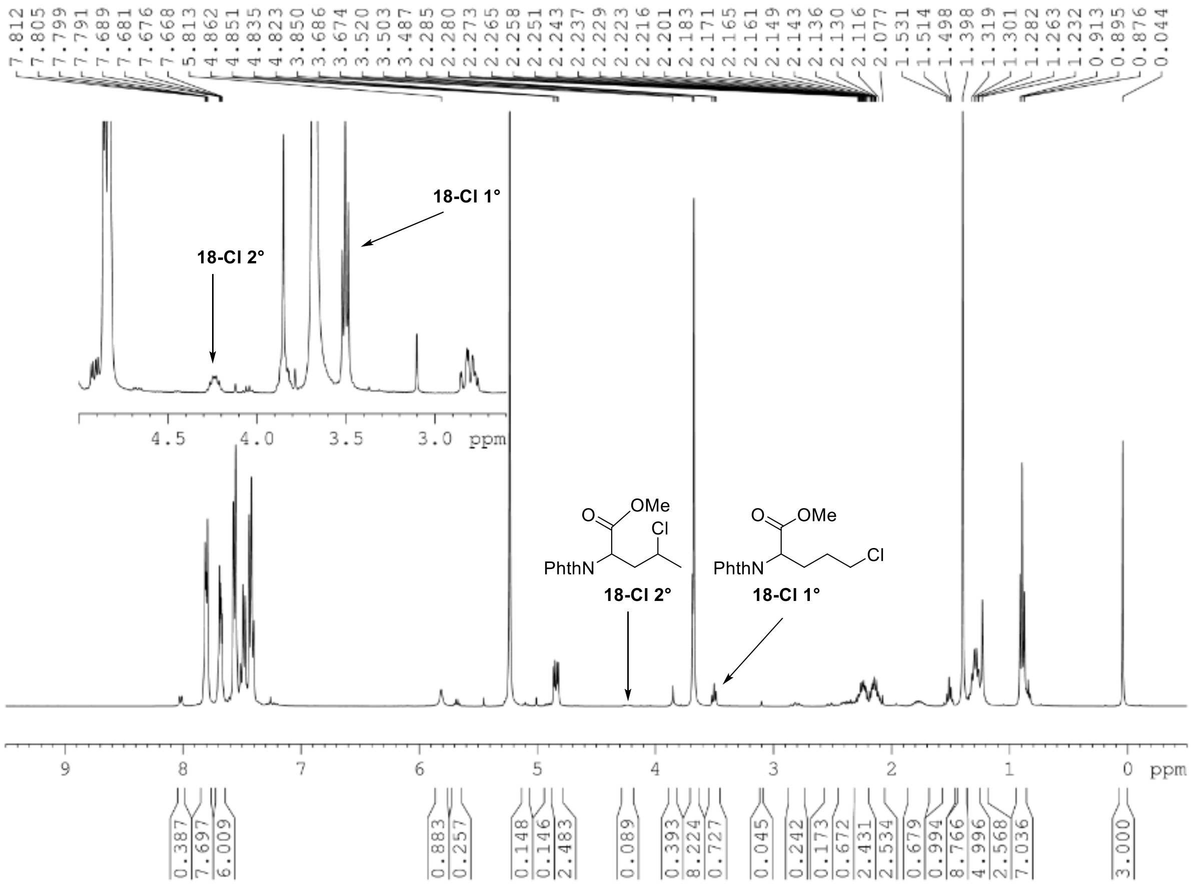

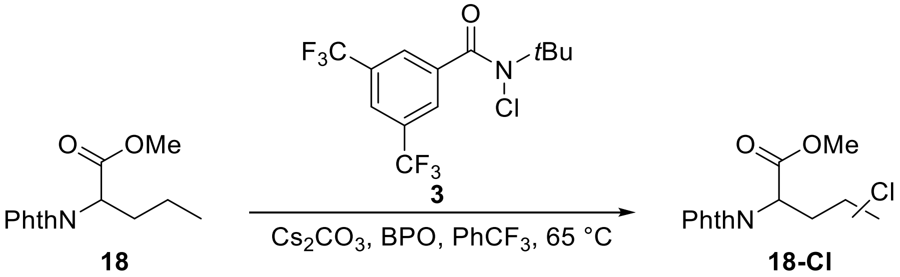

Reaction with *N*-phthaloylnorvaline methyl ester: Prepared according to General Procedure B with 3 equiv of substrate on 0.144 mmol scale using chloroamide **3** and N-phthaloylnorvaline methyl ester giving 39% NMR yield. Chloride products assigned based on analogy to norleucine derivative.[1]

Figure S22. *N*-phthaloylnorvaline methyl ester

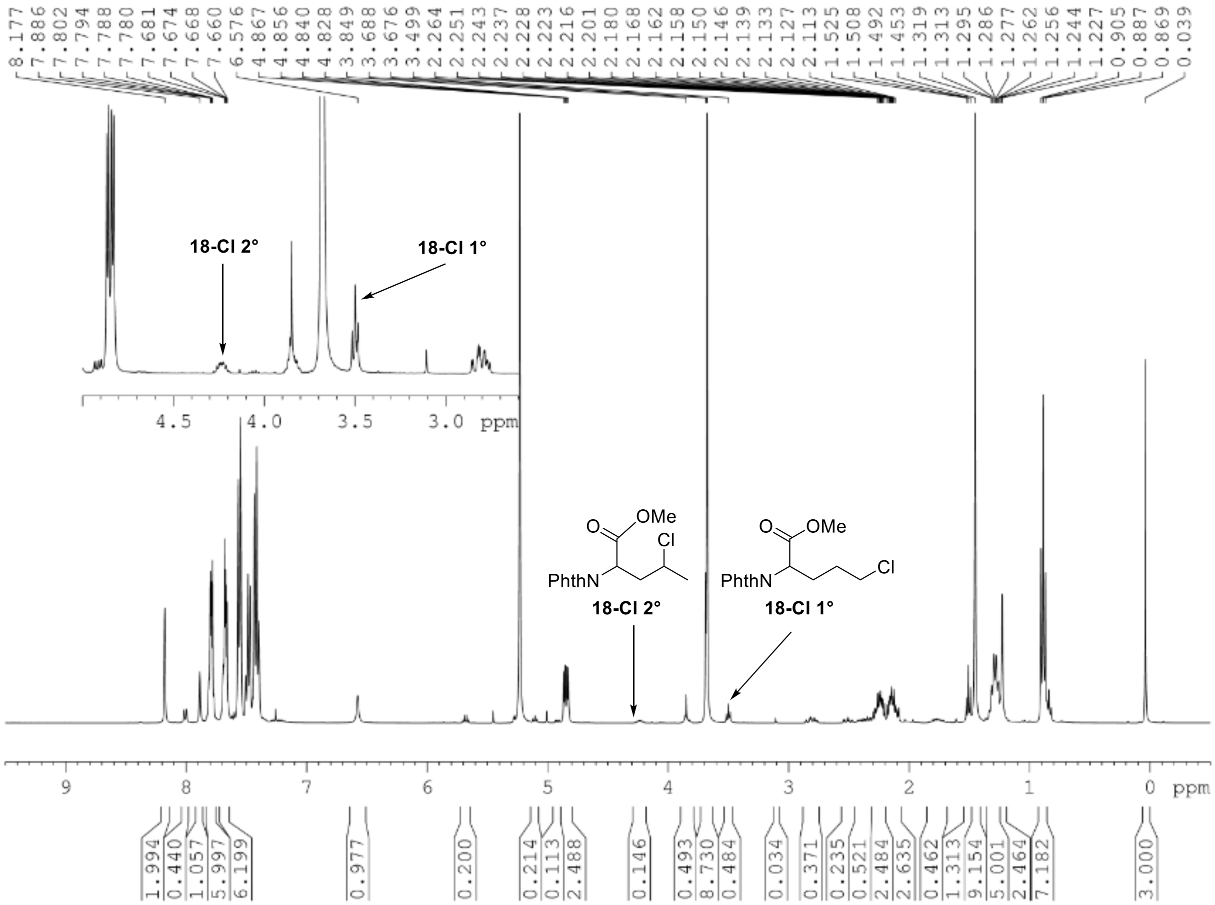

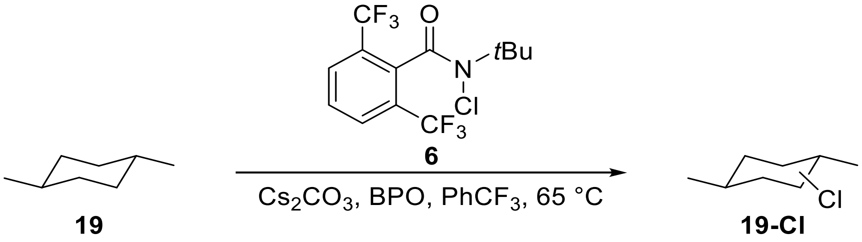

Reaction with *trans*-1,4-dimethylcyclohexane: Prepared according to General Procedure A with 1 equiv of chloroamide on 0.144 mmol scale using chloroamide **6** and *trans*-1,4-dimethylcyclohexane giving 54% GC yield. GC data obtained using Method 3.

Table S23. GC data for functionalization of *trans*-1,4-dimethylcyclohexane with 6.

Product	Retention Time	Peak Area
2°	5.607	38.20
2°	5.912	31.73
1°	7.108	30.08

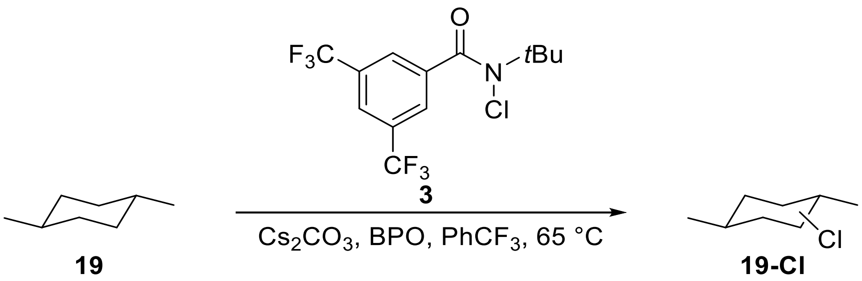

Reaction with *trans*-1,4-dimethylcyclohexane: Prepared according to General Procedure A with 1 equiv of chloroamide on 0.144 mmol scale using chloroamide **3** and *trans*-1,4-dimethylcyclohexane giving 75% GC yield. GC data obtained using Method 3.

Table S24. GC data for functionalization of *trans*-1,4-dimethylcyclohexane with 3.

Product	Retention Time	Peak Area
2°	5.628	50.74
2°	5.932	44.62
1°	7.120	4.65

Figure S23. Chromatograph of *trans*-1,4-dimethylcyclohexane functionalizations

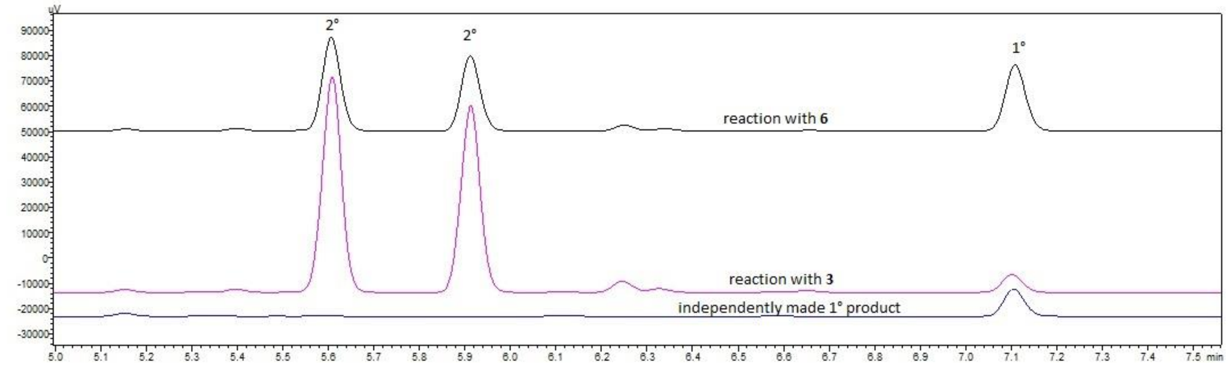

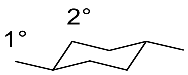

GC assignments supported by ^{1}H NMR spectrum analysis.

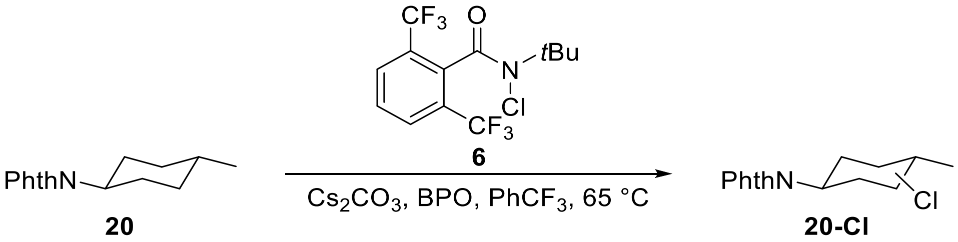

Reaction with *trans*-4-methylcyclohexylphthalimide: Prepared according to General Procedure B with 3 equiv of substrate on 0.144 mmol scale using chloroamide **6** and *trans*-4-methylcyclohexylphthalimide giving 64% GC yield. GC data obtained using Method 4.

Table S25. GC data for functionalization of *trans*-4-methylcyclohexylphthalimide with 6.

Product	Retention Time	Peak Area
2-Cl	14.168	23.17
3-Cl	15.412	23.33
3-Cl	15.651	18.09
1°	16.884	35.42

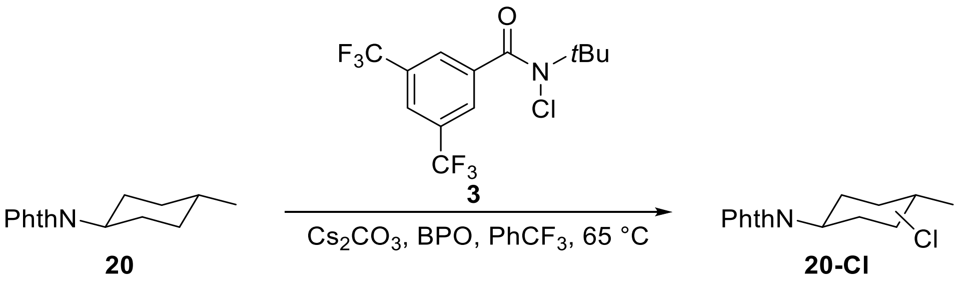

Reaction with *trans*-4-methylcyclohexylphthalimide: Prepared according to General Procedure B with 3 equiv of substrate on 0.144 mmol scale using chloroamide **3** and *trans*-4-methylcyclohexylphthalimide *trans*-4-methylcyclohexylphthalimide giving 76% GC yield. GC data obtained using Method 4.

Table S26. GC data for functionalization of *trans*-4-methylcyclohexylphthalimide with 3.

Product	Retention Time	Peak Area
2-Cl	14.173	26.25
3-Cl	15.426	29.22
3-Cl	15.679	38.09
1°	16.848	6.44

Figure S24. Chromatograph of *trans*-4-methylcyclohexylphthalimide functionalizations

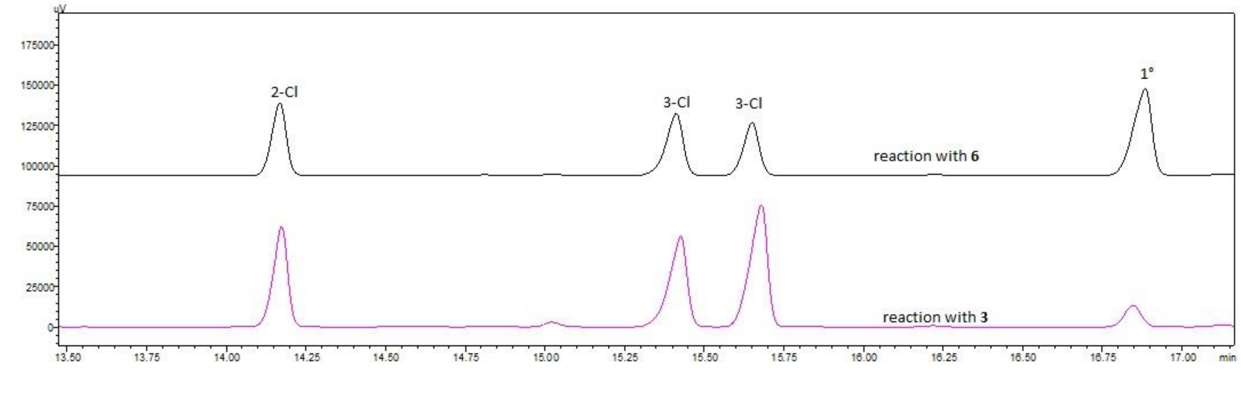

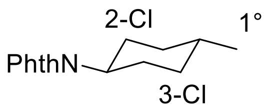

GC assignments supported by ^1H NMR spectrum analysis.

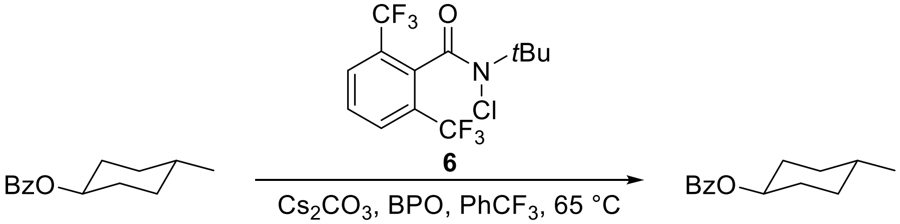

21 **21-Cl**

Reaction with *trans*-4-methylcyclohexyl benzoate: Prepared according to General Procedure A with 1 equiv of chloroamide on 0.144 mmol scale using chloroamide **6** and *trans*-4-methylcyclohexyl benzoate giving 41% GC yield. GC data obtained using Method 4.

Table S27. GC data for functionalization of *trans*-4-methylcyclohexyl benzoate with 6.

Product	Retention Time	Peak Area
3°	10.552	3.007
2-Cl	10.862	8.075
2-Cl	10.906	7.709
3°	10.986	3.481
3-Cl	11.153	19.057
3-Cl	11.487	19.169
1°	12.112	39.502

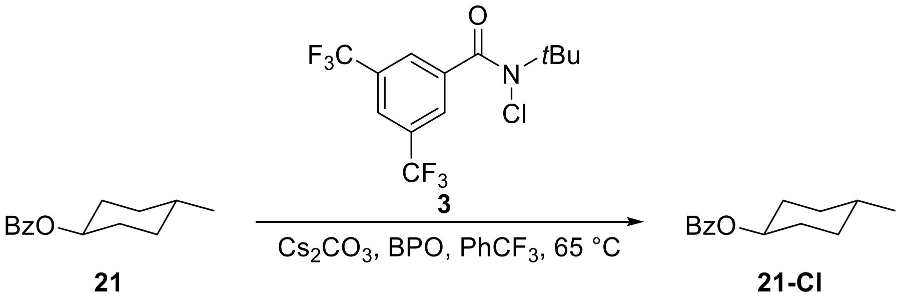

21 **21-Cl**

Reaction with *trans*-4-methylcyclohexyl benzoate: Prepared according to General Procedure A with 1 equiv of chloroamide on 0.144 mmol scale using chloroamide **3** and *trans*-4-methylcyclohexyl benzoate giving 49% GC yield. GC data obtained using Method 4.

Table S28. GC data for functionalization of *trans*-4-methylcyclohexyl benzoate with 3.

Product	Retention Time	Peak Area
3°	10.440	12.308
2-Cl	10.743	13.097
2-Cl	10.785	8.759
3°	10.866	15.931
3-Cl	11.027	18.940
3-Cl	11.354	22.955
1°	11.953	8.010

Figure S25. Chromatograph of *trans*-4-methylcyclohexyl benzoate functionalizations

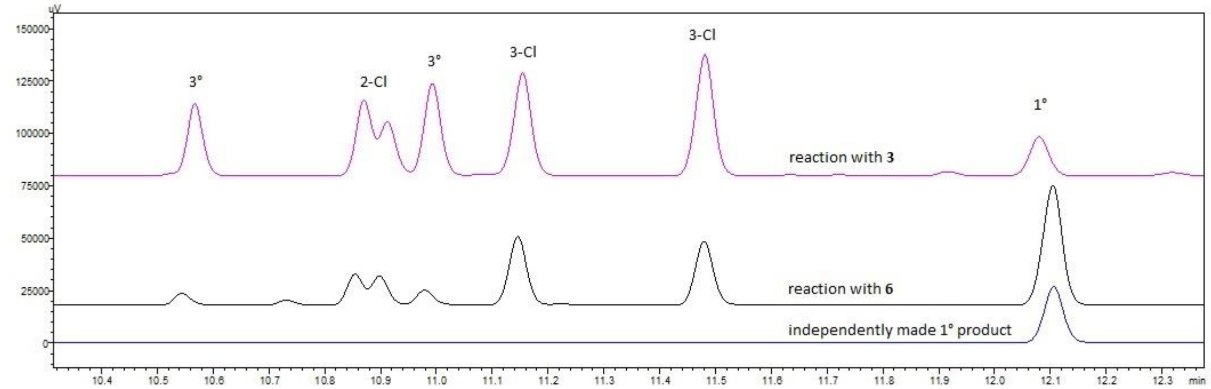

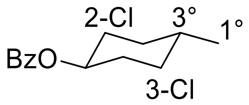

GC assignments supported by ^{1}H NMR spectrum analysis.

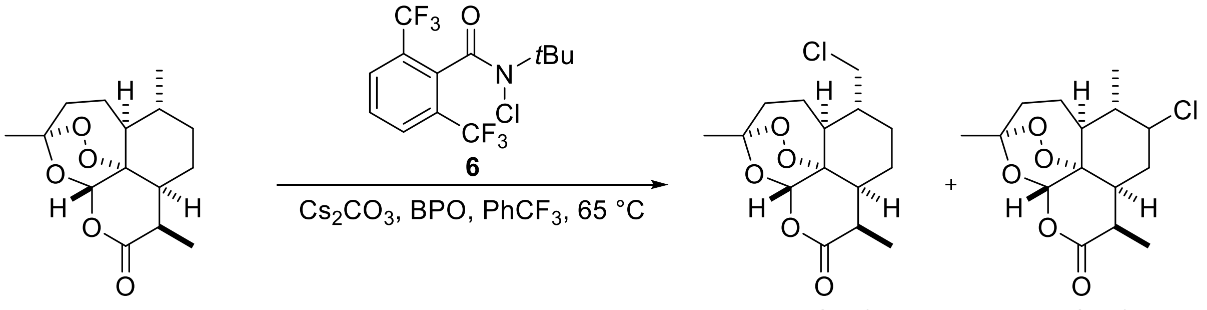

Reaction with Artemisinin: Prepared according to General Procedure D on 0.144 mmol scale using chloroamide **6** and Artemisinin. The crude reaction mixture was subjected to flash chromatography (10 % ethyl acetate in hexanes) where products **22-Cl** and unreacted Artemisinin were recovered. The unreacted Artemisinin was resubjected to conditions according to General Procedure G. The products were isolated and combined to give 27.0 mg of chlorinated products (0.085 mmol, 59% yield). The

stereochemistry of the secondary chloride **22-Cl** was assigned by analogy to the hydroxylation of artemisinin reported by Fasan.[7]

Figure S26. ^{1}H NMR of isolated products 22-Cl of reaction of Artemisinin with 6.

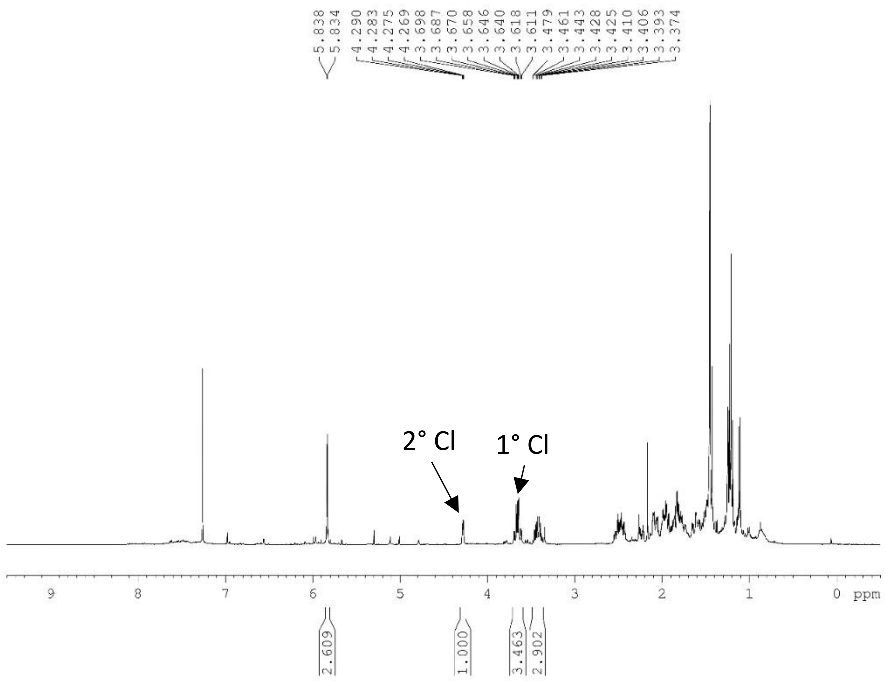

Figure S27. ^{13}C NMR of isolated products 22-Cl of reaction of Artemisinin with 6.

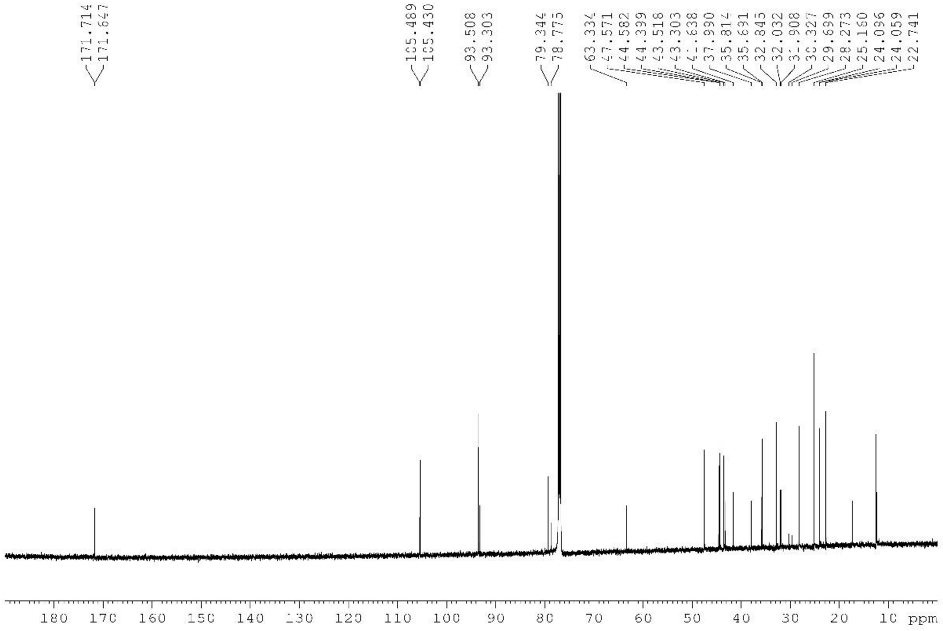

Figure S28. ^{13}**C NMR DEPT 135 of isolated products 22-Cl of reaction of Artemisinin with 6.**

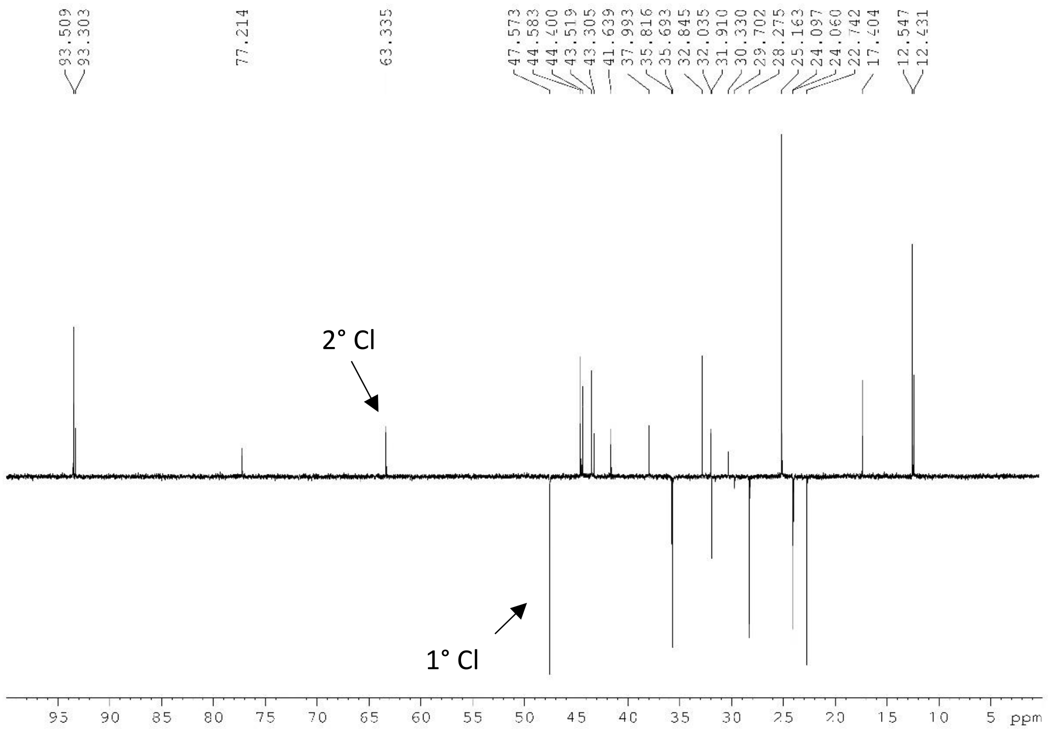

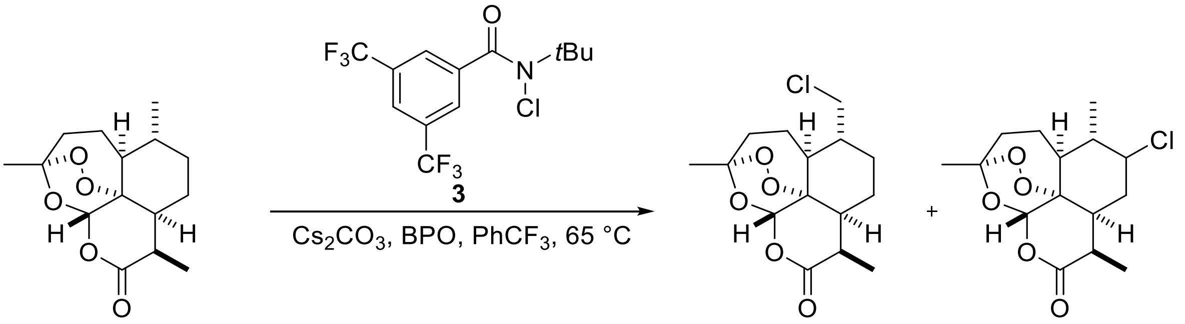

22 **22-Cl 1°** **22-Cl 2°**

Reaction with artemisinin: Prepared according to General Procedure D on 0.144 mmol scale using chloroamide **6** and artemisinin giving a 28% NMR yield.

Figure S29. ^{1}H NMR of crude reaction of artemisinin with 3.

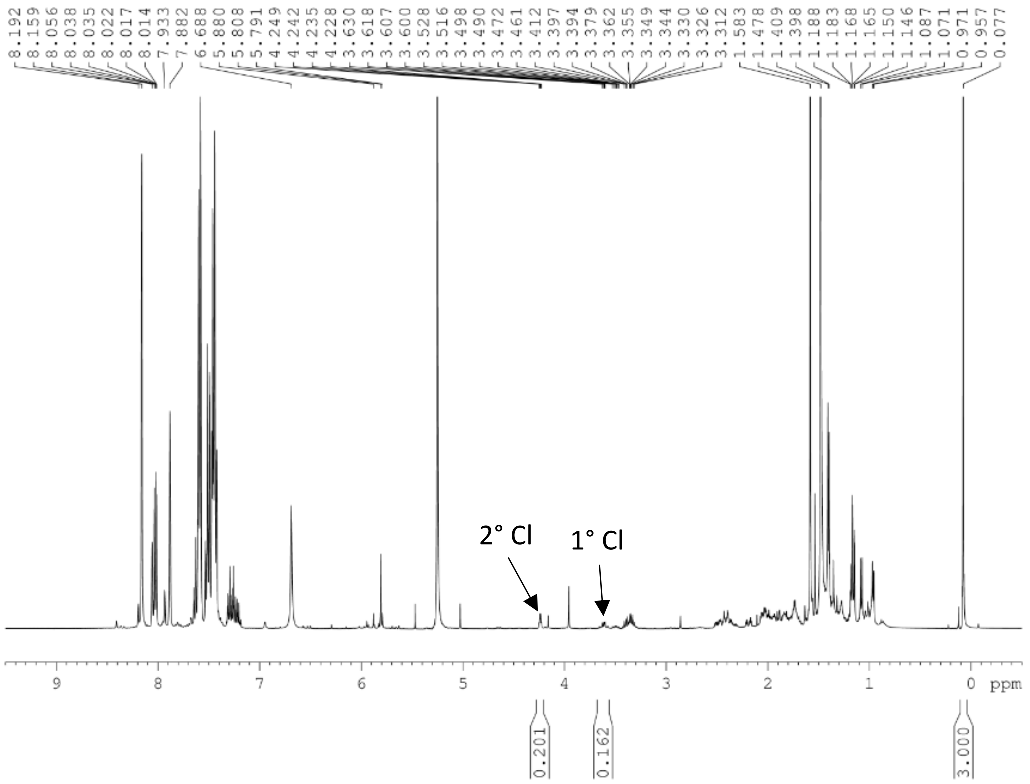

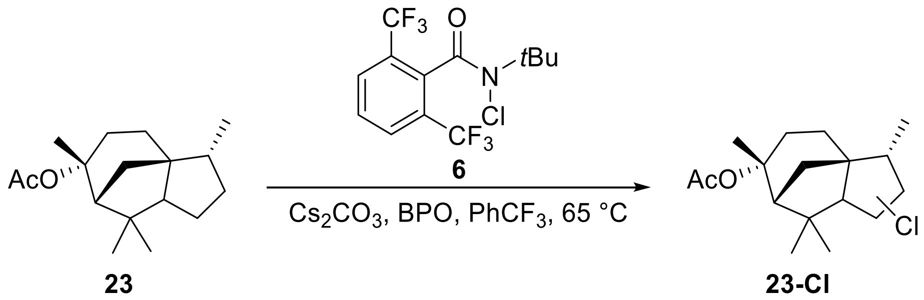

Reaction with cedryl acetate: Prepared according to General Procedure A with 1 equiv of chloroamide on 0.144 mmol scale using chloroamide **6** and cedryl acetate giving 45% NMR yield. GC data obtained using Method 4.

Table S29. GC data for functionalization of cedryl acetate with 6.

Product	Retention Time	Peak Area
3-Cl	11.659	11.398
2-Cl	11.890	13.716
2-Cl	12.143	36.862
1°	12.673	38.024

Figure S30. ^{1}H NMR of crude reaction of cedryl acetate with 6.

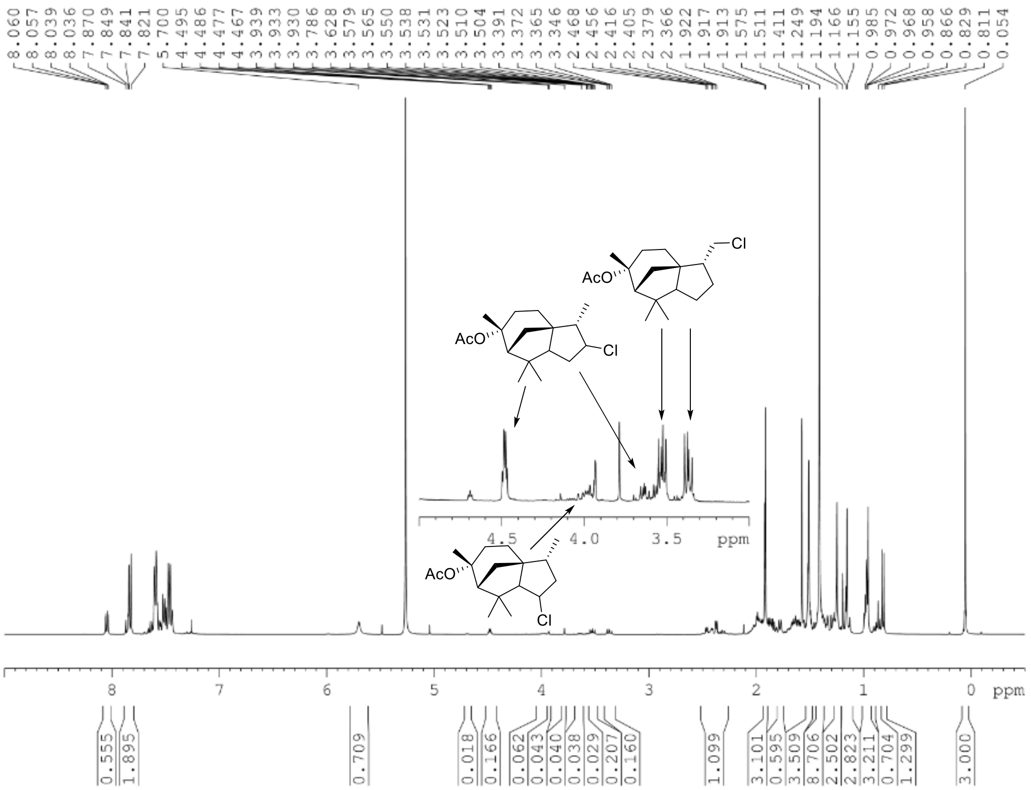

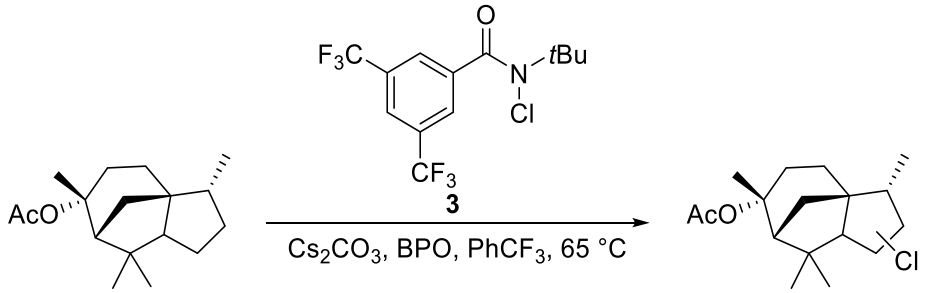

23 **23-Cl**

Reaction with cedryl acetate: Prepared according to General Procedure A with 1 equiv of chloroamide on 0.576 mmol scale using chloroamide **3** and cedryl acetate giving 61% NMR yield. GC data obtained using Method 4.

Table S30. GC data for functionalization of cedryl acetate with 3.

Product	Retention Time	Peak Area
3-Cl	11.671	35.093
2-Cl	11.891	19.503
2-Cl	12.139	33.57

1°	12.642	11.834

Figure S31. ^{1}H NMR of crude reaction of cedryl acetate with 3.

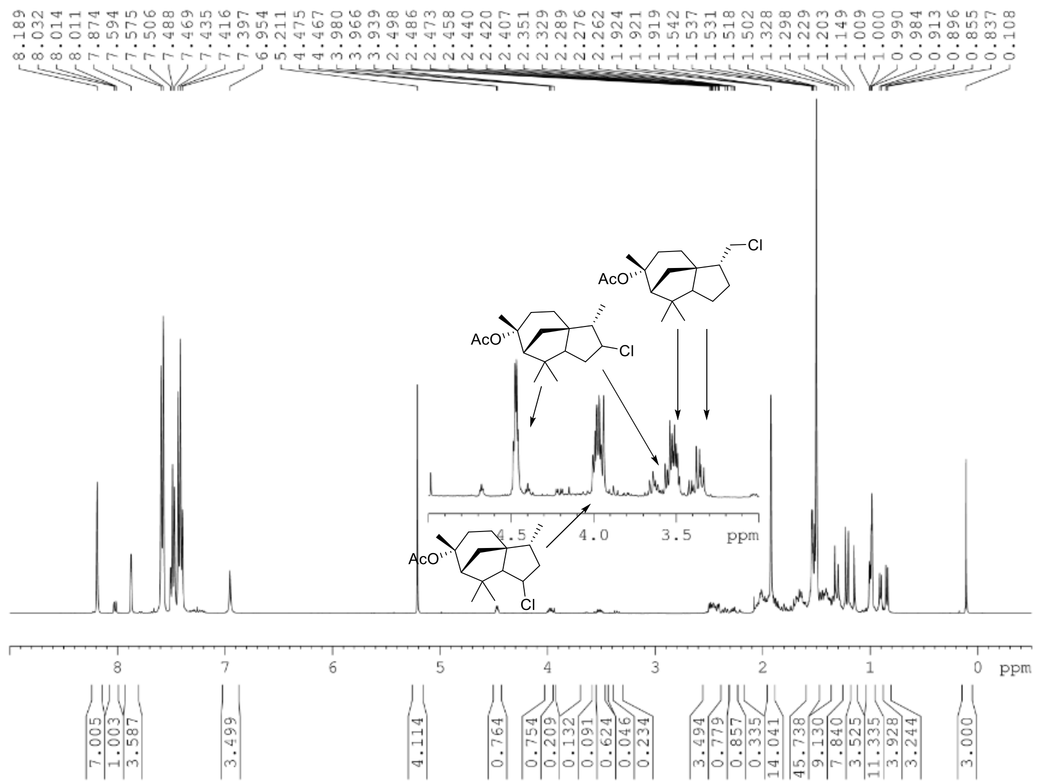

Figure S32. Chromatograph of cedryl acetate functionalizations

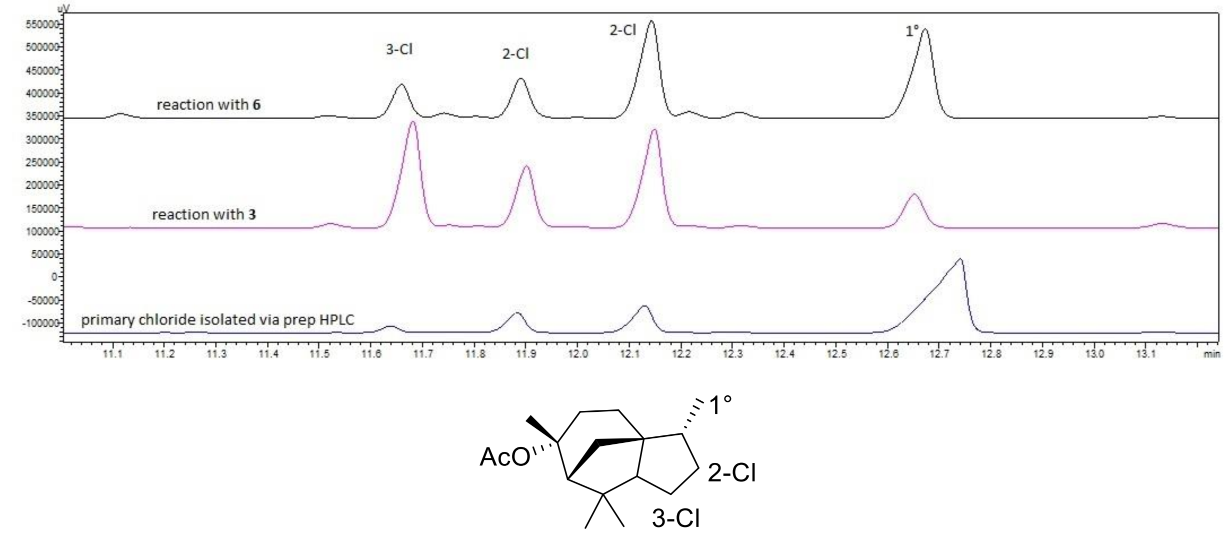

GC assignments supported by ^{1}H NMR spectrum analysis.

Product assignments for cedryl acetate were determined using multiple 1D and 2D NMR experiments including ^{1}H, ^{13}C, DEPT 135, COSY, NOESY, TOCSY, HMQC, and HMBC after prep HPLC isolation. The peaks at 3.4 and 3.5 ppm in the ^{1}H NMR were determined to be diastereotopic protons because both peaks correlated with a single carbon peak at 46 ppm in the HMQC. Using DEPT 135, it can be determined that the carbon peak at 46 ppm corresponds to a methylene carbon signal which is in agreement with a primary functionalization. The peaks at 4.5, 4.0, and 3.6 ppm in the ^{1}H NMR corresponded to carbon peaks at 71.9, 58.4, and 66.3 ppm respectively in the HMQC. All 3 carbon peaks correspond to methine carbon signals in the DEPT 135 which is in agreement with secondary functionalization. Based on the TOCSY all 3 proton peaks correlated with 4 other proton signals, so all 3 proton peaks must correspond with functionalization on the 5 membered ring with the single methyl group.

FIGURE S33. ^{1}H NMR spectra of cedryl acetate functionalization with 6 after prep HPLC isolation

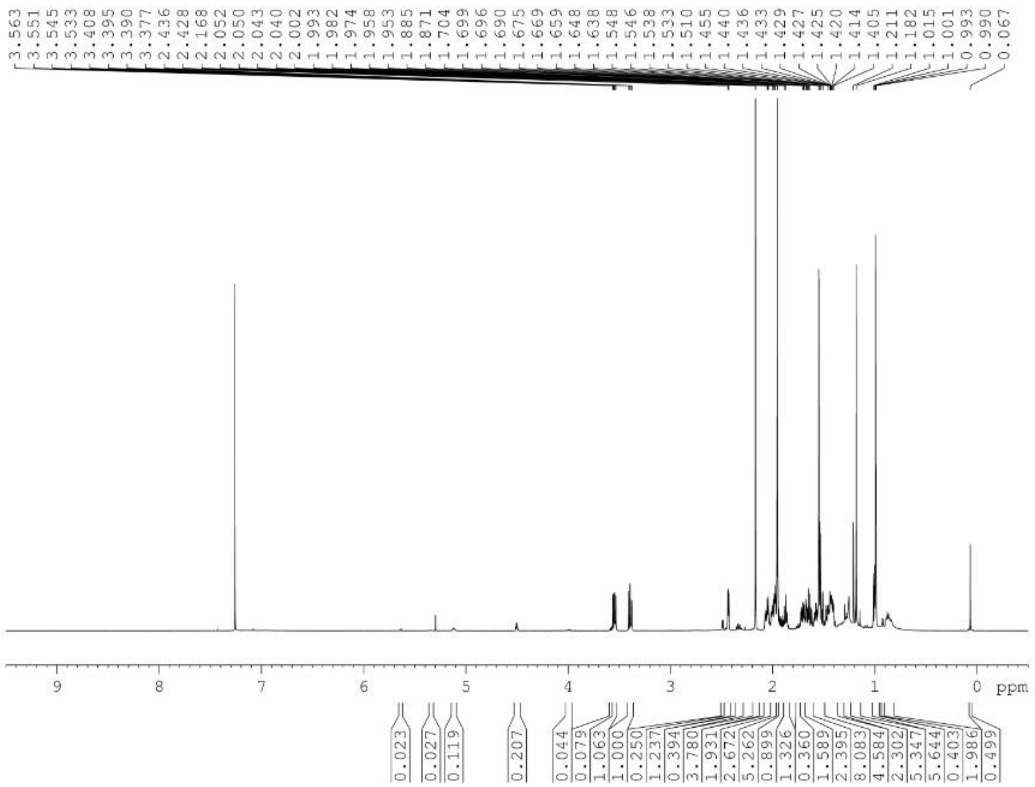

FIGURE S34. ^{13}C NMR spectra of cedryl acetate functionalization with 6 after prep HPLC isolation

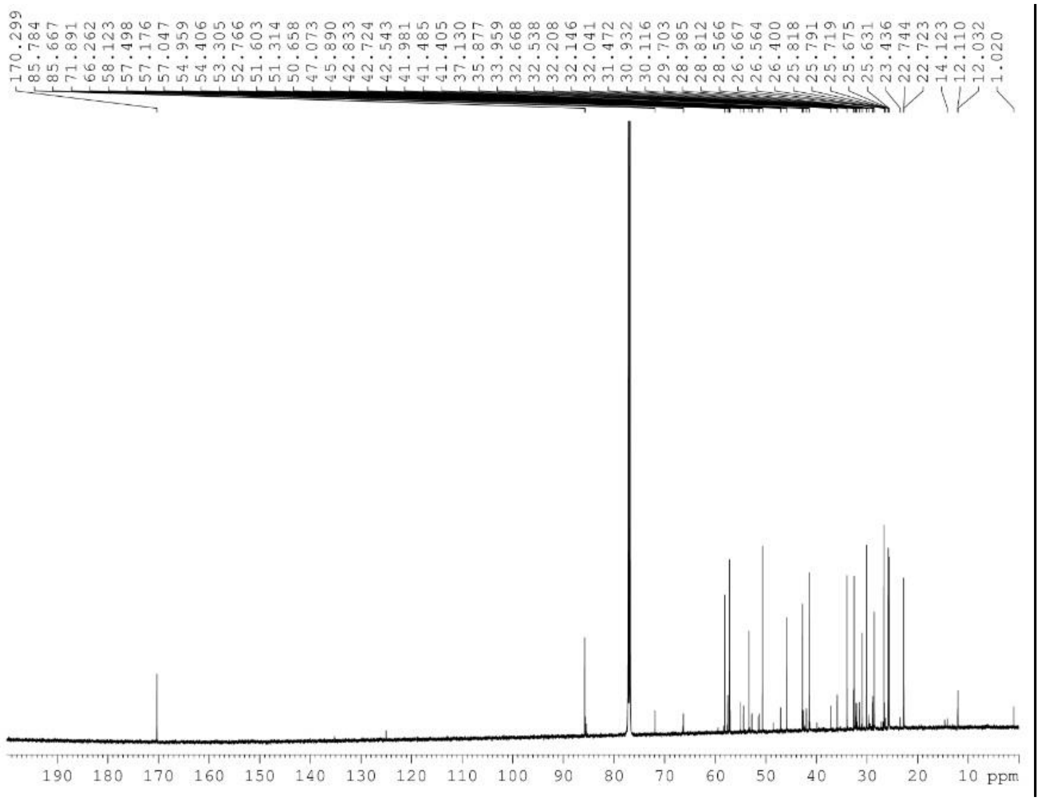

FIGURE S35. HMQC spectra of cedryl acetate functionalization with 6 after prep HPLC isolation

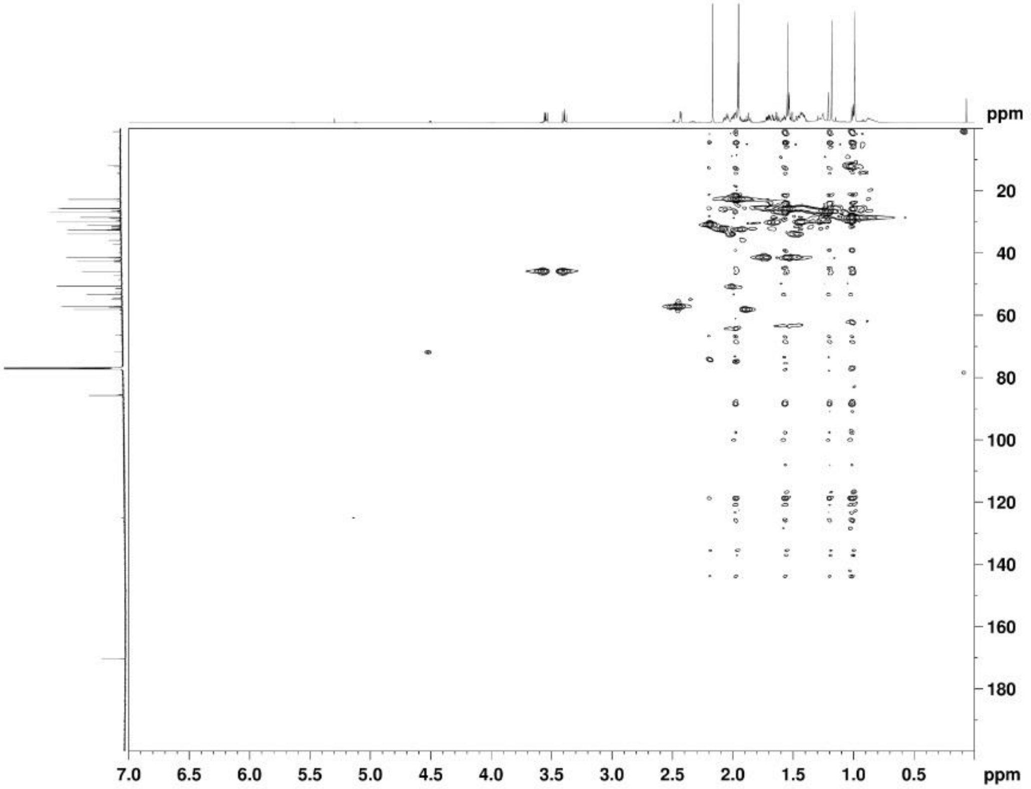

FIGURE S36. DEPT 135 spectra of cedryl acetate functionalization with 6 after prep HPLC isolation

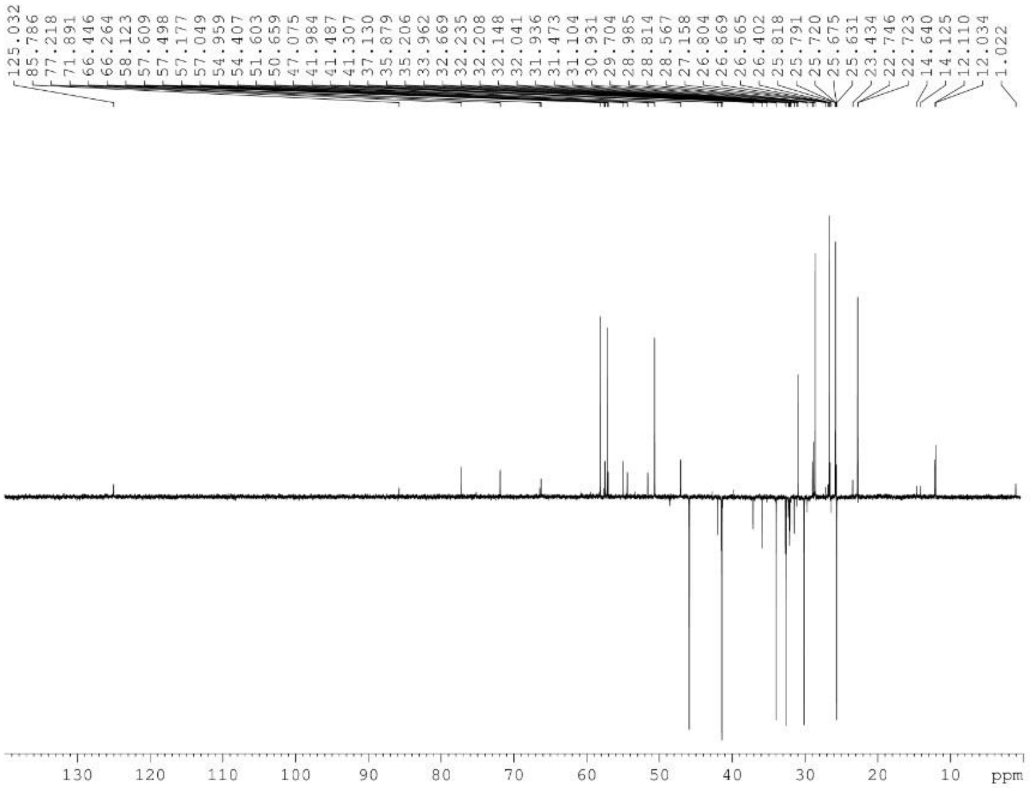

FIGURE S37. HMBC spectra of cedryl acetate functionalization with 6 after prep HPLC isolation

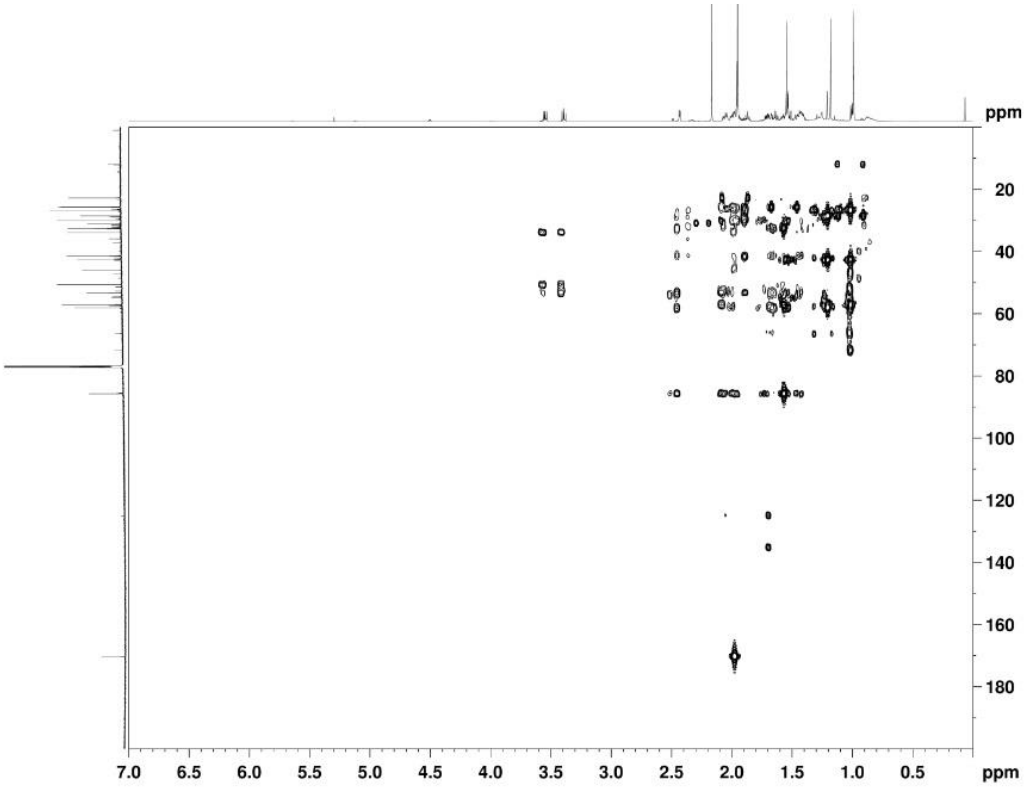

FIGURE S38. COSY spectra of cedryl acetate functionalization with 6 after prep HPLC isolation

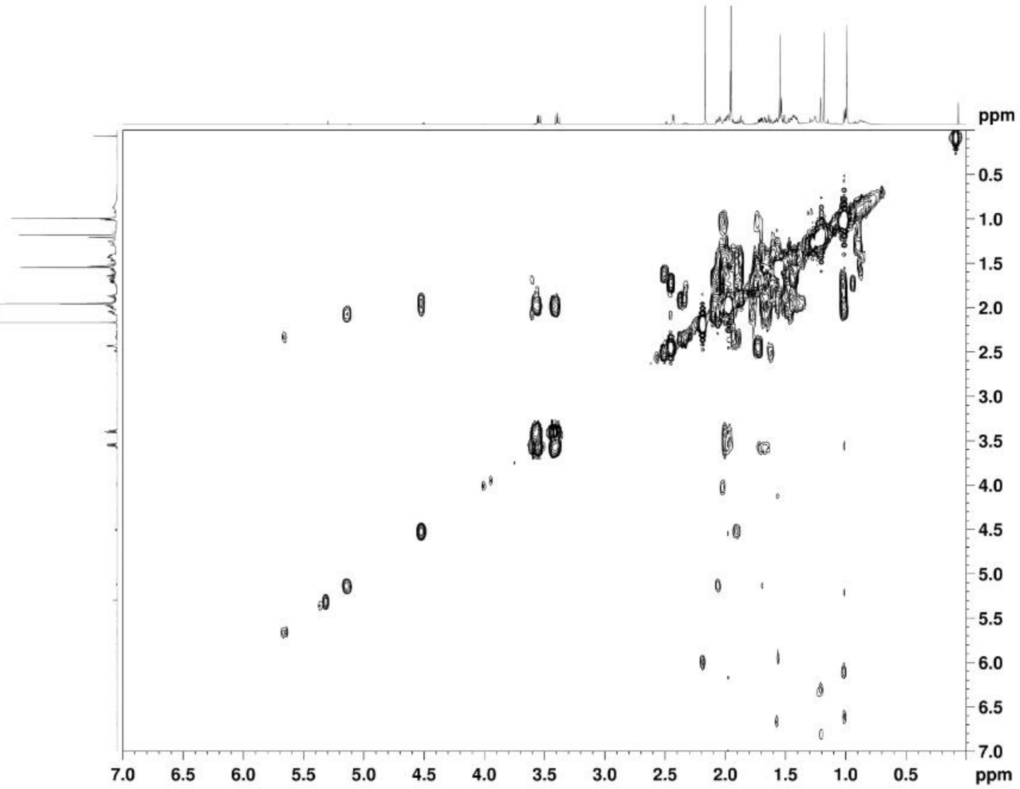

FIGURE S39. ^{1}H NMR spectra of cedryl acetate functionalization with 3 after prep HPLC isolation

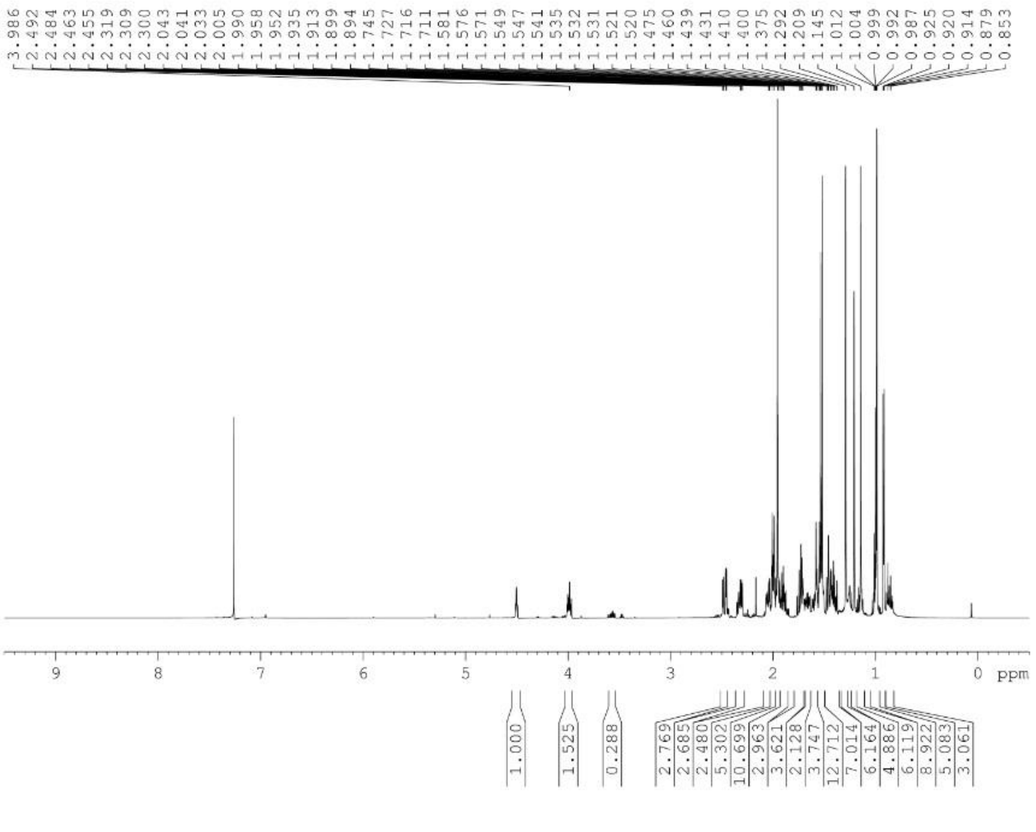

FIGURE S40. ^{13}C NMR spectra of cedryl acetate functionalization with 3 after prep HPLC isolation

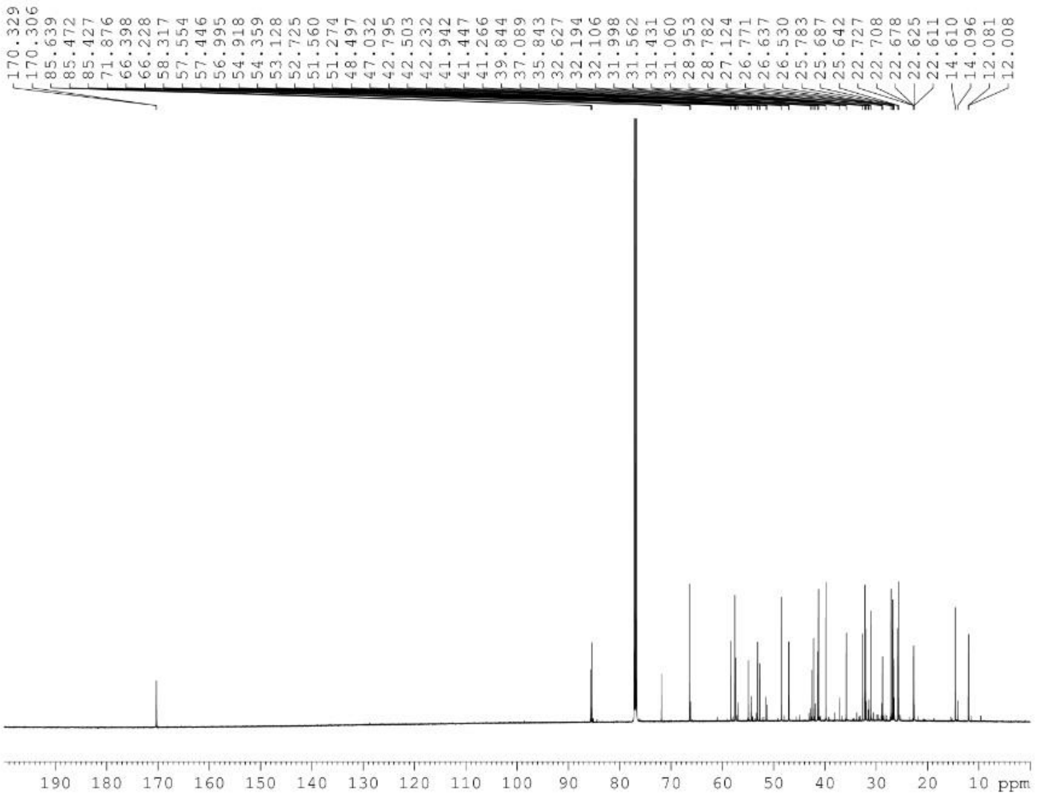

FIGURE S41. ^{13}C NMR spectra of cedryl acetate functionalization with 3 after prep HPLC isolation

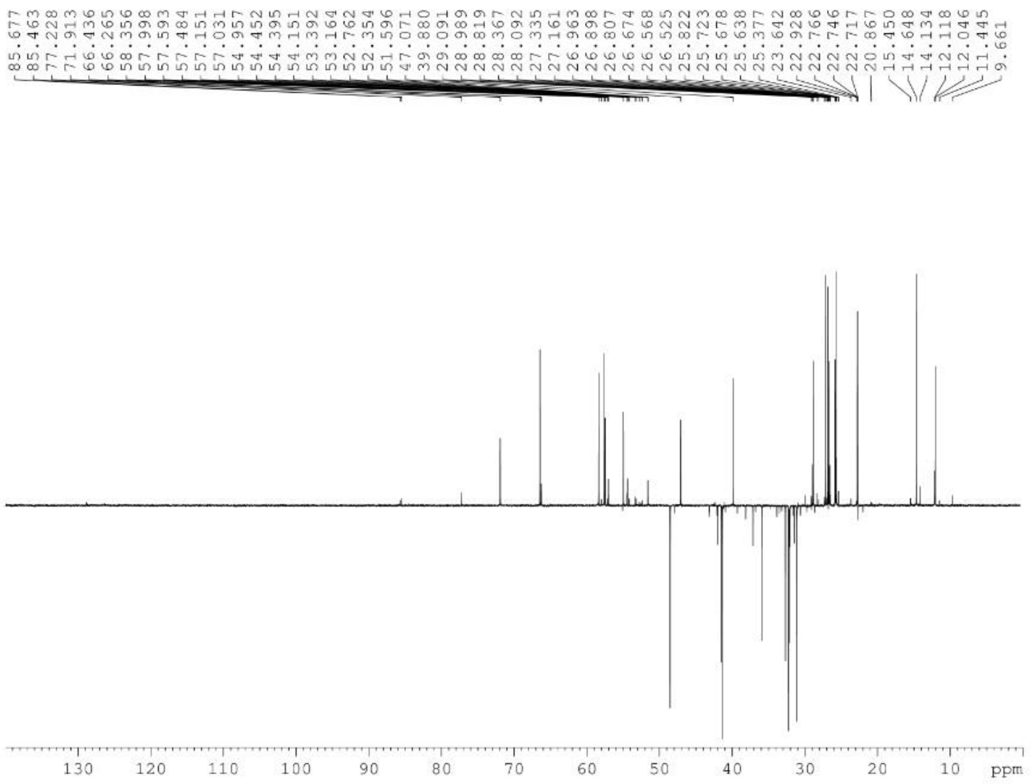

FIGURE S42. HMQC spectra of cedryl acetate functionalization with 3 after prep HPLC isolation

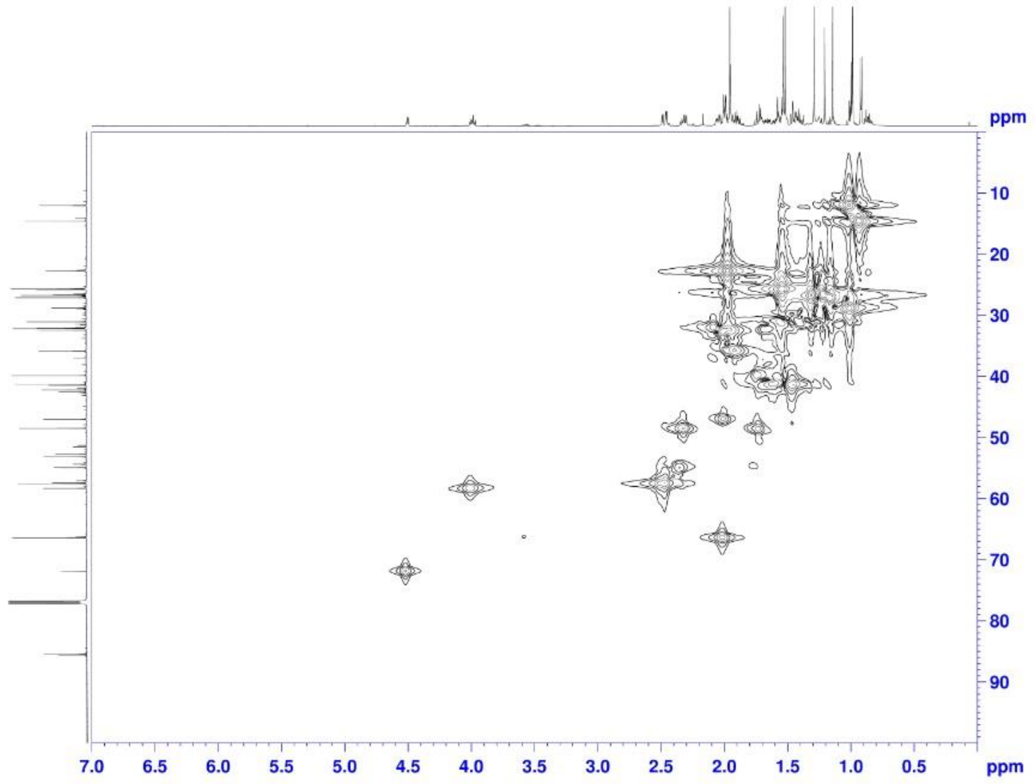

FIGURE S43. TOCSY spectra of cedryl acetate functionalization with 3 after prep HPLC isolation

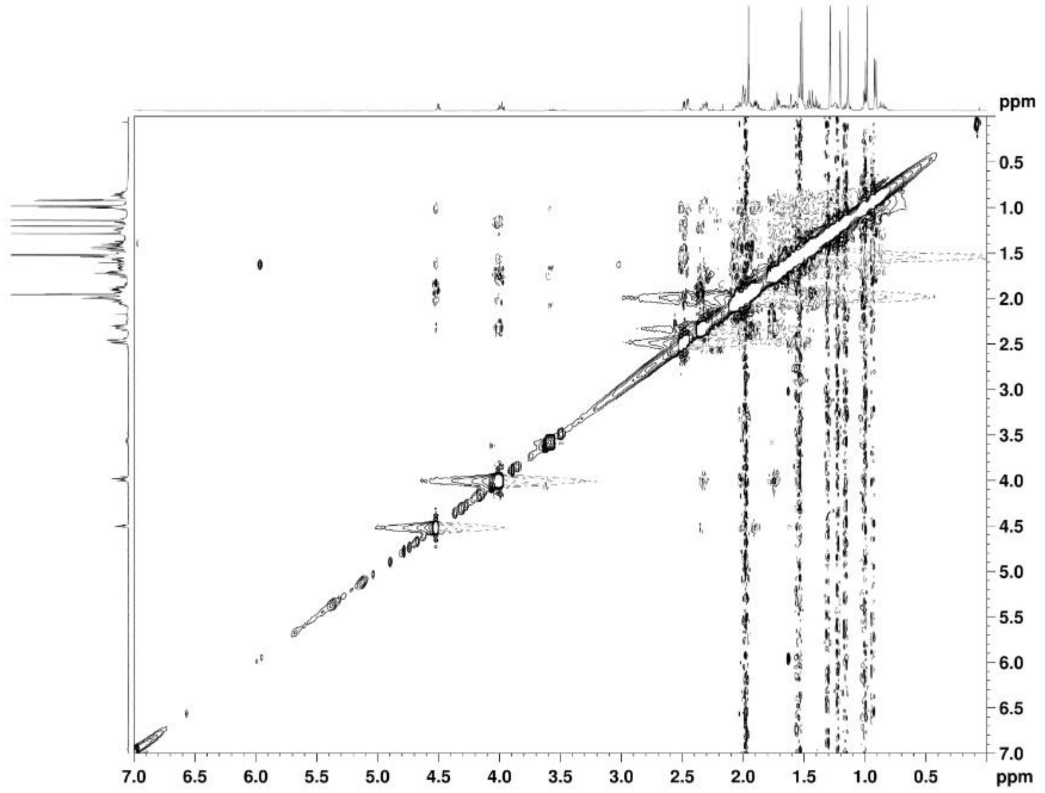

FIGURE S44. COSY spectra of cedryl acetate functionalization with 3 after prep HPLC isolation

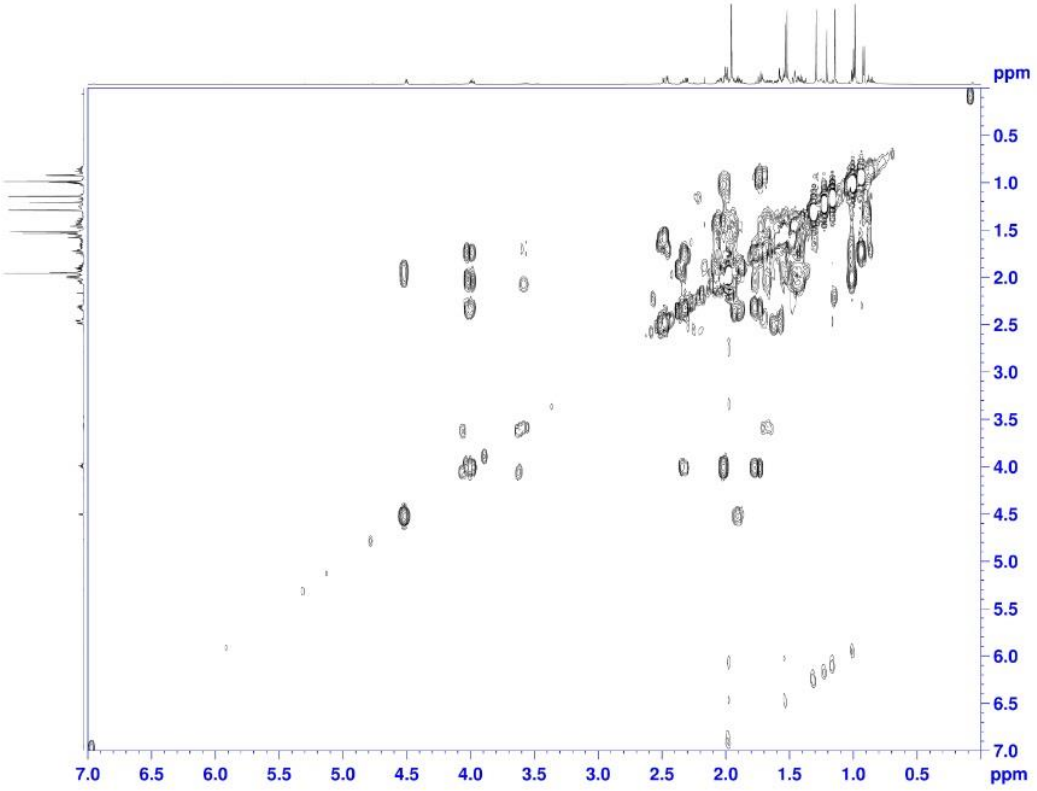

FIGURE S45. NOESY spectra of cedryl acetate functionalization with 3 after prep HPLC isolation

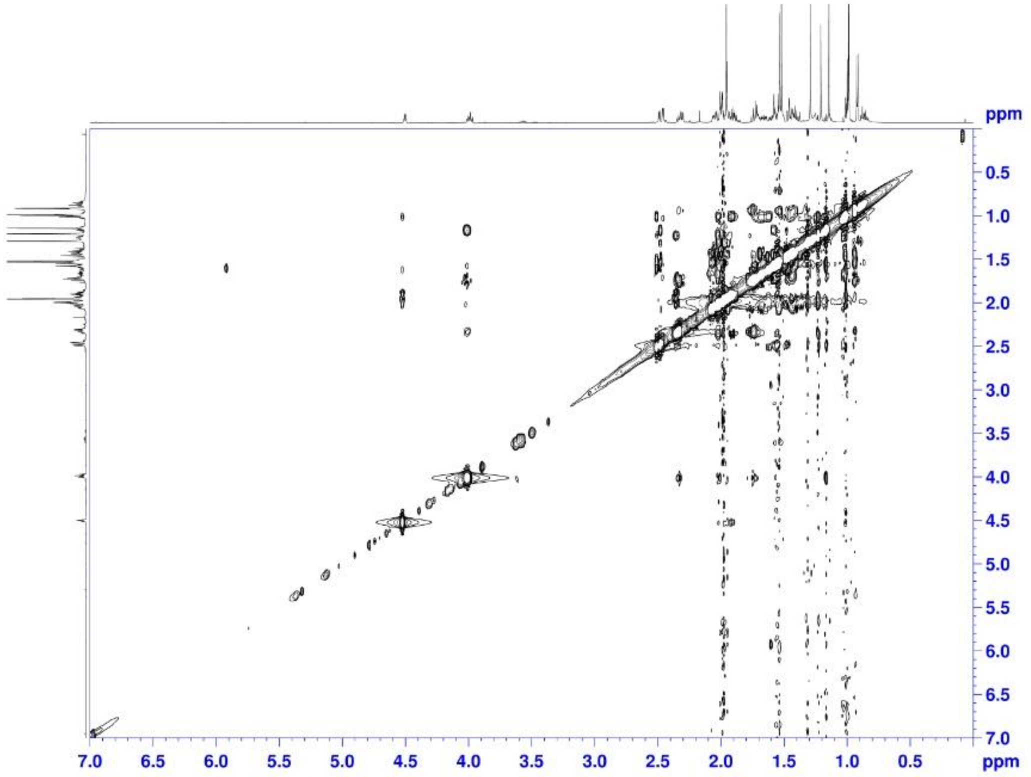

FIGURE S46. HMBC spectra of cedryl acetate functionalization with 3 after prep HPLC isolation

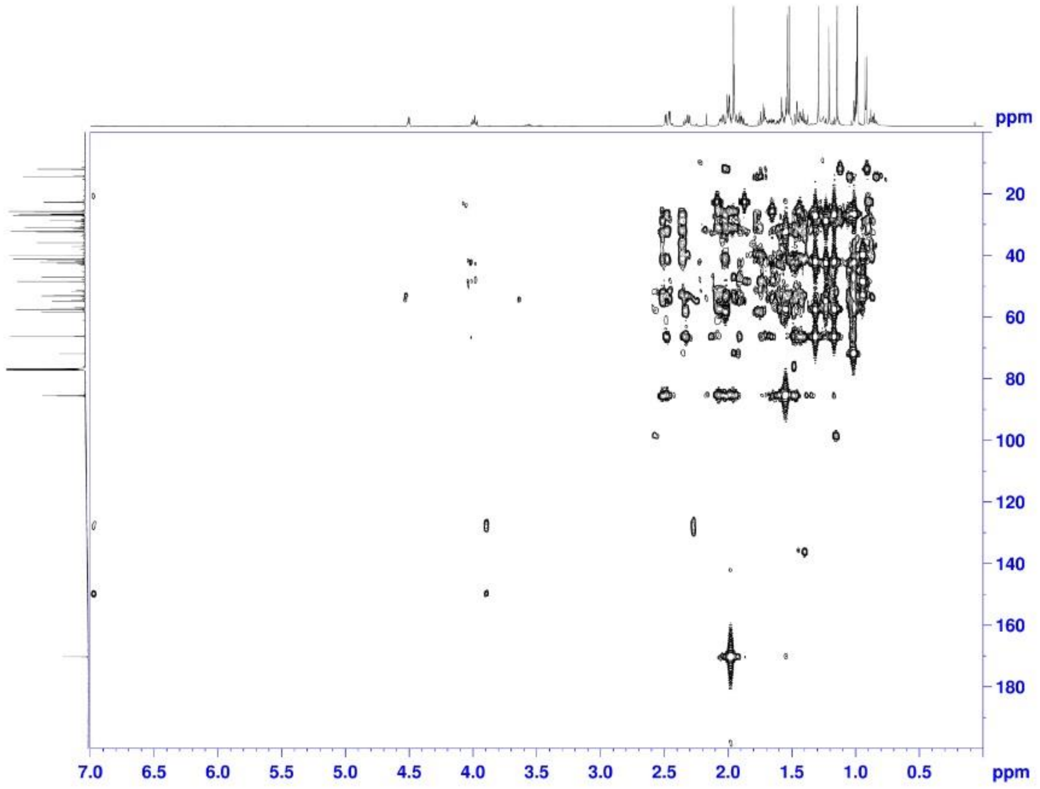

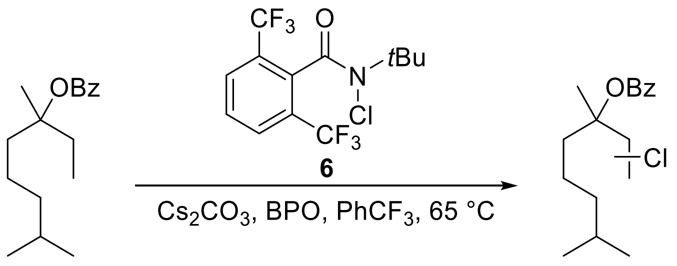

24 **24-Cl**

Reaction with tetrahydrolinalool benzoate: Prepared according to General Procedure B with 3 equiv of substrate on 0.144 mmol scale using chloroamide **6** and tetrahydrolinalool benzoate giving 56% NMR yield. GC data obtained using Method 4.

Table S31. GC data for functionalization of tetrahydrolinalool benzoate with 6.

Product	Retention Time	Peak Area
3°	11.556	7.665
2°	11.764	3.052
1°	11.995	35.915
i-Pr 1°	12.533	53.368

Figure S47. ^{1}H NMR of crude reaction of of tetrahydrolinalool benzoate with 6.

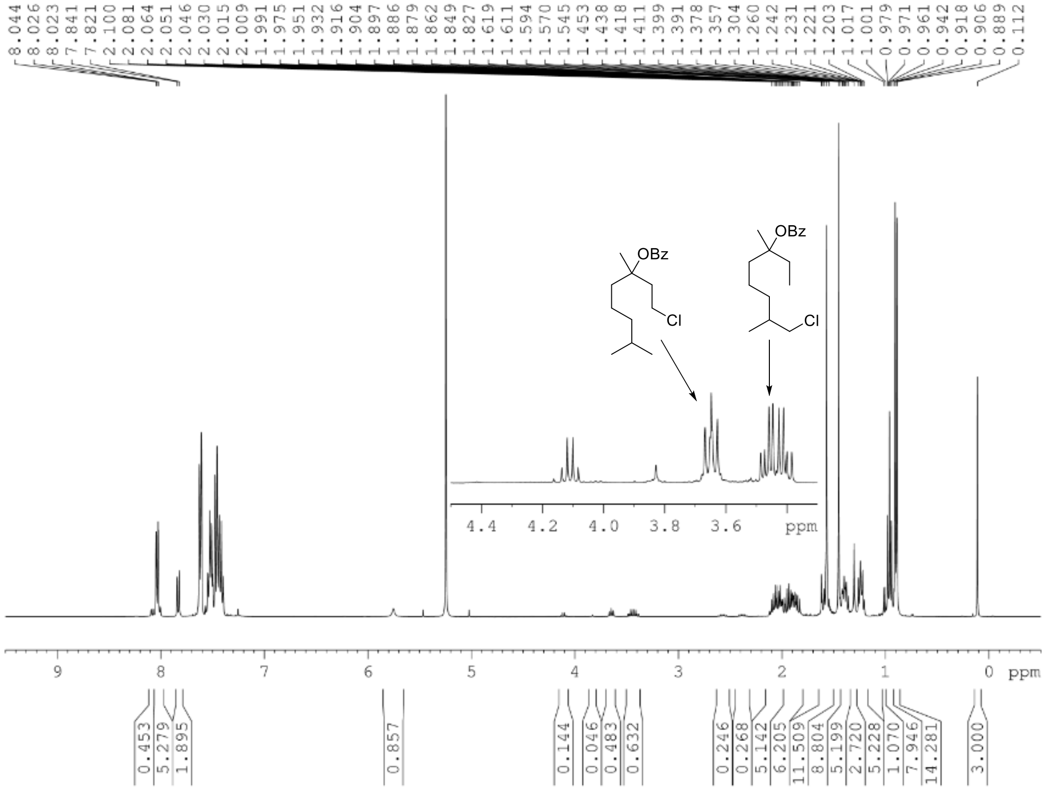

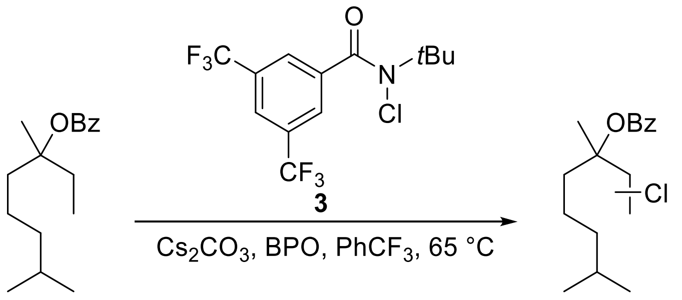

24 **24-Cl**

Reaction with tetrahydrolinalool benzoate: Prepared according to General Procedure B with 3 equiv of substrate on 0.144 mmol scale using chloroamide **3** and tetrahydrolinalool benzoate giving 37% NMR yield. GC data obtained using Method 4.

Table S32. GC data for functionalization of tetrahydrolinalool benzoate with 3.

Product	Retention Time	Peak Area
3°	11.560	29.698
2°	11.775	40.058
1°	11.980	5.337

i-Pr 1°	12.517	24.907

Figure S48. ^{1}H NMR of crude reaction of tetrahydrolinalool benzoate with 3.

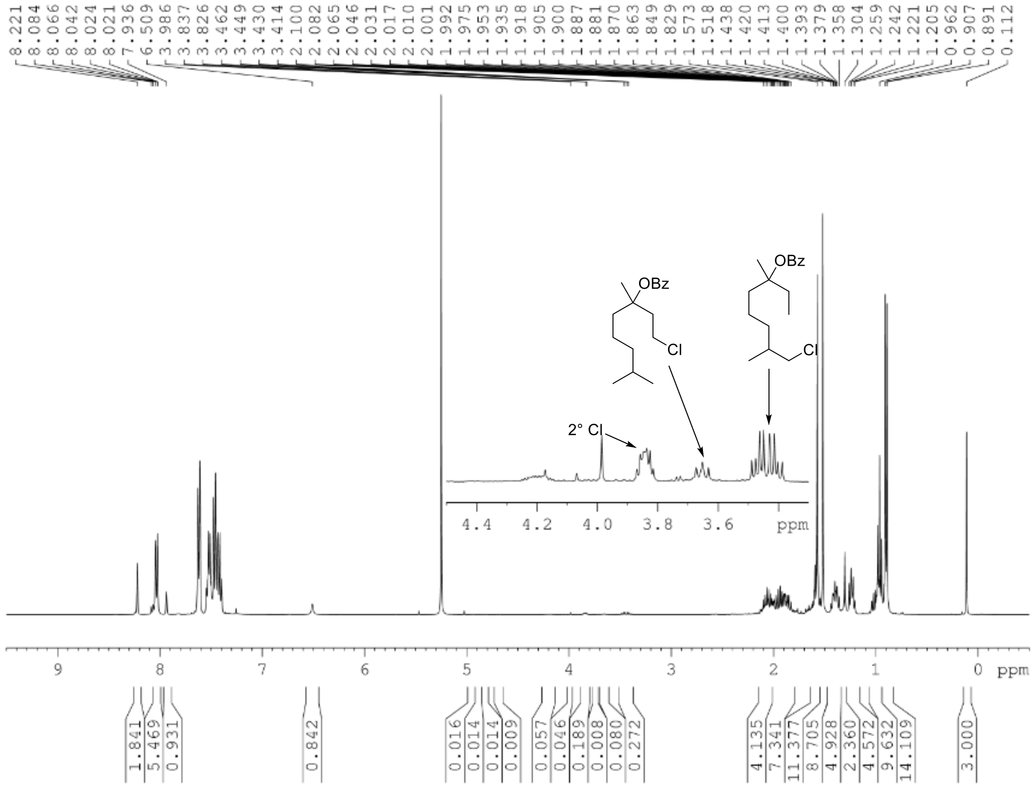

Figure S49. Chromatograph of tetrahydrolinalool benzoate functionalizations

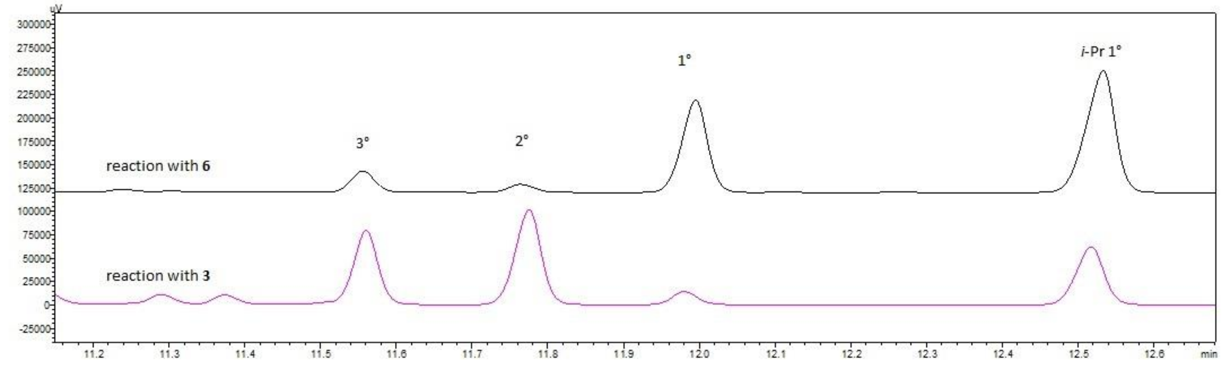

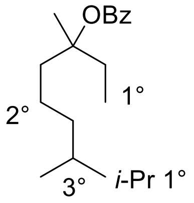

GC assignments supported by ^{1}H NMR spectrum analysis.

Product assignments for tetrahydrolinalool benzoate were determined using multiple 1D and 2D NMR experiments including ^{1}H, ^{13}C, DEPT 135, HMQC, and TOCSY. The peaks at 3.4 and 3.5 ppm in the ^{1}H NMR were determined to be diastereotopic protons because both protons correlate to a single carbon peak at 51.1 ppm in the HMQC. Using DEPT 135, it can be determined that the carbon peak at 51.1 ppm corresponds to a methylene carbon signal which is in agreement with a primary functionalization. Based on the TOCSY, those protons are in a spin system with more than two other proton signals, therefore the peaks at 3.4 and 3.5 ppm must correspond to functionalization on the *iso*-propyl methyl groups of the molecule. The peak at 3.6 ppm in the ^{1}H NMR correlates with a single carbon peak at 39.8 ppm in the HMQC. Using DEPT 135, it can be determined that the carbon peak at 39.8 ppm corresponds to a methylene carbon signal which is in agreement with a primary functionalization. Based on the TOCSY, those protons are in the spin system with only two other proton signals, therefore the peak at 3.6 ppm must correspond to functionalization on the ethyl methyl group. Secondary product tentatively assigned based on steric considerations.

FIGURE S50. ^{1}H NMR spectra of tetrahydrolinalool benzoate functionalization with 3 after isolation

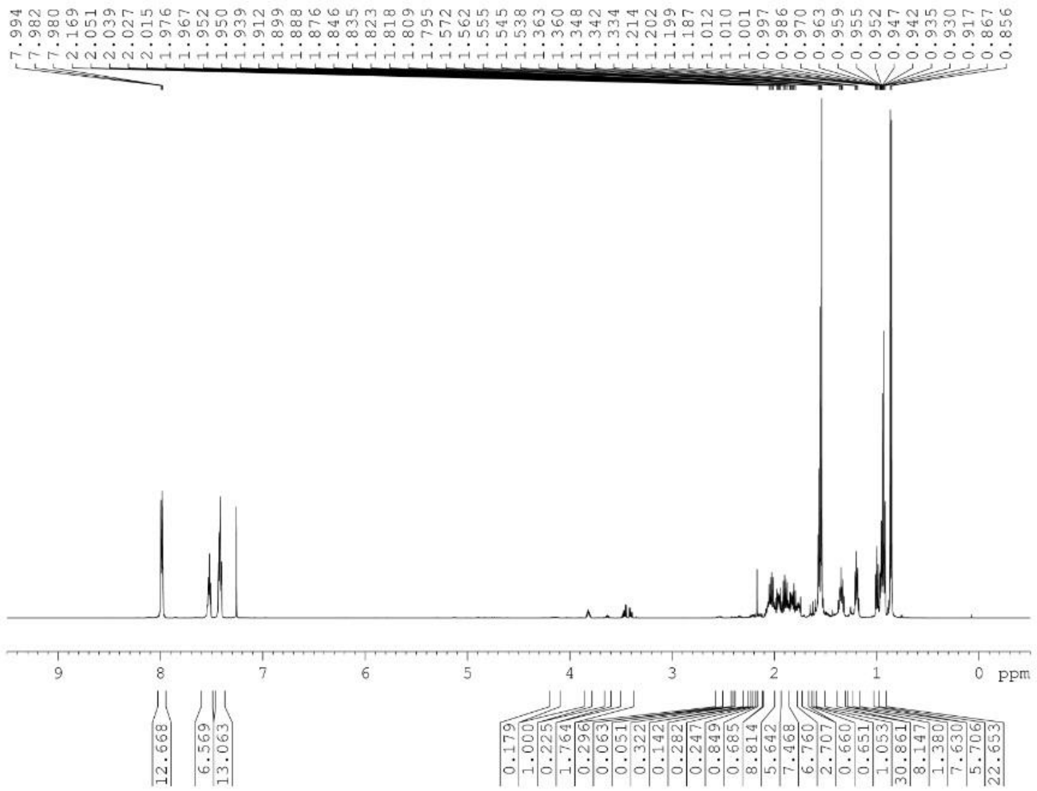

FIGURE S51. ^{13}C NMR spectra of tetrahydrolinalool benzoate functionalization with 3 after isolation

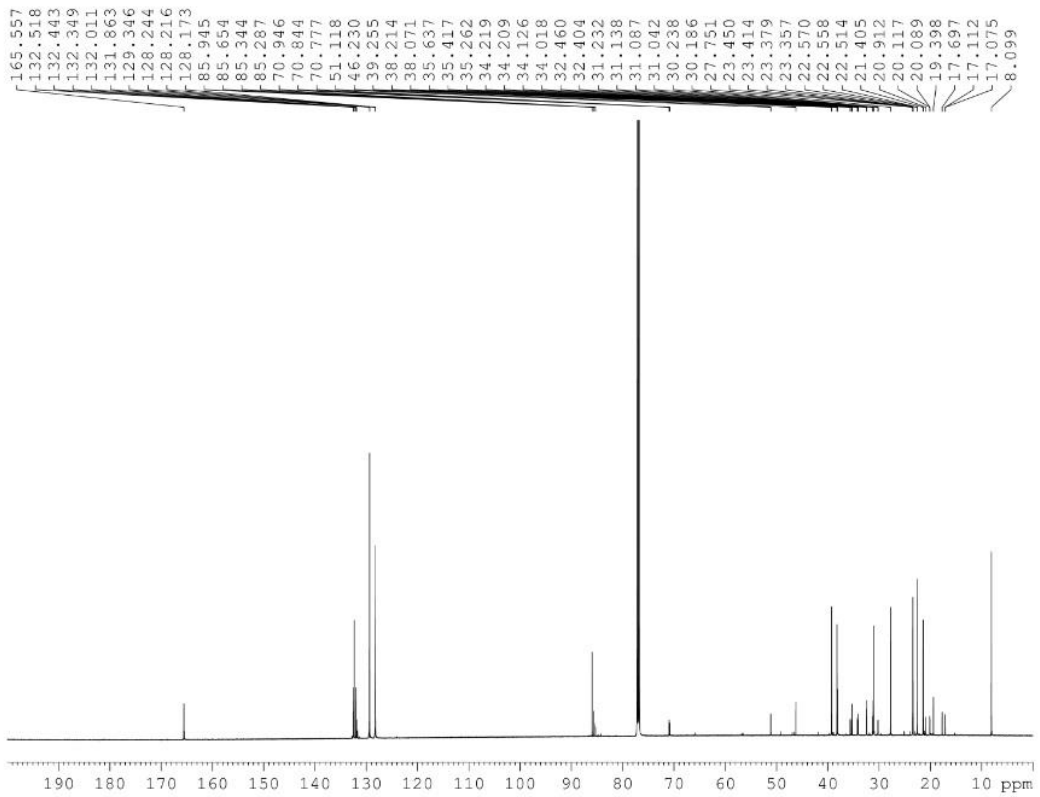

FIGURE S52. DEPT 135 spectra of tetrahydrolinalool benzoate functionalization with 3 after isolation

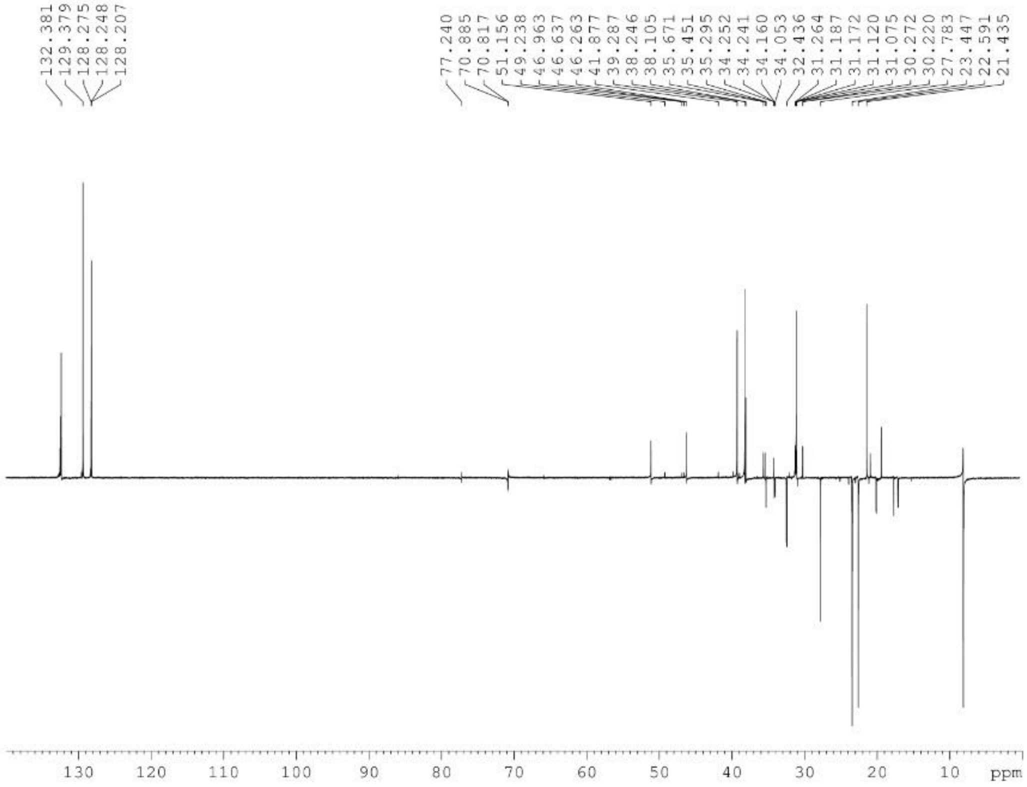

FIGURE S53. HMQC spectra of tetrahydrolinalool benzoate functionalization with 3 after isolation

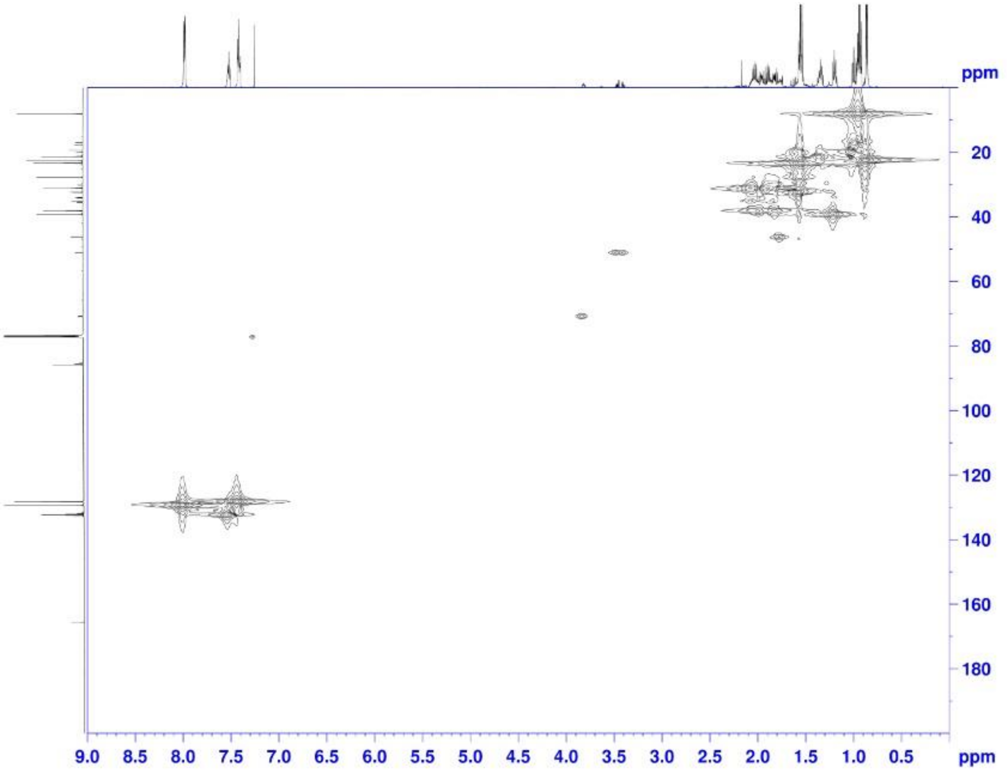

FIGURE S54. TOCSY spectra of tetrahydrolinalool benzoate functionalization with 3 after isolation

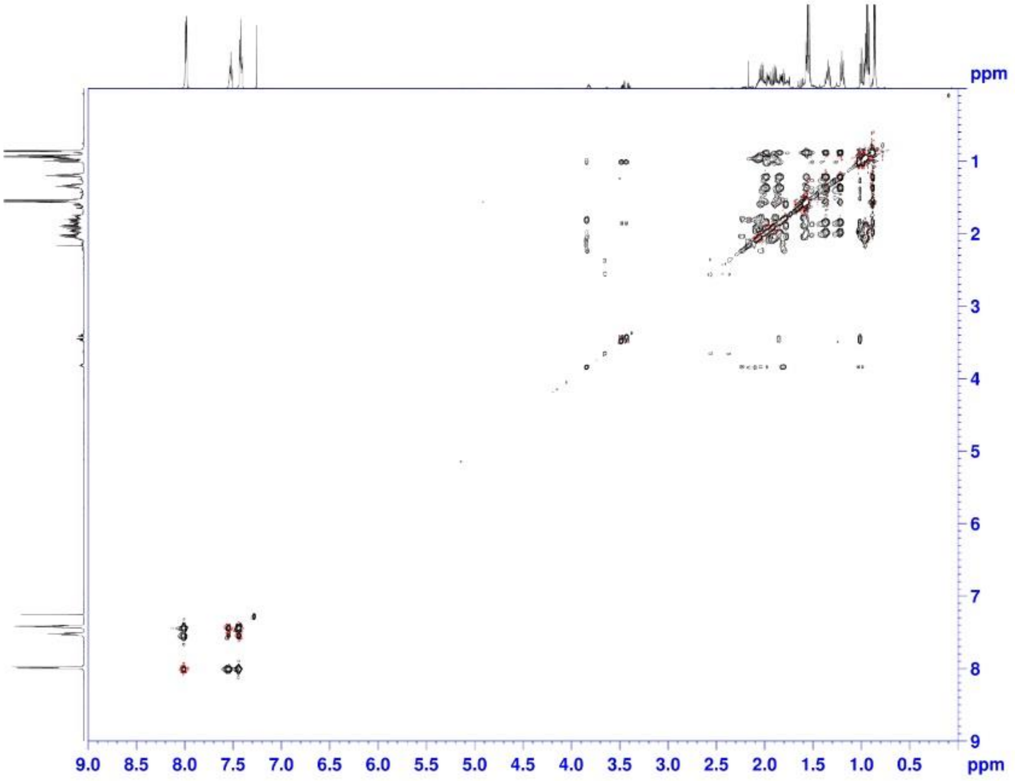

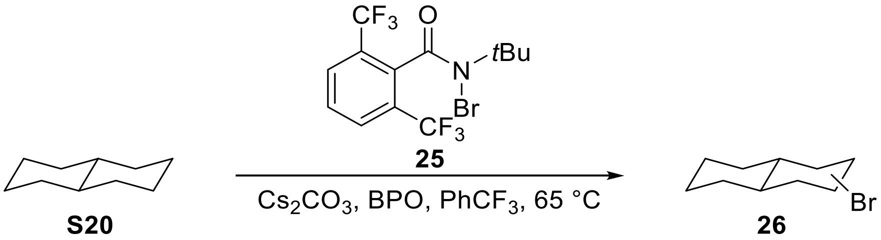

Reaction with *trans*-decalin: Prepared according to General Procedure A with 1 equiv of bromoamide on 0.144 mmol scale using bromoamide **25** and *trans*-decalin giving 49% NMR yield.

Figure S55. ^{1}H NMR of crude reaction of *trans*-decalin with 25.

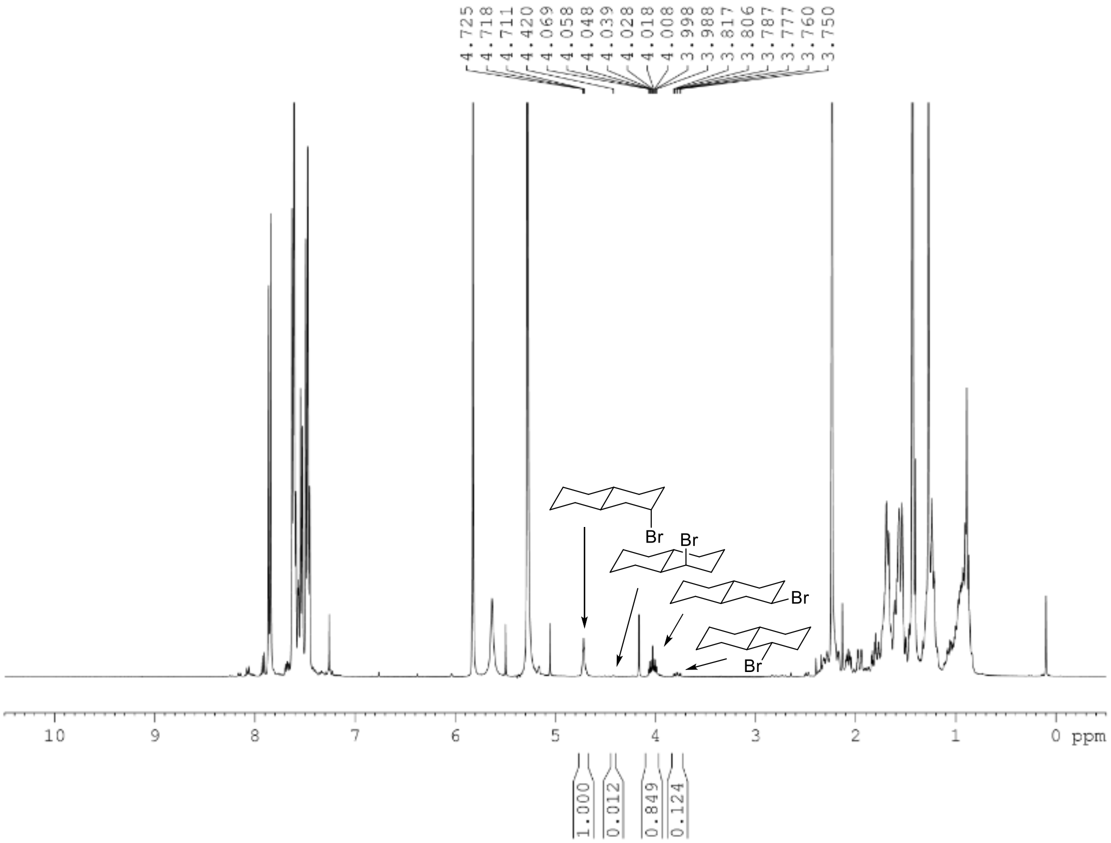

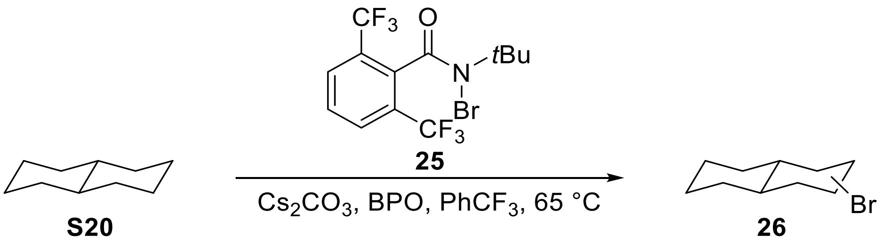

Reaction with *trans*-decalin: Prepared according to General Procedure B with 5 equiv of substrate on 0.144 mmol scale using bromoamide **26** and *trans*-decalin giving 51% NMR yield.

Figure S56. ^{1}H NMR of crude reaction of *trans*-decalin with 25.

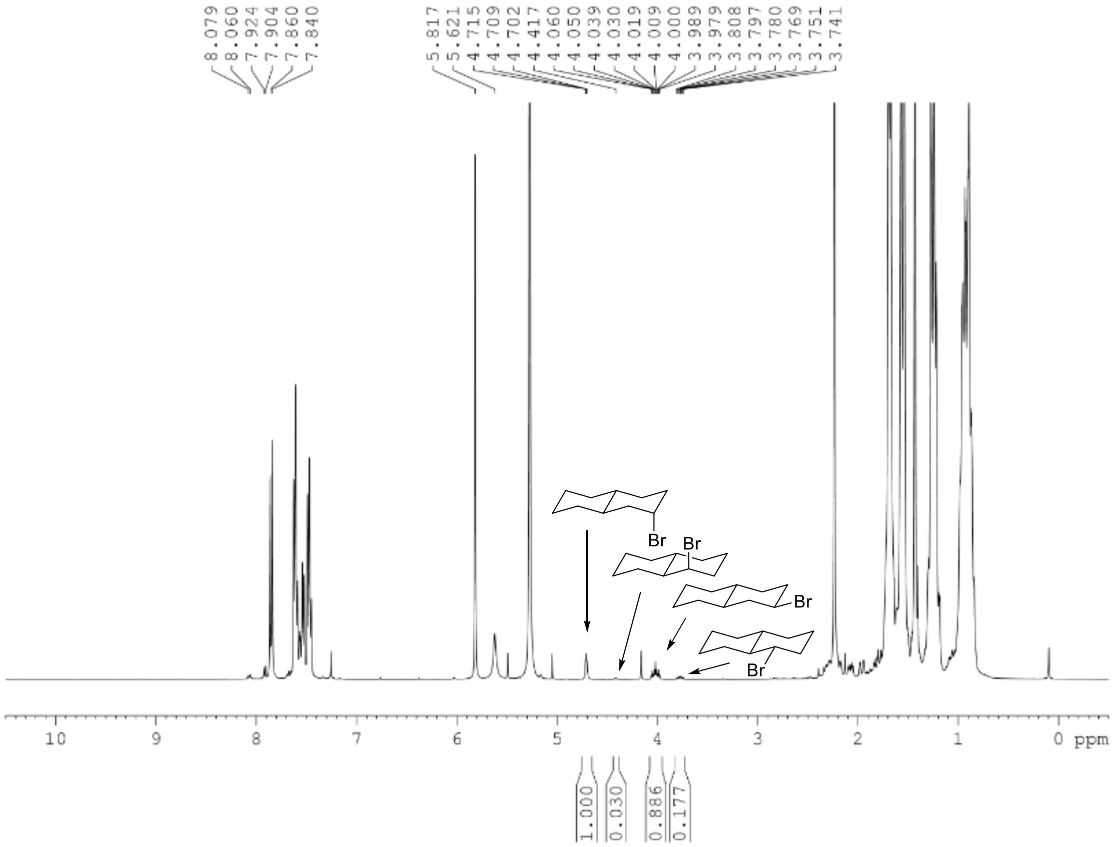

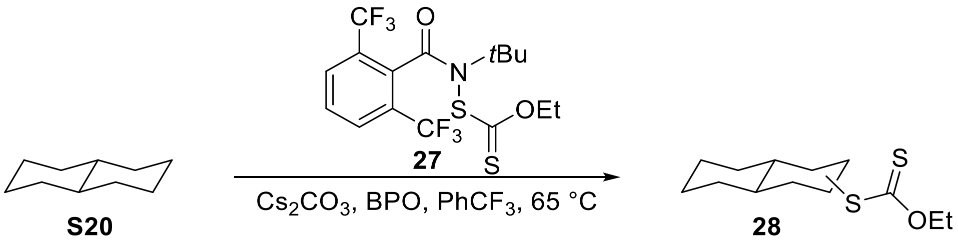

Reaction with *trans*-decalin: Prepared according to General Procedure C on 0.144 mmol scale using xanthylamide **28** and *trans*-decalin giving 76% NMR yield.

Figure S57. ^{1}H NMR of crude reaction of *trans*-decalin with 27.

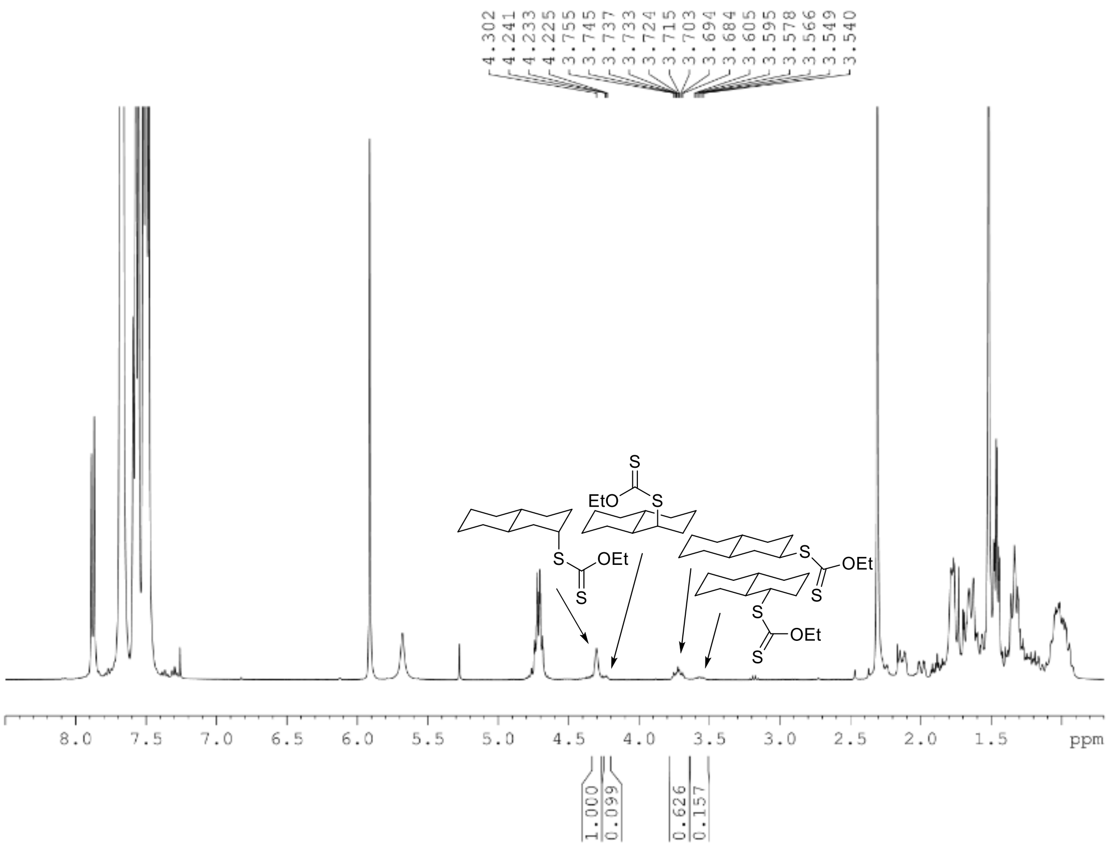

X-Ray Crystallography Data

X-ray Structure Determination: Single crystals suitable for structure analysis were selected from the bulk and mounted on a MiTeGen mounts. Data were collected on a Bruker-Nonius X8 Kappa ApexII diffractometer by ω and ϕ scans using MoKα radiation(λ = 0.71073 Å). Specimen temperature was controlled using a Oxford Cryosystems Cryostream 700. Corrections for Lorentz and polarization effects, and absorption were made using SADABS[8]. The structure was solved using direct methods, and refined using full-matrix least squares (on F^2) using the SHELX[9] software package. All non-hydrogen atoms were refined anisotropically. H atoms were added at calculated positions, with coordinates and U_{iso} values allowed to ride on the parent atom.

Figure S58. Thermal ellipsoid plot for 6, 50% probability level. Selected H atoms removed for clarity.

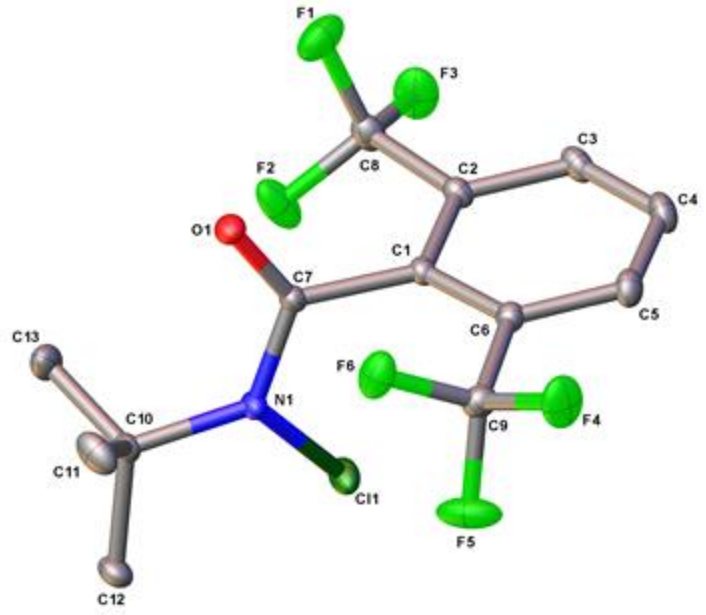

A colorless block-like specimen of $C_{13}H_{12}ClF_6NO$, approximate dimensions 0.332 mm x 0.397 mm x 0.497 mm, was used for the X-ray crystallographic analysis. The X-ray intensity data were measured.

The total exposure time was 2.73 hours. The frames were integrated with the Bruker SAINT software package using a narrow-frame algorithm. The integration of the data using a monoclinic unit cell yielded a total of 54325 reflections to a maximum θ angle of 36.45° (0.60 Å resolution), of which 6844 were independent (average redundancy 7.938, completeness = 99.8%, R_{int} = 2.44%, R_{sig} = 1.56%) and 5844 (85.39%) were greater than 2σ(F^2). The final cell constants of a = 7.6263(6) Å, b = 16.9562(13) Å, c = 10.8430(9) Å, β = 92.648(4)°, volume = 1400.64(19) $Å^3$, are based upon the refinement of the XYZ-centroids of 328 reflections above 20 σ(I) with 7.898° < 2θ < 67.20°. Data were corrected for absorption effects using the Multi-Scan method (SADABS). The ratio of minimum to maximum apparent transmission was 0.954. The calculated minimum and maximum transmission coefficients (based on crystal size) are 0.8490 and 0.8950.

The final anisotropic full-matrix least-squares refinement on F^2 with 202 variables converged at R1 = 3.12%, for the observed data and wR2 = 8.87% for all data. The goodness-of-fit was 1.028. The largest peak in the final difference electron density synthesis was 0.661 $e^-/Å^3$ and the largest hole was -0.371 $e^-/Å^3$ with an RMS deviation of 0.059 $e^-/Å^3$. On the basis of the final model, the calculated density was 1.649 g/cm^3 and F(000), 704 e^-.

Table S33. Sample and crystal data for 6.

Identification code	CCDC 1553660	
Chemical formula	$C_{13}H_{12}ClF_6NO$	
Formula weight	347.69 g/mol	
Temperature	100(2) K	
Wavelength	0.71073 Å	
Crystal size	0.332 x 0.397 x 0.497 mm	
Crystal habit	colorless block	
Crystal system	monoclinic	
Space group	$P2_1/c$	
Unit cell dimensions	a = 7.6263(6) Å	α = 90°
	b = 16.9562(13) Å	β = 92.648(4)°
	c = 10.8430(9) Å	γ = 90°
Volume	1400.64(19) $Å^3$	
Z	4	
Density (calculated)	1.649 g/cm^3	
Absorption coefficient	0.342 mm^{-1}	
F(000)	704	

Table S34. Data collection and structure refinement for 6.

Theta range for data collection	2.23 to 36.45°	
Index ranges	-12<=h<=12, -28<=k<=28, -13<=l<=18	
Reflections collected	54325	
Independent reflections	6844 [R(int) = 0.0244]	
Coverage of independent reflections	99.8%	
Absorption correction	Multi-Scan	
Max. and min. transmission	0.8950 and 0.8490	
Refinement method	Full-matrix least-squares on F^2	
Refinement program	SHELXL-2014/7 (Sheldrick, 2014)	
Function minimized	$\Sigma w(F_o^2 - F_c^2)^2$	
Data / restraints / parameters	6844 / 0 / 202	
Goodness-of-fit on F^2	1.028	
Δ/σ_{max}	0.001	
Final R indices	5844 data; I>2σ(I)	R1 = 0.0312, wR2 = 0.0840
	all data	R1 = 0.0385, wR2 = 0.0887
Weighting scheme	$w=1/[\sigma^2(F_o^2)+(0.0456P)^2+0.3763P]$ where $P=(F_o^2+2F_c^2)/3$	
Largest diff. peak and hole	0.661 and -0.371 eÅ^{-3}	
R.M.S. deviation from mean	0.059 eÅ^{-3}	

Table S35. Atomic coordinates and equivalent isotropic atomic displacement parameters (Å^2) for 6.

U(eq) is defined as one third of the trace of the orthogonalized U_{ij} tensor.

	x/a	y/b	z/c	U(eq)
Cl1	0.06334(2)	0.59161(2)	0.95250(2)	0.01827(5)

	x/a	y/b	z/c	U(eq)
F1	0.97171(9)	0.72173(3)	0.54611(6)	0.03143(13)
F2	0.97412(8)	0.72702(3)	0.74377(6)	0.03009(14)
F3	0.72916(7)	0.72001(3)	0.63829(7)	0.02915(13)
F4	0.21895(8)	0.36524(3)	0.68055(6)	0.02891(13)
F5	0.21523(8)	0.42495(4)	0.85526(6)	0.03280(14)
F6	0.36569(6)	0.47131(3)	0.70980(6)	0.02299(11)
O1	0.31277(7)	0.63976(3)	0.66938(5)	0.01546(10)
N1	0.23834(7)	0.61603(4)	0.86725(5)	0.01186(9)
C1	0.05252(8)	0.56541(4)	0.69423(6)	0.01134(10)
C2	0.90119(9)	0.60461(4)	0.64902(6)	0.01304(11)
C3	0.75549(9)	0.56257(5)	0.60255(7)	0.01657(12)
C4	0.75996(10)	0.48099(5)	0.59826(8)	0.01837(13)
C5	0.91063(10)	0.44129(4)	0.63964(7)	0.01688(12)
C6	0.05571(9)	0.48298(4)	0.68721(6)	0.01276(11)
C7	0.21230(8)	0.61148(4)	0.74185(6)	0.01134(10)
C8	0.89419(10)	0.69327(4)	0.64503(8)	0.01766(13)
C9	0.21378(10)	0.43636(4)	0.73311(7)	0.01593(12)
C10	0.38949(8)	0.66004(4)	0.93100(6)	0.01218(10)
C11	0.55951(10)	0.62246(6)	0.88981(8)	0.02160(15)
C12	0.38191(10)	0.65082(5)	0.07056(7)	0.01736(12)
C13	0.37696(12)	0.74717(5)	0.89683(8)	0.02183(15)

Table S36. Bond lengths (Å) for 6.

Cl1-N1	1.7091(6)	F1-C8	1.3384(10)

F2-C8	1.3361(10)	F3-C8	1.3366(9)
F4-C9	1.3351(9)	F5-C9	1.3381(10)
F6-C9	1.3358(9)	O1-C7	1.2207(8)
N1-C7	1.3673(9)	N1-C10	1.5132(9)
C1-C6	1.4001(10)	C1-C2	1.4006(9)
C1-C7	1.5177(9)	C2-C3	1.3946(10)
C2-C8	1.5048(11)	C3-C4	1.3847(12)
C3-H3	0.95	C4-C5	1.3882(11)
C4-H4	0.95	C5-C6	1.3918(10)
C5-H5	0.95	C6-C9	1.5068(10)
C10-C13	1.5250(11)	C10-C12	1.5251(10)
C10-C11	1.5298(10)	C11-H11A	0.98
C11-H11B	0.98	C11-H11C	0.98
C12-H12A	0.98	C12-H12B	0.98
C12-H12C	0.98	C13-H13A	0.98
C13-H13B	0.98	C13-H13C	0.98

Table S37. Bond angles (°) for 6.

C7-N1-C10	123.69(5)	C7-N1-Cl1	116.30(5)
C10-N1-Cl1	118.03(4)	C6-C1-C2	118.09(6)
C6-C1-C7	121.11(6)	C2-C1-C7	120.69(6)
C3-C2-C1	120.91(7)	C3-C2-C8	118.27(6)
C1-C2-C8	120.78(6)	C4-C3-C2	120.16(7)
C4-C3-H3	119.9	C2-C3-H3	119.9
C3-C4-C5	119.66(7)	C3-C4-H4	120.2

C5-C4-H4	120.2	C4-C5-C6	120.36(7)
C4-C5-H5	119.8	C6-C5-H5	119.8
C5-C6-C1	120.78(6)	C5-C6-C9	117.78(6)
C1-C6-C9	121.43(6)	O1-C7-N1	123.46(6)
O1-C7-C1	120.08(6)	N1-C7-C1	116.41(6)
F2-C8-F3	106.99(7)	F2-C8-F1	106.60(7)
F3-C8-F1	106.47(7)	F2-C8-C2	113.00(6)
F3-C8-C2	111.87(6)	F1-C8-C2	111.52(6)
F4-C9-F6	106.08(6)	F4-C9-F5	107.03(7)
F6-C9-F5	106.49(7)	F4-C9-C6	111.83(6)
F6-C9-C6	113.10(6)	F5-C9-C6	111.88(6)
N1-C10-C13	109.20(6)	N1-C10-C12	109.86(5)
C13-C10-C12	109.60(6)	N1-C10-C11	107.42(6)
C13-C10-C11	112.15(7)	C12-C10-C11	108.57(6)
C10-C11-H11A	109.5	C10-C11-H11B	109.5
H11A-C11-H11B	109.5	C10-C11-H11C	109.5
H11A-C11-H11C	109.5	H11B-C11-H11C	109.5
C10-C12-H12A	109.5	C10-C12-H12B	109.5
H12A-C12-H12B	109.5	C10-C12-H12C	109.5
H12A-C12-H12C	109.5	H12B-C12-H12C	109.5
C10-C13-H13A	109.5	C10-C13-H13B	109.5
H13A-C13-H13B	109.5	C10-C13-H13C	109.5
H13A-C13-H13C	109.5	H13B-C13-H13C	109.5

Table S38. Torsion angles (°) for 6.

C6-C1-C2-C3	2.32(10)	C7-C1-C2-C3	178.52(6)
C6-C1-C2-C8	-175.39(6)	C7-C1-C2-C8	0.80(10)
C1-C2-C3-C4	-1.29(11)	C8-C2-C3-C4	176.49(7)
C2-C3-C4-C5	-0.45(12)	C3-C4-C5-C6	1.08(12)
C4-C5-C6-C1	0.01(11)	C4-C5-C6-C9	178.71(7)
C2-C1-C6-C5	-1.68(10)	C7-C1-C6-C5	-177.86(6)
C2-C1-C6-C9	179.66(6)	C7-C1-C6-C9	3.48(10)
C10-N1-C7-O1	3.17(11)	Cl1-N1-C7-O1	166.78(6)
C10-N1-C7-C1	-179.43(6)	Cl1-N1-C7-C1	-15.82(8)
C6-C1-C7-O1	96.61(8)	C2-C1-C7-O1	-79.47(9)
C6-C1-C7-N1	-80.89(8)	C2-C1-C7-N1	103.03(8)
C3-C2-C8-F2	143.28(7)	C1-C2-C8-F2	-38.94(10)
C3-C2-C8-F3	22.46(10)	C1-C2-C8-F3	-159.76(7)
C3-C2-C8-F1	-96.66(8)	C1-C2-C8-F1	81.11(9)
C5-C6-C9-F4	23.78(9)	C1-C6-C9-F4	-157.53(7)
C5-C6-C9-F6	143.47(7)	C1-C6-C9-F6	-37.83(9)
C5-C6-C9-F5	-96.28(8)	C1-C6-C9-F5	82.42(9)
C7-N1-C10-C13	61.59(8)	Cl1-N1-C10-C13	-101.76(6)
C7-N1-C10-C12	-178.18(6)	Cl1-N1-C10-C12	18.47(7)
C7-N1-C10-C11	-60.26(9)	Cl1-N1-C10-C11	136.40(6)

Table S39. Anisotropic atomic displacement parameters (Å^2) for 6.

The anisotropic atomic displacement factor exponent takes the form: -2π^2[$h^2 a^{*2} U_{11}$ + ... + 2 h k $a^* b^* U_{12}$]

	U_{11}	U_{22}	U_{33}	U_{23}	U_{13}	U_{12}
Cl1	0.01438(7)	0.02799(9)	0.01277(7)	-0.00244(6)	0.00409(5)	-0.00741(6)
F1	0.0404(3)	0.0202(2)	0.0343(3)	0.0060(2)	0.0092(3)	-0.0040(2)
F2	0.0355(3)	0.0187(2)	0.0343(3)	-0.0115(2)	-0.0166(2)	0.0090(2)
F3	0.0180(2)	0.0226(2)	0.0462(4)	0.0018(2)	-0.0056(2)	0.00855(19)
F4	0.0297(3)	0.0124(2)	0.0441(4)	-0.0029(2)	-0.0035(2)	0.00419(19)
F5	0.0289(3)	0.0510(4)	0.0187(2)	0.0131(2)	0.0036(2)	0.0169(3)
F6	0.01256(19)	0.0192(2)	0.0374(3)	0.00244(19)	0.00307(19)	0.00164(16)
O1	0.0149(2)	0.0193(2)	0.0123(2)	0.00171(17)	0.00170(17)	-0.00409(17)
N1	0.0098(2)	0.0158(2)	0.0100(2)	-0.00017(18)	0.00111(17)	-0.00287(17)
C1	0.0103(2)	0.0130(2)	0.0108(2)	-0.00089(19)	-0.00009(19)	-0.00098(19)
C2	0.0113(2)	0.0152(3)	0.0125(3)	-0.0016(2)	-0.00074(19)	0.0009(2)
C3	0.0116(3)	0.0229(3)	0.0150(3)	-0.0035(2)	-0.0014(2)	-0.0003(2)
C4	0.0137(3)	0.0226(3)	0.0187(3)	-0.0057(2)	-0.0007(2)	-0.0047(2)
C5	0.0163(3)	0.0159(3)	0.0185(3)	-0.0036(2)	0.0019(2)	-0.0046(2)
C6	0.0120(2)	0.0131(2)	0.0132(3)	-0.0004(2)	0.0014(2)	-0.00087(19)
C7	0.0107(2)	0.0123(2)	0.0109(2)	-0.00007(19)	-0.00061(18)	-0.00033(19)
C8	0.0151(3)	0.0168(3)	0.0207(3)	-0.0013(2)	-0.0033(2)	0.0029(2)
C9	0.0158(3)	0.0151(3)	0.0171(3)	0.0016(2)	0.0019(2)	0.0012(2)
C10	0.0105(2)	0.0142(2)	0.0117(2)	-0.0008(2)	-0.00099(19)	-0.00145(19)
C11	0.0111(3)	0.0341(4)	0.0194(3)	-0.0063(3)	-0.0006(2)	0.0024(3)
C12	0.0171(3)	0.0234(3)	0.0113(3)	-0.0010(2)	-0.0017(2)	-0.0021(2)
C13	0.0275(4)	0.0147(3)	0.0226(4)	0.0022(3)	-0.0063(3)	-0.0054(3)

Table S40. Hydrogen atomic coordinates and isotropic atomic displacement parameters (Å^2) for 6.

	x/a	y/b	z/c	U(eq)
H3	-0.3471	0.5900	0.5738	0.02
H4	-0.3396	0.4523	0.5671	0.022
H5	-0.0853	0.3854	0.6355	0.02
H11A	0.5638	0.5671	0.9156	0.032
H11B	0.6604	0.6507	0.9278	0.032
H11C	0.5633	0.6256	0.7997	0.032
H12A	0.2768	0.6772	1.0989	0.026
H12B	0.4866	0.6747	1.1109	0.026
H12C	0.3775	0.5947	1.0916	0.026
H13A	0.3851	0.7531	0.8074	0.033
H13B	0.4732	0.7761	0.9393	0.033
H13C	0.2644	0.7683	0.9219	0.033

Computational Data

Methods. All the calculations were carried out using the Gaussian16 program package at the CINECA (Italy) Supercomputer center.[10] In our investigation, all of the stationary points have been initially optimized having recourse to density functional theory (DFT), *viz.* adopting the ωB97XD functional with an unrestricted (U) formalism when systems containing an unpaired number of electrons were considered, and the standard 6-31G(d,p) basis set in the gas phase. To confirm the nature of stationary points, vibrational frequencies have been calculated for all of the optimized structures at the same level of theory as geometry optimizations, and it was verified that local minima had only real frequencies, while transition states (TS) were identified by the presence of a single imaginary frequency corresponding to the expected motion along the reaction coordinate.
For each substrate, reagent, intermediate (amidyl radicals) or reacting situation, a systematic investigation on all of the possible conformations has been carried out. However, only the most stable conformation has been reported and has been considered for further work. For the particular case of *trans* decalin, the activation of both axial and equatorial C-H positions has been considered, but also in this case only the most stable TS has been further considered.
The solvent effect was included by single-point calculations on the optimized geometries obtained in the gas phase at the (U)ωB97XD/6-31G(d,p) level of theory in dichloromethane bulk, by maintaining the default solvent options. The SMD model has been used, since this is the recommended choice for determining solvation energies.
Spin densities plots have been determined via the "cubegen" command.

The work has been devoted to clarify two different aspects. The first one is related to the properties of amidyl radicals **3'** and **6'**, arising from **3** and **6**, respectively. The second aspect is their capability to promote H-abstraction processes from the different positions of the chosen substrates. The following strategy For each reacting situation, the most stable TS describing the hydrogen transfer event has been located and fully analyzed. The detailed characterization of TSs has been performed via the IRC method at the same level of theory as the optimizations (UωB97XD/6-31G(d,p)) by describing (up to) 70 points in each direction (70 points in the forward and 70 points in the reverse direction). Indeed, to have a more reliable estimate of the energy barriers and changes involved in the processes described by TSs, the first and last points of the IRC have been fully re-optimized at the UωB97XD/6-31G(d,p) level of theory, then solvent effect was included as described above. The thermodynamic parameters reported in the text (see Table 2), *viz.* ΔG and $\Delta G^{\ddagger}$ values, have thus been determined according to the following equations:

$$\Delta G = G \text{ (optimized IRC end-point)} - G \text{ (optimized IRC start-point)}$$
$$\Delta G^{\ddagger} = G \text{ (TS)} - G \text{ (optimized IRC start-point)}$$

Where the required Gibbs free energy (G) values were calculated via:

G = Electronic energy at the SMD(CH_2Cl_2)-UωB97XD/6-31G(d,p) level of theory + thermal correction to Gibbs Free Energy at the UωB97XD/6-31G(d,p) level of theory

We also speculated whether the observed energy barriers and changes could be affected by the preliminary formation of complexes between amidyl radicals and substrates or of complexes between the resulting amides and alkyl radicals. This was not the case, however, since all the analyzed reacting situations did not evidence the formation of stabilized complexes (data not reported). Accordingly, we can safely assume that the reported IRC start-point, TS and end-point offer a realistic scenario.

Table S41. Calculated parameters for the TSs describing the processes reported in Table 2 in main text.

	Gibbs Free Energy (G) at the SMD(CH_2Cl_2)-UωB97XD/6-31G(d,p) level of theory, [Hartree]			ΔG‡ [kcal·mol^{-1}] (see Table 2)	ΔG [kcal·mol^{-1}] (see Table 2)
	IRC start-point	TS	IRC end-point		
***trans* decalin**[a]					
**3' – distal position (AX)**	-1622.731866	-1622.713051	-1622.745352	**11.81**	**-8.46**
**3' – proximal position (EQ)**	-1622.732383	-1622.717933	-1622.747068	**9.07**	**-9.21**
**6' – distal position (AX)**	-1622.716965	-1622.701724	-1622.738474	**9.56**	**-13.50**
**6' – proximal position (EQ)**	-1622.718245	-1622.703230	-1622.737301	**9.42**	**-11.96**
**7 (3-methylpentane)**					
**3' – primary position**	-1467.979542	-1467.958001	-1467.988851	**13.52**	**-5.84**
**3' – secondary position**	-1467.979603	-1467.963601	-1467.996299	**10.04**	**-10.48**
**6' – primary position**	-1467.965036	-1467.951132	-1467.981038	**8.72**	**-10.04**
**6' – secondary position**	-1467.964620	-1467.948442	-1467.986756	**10.15**	**-13.89**
**3-pentyl acetate**					
**3' – primary position**	-1656.484293	-1656.464444	-1656.494305	**12.46**	**-6.28**
**3' – secondary position**	-1656.485481	-1656.467088	-1656.498583	**11.54**	**-8.22**
**6' – primary position**	-1656.472800	-1656.456262	-1656.488058	**10.38**	**-9.57**
**6' – secondary position**	-1656.472584	-1656.454679	-1656.492162	**11.24**	**-12.29**

[a] For each reacting situation, it has been indicated whether the most stable transition state followed an axial (AX) or an equatorial (EQ) trajectory.

Table S42. Terms adopted to calculate the Gibbs Free Energy (G) values reported in Table S41.

	IRC start-point			TS			IRC end-point		
	Electronic Energy, vacuo	Thermal correction to Gibbs Free Energy	Electronic Energy, solvent (SMD)	Electronic Energy, vacuo	Thermal correction to Gibbs Free Energy	Electronic Energy, solvent (SMD)	Electronic Energy, vacuo	Thermal correction to Gibbs Free Energy	Electronic Energy, solvent (SMD)
***trans* decalin**									
**3' – distal position (AX)**	-1623.156804	0.443080	-1623.174946	-1623.136367	0.442537	-1623.155588	-1623.168931	0.444212	-1623.189564
**3' – proximal position (EQ)**	-1623.15776	0.443956	-1623.176339	-1623.140187	0.441619	-1623.159552	-1623.169699	0.442927	-1623.189995
**6' – distal position (AX)**	-1623.143282	0.446199	-1623.163164	-1623.125341	0.444509	-1623.146233	-1623.160891	0.444401	-1623.182875
**6' – proximal position (EQ)**	-1623.143817	0.445883	-1623.164128	-1623.125369	0.443616	-1623.146846	-1623.161337	0.446095	-1623.183396
**7 (3-methylpentane)**									
**3' – primary position**	-1468.330618	0.366667	-1468.346209	-1468.308053	0.366327	-1468.324328	-1468.339369	0.367905	-1468.356756
**3' – secondary position**	-1468.331553	0.367063	-1468.346666	-1468.314672	0.366867	-1468.330468	-1468.345177	0.365988	-1468.362287
**6' – primary position**	-1468.316427	0.368418	-1468.333454	-1468.298866	0.366244	-1468.317376	-1468.329877	0.368157	-1468.349195
**6' – secondary position**	-1468.319293	0.371605	-1468.336225	-1468.300925	0.370287	-1468.318729	-1468.338373	0.370179	-1468.356935
**3-pentyl acetate**									
**3' – primary position**	-1656.841157	0.376989	-1656.861282	-1656.81976	0.376332	-1656.840776	-1656.846903	0.375203	-1656.869508
**3' – secondary position**	-1656.846039	0.379068	-1656.864549	-1656.826494	0.378530	-1656.845618	-1656.857405	0.379341	-1656.877924
**6' – primary position**	-1656.832678	0.380425	-1656.853225	-1656.810892	0.377450	-1656.833712	-1656.845413	0.380421	-1656.868479
**6' – secondary position**	-1656.834164	0.381144	-1656.853728	-1656.812313	0.378466	-1656.833145	-1656.850248	0.379685	-1656.871847

Amidyl radical 3'

C	-2.17204741	0.41760276	0.09220282
C	-1.12909298	1.32595625	-0.04739944
C	0.17185288	0.87881366	-0.25196987
C	0.42926540	-0.48868045	-0.31375714
C	-0.61222373	-1.40416619	-0.17576940
C	-1.90769631	-0.94768891	0.02630754
C	1.81800812	-1.01756875	-0.48857299
O	2.04525330	-2.20943105	-0.64589740
C	4.04927725	-0.20211072	0.18923961
C	4.62443259	1.21068788	0.35339782
H	3.96644453	1.82803084	0.97207963
H	5.60323754	1.16148712	0.83912065
N	2.80643930	-0.05814384	-0.55225259
H	-3.18241620	0.76960299	0.26314191
H	4.73754580	1.69404371	-0.62005449
C	3.86411497	-0.87324565	1.55764339
H	3.16681752	-0.29932803	2.17640352
H	4.82303555	-0.91533479	2.08139555
H	3.48587443	-1.89180134	1.45057600
C	4.98779965	-1.04681680	-0.70506842
H	5.96808948	-1.10574701	-0.22199254
H	5.10623321	-0.57949504	-1.68558260
H	4.58397127	-2.05149005	-0.83584983
H	0.98633714	1.58557441	-0.36695004
H	-0.38781390	-2.46402415	-0.22106699
C	-1.41315698	2.80554652	-0.03296454
C	-3.04441936	-1.93244978	0.12555897
F	-0.39015359	3.49764483	0.49359809
F	-2.51152319	3.09348983	0.68209369
F	-1.61136618	3.27579251	-1.27512249
F	-4.04405961	-1.45709621	0.88560573
F	-2.64505134	-3.09683031	0.65730594
F	-3.56019523	-2.20237201	-1.08474357

Amidyl radical 6'

C	-0.93661300	-3.03073000	-1.08559400
C	0.37576100	-2.79906200	-0.70819800
C	0.72757800	-1.61945000	-0.05511700
C	-0.24483600	-0.64831100	0.23321700
C	-1.57500000	-0.90395100	-0.14317300
C	-1.90923700	-2.08476600	-0.79939300
H	1.13849300	-3.54050200	-0.90904800
H	-2.93764600	-2.25760900	-1.08931600
C	0.09380100	0.65179700	0.94540900
O	-0.47883400	1.00388400	1.95633800
C	1.04639300	2.44784700	-0.50557400
C	0.14037500	3.53656900	0.09622300
H	-0.87446700	3.17097600	0.25579500
H	0.10598000	4.38645400	-0.59171300
N	1.16382300	1.33212800	0.41708200
C	-2.70623900	0.06607800	0.14709000
C	2.18912500	-1.45890300	0.32513900
H	-1.20330700	-3.94909200	-1.59613400

F	-2.38169700	1.33436500	-0.17457100
F	-3.79438300	-0.23723000	-0.58501000
F	-3.07860400	0.04696500	1.42563600
F	2.81352700	-2.65176200	0.32519300
F	2.34474800	-0.94184800	1.54657800
F	2.84959000	-0.68626100	-0.55339500
H	0.53735700	3.87831400	1.05482800
C	0.46013600	1.92373900	-1.83299500
H	-0.57475300	1.60049500	-1.70772600
H	0.48469600	2.72602200	-2.57577900
H	1.05409800	1.08562200	-2.20773700
C	2.46115700	2.98687100	-0.73353100
H	2.44349300	3.81862000	-1.44407800
H	2.88943900	3.34160500	0.20741600
H	3.10749600	2.19871200	-1.12768200

Reaction between 3' and *trans* decalin – distal position

TS

C	2.02024326	-0.93789473	1.05195247
C	1.02090013	-0.29715253	1.77787531
C	-0.31699395	-0.48171170	1.45038128
C	-0.66278089	-1.32948107	0.39992643
C	0.33038624	-1.99918626	-0.30738840
C	1.66826332	-1.78122110	0.00437961
C	-2.09883797	-1.56047326	0.02718741
O	-2.45242092	-2.66261347	-0.37795834
C	-4.37051118	-0.57543131	0.02835305
C	-4.92990521	-1.62047914	1.01470838
H	-4.55271912	-2.61680526	0.78344765
H	-6.02184230	-1.63012022	0.94388134
N	-2.92016409	-0.48720716	0.27504810
H	-2.37819708	0.59764675	-0.40188422
C	0.41510013	1.32734764	-1.56388440
C	1.51562126	1.01900439	-2.58237434
H	1.43383933	-0.02514077	-2.90555808
H	1.36355325	1.64201519	-3.47597759
C	-0.97393794	1.06800439	-2.15284743
H	-1.06215493	1.63713417	-3.09243265
H	-1.07640088	0.01246123	-2.43574916
C	0.53245304	2.75324790	-1.00463275
H	0.37458303	3.45915372	-1.83804501
H	3.06353732	-0.76598160	1.28720048
H	-4.65286924	-1.36224183	2.04070840
C	-4.73415913	-0.96435275	-1.41205697
H	-4.33635012	-0.24128987	-2.13000220
H	-4.33957005	-1.95076287	-1.65599770
H	-5.82259119	-0.98094284	-1.52514201
C	-4.97411731	0.78906985	0.38502668
H	-6.06582339	0.72844379	0.36302265
H	-4.66232834	1.09394617	1.38800667
H	-4.67303930	1.56700073	-0.32115157
C	-0.55679309	2.99703415	0.04343822
H	-0.40334708	2.30949434	0.88379448
H	-0.46392915	4.00948534	0.45275198

C	-2.13395308	1.48782860	-1.26737852
H	-3.07483412	1.46102440	-1.82340559
C	2.91067633	1.28249864	-2.01311832
H	3.11167935	0.56906782	-1.20426206
H	3.67053443	1.10286845	-2.78082130
C	1.93391011	2.99564614	-0.43649771
H	2.08072212	2.34757635	0.43816653
H	2.01241305	4.02771932	-0.07490497
C	3.02709324	2.70721990	-1.46723667
H	2.93680523	3.42052972	-2.29796893
H	4.01576628	2.86474108	-1.02331864
C	-1.95747016	2.80837290	-0.53737182
H	-2.72133625	2.91541009	0.24016418
H	-2.14771219	3.61080975	-1.26683510
H	-1.09977505	0.03577044	1.99213715
H	0.04506729	-2.66660555	-1.11342629
C	1.39828207	0.57760187	2.94267817
C	2.73081646	-2.47921031	-0.80239121
F	1.53668207	-0.13517586	4.07146045
F	2.56727513	1.20703092	2.72816603
F	0.46997694	1.51960196	3.17949689
F	2.81273852	-3.78121031	-0.48954884
F	2.46657949	-2.40691968	-2.11897635
F	3.94463351	-1.94035916	-0.60462930
H	0.56015014	0.65454583	-0.71415915

IRC start-point

C	1.70308649	-1.12757979	1.16201274
C	0.69670005	-0.42844857	1.82272915
C	-0.62946985	-0.55834125	1.42992575
C	-0.95217210	-1.38818475	0.35617498
C	0.05067870	-2.08349566	-0.31265820
C	1.37373264	-1.95143086	0.09258789
C	-2.36322448	-1.57227335	-0.10765678
O	-2.67217921	-2.49066655	-0.85999684
C	-4.50894347	-0.34794402	-0.21061947
C	-5.52455349	-1.47422405	0.10917944
H	-5.21013421	-2.41007100	-0.35491645
H	-6.50097468	-1.18147613	-0.28877090
N	-3.28411901	-0.72949907	0.46851528
H	-1.74982652	1.59787953	-0.04536673
C	0.72281378	1.45600518	-1.44584911
C	1.62633603	0.79979062	-2.49343380
H	1.23590372	-0.19265826	-2.74763342
H	1.59462187	1.39786684	-3.41579361
C	-0.71902957	1.60052521	-1.94164856
H	-0.71888351	2.19973888	-2.86357413
H	-1.11561732	0.61295668	-2.21271574
C	1.28797499	2.81079682	-0.98471184
H	1.29468238	3.48061147	-1.86153123
H	2.73683528	-1.01533018	1.46559187
H	-5.61407149	-1.61899200	1.18848044
C	-4.37242153	-0.17343522	-1.72953609
H	-3.61116705	0.57488584	-1.96543474
H	-4.10418974	-1.11256593	-2.21405933

H	-5.32457116	0.17322030	-2.14103011
C	-4.97834584	0.95795685	0.44673217
H	-5.95062785	1.24993758	0.03968779
H	-5.07246888	0.83073537	1.52808160
H	-4.26796143	1.76602101	0.25393244
C	0.38453339	3.43835311	0.07997207
H	0.38635689	2.79938501	0.97359926
H	0.79218676	4.40797025	0.38947282
C	-1.62533723	2.27047013	-0.90606322
H	-2.62456027	2.43025218	-1.32716915
C	3.07594703	0.68397203	-2.01762512
H	3.12712459	-0.01145438	-1.17209542
H	3.69971572	0.25501015	-2.80876992
C	2.73149959	2.66197340	-0.49715608
H	2.73753130	2.02998069	0.40217200
H	3.12218050	3.64041573	-0.19320107
C	3.62809785	2.03714022	-1.56712191
H	3.68377270	2.71403277	-2.43064718
H	4.65001978	1.92331831	-1.18951706
C	-1.04951397	3.60325547	-0.42338987
H	-1.68504630	4.02673567	0.36200666
H	-1.05765857	4.31915451	-1.25653989
H	-1.41612385	-0.02176579	1.94712395
H	-0.22084277	-2.72451054	-1.14456862
C	1.05290424	0.44301094	2.99739800
C	2.43456311	-2.73989810	-0.63040602
F	1.10148913	-0.26224196	4.13852855
F	2.25728916	1.01733247	2.83666826
F	0.15536445	1.42630639	3.17566521
F	2.32320060	-4.05254227	-0.37392767
F	2.33090761	-2.59002205	-1.96160366
F	3.67237581	-2.36342441	-0.27395130
H	0.70928179	0.80390152	-0.56187762

IRC end-point

C	1.82271348	-1.09193435	1.09754218
C	0.84464757	-0.40239322	1.80691670
C	-0.49519466	-0.51224484	1.45239746
C	-0.86657145	-1.31871748	0.37743518
C	0.10741968	-2.02339160	-0.32253762
C	1.44656832	-1.89822802	0.03023637
C	-2.30163277	-1.50279278	-0.04826512
O	-2.65596433	-2.55155820	-0.56953726
C	-4.54787714	-0.39508244	-0.13093053
C	-5.28510258	-1.55548588	0.54794056
H	-4.92358840	-2.51487218	0.17728567
H	-6.35698687	-1.47752920	0.34277303
N	-3.11538174	-0.44739792	0.21580627
H	-2.65309496	0.45071173	0.30818059
C	0.64532867	1.43176851	-1.48785409
C	1.61640511	0.87930483	-2.53437849
H	1.30386715	-0.12886273	-2.82942500
H	1.56185385	1.50535401	-3.43700621
C	-0.79147702	1.47500767	-2.02540161
H	-0.77487199	1.98256737	-3.00884065

H	-1.14909375	0.45704770	-2.23056646
C	1.08960283	2.80760578	-0.96478244
H	1.04907875	3.51832258	-1.80800992
H	2.86744991	-0.99108398	1.36499621
H	-5.13738358	-1.51764596	1.63127328
C	-4.72949492	-0.45583476	-1.65378111
H	-4.21195934	0.37777089	-2.13957119
H	-4.32656657	-1.39267430	-2.04303853
H	-5.79130571	-0.39565683	-1.91144848
C	-5.07736819	0.93253442	0.42019693
H	-6.14514071	1.02468936	0.20592993
H	-4.93898483	0.98539491	1.50451105
H	-4.57078949	1.78991531	-0.03561812
C	0.12918570	3.29762268	0.12197465
H	0.15686311	2.59407035	0.96352941
H	0.46479737	4.26504607	0.51242433
C	-1.75028817	2.19505693	-1.13407689
H	-2.80981227	2.10091640	-1.36619555
C	3.05924674	0.85337103	-2.02778658
H	3.14457227	0.13375593	-1.20522490
H	3.72922191	0.49997547	-2.81842405
C	2.53406284	2.75407112	-0.45814910
H	2.57481424	2.09855907	0.42290731
H	2.84331833	3.75002726	-0.11956531
C	3.49590042	2.23063431	-1.52596956
H	3.51699327	2.93463037	-2.36915203
H	4.51532787	2.18500925	-1.12817270
C	-1.30654424	3.41799929	-0.39913414
H	-2.00005631	3.65264834	0.41719787
H	-1.35614876	4.28259450	-1.08656969
H	-1.24729418	0.02242661	2.02148970
H	-0.20137703	-2.66458656	-1.14110741
C	1.24498289	0.43013565	2.99485374
C	2.47807634	-2.67015511	-0.75036137
F	1.34345930	-0.31233400	4.10846161
F	2.43902532	1.01752245	2.80571831
F	0.35079355	1.40256062	3.24487447
F	2.37350253	-3.98874187	-0.52739344
F	2.32893495	-2.48260145	-2.07318813
F	3.72920339	-2.30420872	-0.42937994
H	0.66187544	0.75284572	-0.62725468

Reaction between 3' and *trans* decalin – proximal position

TS

C	-2.37828313	0.87505942	-0.87560355
C	-1.33712786	1.78583600	-0.75806182
C	-0.03919851	1.42239904	-1.10793289
C	0.21722843	0.14011313	-1.58012450
C	-0.83173232	-0.76286425	-1.74621952
C	-2.11872171	-0.39900879	-1.37655572
C	1.61371829	-0.32861379	-1.87622957
O	1.81492417	-1.14558512	-2.76848714
C	3.98117516	-0.15524456	-1.15362204
C	4.51072720	0.14490705	-2.56964307

H	4.02011017	-0.48567671	-3.31056884
H	5.58842296	-0.04375917	-2.59396337
N	2.57326331	0.26861018	-1.09934653
H	2.14259276	0.20106066	0.22206407
H	-3.38595100	1.15658190	-0.59270618
H	4.33862739	1.19449226	-2.82460550
C	4.15968962	-1.64096636	-0.81471171
H	3.79239435	-1.85355762	0.19434363
H	3.61526741	-2.26509387	-1.52530513
H	5.21988194	-1.90955184	-0.85217623
C	4.75026809	0.71903961	-0.15535723
H	5.82232433	0.51697106	-0.23237089
H	4.57882338	1.77864732	-0.36458755
H	4.44740632	0.51793890	0.87572871
C	1.81990708	0.06459146	1.42786407
C	0.58370048	-0.80722666	1.52945924
C	0.06998606	-0.83089983	2.98912483
C	-0.13489794	0.59229231	3.51299352
C	1.14591222	1.42250627	3.41899837
C	1.65509494	1.47015526	1.96974232
H	-0.21937743	-0.34547769	0.93962304
H	2.69417757	-0.44290956	1.85555341
H	-0.92440154	1.08025519	2.92505056
H	-0.48832460	0.55589538	4.54993149
H	0.97140091	2.44161923	3.77902001
H	1.91682364	0.98190161	4.06473419
H	0.91849247	2.01340343	1.36950852
H	2.59928382	2.01992918	1.90390635
H	0.84335587	-1.31576862	3.60737249
C	-1.20578575	-1.67476735	3.07236432
C	0.81676801	-2.22919085	1.01063610
C	-0.44386775	-3.08731199	1.12374880
C	-0.98502669	-3.09772669	2.55508728
H	-1.56621546	-1.69606313	4.10751874
H	-1.92023231	-3.66523640	2.60059040
H	-1.98974648	-1.18962857	2.47319033
H	-0.26942930	-3.61249564	3.21059394
H	1.62711783	-2.68830847	1.59560236
H	1.16515261	-2.20422057	-0.02809612
H	-1.21613293	-2.68452287	0.45779756
H	-0.23627643	-4.10774565	0.78551112
H	0.78061123	2.12370350	-1.00419833
H	-0.61884186	-1.75079848	-2.13826878
C	-3.24719140	-1.39314769	-1.43823461
C	-1.59172728	3.14874878	-0.17342408
F	-3.54291439	-1.85662417	-0.20621847
F	-2.94915125	-2.45229487	-2.20041397
F	-4.36933368	-0.83861108	-1.92249644
F	-2.86575945	3.53445032	-0.33006367
F	-1.33739705	3.16255640	1.15298631
F	-0.80691816	4.08282871	-0.72860071

IRC start-point

C	1.85933645	-0.38976494	-1.54238597
C	0.78607923	-1.27238913	-1.58873433

C	-0.51933149	-0.79865707	-1.53105839
C	-0.74979508	0.57211749	-1.43115897
C	0.32098120	1.46337836	-1.40623161
C	1.62068353	0.97818005	-1.45134366
C	-2.13269979	1.12965630	-1.30538812
O	-2.36831827	2.31949474	-1.48242814
C	-4.30052511	0.48467571	-0.29095133
C	-5.31592403	1.08841715	-1.29349501
H	-4.95839491	2.05289370	-1.65723230
H	-6.27183950	1.22543842	-0.77884435
N	-3.11416472	0.19363289	-1.07665436
H	-1.84438012	-0.61046306	1.13617845
H	2.87672041	-0.76558612	-1.56683577
H	-5.46609586	0.41689720	-2.14219086
C	-4.07624757	1.44818037	0.88239451
H	-3.35794385	1.03273770	1.59477476
H	-3.71264758	2.41696874	0.53751722
H	-5.02231344	1.60022247	1.40973081
C	-4.82251047	-0.86710310	0.21688132
H	-5.77211176	-0.72511760	0.74071811
H	-4.97784554	-1.55754756	-0.61581730
H	-4.11086837	-1.31883427	0.91425865
C	-1.15978440	-0.90345848	1.94280692
C	0.06374666	0.01695318	1.92660098
C	1.11376399	-0.42375204	2.95956890
C	1.48846317	-1.89323195	2.75012561
C	0.26181005	-2.80489511	2.81835865
C	-0.79342443	-2.38109631	1.79465315
H	0.54285997	-0.07797535	0.94188037
H	-1.70462282	-0.75467162	2.88698026
H	1.96369206	-2.00262438	1.76517011
H	2.23261794	-2.19767994	3.49555487
H	0.55186555	-3.84735023	2.64966697
H	-0.16925193	-2.75459088	3.82762015
H	-0.39827495	-2.55855490	0.78833165
H	-1.69086868	-3.00278147	1.88898909
H	0.65846128	-0.32851930	3.96005540
C	2.33339951	0.50016765	2.90580176
C	-0.32450780	1.48581061	2.12094796
C	0.89103598	2.41512527	2.11865633
C	1.94599987	1.96304270	3.13075646
H	3.07453019	0.18238309	3.64874417
H	2.83070804	2.60516603	3.06495464
H	2.81050534	0.40165646	1.92051613
H	1.54604486	2.07753450	4.14757783
H	-0.85740143	1.58665984	3.07800333
H	-1.03132888	1.79255327	1.33961843
H	1.34099278	2.42250709	1.11908232
H	0.57846019	3.44429330	2.32549418
H	-1.35861960	-1.48318007	-1.55200367
H	0.12041368	2.52545339	-1.32696871
C	2.80223314	1.91043200	-1.39764809
C	1.07476920	-2.74625494	-1.69783799
F	3.65618829	1.54961885	-0.42189815
F	2.43561495	3.17783173	-1.16415085
F	3.49382355	1.89119807	-2.54775762
F	1.68318360	-3.04190732	-2.85703111

F	1.89546305	-3.15209998	-0.71140791
F	-0.04012618	-3.48713903	-1.62369144

IRC end-point

C	-2.07245292	0.04772649	-1.37666709
C	-1.07396412	0.96696402	-1.67504056
C	0.26279137	0.58073976	-1.67308814
C	0.60365588	-0.73585997	-1.37555150
C	-0.39688491	-1.67264452	-1.12914855
C	-1.72659953	-1.27369842	-1.10898114
C	2.03490725	-1.19534495	-1.26553785
O	2.35829807	-2.31951954	-1.62289447
C	4.28934332	-0.50429924	-0.41195102
C	5.04780353	-0.91005711	-1.68032569
H	4.66891684	-1.85392980	-2.07312540
H	6.11168840	-1.02250149	-1.45064233
N	2.87050310	-0.26526325	-0.73242250
H	2.42090543	0.46296632	-0.18317862
H	-3.11112693	0.35855355	-1.34694932
H	4.94033394	-0.13992223	-2.44990805
C	4.40837741	-1.58922015	0.66708396
H	3.88096252	-1.28272280	1.57659291
H	3.97796840	-2.52675227	0.30920374
H	5.45915316	-1.76013870	0.92053435
C	4.83774993	0.82614841	0.11232145
H	5.89531290	0.72013863	0.36727390
H	4.74250945	1.61149038	-0.64388420
H	4.30430795	1.14599700	1.01399871
C	1.36218225	1.30345838	1.71153515
C	0.22391808	0.35095909	1.88685547
C	-0.84061826	0.96832131	2.83399746
C	-1.24746266	2.35589284	2.33066815
C	-0.04858892	3.29874621	2.20695460
C	1.03377044	2.69693537	1.28107299
H	-0.29169297	0.24201442	0.92117272
H	2.26351043	1.13226872	2.30062244
H	-1.73148106	2.25284124	1.34946980
H	-1.99578174	2.78803050	3.00529071
H	-0.36410045	4.27330790	1.81954786
H	0.38357881	3.47065963	3.20068344
H	0.62624192	2.69237533	0.26087025
H	1.92429652	3.33257860	1.26721467
H	-0.37657989	1.08384095	3.82679880
C	-2.03118438	0.01646430	2.96884814
C	0.65705861	-1.03976288	2.35708674
C	-0.53582220	-1.97831857	2.55049641
C	-1.59843556	-1.36482082	3.46557209
H	-2.77692369	0.44907479	3.64622342
H	-2.46540748	-2.03034263	3.53120742
H	-2.51642659	-0.08812736	1.98859925
H	-1.19259629	-1.27087068	4.48207580
H	1.20060858	-0.93757935	3.30744806
H	1.36407378	-1.46860479	1.63643016
H	-0.98985977	-2.19253517	1.57612426
H	-0.19578380	-2.93874233	2.95174770

H	1.03693497	1.30790517	-1.89025887
H	-0.11555534	-2.69866852	-0.92326696
C	-2.82522565	-2.25049546	-0.78211856
C	-1.46669142	2.37992028	-2.01376112
F	-3.59218793	-1.79135883	0.22547384
F	-2.34551968	-3.44436294	-0.40904713
F	-3.63815425	-2.44621133	-1.83155082
F	-1.99384274	2.46442847	-3.24420818
F	-2.39282398	2.84686428	-1.15698931
F	-0.41787802	3.21884788	-1.96920417

Reaction between 6' and *trans* decalin – distal position

TS

C	-1.85418111	1.23556204	2.34643014
C	-1.06310795	0.13202704	2.63212318
C	0.13309014	-0.07472490	1.94878311
C	0.54428507	0.82393516	0.95315800
C	-0.25858209	1.93837817	0.68368896
C	-1.44868818	2.14076611	1.37721202
H	-1.36281889	-0.56608201	3.40351426
H	-2.06342730	3.00169611	1.14410799
C	1.88734216	0.67619025	0.26913792
O	2.74302615	1.52678339	0.47895889
C	3.29279835	-0.73717279	-1.18876616
C	4.46680546	-0.74919364	-0.18645712
H	4.63739339	0.24159846	0.23088887
H	5.37001256	-1.08287861	-0.70655817
N	2.05916026	-0.47704685	-0.42799109
H	0.89464119	-0.81059600	-1.15727210
C	-2.15536207	-0.32450722	-1.10454805
C	-3.31672124	0.64890374	-1.32085007
H	-2.93992630	1.67805485	-1.28132512
H	-3.72179228	0.50150365	-2.33267213
C	-1.06707504	-0.12481415	-2.15961716
H	-1.52481208	-0.25898324	-3.15362522
H	-0.69399910	0.90203396	-2.14126720
C	-2.62782798	-1.78427438	-1.06337298
H	-3.04593501	-2.03504446	-2.05316603
C	0.13643282	2.98662824	-0.33557916
C	0.98144732	-1.26357590	2.36265216
H	-2.78284418	1.39245599	2.88340719
F	0.80266389	2.47420522	-1.38539423
F	-0.96089132	3.58907817	-0.84059219
F	0.89694481	3.94317939	0.20088082
F	0.58265636	-1.73718493	3.55802027
F	0.89923439	-2.29214601	1.50304314
F	2.27497439	-0.93964076	2.48659913
H	4.25667252	-1.44517268	0.62953196
C	3.52200325	0.31043027	-2.28466728
H	2.69049517	0.31509817	-2.99647631
H	3.61200018	1.30634837	-1.84782629
H	4.43944232	0.08350732	-2.83646833
C	3.19241445	-2.14471292	-1.79023515
H	4.15948754	-2.42949787	-2.21356320

H	2.92519051	-2.86854997	-1.01512506
H	2.45463038	-2.20294301	-2.59291319
C	-1.43553181	-2.70282533	-0.79194896
H	-1.02510078	-2.46606323	0.19585910
H	-1.76434074	-3.74788543	-0.75382991
C	0.08480013	-1.10783512	-2.07015614
H	0.74280915	-1.00636009	-2.93805423
C	-4.43416228	0.44761566	-0.29607997
H	-4.05957626	0.71994975	0.69932009
H	-5.27280340	1.11791162	-0.51325399
C	-3.73517902	-1.96127944	-0.02104588
H	-3.31183698	-1.76669736	0.97561318
H	-4.08105296	-3.00166955	-0.01759283
C	-4.90642919	-1.00800748	-0.26671490
H	-5.37643720	-1.25533758	-1.22833595
H	-5.67457323	-1.14328254	0.50238018
C	-0.33741177	-2.55201624	-1.84529907
H	0.52871336	-3.16717921	-1.58515904
H	-0.71216078	-2.93458334	-2.80734411
H	-1.72358104	-0.11669414	-0.11862500

IRC start-point

C	-1.44648535	2.12282744	1.99702780
C	-1.14646310	0.85417256	2.47045097
C	-0.03221320	0.16732666	1.99692224
C	0.81332951	0.76046225	1.04692833
C	0.49739094	2.04144182	0.57708289
C	-0.62868593	2.71203599	1.04596696
H	-1.78109825	0.38835972	3.21386672
H	-0.86402165	3.69450715	0.65585119
C	2.08789341	0.08389251	0.59086464
O	3.17775963	0.56621176	0.86575898
C	2.86304110	-1.74081321	-0.88668524
C	4.00939731	-2.24424750	0.03201470
H	4.56035677	-1.40347154	0.45257531
H	4.68061259	-2.86309628	-0.57102289
N	1.89075513	-1.13348543	-0.00005012
H	0.31120982	0.07126998	-1.90745325
C	-2.35947605	0.25887056	-1.07752291
C	-3.58800000	1.04209716	-0.60615911
H	-3.28750334	2.05183472	-0.30014830
H	-4.27645420	1.16536628	-1.45479411
C	-1.62034642	0.97670468	-2.21018148
H	-2.31861187	1.14755408	-3.04251389
H	-1.29199684	1.96611894	-1.87051902
C	-2.75038771	-1.17635327	-1.47359232
H	-3.47310797	-1.10239754	-2.30414496
C	1.37043126	2.76238950	-0.42824822
C	0.19689687	-1.23229448	2.53290241
H	-2.32434890	2.64460551	2.36059329
F	1.87021416	1.93498672	-1.36216989
F	0.66520671	3.70229714	-1.08533812
F	2.40017917	3.37997559	0.15821178
F	-0.44660317	-1.40576474	3.70213897
F	-0.27211628	-2.16918118	1.69494064

F	1.49122820	-1.49134371	2.76416111
H	3.60620918	-2.85199677	0.84575568
C	3.40608926	-0.78441341	-1.95562650
H	2.59637116	-0.43401836	-2.60217403
H	3.89458077	0.07874488	-1.50165455
H	4.13449500	-1.31192229	-2.57798981
C	2.17719409	-2.95075778	-1.53031183
H	2.89050731	-3.50086834	-2.15086503
H	1.78440606	-3.62014075	-0.76087111
H	1.34599459	-2.63159132	-2.16202556
C	-1.53569643	-1.95834419	-1.98131319
H	-0.81647570	-2.07297888	-1.15704737
H	-1.84230406	-2.96825611	-2.27895746
C	-0.42565035	0.16666487	-2.71614021
H	0.07345044	0.69426758	-3.53601264
C	-4.31771235	0.33476766	0.53795492
H	-3.66585380	0.31915563	1.42127309
H	-5.21739599	0.89372848	0.81870524
C	-3.44634555	-1.88040819	-0.30549019
H	-2.73065949	-1.97794390	0.52277187
H	-3.72757420	-2.89888166	-0.59875552
C	-4.67855050	-1.10575776	0.16675704
H	-5.42534720	-1.09403650	-0.63883634
H	-5.14411559	-1.61161833	1.01965617
C	-0.86040882	-1.23341762	-3.14633647
H	-0.00261518	-1.80457154	-3.51840776
H	-1.56585673	-1.15583760	-3.98496903
H	-1.66464648	0.17301283	-0.22869864

IRC end-point

C	-1.68271100	1.40715800	2.32331900
C	-0.98605700	0.24600200	2.62502500
C	0.19826900	-0.05558100	1.95673800
C	0.69894800	0.80514700	0.96980900
C	-0.03319000	1.95444800	0.65469900
C	-1.21278300	2.25386600	1.33016900
H	-1.35206200	-0.42467900	3.39220500
H	-1.76656100	3.14732200	1.06826900
C	2.07582700	0.58854500	0.35884800
O	2.98629700	1.33834100	0.67453000
C	3.39985700	-0.91046900	-1.13902200
C	4.47891200	-1.19573700	-0.08700700
H	4.73397700	-0.28862800	0.46149700
H	5.37877100	-1.57755200	-0.57869400
N	2.16387200	-0.46671400	-0.47260700
H	1.29113200	-0.88502500	-0.77596300
C	-2.38494800	-0.16885100	-1.07726500
C	-3.52539900	0.85210900	-1.08023700
H	-3.10581800	1.86448800	-1.03070100
H	-4.06031000	0.78224400	-2.03857700
C	-1.43479900	0.06186100	-2.25915500
H	-2.04273500	0.08362600	-3.18410300
H	-0.97139200	1.05075100	-2.18882200
C	-2.90967800	-1.61321000	-1.06119900
H	-3.46121400	-1.78155100	-2.00194500

C	0.43286200	2.92125400	-0.41216900
C	0.92243100	-1.33251500	2.32744600
H	-2.59598800	1.64684100	2.85588000
F	1.04568100	2.29748100	-1.43322800
F	-0.61926500	3.57957600	-0.94208000
F	1.27190000	3.84103700	0.06802000
F	0.42820900	-1.86633800	3.45571900
F	0.79734100	-2.27684200	1.37084700
F	2.23203400	-1.13749300	2.52238600
H	4.12386300	-1.94608000	0.62445800
C	3.86770200	0.16255500	-2.12976600
H	3.09258600	0.35594000	-2.87786700
H	4.08203400	1.09405600	-1.60244100
H	4.77409400	-0.16774000	-2.64655600
C	3.05592000	-2.20830500	-1.87497500
H	3.95415800	-2.61841100	-2.34340900
H	2.66075600	-2.95387500	-1.17726600
H	2.31553400	-2.03908800	-2.66304600
C	-1.74155100	-2.60064600	-1.00473600
H	-1.19081500	-2.44110000	-0.06962200
H	-2.11854700	-3.62949000	-0.97873400
C	-0.39390800	-0.99848700	-2.39386800
H	0.47805900	-0.77569800	-3.00563700
C	-4.51389300	0.62236200	0.06379300
H	-4.00867800	0.81561600	1.01857500
H	-5.34473700	1.33290100	-0.00487600
C	-3.88389800	-1.81936600	0.10222600
H	-3.33159700	-1.70298400	1.04663400
H	-4.26836300	-2.84601000	0.08812800
C	-5.03764200	-0.81553400	0.06494300
H	-5.63347700	-0.98517500	-0.84219800
H	-5.70839300	-0.97547200	0.91603500
C	-0.78456100	-2.42712000	-2.19115900
H	0.10117500	-3.06116800	-2.06879300
H	-1.29010200	-2.79256400	-3.10406900
H	-1.81018800	-0.03494200	-0.15007100

Reaction between 6' and *trans* decalin – proximal position

TS

C	0.93389300	-3.50006800	0.69915600
C	1.95396600	-2.89629000	-0.02454300
C	2.12395000	-1.51607500	0.01699700
C	1.27550400	-0.71745600	0.79849400
C	0.27807700	-1.34223100	1.55077000
C	0.10099300	-2.72381600	1.48936100
H	2.62109900	-3.49690200	-0.63006800
H	-0.69216700	-3.18536500	2.06589900
C	1.49613000	0.78073600	0.87518900
O	2.07714100	1.25476300	1.84422900
C	1.24881400	2.91907100	-0.36746500
C	2.75668200	3.22835400	-0.45654500
H	3.25604900	2.95707000	0.47454600
H	2.89896100	4.29822300	-0.63881500
N	1.13694600	1.46085700	-0.23379500

H	0.06141800	0.82839700	-0.95982800
C	-0.60065100	-0.58186200	2.51942200
C	3.22859900	-0.91216600	-0.82785200
H	0.79667200	-4.57444500	0.65377700
F	-0.78332600	0.70191200	2.18053700
F	-1.82865100	-1.14157400	2.58643200
F	-0.09290000	-0.60532600	3.75638000
F	4.11360300	-1.85385500	-1.20269100
F	2.75880000	-0.35690900	-1.95921200
F	3.91385300	0.03252100	-0.17459700
H	3.20917900	2.66503800	-1.27622000
C	0.61190800	3.68613200	0.79859600
H	-0.46381000	3.49924500	0.85109100
H	1.06619200	3.39492100	1.74572100
H	0.75740800	4.76075900	0.64950600
C	0.58079200	3.32601000	-1.68480800
H	0.78065300	4.38265700	-1.88141900
H	0.97276800	2.73648100	-2.51879700
H	-0.50339800	3.19196000	-1.63502700
C	-0.85860100	0.33559700	-1.62139600
C	-2.10012800	0.21505200	-0.75951300
C	-3.23766600	-0.47167200	-1.55462400
C	-2.77123700	-1.79976500	-2.15417600
C	-1.52278000	-1.62529800	-3.01794900
C	-0.39098900	-0.98549300	-2.20070200
H	-1.86789700	-0.45134400	0.08289500
H	-0.99047400	1.08875800	-2.40806100
H	-2.54968300	-2.50109300	-1.33649400
H	-3.58406700	-2.24515800	-2.73966200
H	-1.19029900	-2.59036900	-3.41488700
H	-1.75914900	-0.99006200	-3.88166900
H	-0.13074100	-1.67099200	-1.38751700
H	0.50512200	-0.84769200	-2.80928600
H	-3.50643400	0.20044700	-2.38614200
C	-4.47099200	-0.64116400	-0.66147800
C	-2.57577600	1.55546300	-0.19938100
C	-3.81088600	1.38936400	0.68610100
C	-4.94201500	0.68892600	-0.06989600
H	-5.27846400	-1.11083600	-1.23571000
H	-5.79908700	0.52523700	0.59190400
H	-4.21973700	-1.33343700	0.15490300
H	-5.29085700	1.34026400	-0.88284700
H	-2.80849200	2.22881600	-1.03757300
H	-1.76477200	2.01808700	0.36758000
H	-3.53454000	0.79388300	1.56484300
H	-4.14514300	2.36432500	1.05590200

IRC start-point

C	-1.00186900	-0.19104900	2.98423300
C	-0.34689200	-1.31961500	2.51390200
C	0.69551200	-1.20146900	1.59780400
C	1.09420800	0.06210200	1.13985600
C	0.45310500	1.19651700	1.65608000
C	-0.59038300	1.06676900	2.56750400
H	-0.63532900	-2.30251600	2.86601200

H	-1.08281700	1.95536200	2.94203700
C	2.19206900	0.21276100	0.10788800
O	3.26260800	0.74248500	0.36144700
C	2.41642000	-0.00308600	-2.35629900
C	3.82783200	-0.64308400	-2.42531700
H	4.49054000	-0.17718600	-1.69499300
H	4.22656600	-0.48961600	-3.43270300
N	1.88797500	-0.41223700	-1.07010600
H	-0.46954900	-1.48582700	-1.29050300
C	0.90217900	2.59191200	1.27713900
C	1.39851800	-2.47902300	1.17482000
H	-1.82075200	-0.29062300	3.68744600
F	1.07181200	2.72520400	-0.05119900
F	-0.00654600	3.51284800	1.64594700
F	2.05477400	2.91986500	1.86665000
F	1.27265000	-3.41742400	2.13167500
F	0.89383700	-3.00908400	0.05280900
F	2.71129900	-2.29217100	0.98253100
H	3.77203600	-1.71581400	-2.22580000
C	2.50412300	1.51417600	-2.55616000
H	1.50807000	1.96433900	-2.53094300
H	3.12192300	1.98037600	-1.78767500
H	2.94513100	1.72385500	-3.53484100
C	1.51495200	-0.63959400	-3.42217200
H	1.92305200	-0.44498900	-4.41818400
H	1.44540200	-1.71966500	-3.27024600
H	0.50630900	-0.22039900	-3.37309100
C	-1.56205800	-1.45974300	-1.35592800
C	-2.08060700	-0.13250500	-0.79985700
C	-3.61780600	-0.08270900	-0.83512400
C	-4.21132900	-1.28035000	-0.08779500
C	-3.69440800	-2.61056000	-0.64026200
C	-2.16525500	-2.65761100	-0.62130900
H	-1.78378800	-0.07335100	0.25906300
H	-1.81816200	-1.52099000	-2.42447300
H	-3.94072200	-1.19954800	0.97577600
H	-5.30637300	-1.24500900	-0.13622800
H	-4.10996300	-3.44721600	-0.06777700
H	-4.04681800	-2.73047200	-1.67384800
H	-1.82018300	-2.64525600	0.42098400
H	-1.80201900	-3.59381300	-1.05753000
H	-3.92382800	-0.15847200	-1.89238900
C	-4.13596000	1.25213000	-0.29361700
C	-1.46699600	1.06981300	-1.51946200
C	-2.00558100	2.40002500	-0.99157200
C	-3.53373200	2.44077800	-1.04539000
H	-5.23087400	1.27728800	-0.34912900
H	-3.90336700	3.38353700	-0.62766800
H	-3.87295500	1.32724400	0.77227400
H	-3.86299200	2.40864400	-2.09296700
H	-1.68212600	0.99069500	-2.59571400
H	-0.37578500	1.03640000	-1.41324700
H	-1.68924500	2.53096900	0.04975800
H	-1.57474800	3.23518400	-1.55373800

IRC end-point

C	0.12896200	-3.14667300	1.60300100
C	1.23245600	-2.99457100	0.77524100
C	1.71745700	-1.72311400	0.47941500
C	1.10999900	-0.58303300	1.02129600
C	0.00887100	-0.75325300	1.86590200
C	-0.48278900	-2.02606500	2.14554300
H	1.71900300	-3.86299400	0.34885500
H	-1.34877200	-2.13308800	2.78783900
C	1.67877900	0.80528300	0.76466300
O	2.31354000	1.37188500	1.63972400
C	1.92742700	2.55072300	-1.00931700
C	3.46118100	2.52157900	-1.01386900
H	3.84408600	2.42676400	0.00353200
H	3.85062500	3.44537200	-1.45245000
N	1.41543300	1.28056700	-0.46798600
H	0.82962000	0.71235200	-1.07321400
C	-0.66382300	0.42222200	2.54178500
C	2.87770000	-1.60690100	-0.48559400
H	-0.25266500	-4.13675200	1.82479100
F	-0.65304100	1.53184100	1.78756400
F	-1.96012400	0.14366200	2.79782700
F	-0.08724600	0.71754600	3.70971800
F	3.49421600	-2.78780000	-0.65362000
F	2.46688600	-1.20874200	-1.70757600
F	3.80139800	-0.73108600	-0.07397600
H	3.82219200	1.67417400	-1.60307500
C	1.40662600	3.72586100	-0.17282900
H	0.31279300	3.74991000	-0.18118100
H	1.74497500	3.63562600	0.86049100
H	1.77556000	4.66976100	-0.58590600
C	1.40462700	2.64993500	-2.44461600
H	1.77997000	3.56287300	-2.91382100
H	1.74078800	1.79545000	-3.04170500
H	0.31047000	2.68232100	-2.46557900
C	-0.98243000	-0.22437400	-2.09415700
C	-2.00281500	0.19610100	-1.08576400
C	-3.31344600	-0.61220800	-1.29047400
C	-3.01591100	-2.11344600	-1.31043000
C	-2.00470100	-2.47997400	-2.39789100
C	-0.69410800	-1.68581200	-2.21019300
H	-1.63766400	-0.09314500	-0.08689700
H	-0.79661600	0.42498800	-2.94891900
H	-2.61791100	-2.41050100	-0.32849300
H	-3.94793900	-2.67232600	-1.45572300
H	-1.79171000	-3.55450400	-2.38084500
H	-2.43127400	-2.25259700	-3.38287600
H	-0.22956700	-2.04305800	-1.28152500
H	0.00939900	-1.89383100	-3.02050600
H	-3.72000400	-0.33288900	-2.27595300
C	-4.34181800	-0.22506300	-0.22568300
C	-2.27433000	1.70245200	-1.07456500
C	-3.34872100	2.08824700	-0.05484300
C	-4.63192200	1.27720700	-0.24768000
H	-5.26541600	-0.79642400	-0.37727200
H	-5.36032000	1.53414300	0.52872200
H	-3.95113100	-0.50575800	0.76277000
H	-5.09058500	1.53982400	-1.21085000

H	-2.59118500	2.01166300	-2.08108500
H	-1.34324300	2.23508400	-0.85294700
H	-2.96103300	1.90862800	0.95355500
H	-3.56122600	3.16036800	-0.12470300

Reaction between 3' and 7 – primary position

TS

C	-2.14973058	0.65480438	-0.86692853
C	-1.10367308	1.56737548	-0.80652036
C	0.21567694	1.14153239	-0.91685070
C	0.49766553	-0.21250149	-1.06634418
C	-0.54736444	-1.12949109	-1.14925077
C	-1.86302085	-0.69531928	-1.04492991
C	1.90548329	-0.73832367	-1.11605161
O	2.13929525	-1.81371703	-1.65708423
C	4.24785496	-0.32490080	-0.40272899
C	4.83979281	-0.58003479	-1.80266311
H	4.37380386	-1.44503360	-2.27287995
H	5.91498215	-0.75851206	-1.70484217
N	2.84689947	0.09839844	-0.56883778
H	2.37185330	0.47935195	0.64227322
C	1.90002212	0.62911034	1.80840776
C	1.10481432	-0.60541163	2.20522591
H	1.21848182	-1.38531125	1.44008369
H	1.52870012	-1.03921490	3.12059952
H	-3.17480164	0.98844927	-0.76516654
H	4.69276305	0.29513853	-2.44183039
C	4.37486317	-1.57540533	0.47729308
H	3.97306800	-1.38510279	1.47809970
H	3.83665972	-2.41492187	0.03400796
H	5.42774644	-1.85298105	0.58503782
C	4.99830402	0.85393519	0.22840203
H	6.06534051	0.62334414	0.29277338
H	4.87196241	1.75603618	-0.37627023
H	4.64211568	1.06502951	1.24005226
H	-0.31101857	-2.18011433	-1.27886809
H	1.03037408	1.85535071	-0.87362444
C	-2.97843788	-1.70188441	-1.14803875
C	-1.36413367	3.01602294	-0.49941376
F	-2.65042795	3.35103551	-0.66808712
F	-1.04207138	3.28937099	0.78143566
F	-0.62352512	3.82844069	-1.26857815
F	-4.13048303	-1.22484111	-0.64894640
F	-2.68281711	-2.82933349	-0.47871476
F	-3.20943034	-2.05050156	-2.42318405
H	1.30395682	1.53634644	1.67978864
H	2.78398884	0.82960230	2.41486958
C	-0.39389731	-0.33814592	2.41948828
H	-0.75839461	0.22568922	1.55108581
C	-1.17093799	-1.65940719	2.47049029
H	-0.94427953	-2.22983209	1.56183676
H	-0.80353969	-2.26130417	3.31316808
C	-2.68522470	-1.49465867	2.57716285
H	-3.18946254	-2.45925781	2.47773643

H	-2.98276719	-1.06143010	3.53628938
H	-3.06498550	-0.84228520	1.78407000
C	-0.62212454	0.52177395	3.66483266
H	-0.34242781	-0.02953105	4.57067165
H	-0.02473526	1.43843634	3.63309970
H	-1.66884128	0.82104321	3.76165677

IRC start-point

C	-1.84907632	0.26254706	1.12420943
C	-0.77181852	-0.51571553	1.54453554
C	0.53070756	-0.09406088	1.32504431
C	0.76159562	1.11598310	0.66805946
C	-0.30793938	1.89629310	0.24503466
C	-1.61130663	1.46527390	0.47479766
C	2.14727277	1.58158037	0.34885752
O	2.36759573	2.69018019	-0.12030540
C	4.27240058	0.43588314	-0.17926029
C	5.30295221	1.56339258	0.07206353
H	4.89632797	2.52583330	-0.24050733
H	6.20614765	1.34162145	-0.50480916
N	3.15216284	0.70925412	0.70594821
H	2.32929420	-2.49477610	-1.10541537
C	1.25624090	-2.71162270	-1.12350912
C	0.49571846	-1.65230949	-1.91855660
H	0.73594447	-0.66204507	-1.51167982
H	0.84698395	-1.64692648	-2.95991486
H	-2.86523512	-0.07571931	1.28875878
H	5.56593039	1.61371010	1.13140537
C	3.87839053	0.39182467	-1.66243614
H	3.12558664	-0.38231369	-1.84000596
H	3.48055569	1.35287532	-1.99424983
H	4.75615630	0.15455737	-2.26984711
C	4.86182667	-0.90825201	0.26696910
H	5.75109019	-1.14176625	-0.32545590
H	5.14046767	-0.87298927	1.32305529
H	4.13631012	-1.71512007	0.12926904
H	-0.10465578	2.82842474	-0.27155030
H	1.36967365	-0.69788563	1.65069065
C	-2.75232057	2.32397616	-0.00426976
C	-1.05249778	-1.81222938	2.25628080
F	-1.49345894	-1.60248382	3.50630299
F	-2.00169864	-2.51949242	1.61852796
F	0.04083977	-2.58693442	2.34055897
F	-3.94141380	1.73094014	0.17611491
F	-2.63022561	2.60184872	-1.31382554
F	-2.78231647	3.49878418	0.64378418
H	0.90535146	-2.75429119	-0.08747433
H	1.13801078	-3.70982264	-1.55420216
C	-1.03106250	-1.81774196	-1.90641871
H	-1.35083736	-1.86838870	-0.85614419
C	-1.69969838	-0.58792045	-2.53750643
H	-1.26433612	0.31467165	-2.09291716
H	-1.44687554	-0.55263008	-3.60618094
C	-3.21598399	-0.53052043	-2.36560627
H	-3.61635345	0.41257006	-2.74584847

H	-3.72091980	-1.34347698	-2.89498437
H	-3.49162886	-0.60227114	-1.30776126
C	-1.46755609	-3.11618484	-2.58975131
H	-1.16210941	-3.11831163	-3.64318509
H	-1.02645144	-3.99396744	-2.10959791
H	-2.55294129	-3.24292232	-2.55503749

IRC end-point

C	-2.09337593	0.28319806	-1.06055187
C	-1.06370101	1.19826798	-1.24271466
C	0.26501469	0.78738828	-1.20485867
C	0.57387761	-0.54934627	-0.97017027
C	-0.45559669	-1.47274709	-0.80831815
C	-1.78010085	-1.05493707	-0.84603337
C	1.98832119	-1.06784230	-0.88898109
O	2.23557403	-2.22787399	-1.19035440
C	4.32557600	-0.47501645	-0.20651909
C	4.98953453	-0.96166837	-1.49940884
H	4.52632114	-1.88601634	-1.84597585
H	6.05342614	-1.14391904	-1.32025687
N	2.90644224	-0.16405994	-0.46059940
H	2.54682043	0.65793864	0.01249480
C	1.51521808	1.83076302	1.80194339
C	0.92964316	0.60230246	2.40978445
H	1.26050064	-0.28853190	1.84742408
H	1.32908221	0.45858330	3.42428572
H	-3.12600495	0.60879381	-1.07934909
H	4.89582912	-0.20456355	-2.28353630
C	4.44181828	-1.53252454	0.89976793
H	3.97023728	-1.17421239	1.82087411
H	3.95425964	-2.45903800	0.59148702
H	5.49439811	-1.74307002	1.11233297
C	4.97602539	0.83443378	0.24782207
H	6.04079902	0.67644470	0.43638739
H	4.87461156	1.60883344	-0.51905562
H	4.52150082	1.19920057	1.17537254
H	-0.19830465	-2.51435574	-0.65009297
H	1.05358035	1.51316622	-1.37119493
C	-2.87452777	-2.07791914	-0.68967296
C	-1.36640065	2.66535109	-1.37222378
F	-2.62053197	2.89183582	-1.78362499
F	-1.21927975	3.28784057	-0.18317041
F	-0.53420554	3.27149594	-2.23274668
F	-4.04684112	-1.50997471	-0.36141634
F	-2.57359381	-2.97180761	0.26648641
F	-3.06845799	-2.76519470	-1.82579305
H	0.91522349	2.45900947	1.15005410
H	2.48856581	2.20271694	2.10546329
C	-0.60737926	0.60555624	2.45621988
H	-0.96574395	0.84625521	1.44660834
C	-1.13880572	-0.78718129	2.81666593
H	-0.73847874	-1.50920393	2.09426107
H	-0.74207142	-1.08040321	3.79854254
C	-2.66205861	-0.89426200	2.82727973
H	-2.97990592	-1.93289809	2.94738800

H	-3.10443054	-0.31465318	3.64240032
H	-3.08860837	-0.52643391	1.88848139
C	-1.11599483	1.69279246	3.40517365
H	-0.84204370	1.46006729	4.44138401
H	-0.68231729	2.66561453	3.15392749
H	-2.20326807	1.79467599	3.35848322

Reaction between 3' and 7 – secondary position

TS

C	-2.55191971	0.50172741	-0.45586951
C	-1.55429809	1.46066580	-0.58086320
C	-0.24890794	1.08630187	-0.88432430
C	0.06180288	-0.25777836	-1.06381895
C	-0.94021232	-1.22079481	-0.97147781
C	-2.23802497	-0.83982124	-0.65659009
C	1.46378774	-0.71509990	-1.35017901
O	1.65689354	-1.73317302	-2.00595255
C	3.86282397	-0.22184200	-0.95584867
C	4.23425682	-0.30095109	-2.45008653
H	3.73787081	-1.14258930	-2.93184044
H	5.31804471	-0.42288487	-2.54017395
N	2.43511869	0.12373700	-0.86475405
H	2.11136552	0.38691992	0.46219774
C	1.93661052	0.61442941	1.68489115
C	1.76236792	2.11717307	1.76136699
H	1.92443151	2.47579994	2.78414017
H	2.48332923	2.62912365	1.11753721
H	0.75524514	2.41843764	1.46191115
C	0.79799455	-0.25075828	2.20742813
H	-0.11540413	0.01071901	1.65660993
H	-3.56759911	0.79596877	-0.21968586
H	3.94778854	0.62228661	-2.96176010
C	4.19579679	-1.54137363	-0.24729901
H	3.94642004	-1.48279957	0.81689935
H	3.64050487	-2.36815020	-0.69328293
H	5.26625300	-1.75205983	-0.33214038
C	4.64824447	0.93663123	-0.32846697
H	5.72105531	0.77541675	-0.46658584
H	4.37519383	1.88390823	-0.80210028
H	4.46150844	1.02066332	0.74520513
H	-0.68586907	-2.26114329	-1.14059401
H	0.53333574	1.83020945	-0.98136513
C	-3.30461834	-1.88513711	-0.46483493
C	-1.86459962	2.90926943	-0.31727899
F	-1.59624339	3.23446193	0.96474848
F	-1.12979341	3.72404763	-1.08797946
F	-3.15666276	3.19525806	-0.53086729
F	-4.51655367	-1.42814192	-0.81637525
F	-3.38756059	-2.25976675	0.82721378
F	-3.05820305	-2.98935353	-1.18164550
H	2.89648258	0.29023070	2.10554937
C	1.08938435	-1.74215281	1.98358239
H	1.96942278	-2.02303251	2.57880535
H	1.37492448	-1.90053768	0.93793274

C	-0.07674711	-2.67008222	2.31754901
H	-0.98488958	-2.37110762	1.78515292
H	0.15468118	-3.69935314	2.03010337
H	-0.30542577	-2.67389530	3.38700473
C	0.54457728	0.06166970	3.69118160
H	0.28596466	1.11316433	3.83993287
H	-0.28407996	-0.53611649	4.07877778
H	1.43370274	-0.16096096	4.29244732

IRC start-point

C	-2.32703646	0.53300171	-0.65451034
C	-1.29271549	1.45818812	-0.74516419
C	0.00707677	1.03980073	-1.00174476
C	0.27164647	-0.31778360	-1.17297966
C	-0.76071194	-1.24929880	-1.08867107
C	-2.05469361	-0.82070809	-0.82570046
C	1.65727617	-0.82322227	-1.42729291
O	1.86509588	-1.96180617	-1.83132442
C	3.98617576	-0.23502449	-0.81280125
C	4.76822542	-0.63217108	-2.08974399
H	4.35497545	-1.54809747	-2.51495112
H	5.81525775	-0.79768162	-1.81814934
N	2.65316436	0.11268973	-1.27298110
H	1.79456342	0.52340672	1.14651627
C	1.43851439	0.70477820	2.16850733
C	1.12526204	2.19181577	2.31855668
H	0.95354275	2.46796168	3.36263258
H	1.95317747	2.80505577	1.94984842
H	0.22872479	2.46627757	1.75536774
C	0.26043608	-0.24204499	2.43947923
H	-0.53788198	0.00871097	1.72436024
H	-3.34061130	0.86462160	-0.45605264
H	4.71964981	0.16464306	-2.83574298
C	4.03757082	-1.37161057	0.21695680
H	3.48544021	-1.10056366	1.12098990
H	3.62170198	-2.29504886	-0.18789566
H	5.07807970	-1.55417817	0.50028280
C	4.58427269	1.04990252	-0.22207444
H	5.62747306	0.87898485	0.05874176
H	4.54273061	1.86434550	-0.94953518
H	4.03410872	1.35464628	0.67312427
H	-0.53391730	-2.30019397	-1.22607437
H	0.81502804	1.75783304	-1.07303495
C	-3.18207761	-1.80873114	-0.68001920
C	-1.61063630	2.91518368	-0.53698135
F	-2.01328491	3.14276125	0.72760477
F	-0.55225751	3.70288693	-0.76856660
F	-2.60679803	3.31956975	-1.34065588
F	-4.26545653	-1.41923840	-1.37083660
F	-3.56091180	-1.91997796	0.60743769
F	-2.84123583	-3.03123076	-1.10511853
H	2.27287513	0.43605643	2.83246047
C	0.68427684	-1.69379714	2.17181346
H	1.49526397	-1.96129393	2.86398005
H	1.11756369	-1.75259595	1.16592390

C	-0.44066607	-2.72117647	2.28137287
H	-1.30574665	-2.42540856	1.68017889
H	-0.10788132	-3.70244115	1.93118709
H	-0.78424708	-2.84131883	3.31269604
C	-0.30167895	-0.06231533	3.85146862
H	-0.64013681	0.96331163	4.02110940
H	-1.15817663	-0.71818341	4.02800318
H	0.46192428	-0.29488595	4.60388434

IRC end-point

C	-2.44818009	0.23883043	-0.69214291
C	-1.42960772	1.11582026	-1.04195045
C	-0.12230250	0.66000333	-1.18439650
C	0.17036883	-0.68447572	-0.97483036
C	-0.85244396	-1.57197633	-0.64774952
C	-2.15215494	-1.10742393	-0.49877273
C	1.56554773	-1.24301335	-1.10098957
O	1.73789704	-2.39722817	-1.46807379
C	3.98507176	-0.70786876	-0.72350439
C	4.45063609	-1.21611752	-2.09242858
H	3.93560591	-2.13890028	-2.35973359
H	5.52791429	-1.40569496	-2.06473760
N	2.55204947	-0.36657657	-0.77559617
H	2.27023828	0.40549205	-0.17677268
C	1.71158545	1.54093400	1.71892999
C	1.64666650	2.88784856	1.08016624
H	1.78097615	3.69256808	1.82020592
H	2.43822106	3.02366100	0.33361087
H	0.68000121	3.05982357	0.59901527
C	0.49827159	0.87502538	2.29445159
H	-0.30921546	0.92630661	1.54936160
H	-3.46570678	0.59689314	-0.58399192
H	4.25203015	-0.46598498	-2.86367325
C	4.23253716	-1.76081347	0.36528862
H	3.90439888	-1.38569437	1.34052614
H	3.68124210	-2.67526423	0.13730191
H	5.29845621	-2.00014899	0.42933078
C	4.72291973	0.59082320	-0.38389596
H	5.80028193	0.41004354	-0.34884803
H	4.52752878	1.35777527	-1.14000009
H	4.41707878	0.97910113	0.59339940
H	-0.61176424	-2.62050844	-0.51647925
H	0.66115733	1.35244611	-1.47109578
C	-3.25517409	-2.03893172	-0.07020396
C	-1.73834899	2.58113885	-1.18393282
F	-1.74884226	3.18691853	0.02218207
F	-0.82651468	3.21808862	-1.93353057
F	-2.94231712	2.78952851	-1.73457226
F	-4.39393081	-1.79106536	-0.73710184
F	-3.53064958	-1.88806037	1.23985581
F	-2.93617689	-3.32391193	-0.26602451
H	2.67850814	1.21591420	2.10672765
C	0.78156618	-0.60430536	2.59781842
H	1.57019947	-0.66209210	3.36093235
H	1.19858752	-1.06892095	1.69671637

C	-0.43546397	-1.40371970	3.05558162
H	-1.26091493	-1.31331676	2.34310898
H	-0.18966860	-2.46546370	3.14619392
H	-0.80060302	-1.06835673	4.03057693
C	0.00440098	1.64156582	3.53864918
H	-0.18348901	2.69228095	3.29972030
H	-0.92900317	1.21727540	3.91906757
H	0.75092405	1.60108093	4.33949400

Reaction between 6' and 7 – primary position

TS

C	0.99534707	-3.13806727	0.93186499
C	1.43107010	-1.97571620	1.55162607
C	0.67345406	-0.80951410	1.47493406
C	-0.53411901	-0.79505908	0.76475998
C	-0.97347104	-1.98005516	0.16583090
C	-0.21062599	-3.14224325	0.24657091
H	2.36066415	-1.97071622	2.10729713
H	-0.56385002	-4.04654731	-0.23388715
C	-1.38151206	0.46209002	0.69611997
O	-2.40059315	0.54462202	1.36449799
C	-1.52489902	2.71827820	-0.32185307
C	-1.68662006	3.44387523	1.02846403
H	-2.36802013	2.90002318	1.68210205
H	-2.08445807	4.44731330	0.84849002
N	-0.82382499	1.45386109	-0.05545105
H	-0.34465593	0.92285507	-1.21718214
C	-0.05769189	0.45808706	-2.35160722
C	1.39686323	0.77461707	-2.60739319
H	1.63806526	0.51377507	-3.65019926
H	1.54552124	1.85805114	-2.52507416
C	-2.31197112	-2.05490614	-0.53706418
C	1.19639009	0.42125796	2.19183214
H	1.59178510	-4.04140534	0.98963500
F	-2.58307211	-0.94518804	-1.24932223
F	-2.34317011	-3.08435920	-1.40344026
F	-3.31482521	-2.23221315	0.32719685
F	2.08729213	0.07531091	3.13886923
F	1.83142416	1.27437603	1.36837311
F	0.22118601	1.10668201	2.80009617
H	-0.71750800	3.53438422	1.52579009
C	-2.88497610	2.50078820	-0.99521216
H	-2.76264308	1.98193019	-1.95102023
H	-3.53939817	1.90517016	-0.35712214
H	-3.36453912	3.46484028	-1.19073317
C	-0.60780092	3.56056027	-1.21644410
H	-1.01959094	4.56755034	-1.32584910
H	0.39014714	3.63536625	-0.77500904
H	-0.51434190	3.12968925	-2.21711918
H	-0.29433692	-0.60268602	-2.26705423
H	-0.75958892	0.95006811	-3.02770827
C	2.36579227	0.05106399	-1.66171311
C	3.75737037	0.69546602	-1.70810207
H	4.19176842	0.55591903	-2.70787513

H	3.64172337	1.77761610	-1.57032105
C	4.71854841	0.16821096	-0.64358598
H	4.96295942	-0.88686212	-0.79838500
H	5.65886148	0.72653899	-0.65266795
H	4.27757535	0.27101496	0.35356408
C	2.41803026	-1.45016411	-1.95378615
H	2.84702532	-1.63582311	-2.94593222
H	3.02707329	-1.98123416	-1.21749109
H	1.42345918	-1.90457914	-1.92505018
H	1.98323722	0.19084899	-0.64643104

IRC start-point

C	2.33534382	1.64112345	-1.75486222
C	2.15723185	0.27738414	-1.93526533
C	0.94123756	-0.32311467	-1.62255522
C	-0.12132110	0.44470803	-1.12464478
C	0.07459639	1.81801789	-0.94205014
C	1.29501056	2.40935568	-1.25659731
H	2.96492980	-0.33029494	-2.32356727
H	1.42889543	3.47179632	-1.09453615
C	-1.47189063	-0.17793292	-0.84049054
O	-2.43767612	0.07011060	-1.54821056
C	-2.62210199	-1.53463746	0.87995891
C	-3.34599584	-2.51498263	-0.08303358
H	-3.74734842	-1.97588157	-0.94162769
H	-4.16261286	-2.99300158	0.46630566
N	-1.43908588	-1.11401596	0.15654346
H	-0.68004256	0.59794456	2.10999401
C	-0.18520732	0.71709926	3.07935079
C	1.04076259	-0.18804679	3.16823472
H	1.57622259	0.00324909	4.10865049
H	0.71125184	-1.23386042	3.20858360
C	-1.02746568	2.71706525	-0.42748287
C	0.83906495	-1.82377753	-1.80514194
H	3.28626648	2.10221904	-1.99615741
F	-1.78937811	2.11430400	0.50294368
F	-0.51573797	3.81926206	0.14859610
F	-1.83945724	3.12100579	-1.40992523
F	1.75735879	-2.26069253	-2.68606201
F	1.06531314	-2.48136704	-0.65713886
F	-0.35827219	-2.20811504	-2.26889943
H	-2.65609736	-3.28611490	-0.43382645
C	-3.56115875	-0.39453861	1.29262964
H	-3.05864125	0.28515830	1.98566540
H	-3.90318079	0.17211430	0.42603893
H	-4.43291557	-0.81392441	1.80309606
C	-2.13393731	-2.30909942	2.11056699
H	-2.98523496	-2.75358171	2.63429543
H	-1.44483539	-3.10336777	1.81288432
H	-1.61484000	-1.64283519	2.80393100
H	0.07285989	1.77435799	3.18700828
H	-0.91349396	0.47828304	3.86096266
C	2.01540492	-0.03826966	1.99217912
C	3.16820097	-1.04508603	2.12233387
H	3.81661315	-0.73820881	2.95471608

H	2.75171991	-2.02061412	2.40221042
C	4.00094854	-1.22263584	0.85359427
H	4.44713124	-0.28082788	0.51856785
H	4.81695806	-1.93289473	1.01584242
H	3.37872884	-1.60985532	0.04185314
C	2.53186263	1.39811419	1.87114760
H	3.02373045	1.70685868	2.80182859
H	3.25572033	1.49991929	1.05812929
H	1.72230757	2.10454352	1.66719526
H	1.45896315	-0.28800712	1.07897609

IRC end-point

C	-2.71554360	1.23037003	1.70085332
C	-2.24698732	-0.04996422	1.95474114
C	-0.93272297	-0.39011337	1.64541934
C	-0.06330459	0.55328005	1.08195722
C	-0.55393539	1.83732933	0.82178614
C	-1.87073533	2.16944707	1.12841632
H	-2.90099688	-0.79293172	2.39380920
H	-2.23346175	3.16539105	0.90548694
C	1.40500569	0.23800533	0.83278429
O	2.26139688	0.75629695	1.53005257
C	2.94129970	-1.09791854	-0.63307716
C	3.63998131	-1.83092468	0.51812586
H	3.81897102	-1.15081874	1.35225960
H	4.59939979	-2.23058526	0.17596424
N	1.62307508	-0.61658778	-0.18620116
H	0.81916138	-0.94753448	-0.69646758
C	0.29677664	0.32783544	-3.25041977
C	-0.78483573	-0.69541321	-3.23003947
H	-1.32990483	-0.68119004	-4.18663587
H	-0.34182971	-1.70698366	-3.17842412
C	0.32051887	2.92083774	0.22674167
C	-0.49704016	-1.82035042	1.86964911
H	-3.73946567	1.49280500	1.94127084
F	1.19403123	2.44151144	-0.67627699
F	-0.43221404	3.83420294	-0.41693818
F	1.01858002	3.56969615	1.16112374
F	-1.35582880	-2.48025035	2.66091129
F	-0.45357912	-2.50284852	0.70077358
F	0.71437691	-1.91420972	2.42784202
H	3.02111095	-2.66073993	0.87061444
C	3.78053393	0.08697531	-1.12503367
H	3.27463617	0.59031649	-1.95425950
H	3.93266803	0.80608741	-0.31830930
H	4.75626110	-0.26471585	-1.47394044
C	2.66785963	-2.06697018	-1.78595155
H	3.60908764	-2.47539154	-2.16208154
H	2.04234620	-2.90240504	-1.45280939
H	2.16281231	-1.55313052	-2.61166524
H	0.54706134	0.90572967	-2.36785339
H	0.90427814	0.47055766	-4.13748620
C	-1.79499133	-0.54929521	-2.08048278
C	-2.80061727	-1.70961900	-2.10844936
H	-3.45574517	-1.58560555	-2.98147496

H	-2.25327027	-2.64712785	-2.26776620
C	-3.64350248	-1.85148041	-0.84275974
H	-4.20619530	-0.94001317	-0.61885423
H	-4.36381029	-2.66839310	-0.94273760
H	-3.00600762	-2.07347921	0.01769519
C	-2.48711856	0.81466991	-2.12522620
H	-3.03931749	0.93016455	-3.06539594
H	-3.19263562	0.93483041	-1.29884689
H	-1.76408882	1.63279924	-2.05965627
H	-1.24590736	-0.61502027	-1.13057547

Reaction between 6' and 7 – secondary position

TS

C	-1.25377947	-3.43262425	0.53724514
C	-2.21363647	-2.44863555	0.73578100
C	-2.02236177	-1.16674505	0.23012854
C	-0.85946646	-0.85329291	-0.48900922
C	0.07827267	-1.86310611	-0.71600195
C	-0.11381204	-3.14212014	-0.19590288
H	-3.11915191	-2.67484766	1.28484706
H	0.63435791	-3.90652390	-0.37012261
C	-0.67063470	0.53628423	-1.06529516
O	-0.92144731	0.74012094	-2.24646110
C	-0.12530731	2.89154114	-0.49630155
C	-1.49899106	3.46771757	-0.89567869
H	-1.86669559	2.98337998	-1.80119698
H	-1.40259713	4.54224531	-1.08038737
N	-0.36073057	1.48253952	-0.15102599
H	0.40581664	0.95247439	0.94652932
C	1.00037679	0.57208451	1.96749859
C	0.04887442	-0.33044154	2.72480165
H	0.37675187	-0.43960650	3.76481754
H	-0.96049224	0.08146613	2.73796851
H	0.00970541	-1.32974801	2.28025754
C	2.33999102	-0.03894540	1.58027130
C	1.28703920	-1.65074741	-1.59986800
C	-3.10470018	-0.13436163	0.48319987
H	-1.40266491	-4.42718336	0.94203857
F	1.72549427	-0.38333064	-1.60466596
F	2.32080640	-2.41497975	-1.18663578
F	1.02877698	-1.99333088	-2.86640211
F	-4.25429324	-0.72862840	0.85043381
F	-2.78470367	0.72008002	1.47290253
F	-3.36992289	0.60030503	-0.60193788
H	-2.22234055	3.30883600	-0.09224509
C	0.88579350	3.08225514	-1.63463044
H	1.87179849	2.70186692	-1.35535778
H	0.55368263	2.56731740	-2.53604474
H	0.98955173	4.14992061	-1.85269495
C	0.36458886	3.61270643	0.76350416
H	0.39839913	4.68900842	0.57492738
H	-0.30984195	3.42821481	1.60483851
H	1.37185707	3.28831362	1.03900200
H	1.13549172	1.52876181	2.48546988

C	3.18840390	0.94869105	0.77127337
H	2.56379025	1.35120274	-0.03024979
H	3.45838290	1.79742193	1.41527099
C	4.44559481	0.34352785	0.15093463
H	5.16513700	0.01888892	0.90816150
H	4.18920954	-0.52163152	-0.46704474
H	4.95065984	1.07312080	-0.48818363
C	3.08115276	-0.52640114	2.83560459
H	3.26426539	0.30647851	3.52449600
H	2.51082473	-1.29062578	3.36963777
H	4.04712542	-0.96423815	2.57151995
H	2.14385777	-0.91861667	0.95403464

IRC start-point

C	-1.41788624	1.94806365	-2.28494931
C	-0.06094716	2.10780412	-2.04636160
C	0.69042902	1.06364210	-1.51293675
C	0.08208809	-0.16288721	-1.21134574
C	-1.28170165	-0.32312956	-1.49162651
C	-2.02392213	0.72844249	-2.02046948
H	0.42730522	3.04444460	-2.28609573
H	-3.07911310	0.58742029	-2.21832526
C	0.87036023	-1.29837540	-0.59311631
O	1.07320161	-2.35138979	-1.17680524
C	1.69871894	-1.90508317	1.67001487
C	3.05704231	-2.54191655	1.27560066
H	2.94310654	-3.14923163	0.37680225
H	3.39507505	-3.17186128	2.10402778
N	1.41360370	-0.95486526	0.61393036
H	0.66430523	1.38837607	1.54004805
C	0.02603238	2.15436821	1.99476833
C	0.44969831	3.52318310	1.46715475
H	-0.05880229	4.34048337	1.98696444
H	1.52554496	3.67224706	1.58794936
H	0.22066892	3.61396547	0.39946738
C	-1.43997080	1.78941909	1.72547201
C	-1.97891342	-1.65042074	-1.28103252
C	2.17919996	1.29913175	-1.33068855
H	-1.99907393	2.76797864	-2.69104045
F	-1.66718664	-2.20605143	-0.09624996
F	-3.31608547	-1.50556495	-1.30145696
F	-1.66429506	-2.53101912	-2.23503558
F	2.60756643	2.25189806	-2.17977968
F	2.49619716	1.71643280	-0.09807430
F	2.89843168	0.19784483	-1.58662211
H	3.80453797	-1.76653113	1.09199739
C	0.63044004	-2.98873136	1.85403059
H	-0.32432748	-2.54172855	2.14422529
H	0.48733952	-3.56403914	0.93848193
H	0.94136919	-3.67060299	2.65068306
C	1.87159466	-1.08766687	2.95663569
H	2.18754658	-1.74216206	3.77412424
H	2.62176902	-0.30564637	2.81501506
H	0.92861909	-0.61149727	3.23849198
H	0.21071421	2.10500211	3.07754645

C	-1.69400036	0.33070515	2.12916206
H	-0.97843058	-0.30008898	1.58672135
H	-1.46039798	0.21063451	3.19697201
C	-3.10715564	-0.17894067	1.85817048
H	-3.84475997	0.30958595	2.50097454
H	-3.39813705	0.00350496	0.81871738
H	-3.17640752	-1.25553994	2.03395740
C	-2.40699221	2.75122457	2.42034772
H	-2.29260701	2.69135821	3.50973162
H	-2.22682533	3.78754440	2.12195157
H	-3.44875634	2.52233337	2.17951756
H	-1.60744051	1.86436766	0.64000772

IRC end-point

C	-0.58931900	-3.58452300	0.02983800
C	-1.67839000	-2.82271800	0.43076900
C	-1.74919700	-1.47283800	0.10009700
C	-0.73121100	-0.86638000	-0.64880600
C	0.35687900	-1.64647800	-1.04868800
C	0.42845000	-2.99502000	-0.70493800
H	-2.47835700	-3.27465200	1.00384900
H	1.28649600	-3.57916000	-1.01585400
C	-0.85289000	0.58450900	-1.09335000
O	-1.16390400	0.84680800	-2.24360800
C	-0.74839900	2.93051100	-0.22330500
C	-2.20033200	3.27469500	-0.57833900
H	-2.46754900	2.84672000	-1.54606600
H	-2.32586600	4.36064600	-0.62611000
N	-0.60957200	1.46947500	-0.10733300
H	-0.28822500	1.09185300	0.77812200
C	1.09037800	0.12796700	2.37898000
C	0.32597000	-1.05235100	2.87421300
H	0.66974700	-1.36659000	3.87277300
H	-0.74248100	-0.83783500	2.95978000
H	0.45238700	-1.91142600	2.20630300
C	2.41109000	-0.04576700	1.69147300
C	1.46576500	-1.08924600	-1.91500900
C	-2.93319400	-0.67371500	0.60129400
H	-0.53443500	-4.63512000	0.29133900
F	1.69226200	0.21493500	-1.70480400
F	2.62879600	-1.72750400	-1.66178400
F	1.20306000	-1.25270900	-3.21513300
F	-3.91631600	-1.47617200	1.03890600
F	-2.59421500	0.12032100	1.64096700
F	-3.45516600	0.11755700	-0.34185800
H	-2.88040200	2.87557500	0.17909300
C	0.21530000	3.46967900	-1.28774200
H	1.25172700	3.24132800	-1.02167100
H	-0.00272900	3.02168800	-2.25842700
H	0.11179600	4.55624700	-1.36737200
C	-0.39497000	3.50440800	1.15109000
H	-0.50292100	4.59188700	1.13944800
H	-1.06053400	3.10349800	1.92318400
H	0.63952000	3.26844600	1.42152600
H	0.88783300	1.09447200	2.84358900

C	2.87970000	1.26330400	1.04079100
H	2.07963600	1.61738300	0.38339000
H	3.00387900	2.02350400	1.82497100
C	4.16682100	1.14249700	0.22842100
H	5.03243700	0.92512900	0.86082000
H	4.08043000	0.34548200	-0.51557600
H	4.37560900	2.07413900	-0.30499300
C	3.45719000	-0.61411000	2.67286400
H	3.66835700	0.10721300	3.47006100
H	3.09923500	-1.53765000	3.13697600
H	4.39624300	-0.84308300	2.16138200
H	2.28276800	-0.79489400	0.89509900

Reaction between 6' and 3-pentyl acetate – primary position

TS

C	-3.03010670	2.45008197	-1.56408997
C	-3.38246305	1.11260664	-1.67330724
C	-2.81451391	0.16182570	-0.82819738
C	-1.87466525	0.54330206	0.13777893
C	-1.54030475	1.89805884	0.24631527
C	-2.11258374	2.84311751	-0.60094550
H	-4.11144805	0.80071590	-2.41097994
H	-1.82930342	3.88448660	-0.50806546
C	-1.28703509	-0.45752580	1.11489916
O	-1.63119279	-0.41576236	2.28923763
C	0.29945018	-2.36533457	1.34479666
C	-0.71424828	-3.30746403	2.02453570
H	-1.31322579	-2.76263498	2.75461853
H	-0.17122822	-4.11041007	2.53249436
N	-0.48423846	-1.39510357	0.56834760
H	0.10582886	-0.95983524	-0.60361038
C	0.54402847	-0.60856944	-1.71810888
C	1.20829423	0.74513870	-1.66325567
H	1.47049868	1.05355081	-2.68536455
H	0.50852620	1.49639072	-1.28759169
C	-0.56030386	2.38502776	1.29212785
C	-3.23994566	-1.28293939	-1.00376299
H	-3.47492492	3.18548397	-2.22478640
F	0.46473544	1.53045691	1.46843208
F	-0.01182429	3.56134757	0.91918399
F	-1.13794642	2.57825733	2.47751761
F	-4.40489896	-1.35935839	-1.67268920
F	-2.34420992	-1.98917798	-1.71712901
F	-3.41631724	-1.91153767	0.16283867
H	-1.38057317	-3.74829779	1.27856265
C	1.20358134	-1.68744236	2.38006350
H	1.97192493	-1.08435764	1.88970682
H	0.61564269	-1.06072598	3.05230849
H	1.71700701	-2.45132219	2.97225294
C	1.14295141	-3.17734133	0.35649513
H	1.61843964	-4.00868566	0.88318260
H	0.51861724	-3.58092312	-0.44632408
H	1.93849968	-2.56407112	-0.07262015
H	1.22164990	-1.41945524	-1.99169257

H	-0.36096667	-0.63738086	-2.32516178
C	2.46990601	0.78423892	-0.81291024
C	3.02303614	2.19362445	-0.64107229
H	3.19155088	2.63141161	-1.63281383
H	2.24317971	2.78937086	-0.15662033
C	4.30371513	2.25163284	0.18688574
H	5.12455823	1.73272691	-0.31662826
H	4.61446754	3.28753434	0.34555499
H	4.16094634	1.78145783	1.16375293
H	2.27812798	0.33938047	0.16637620
O	3.45039768	-0.03262983	-1.49302330
C	4.15828712	-0.91940636	-0.77490132
O	4.02713361	-1.10222988	0.41316651
C	5.12650569	-1.66184975	-1.65771467
H	5.78209998	-0.95389419	-2.17017428
H	5.71470259	-2.34980029	-1.05300129
H	4.57700333	-2.21459823	-2.42403353

IRC start-point

C	1.84263085	-3.00087570	-1.28240833
C	2.49935448	-1.83315149	-1.64290645
C	2.36994064	-0.68141512	-0.87049043
C	1.56643222	-0.68683296	0.27826571
C	0.91990220	-1.87648965	0.63796574
C	1.06048675	-3.02402574	-0.13686456
H	3.13124826	-1.81386538	-2.52221679
H	0.54769709	-3.93137882	0.15776051
C	1.43567005	0.54353147	1.15352149
O	1.91782946	0.57212564	2.27617662
C	0.20982745	2.69428728	1.20130004
C	1.37178191	3.59057012	1.71017390
H	1.93283722	3.07702715	2.49165265
H	0.93806991	4.51068231	2.11319802
N	0.86779059	1.60472251	0.50725017
H	-0.17382677	1.18437397	-1.76844914
C	-0.68172679	0.73233313	-2.62577215
C	-1.17863634	-0.66913177	-2.28832081
H	-1.66162766	-1.12654906	-3.16049746
H	-0.33207185	-1.31179012	-2.02263879
C	0.07038270	-1.95618470	1.88795486
C	3.14578893	0.54494457	-1.31522496
H	1.94724884	-3.89349898	-1.88846434
F	-0.73957139	-0.88679075	2.00594659
F	-0.72312222	-3.04309153	1.86726482
F	0.80881808	-2.02904365	2.99719001
F	4.20849882	0.18235925	-2.05868880
F	2.41092085	1.36807230	-2.07322211
F	3.61953690	1.25059323	-0.27980259
H	2.04789581	3.84697919	0.89082370
C	-0.69058536	2.25635426	2.36204164
H	-1.51803168	1.64877522	1.98974727
H	-0.12875776	1.69630338	3.11076984
H	-1.11419602	3.14533600	2.83837587
C	-0.60009864	3.46131511	0.14875474
H	-1.02377787	4.36564706	0.59507819

H	0.03628266	3.74432220	-0.69405956
H	-1.42469274	2.84431738	-0.21503717
H	-1.51474824	1.38045177	-2.91370190
H	0.02960752	0.70795147	-3.45503110
C	-2.15642064	-0.69032033	-1.12224869
C	-2.61993404	-2.09215175	-0.74610755
H	-3.07239057	-2.56078999	-1.62854138
H	-1.72756051	-2.67735062	-0.50146448
C	-3.59202874	-2.12383844	0.42981763
H	-4.52699615	-1.61363594	0.18137301
H	-3.83722672	-3.15382060	0.70189120
H	-3.16208208	-1.63200137	1.30524638
H	-1.71574231	-0.19617255	-0.25068236
O	-3.31536364	0.07701944	-1.52815517
C	-3.77854394	1.02992447	-0.71014973
O	-3.30536863	1.30358957	0.36999278
C	-4.96655663	1.72380351	-1.32362569
H	-5.73096013	0.99070085	-1.59114157
H	-5.36892489	2.44865112	-0.61808349
H	-4.66031671	2.22963215	-2.24302199

IRC end-point

C	-1.85277440	3.05720529	-1.31265737
C	-2.53815541	1.89069677	-1.62072452
C	-2.37122689	0.75073527	-0.83723455
C	-1.50094777	0.76247062	0.26071867
C	-0.83734737	1.95416782	0.57617182
C	-1.01454960	3.09181303	-0.20666834
H	-3.21790624	1.86492211	-2.46344022
H	-0.48811714	4.00281998	0.05083227
C	-1.31622915	-0.46889381	1.13604662
O	-1.77083618	-0.49076736	2.26813200
C	-0.33754520	-2.77380916	1.11925662
C	-1.65644642	-3.48510605	1.44477174
H	-2.22246728	-2.92019320	2.18734264
H	-1.45136288	-4.48348742	1.84296355
N	-0.62861083	-1.45467536	0.52930107
H	-0.28056607	-1.27812298	-0.40702798
C	0.61848685	-0.75827473	-2.48965428
C	1.17270114	0.59910506	-2.24162604
H	1.66478462	0.98157737	-3.15273316
H	0.36686927	1.30600675	-2.01416994
C	0.05111753	2.05824932	1.79957089
C	-3.17378884	-0.48488240	-1.19439068
H	-1.98405461	3.94282054	-1.92391842
F	0.84671929	0.98523289	1.94356885
F	0.86414390	3.13220789	1.71013777
F	-0.65725836	2.19759559	2.92012834
F	-4.19699858	-0.17674502	-2.00815549
F	-2.43513530	-1.40833438	-1.84430628
F	-3.68791586	-1.08164048	-0.11478869
H	-2.26684711	-3.58668540	0.54268044
C	0.52760853	-2.60040855	2.37282574
H	1.46065514	-2.09282050	2.11640453
H	-0.00561589	-2.01506233	3.12402596

H	0.76606687	-3.58150938	2.79573221
C	0.43369409	-3.55424309	0.05208085
H	0.65390865	-4.56077805	0.41678414
H	-0.15587740	-3.64548119	-0.86717336
H	1.38252354	-3.05811924	-0.17256506
H	1.27376409	-1.62341788	-2.45995946
H	-0.36420683	-0.88300317	-2.92711876
C	2.19346431	0.64074623	-1.10602381
C	2.72381701	2.04139283	-0.83151838
H	3.16910440	2.43827741	-1.75203780
H	1.86512241	2.67741136	-0.59317857
C	3.73065020	2.09044633	0.31380251
H	4.62964234	1.51655545	0.07127428
H	4.03681795	3.11982081	0.51678703
H	3.29937023	1.67607851	1.22797726
H	1.76307161	0.21748901	-0.19475480
O	3.30110981	-0.19664974	-1.50660200
C	3.70514091	-1.16854071	-0.67266057
O	3.19401854	-1.41251800	0.39552895
C	4.87800257	-1.91133530	-1.25609118
H	5.70720267	-1.21939809	-1.42294678
H	5.18319418	-2.70213893	-0.57311750
H	4.60483381	-2.33541018	-2.22547660

Reaction between 6' and 3-pentyl acetate – secondary position

TS

C	-0.16425198	-3.44039478	0.61694368
C	0.99814855	-3.17329744	-0.09333923
C	1.57295220	-1.90603621	-0.05131807
C	0.99147829	-0.89256278	0.72174374
C	-0.14568564	-1.19201016	1.47696780
C	-0.73235355	-2.45295440	1.40774660
H	1.46155384	-3.94856723	-0.69093612
H	-1.64105137	-2.65024486	1.96196499
C	1.61037563	0.48842123	0.76997426
O	2.31432142	0.81525725	1.71560301
C	1.87731083	2.61700903	-0.48406162
C	3.41458429	2.54806763	-0.58337979
H	3.83939104	2.16468188	0.34501082
H	3.81176695	3.55063675	-0.77070489
N	1.41504072	1.22751979	-0.35032974
H	0.21961242	0.90987691	-1.00758852
C	-0.80865411	0.65238374	-1.68371098
C	-0.64450178	-0.73207536	-2.26971121
H	-1.37387844	-0.87549885	-3.07518887
H	0.35209019	-0.86742326	-2.69013718
H	-0.82110775	-1.50620024	-1.51937733
C	-2.02110864	0.83179432	-0.78946183
H	-1.94430113	0.17190100	0.07530282
C	-0.72965693	-0.19424328	2.45229626
C	2.82582382	-1.66220871	-0.86828050
H	-0.62342404	-4.42049719	0.56003084
F	-0.63181277	1.07844938	2.02023282
F	-2.03820691	-0.42611004	2.66145294

F	-0.11943936	-0.25794142	3.63991637
F	3.41421334	-2.82481116	-1.20072954
F	2.57263280	-1.01864798	-2.02414480
F	3.73278359	-0.93546685	-0.20580122
H	3.70964473	1.88993026	-1.40438101
C	1.45209720	3.51302667	0.68645320
H	0.36297851	3.57558823	0.75884239
H	1.84254444	3.12991270	1.62906898
H	1.83544612	4.52584886	0.52784721
C	1.32338668	3.17919326	-1.79730250
H	1.78523631	4.14920545	-1.99888444
H	1.54457226	2.50917335	-2.63329057
H	0.24162177	3.33054143	-1.73999282
H	-0.75323919	1.43043696	-2.45107202
C	-2.25993028	2.26620169	-0.35101666
H	-1.33760780	2.62067467	0.11679044
H	-2.42882877	2.88455850	-1.24110228
C	-3.42209033	2.40169036	0.62753342
H	-4.35776469	2.06680021	0.17112456
H	-3.24292417	1.79856319	1.52167016
H	-3.55248438	3.44117121	0.93892871
O	-3.18862072	0.40869175	-1.53681576
C	-3.76117186	-0.76240886	-1.20228507
O	-3.35885479	-1.50043020	-0.33390375
C	-4.96918447	-1.01974239	-2.06379740
H	-5.68815885	-0.20511491	-1.94828130
H	-4.67317017	-1.04946870	-3.11540908
H	-5.42551394	-1.96564358	-1.77729896

IRC start-point

C	1.43848337	-0.37764373	-2.67395610
C	0.61185130	-1.43198585	-2.31974024
C	-0.54300885	-1.20594393	-1.57291428
C	-0.88745195	0.09407712	-1.18049019
C	-0.06796989	1.15707425	-1.58782105
C	1.08928327	0.91814533	-2.32103801
H	0.85856238	-2.44250289	-2.62230031
H	1.73017231	1.74596443	-2.59502390
C	-2.10307914	0.35475004	-0.31793323
O	-3.08062318	0.96495408	-0.72069530
C	-2.70050741	0.19594282	2.08689888
C	-4.15426048	-0.32473624	1.93542169
H	-4.65559945	0.18918584	1.11450362
H	-4.68796062	-0.13196932	2.87102672
N	-2.01377423	-0.27003310	0.89878782
H	0.23330691	-1.55201516	1.44907299
C	1.30687697	-1.61296115	1.65633910
C	1.89537111	-2.85095117	0.98747602
H	2.95356817	-2.96635216	1.23819913
H	1.36905807	-3.75482827	1.30306992
H	1.81400222	-2.77889608	-0.10170706
C	1.93623702	-0.31721500	1.17002723
H	1.84244912	-0.24175192	0.08401914
C	-0.43198999	2.59039232	-1.26856296
C	-1.40371391	-2.41028407	-1.24260144

H	2.35786949	-0.56087467	-3.21575307
F	-0.79036015	2.74409322	0.02211411
F	0.60407708	3.41880543	-1.48257481
F	-1.44725801	3.02559938	-2.01977108
F	-1.23908778	-3.37175006	-2.16932256
F	-1.09086699	-2.96312619	-0.06069037
F	-2.70996702	-2.11222909	-1.21776154
H	-4.15754143	-1.39978531	1.74053659
C	-2.69172448	1.71771092	2.27376101
H	-1.66981742	2.08446797	2.40357915
H	-3.14294644	2.22562801	1.42074494
H	-3.25863761	1.97339585	3.17347304
C	-2.03130646	-0.50032130	3.27888698
H	-2.56878360	-0.26151637	4.20110601
H	-2.03026141	-1.58416637	3.13803889
H	-0.99555240	-0.16658125	3.38799912
H	1.42102887	-1.67976023	2.74554519
C	1.33808588	0.92275902	1.82092031
H	0.28147083	0.95513001	1.53098419
H	1.36553577	0.80760293	2.91192138
C	2.02664193	2.22113517	1.41271245
H	3.04932996	2.25594317	1.79832058
H	2.07767404	2.31655926	0.32513339
H	1.48869882	3.08871019	1.80221850
O	3.34959209	-0.36596799	1.47826839
C	4.22679925	-0.15182487	0.48636242
O	3.92889333	0.08840323	-0.66161567
C	5.63863029	-0.25069688	1.00374859
H	5.79760921	0.49481512	1.78691072
H	5.80285929	-1.23459297	1.44984456
H	6.33910544	-0.08776378	0.18643561

IRC end-point

C	-0.63755208	-3.20042305	0.96061716
C	0.52312125	-3.17331566	0.20168401
C	1.28969597	-2.01235183	0.13354589
C	0.91143888	-0.86727348	0.84533534
C	-0.25100754	-0.91661913	1.62172302
C	-1.02788587	-2.07075424	1.66511153
H	0.83544642	-4.05163458	-0.34967392
H	-1.94885007	-2.07373996	2.23341704
C	1.76765491	0.39016007	0.82234513
O	2.45446738	0.69308789	1.78333953
C	2.44026860	2.28167064	-0.68085499
C	3.93673615	1.94554731	-0.67298883
H	4.25047375	1.63288841	0.32437601
H	4.51965917	2.82372036	-0.96693207
N	1.66585646	1.07949107	-0.33362512
H	1.03576606	0.70667134	-1.03298223
C	-1.04179654	0.23197663	-2.06587043
C	-1.04897838	-1.22424359	-2.37239891
H	-1.77438739	-1.45219987	-3.16875735
H	-0.07158931	-1.56769027	-2.72080193
H	-1.33943850	-1.80794137	-1.49445872
C	-1.98706008	0.79742121	-1.06163331

H	-1.87871108	0.27013548	-0.10915833
C	-0.68354467	0.26149062	2.46737764
C	2.51022000	-2.01087776	-0.75903012
H	-1.24460116	-4.09750911	0.99591520
F	-0.44106897	1.44191304	1.86749897
F	-2.00780071	0.22185318	2.70690807
F	-0.06556472	0.27703465	3.65140239
F	2.87695369	-3.25731585	-1.09399052
F	2.27789089	-1.35528970	-1.92062013
F	3.56730338	-1.42388830	-0.18683168
H	4.14657139	1.13383264	-1.37487663
C	2.12855510	3.40947530	0.31027740
H	1.06235244	3.65507802	0.29244115
H	2.39916793	3.10989992	1.32376931
H	2.69376893	4.30752199	0.04264528
C	2.00000094	2.68085968	-2.09167418
H	2.55805326	3.56051965	-2.42185818
H	2.19038743	1.87033785	-2.80374046
H	0.93319645	2.92820378	-2.11658379
H	-0.57900363	0.91681751	-2.77587198
C	-1.84538798	2.29693325	-0.85329594
H	-0.82155273	2.46941491	-0.50427534
H	-1.94972988	2.80475110	-1.81997899
C	-2.84057770	2.86079791	0.15578137
H	-3.86700326	2.73621056	-0.19892694
H	-2.74633353	2.35224207	1.11854133
H	-2.66564798	3.92730599	0.31883772
O	-3.35500493	0.54338952	-1.51338635
C	-4.03690677	-0.44849731	-0.91975481
O	-3.58752152	-1.18025299	-0.06709257
C	-5.44196628	-0.51931069	-1.45902150
H	-5.97641408	0.40083761	-1.20900176
H	-5.42157697	-0.60174082	-2.54804971
H	-5.95611590	-1.37492465	-1.02448070

Reaction between 3' and 3-pentyl acetate – primary position

TS

C	1.94824818	-1.30393669	0.81079544
C	1.10387002	-0.39825878	1.44318204
C	-0.27347681	-0.50808563	1.30275982
C	-0.81592367	-1.51173890	0.50354629
C	0.02394065	-2.43139351	-0.11727914
C	1.40185059	-2.31877341	0.03279391
C	-2.29908478	-1.62975769	0.28080492
O	-2.81361566	-2.72822545	0.11125982
C	-4.39791171	-0.32326538	-0.00819694
C	-5.20910249	-1.11938236	1.03134382
H	-4.98930379	-2.18458691	0.96008780
H	-6.27637847	-0.96056006	0.84917373
N	-2.97553575	-0.43657345	0.35138628
H	-2.28689962	0.47388141	-0.35486866
C	-1.58896623	1.20256719	-1.11766119
C	-1.17356425	2.40643576	-0.30322837
H	-1.99619772	3.12710466	-0.24918590

H	-0.93999514	2.11339919	0.72471578
H	3.02320412	-1.21795861	0.91797589
H	-4.97534532	-0.77376793	2.04219427
C	-4.68613391	-0.83094607	-1.42689157
H	-4.10278057	-0.26751412	-2.16241489
H	-4.43779214	-1.88935183	-1.51655913
H	-5.74606989	-0.69757027	-1.66365474
C	-4.76472755	1.16099175	0.11077313
H	-5.83941670	1.29318154	-0.04102629
H	-4.50171291	1.54354767	1.10100906
H	-4.24511502	1.76176812	-0.64203236
H	-0.41154270	-3.22354736	-0.71620823
H	-0.93725925	0.18526331	1.80436903
C	2.31405850	-3.24751275	-0.72292887
C	1.69504863	0.67438252	2.31779835
F	0.85847528	1.71646502	2.46975982
F	1.97049905	0.21026828	3.54735820
F	2.84619046	1.14826305	1.81180600
F	3.49461186	-3.40115165	-0.10456506
F	2.57547768	-2.76602144	-1.95611252
F	1.77188355	-4.46111483	-0.88291712
H	-2.21241299	1.42272475	-1.98556170
H	-0.77054098	0.53508499	-1.38836966
C	0.06254638	3.11839230	-0.85998529
H	-0.09407510	3.38794389	-1.90862589
C	0.44470018	4.34360463	-0.03816603
H	-0.41877372	5.01847979	-0.01973508
H	0.62765106	4.02618836	0.99438313
C	1.66728140	5.07177419	-0.58943831
H	1.89650089	5.95731639	0.00881101
H	2.54431438	4.41953790	-0.57535941
H	1.50193340	5.39511112	-1.62227565
O	1.18077543	2.20903422	-0.79968270
C	1.62551017	1.67250659	-1.95290233
O	1.09640552	1.84063895	-3.02611876
C	2.85440201	0.83273090	-1.72793710
H	3.23027359	0.94114477	-0.71192273
H	2.61172365	-0.21490158	-1.92073543
H	3.61660606	1.13012956	-2.45061430

IRC start-point

C	-1.07079302	1.61914833	0.96413801
C	-0.46451450	0.51447443	1.54779217
C	0.88110752	0.25012184	1.32208842
C	1.62500949	1.09947113	0.50545240
C	1.02541897	2.21437979	-0.07547478
C	-0.32260889	2.46281823	0.14849653
C	3.06410024	0.82816103	0.20321077
O	3.83009150	1.70651214	-0.17352157
C	4.45268922	-1.15952350	-0.32973846
C	5.80859668	-0.89839576	0.36776785
H	6.06400503	0.16129854	0.30745837
H	6.58260196	-1.48426526	-0.13739625
N	3.46790097	-0.45753468	0.47461853
H	1.50418023	-1.02961397	-1.69901780

C	0.42920216	-1.18037187	-1.83120572
C	-0.07354501	-2.30681014	-0.93535791
H	0.42854510	-3.24741367	-1.19157455
H	0.16889225	-2.09530072	0.11126123
H	-2.11936875	1.82338317	1.14194633
H	5.76731106	-1.19774780	1.41758270
C	4.52376739	-0.72761283	-1.80043520
H	3.56587074	-0.88862259	-2.30410039
H	4.79672269	0.32378354	-1.89362863
H	5.27554443	-1.33120530	-2.31670268
C	4.09160008	-2.64949043	-0.22513502
H	4.84728056	-3.25098773	-0.73829050
H	4.04048037	-2.96157691	0.82079387
H	3.12158937	-2.84418027	-0.69292240
H	1.62159442	2.86989513	-0.70047841
H	1.36024674	-0.61094411	1.77308981
C	-1.00108726	3.60941894	-0.55239159
C	-1.25182769	-0.36372429	2.48344822
F	-0.80522645	-1.63312354	2.46305113
F	-1.14716656	0.06451978	3.75268269
F	-2.55734949	-0.38462224	2.18055248
F	-2.04301385	4.07735695	0.15121013
F	-1.47968853	3.22247537	-1.75310962
F	-0.16157266	4.62818720	-0.77392499
H	0.24269878	-1.39843818	-2.88736196
H	-0.07008353	-0.23864953	-1.58647056
C	-1.57506793	-2.53696732	-1.05290308
H	-1.84034010	-2.77974682	-2.08638994
C	-2.09182488	-3.61958973	-0.11242037
H	-1.56517439	-4.54902679	-0.35993969
H	-1.81139730	-3.35648939	0.91286987
C	-3.60043266	-3.83395409	-0.20298240
H	-3.91318782	-4.66859342	0.42996754
H	-4.13917365	-2.94320847	0.13228235
H	-3.91084085	-4.04582205	-1.23033519
O	-2.23023063	-1.29210858	-0.72070721
C	-3.13726320	-0.80596776	-1.58978617
O	-3.43751633	-1.33674477	-2.63208479
C	-3.70421599	0.49826089	-1.09590960
H	-3.85064409	0.46686430	-0.01539027
H	-3.00068720	1.30274953	-1.32757912
H	-4.64443570	0.69910828	-1.60757580

IRC end-point

C	-1.34121800	1.74943700	0.85545500
C	-0.78834000	0.61315200	1.43509700
C	0.55628200	0.31467900	1.25268400
C	1.35795300	1.14763300	0.47324600
C	0.81161400	2.29514100	-0.09286300
C	-0.53599800	2.58530200	0.09113600
C	2.82357700	0.87644500	0.23495400
O	3.60575600	1.80471800	0.08456100
C	4.50200300	-0.95816900	-0.10111200
C	5.50913700	-0.43870600	0.93035900
H	5.58125600	0.64853300	0.88446900

H	6.49549900	-0.86768600	0.72986200
N	3.15759300	-0.44013100	0.21209900
H	2.39776700	-1.08485900	0.02318100
C	0.84535300	-1.97243900	-1.44368000
C	0.08032400	-2.82549700	-0.49333400
H	0.49918800	-3.83638600	-0.45690900
H	0.12815500	-2.41712100	0.52460700
H	-2.38918700	1.98408600	0.99976100
H	5.20434200	-0.72944500	1.94001100
C	4.91173700	-0.54488200	-1.52112000
H	4.18941600	-0.92298000	-2.25189000
H	4.95718200	0.54265600	-1.60147100
H	5.89560800	-0.95674200	-1.76562800
C	4.39825600	-2.48325100	-0.00507500
H	5.36680000	-2.93953500	-0.22442100
H	4.09278200	-2.79106500	0.99995700
H	3.67021700	-2.87361800	-0.72555200
H	1.45521900	2.95196600	-0.66764600
H	0.98475100	-0.55737000	1.73345600
C	-1.13752500	3.77626900	-0.60641600
C	-1.65263400	-0.24784400	2.31635300
F	-1.10837300	-1.46325600	2.51961600
F	-1.82548700	0.31013500	3.52548900
F	-2.87350500	-0.43200400	1.79191700
F	-2.27740700	4.17640400	-0.02364300
F	-1.43101700	3.48129200	-1.88947400
F	-0.29734700	4.81846900	-0.62669000
H	1.43301300	-2.41327700	-2.24168200
H	0.59942500	-0.91849600	-1.53339200
C	-1.41331300	-2.94317900	-0.84712900
H	-1.52433600	-3.34521300	-1.85779100
C	-2.18926000	-3.78609600	0.15720700
H	-1.73587600	-4.78406900	0.17582000
H	-2.05952900	-3.35479900	1.15497700
C	-3.67439400	-3.88961400	-0.17842400
H	-4.19029600	-4.53943200	0.53311200
H	-4.14678200	-2.90473400	-0.13830400
H	-3.82634500	-4.29849300	-1.18254700
O	-1.98339000	-1.62124700	-0.83244700
C	-2.33072500	-1.07030400	-2.01325400
O	-2.14253500	-1.59067900	-3.08632200
C	-2.96320900	0.28038600	-1.81257400
H	-3.41008400	0.35956400	-0.82170500
H	-2.19913200	1.05531600	-1.91556900
H	-3.71034500	0.44163400	-2.58971400

Reaction between 3' and 3-pentyl acetate – secondary position

TS

C	-2.32674177	0.49446523	-1.02869575
C	-1.36207284	1.48937400	-0.94471247
C	-0.01281858	1.17718478	-1.07234161
C	0.37288538	-0.13794053	-1.30419763
C	-0.59033943	-1.13742061	-1.41025426
C	-1.93182371	-0.82157665	-1.25045945

C	1.81687041	-0.51479056	-1.46281051
O	2.13475416	-1.44891146	-2.19027714
C	4.13375309	0.06129877	-0.78181929
C	4.62835778	0.18182715	-2.23737770
H	4.24076736	-0.63073675	-2.85089241
H	5.72200285	0.14689868	-2.24396607
N	2.68241216	0.30612004	-0.78130657
H	2.21763116	0.36298920	0.51003683
C	1.96380585	0.37362981	1.75054073
C	1.56425351	1.79772172	2.06200033
H	1.40787864	1.91565966	3.14113791
H	2.34113915	2.49855991	1.74703757
H	0.62958005	2.06538970	1.56451850
C	0.97585489	-0.70380470	2.15301767
H	-3.37689821	0.73948310	-0.92420754
H	4.31184292	1.13604524	-2.66804244
C	4.50533767	-1.31009625	-0.20262802
H	4.18931038	-1.38991635	0.84216337
H	4.03257621	-2.10958441	-0.77531138
H	5.59021126	-1.44879023	-0.23549226
C	4.78023316	1.18717167	0.03529779
H	5.86956695	1.10714086	-0.01851488
H	4.48279245	2.16290241	-0.35874087
H	4.49798313	1.14238370	1.09033589
H	-0.27467044	-2.15530250	-1.61107148
H	0.74540960	1.94890075	-1.00361692
C	-2.96022871	-1.91822089	-1.23335589
C	-1.75941081	2.90736283	-0.64325586
F	-1.60296062	3.18175247	0.67354231
F	-1.00676035	3.79153899	-1.31120828
F	-3.04345552	3.14965119	-0.93959214
F	-3.12362302	-2.40060225	0.02014014
F	-2.60829291	-2.95343810	-2.00535223
F	-4.16279229	-1.48750101	-1.64175356
H	2.94530089	0.11564903	2.16041343
C	1.25867306	-2.08700620	1.57032525
H	2.22469119	-2.41489631	1.97187890
H	1.38302016	-2.03064570	0.48577003
C	0.17206918	-3.10280608	1.91145576
H	-0.77098321	-2.83758409	1.42661817
H	0.45742309	-4.10166136	1.57233031
H	-0.00360644	-3.14779546	2.99116518
H	1.01375910	-0.77096954	3.24789121
O	-0.36458870	-0.31156304	1.81309340
C	-1.19711181	-0.02816939	2.84147961
O	-0.85862508	-0.02461192	4.00066643
C	-2.57930673	0.28855649	2.34682485
H	-2.55716318	1.24185029	1.81414558
H	-2.91647682	-0.48237456	1.65203826
H	-3.25477165	0.36108048	3.19747211

IRC start-point

C	-1.99209692	0.26575769	-1.36303592
C	-0.99510345	1.23367382	-1.39496697
C	0.34430158	0.86705469	-1.36450228

C	0.68719862	-0.48216626	-1.30557272
C	-0.30731989	-1.45599373	-1.26080650
C	-1.64241591	-1.07763746	-1.28148919
C	2.11424755	-0.93142128	-1.28734840
O	2.42543632	-2.09813834	-1.50076873
C	4.30975910	-0.13892645	-0.45544923
C	5.27837601	-0.61935878	-1.56470044
H	4.97085728	-1.59864892	-1.93480597
H	6.28427501	-0.69143474	-1.13998053
N	3.03821488	0.07397493	-1.12324120
H	1.69833674	0.74804388	1.05228984
C	1.42423314	0.94420662	2.09589300
C	0.95398876	2.38806718	2.24014905
H	0.62548693	2.59528625	3.26328020
H	1.75795495	3.08686690	1.99245140
H	0.11465118	2.58934149	1.57044315
C	0.39247779	-0.08313650	2.54933900
H	-3.03546000	0.55532008	-1.40852062
H	5.29677734	0.08993064	-2.39550607
C	4.27351209	-1.15444722	0.69421055
H	3.57848504	-0.82964866	1.47279702
H	3.97317839	-2.14249938	0.34394649
H	5.26877845	-1.22977013	1.14124606
C	4.75847220	1.23701653	0.05808948
H	5.75867653	1.16252448	0.49425953
H	4.78163494	1.96392618	-0.75751098
H	4.07435254	1.60264784	0.82949869
H	-0.01914410	-2.50046927	-1.21894719
H	1.12524973	1.61711681	-1.40760172
C	-2.72200863	-2.12018419	-1.17091019
C	-1.38088212	2.68766724	-1.43157888
F	-1.64814828	3.15592947	-0.19209629
F	-0.40629956	3.45464955	-1.93571330
F	-2.48627754	2.88860467	-2.16412173
F	-3.07053488	-2.32523494	0.11870292
F	-2.33101394	-3.30212295	-1.66168500
F	-3.83762659	-1.75148504	-1.81783918
H	2.32993169	0.78104586	2.69332664
C	0.80344761	-1.52337208	2.26116409
H	1.69322284	-1.73622666	2.86607594
H	1.11204914	-1.61696854	1.21413087
C	-0.29529953	-2.53729875	2.56640467
H	-1.14278189	-2.40372068	1.88952240
H	0.07389377	-3.55919870	2.44682380
H	-0.66218416	-2.42494462	3.59151419
H	0.20574677	0.03644050	3.62027575
O	-0.85363830	0.18460967	1.87059109
C	-1.91709995	0.50692182	2.63734555
O	-1.88615074	0.59720328	3.84122094
C	-3.13913161	0.73693571	1.79191939
H	-2.94935767	1.55320878	1.09225707
H	-3.36003300	-0.16383685	1.21591817
H	-3.98009232	0.98522771	2.43700672

IRC end-point

C	-2.16615939	0.17818334	-1.24758432
C	-1.23201160	1.20115480	-1.33645112
C	0.12959734	0.91538715	-1.34383085
C	0.56147758	-0.40511819	-1.27495084
C	-0.37276778	-1.43504549	-1.19810046
C	-1.72789332	-1.13957581	-1.16541210
C	2.01964714	-0.78592775	-1.31197700
O	2.36947303	-1.84950064	-1.80470158
C	4.30665576	-0.05263297	-0.59004712
C	4.96466322	-0.28158056	-1.95506338
H	4.59482392	-1.19921659	-2.41339075
H	6.04898883	-0.35854020	-1.83101823
N	2.85495768	0.13538265	-0.76293630
H	2.41961547	0.76534698	-0.09869045
C	1.54668936	0.93751757	2.12451872
C	1.08833122	2.34272975	1.93972050
H	0.75446046	2.77679687	2.89496308
H	1.88723274	2.97860227	1.54802215
H	0.23161202	2.39970917	1.26090438
C	0.60315272	-0.14991240	2.52313323
H	-3.22617331	0.40258590	-1.25150839
H	4.75210180	0.55701316	-2.62464214
C	4.57771123	-1.23382175	0.35169965
H	4.11847293	-1.05629906	1.32974826
H	4.16486585	-2.15367339	-0.06709613
H	5.65425689	-1.36540535	0.49657423
C	4.83484232	1.24756152	0.02342575
H	5.91576719	1.18018699	0.17005430
H	4.62891430	2.09921067	-0.63222338
H	4.37769884	1.44211308	1.00022347
H	-0.02010655	-2.45989457	-1.17049186
H	0.84818979	1.72218716	-1.43871011
C	-2.73488398	-2.24214773	-0.98296933
C	-1.69199939	2.63221676	-1.37688920
F	-1.77503549	3.15165476	-0.12957058
F	-0.84608157	3.41257117	-2.06284931
F	-2.90737608	2.75552580	-1.92694384
F	-3.01274527	-2.42943259	0.32731598
F	-2.29495720	-3.41228258	-1.45936302
F	-3.89897389	-1.96021969	-1.58703875
H	2.59033356	0.75404932	2.37455470
C	1.08271329	-1.55023140	2.13989822
H	2.01714521	-1.72996276	2.68476029
H	1.33697989	-1.57730616	1.07728772
C	0.06487838	-2.63925977	2.46174539
H	-0.84359850	-2.50811461	1.86910780
H	0.47492814	-3.62815310	2.24150886
H	-0.21669090	-2.61792838	3.51977744
H	0.45753253	-0.11207659	3.61365601
O	-0.69326291	0.06536282	1.92938596
C	-1.63859667	0.61568449	2.72095792
O	-1.43366279	0.99729798	3.84858067
C	-2.95878936	0.70517665	2.00817571
H	-2.90478015	1.50711765	1.26815700
H	-3.17475035	-0.22887101	1.48793574
H	-3.73992594	0.93506153	2.73101916

References

1 R. K. Quinn, Z. A. Könst, S. E. Michalak, Y. Schmidt, A. R. Szklarski, A. R. Flores, S. Nam, D. A. Horne, C. D. Vanderwal and E. J. Alexanian, *J. Am. Chem. Soc.*, 2016, **138**, 696–702.
2 V. A. Schmidt, R. K. Quinn, A. T. Brusoe and E. J. Alexanian, *J. Am. Chem. Soc.*, 2014, **136**, 14389–14392.
3 B. T. Sargent and E. J. Alexanian, *J. Am. Chem. Soc.*, 2016, **138**, 7520–7523.
4 L. Jiao, E. Herdtweck and T. Bach, *J. Am. Chem. Soc.*, 2012, **134**, 14563–14572.
5 S. R. Hitchcock, F. Perron, V. A. Martin and K. F. Albizati, *Synthesis*, 1990, **1990**, 1059–1061.
6 U. S. Patent 20150299217, 2013.
7 K. Zhang, B. M. Shafer, M. D. Demars, H. A. Stern and R. Fasan, *J. Am. Chem. Soc.*, 2012, **134**, 18695–18704.
8 Bruker-AXS Inc. (2014), Madison Wisconsin, USA.
9 Sheldrick, G. M. (2008). Acta Cryst. A64, 112-122.
10 Gaussian 16, Revision A.03, Frisch, M. J.; Trucks, G. W.; Schlegel, H. B.; Scuseria, G. E.; Robb, M. A.; Cheeseman, J. R.; Scalmani, G.; Barone, V.; Petersson, G. A.; Nakatsuji, H.; Li, X.; Caricato, M.; Marenich, A. V.; Bloino, J.; Janesko, B. G.; Gomperts, R.; Mennucci, B.; Hratchian, H. P.; Ortiz, J. V.; Izmaylov, A. F.; Sonnenberg, J. L.; Williams-Young, D.; Ding, F.; Lipparini, F.; Egidi, F.; Goings, J.; Peng, B.; Petrone, A.; Henderson, T.; Ranasinghe, D.; Zakrzewski, V. G.; Gao, J.; Rega, N.; Zheng, G.; Liang, W.; Hada, M.; Ehara, M.; Toyota, K.; Fukuda, R.; Hasegawa, J.; Ishida, M.; Nakajima, T.; Honda, Y.; Kitao, O.; Nakai, H.; Vreven, T.; Throssell, K.; Mont-gomery, J. A., Jr.; Peralta, J. E.; Ogliaro, F.; Bearpark, M. J.; Heyd, J. J.; Brothers, E. N.; Kudin, K. N.; Staroverov, V. N.; Keith, T. A.; Koba-yashi, R.; Normand, J.; Raghavachari, K.; Rendell, A. P.; Burant, J. C.; Iyengar, S. S.; Tomasi, J.; Cossi, M.; Millam, J. M.; Klene, M.; Adamo, C.; Cammi, R.; Ochterski, J. W.; Martin, R. L.; Morokuma, K.; Farkas, O.; Foresman, J. B.; Fox, D. J. Gaussian, Inc., Wallingford CT, 2016.

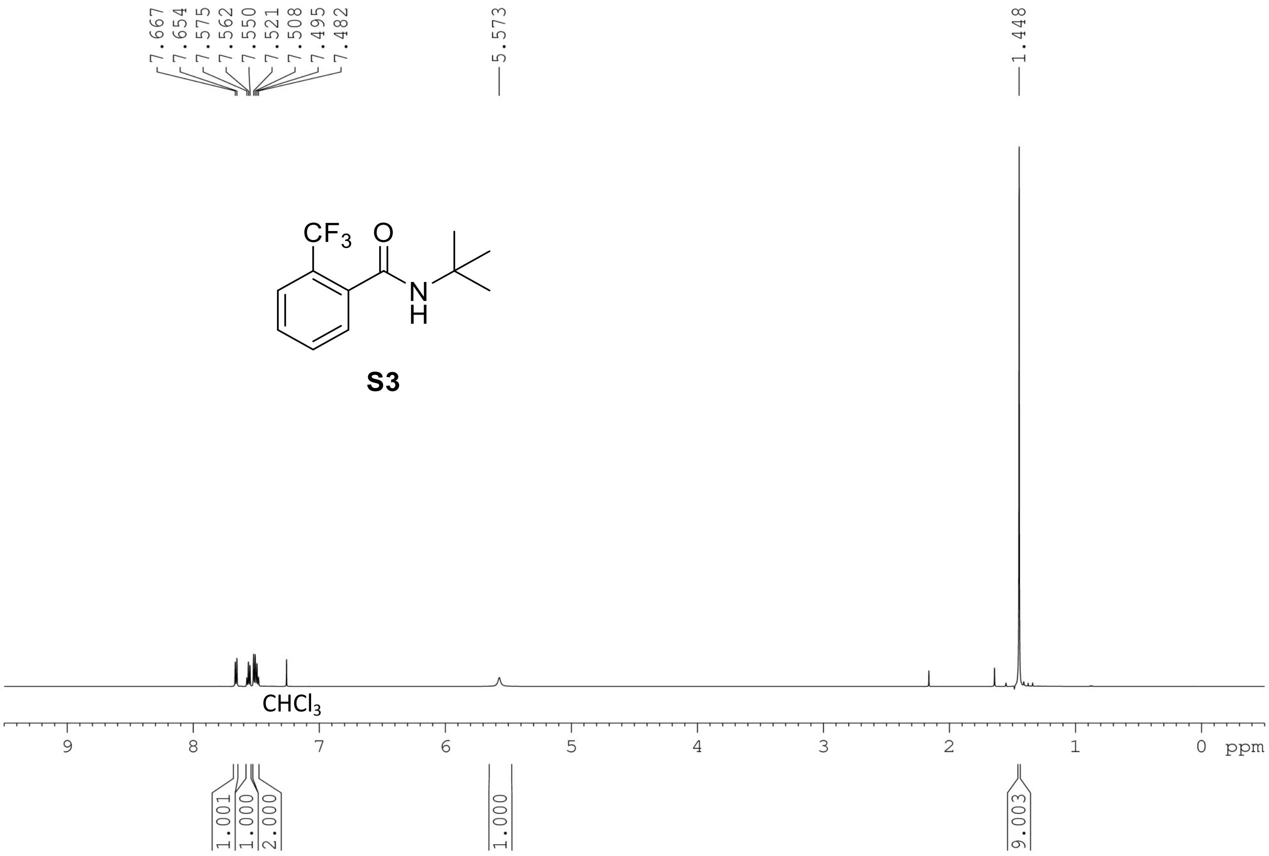
CF3
O
N
H
S3
7.667
7.654
7.575
7.562
7.550
7.521
7.508
7.495
7.482
5.573
1.448
CHCl3
9 8 7 6 5 4 3 2 1 0 ppm
1.001
1.000
2.000
1.000
9.003

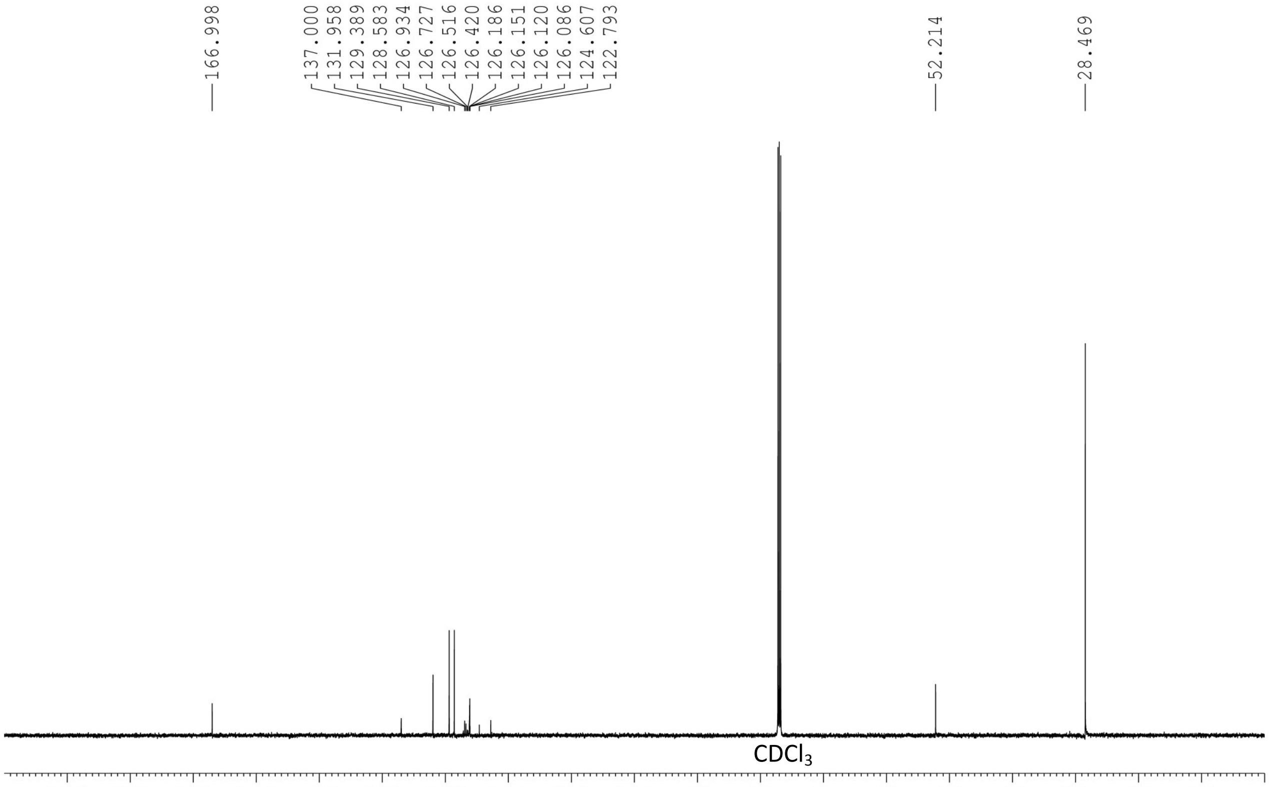
166.998
137.000
131.958
129.389
128.583
126.934
126.727
126.516
126.420
126.186
126.151
126.120
126.086
124.607
122.793
52.214
28.469
CDCl3
190 180 170 160 150 140 130 120 110 100 90 80 70 60 50 40 30 20 10 ppm

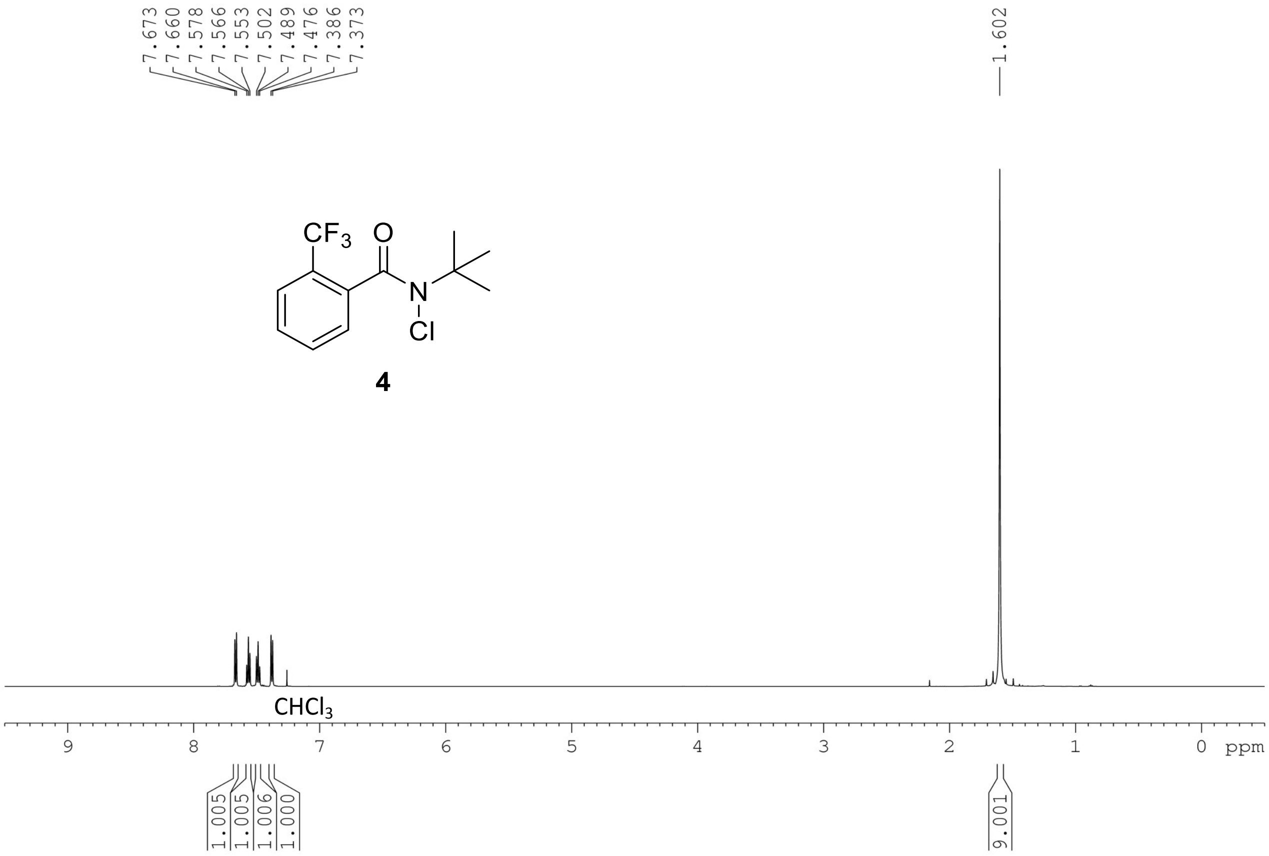
7.673
7.660
7.578
7.566
7.553
7.502
7.489
7.476
7.386
7.373
1.602
CF3
O
N
Cl
4
CHCl3
9 8 7 6 5 4 3 2 1 0 ppm
1.005
1.005
1.006
1.000
9.001

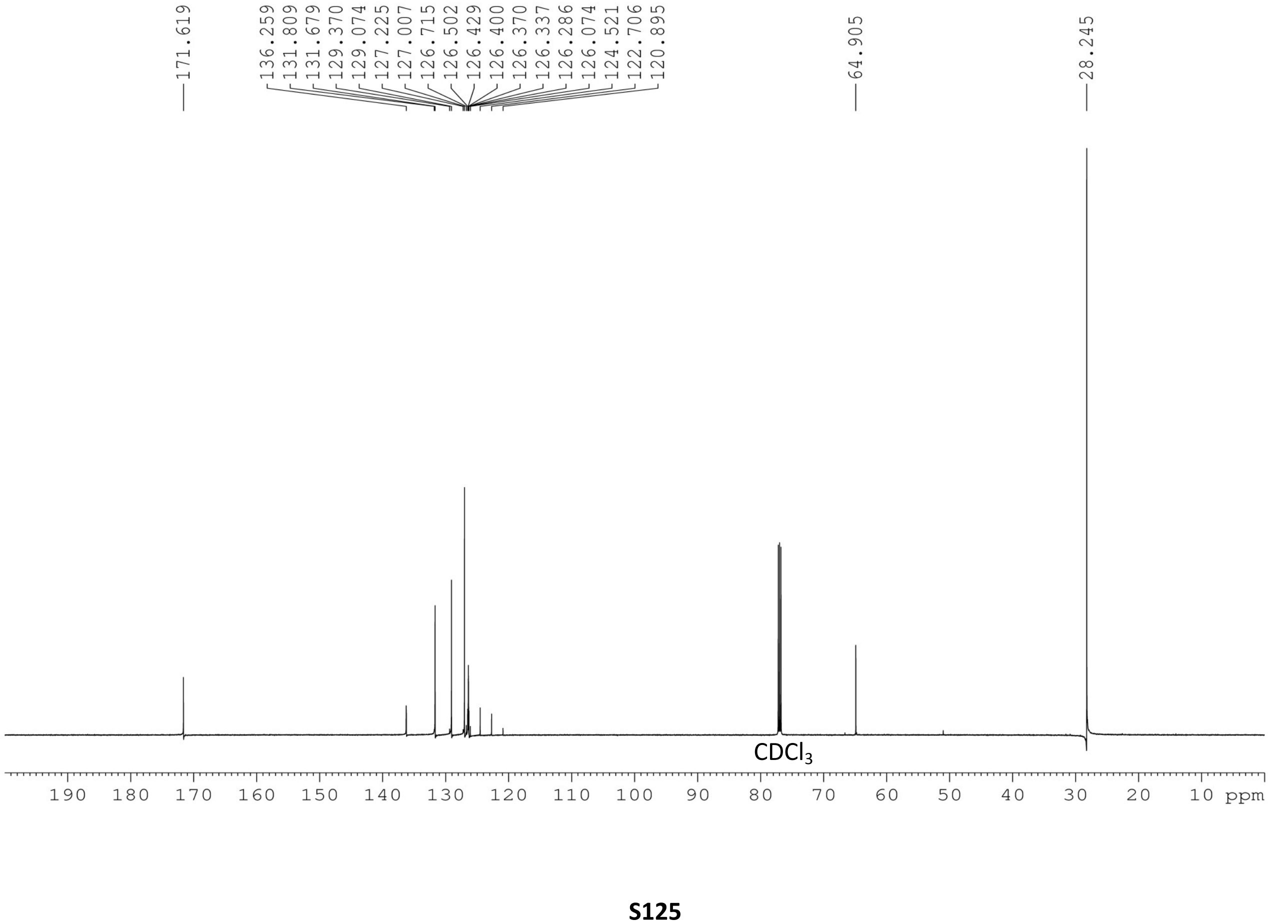
171.619
136.259
131.809
131.679
129.370
129.074
127.225
127.007
126.715
126.502
126.429
126.400
126.370
126.337
126.286
126.074
124.521
122.706
120.895
64.905
28.245
CDCl3
190 180 170 160 150 140 130 120 110 100 90 80 70 60 50 40 30 20 10 ppm

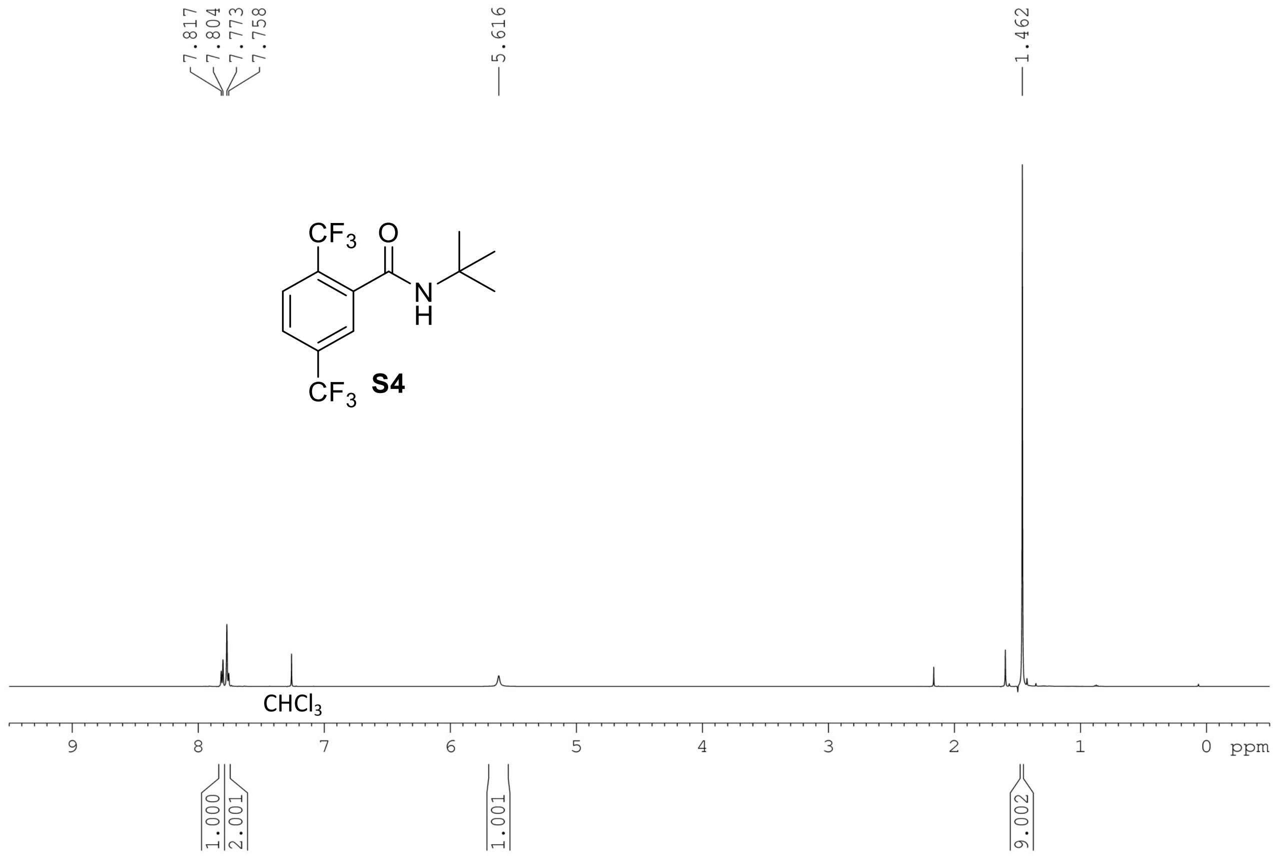
7.817
7.804
7.773
7.758
5.616
1.462
CF3
O
N
H
CF3
S4
CHCl3
9 8 7 6 5 4 3 2 1 0 ppm
1.000
2.001
1.001
9.002

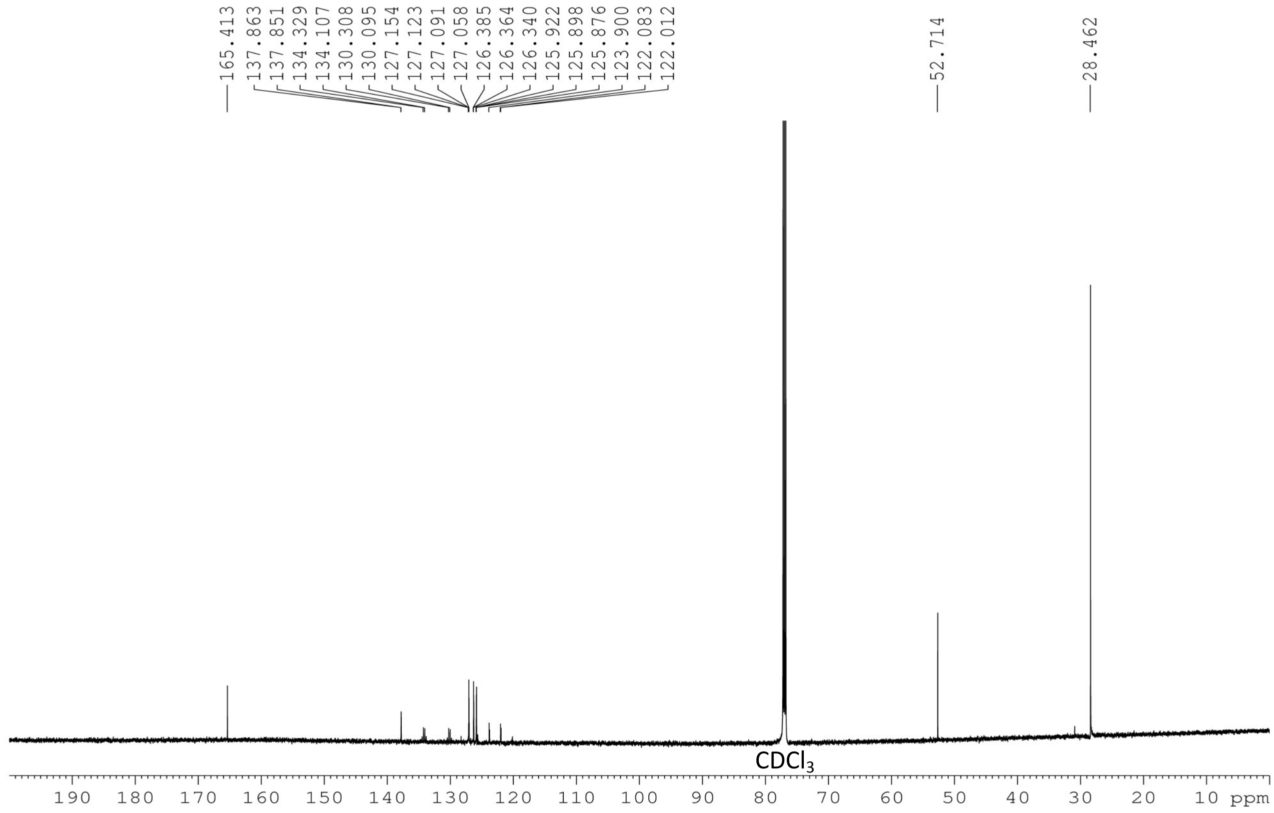
165.413
137.863
137.851
134.329
134.107
130.308
130.095
127.154
127.123
127.091
127.058
126.385
126.364
126.340
125.922
125.898
125.876
123.900
122.083
122.012
52.714
28.462
CDCl3
190 180 170 160 150 140 130 120 110 100 90 80 70 60 50 40 30 20 10 ppm

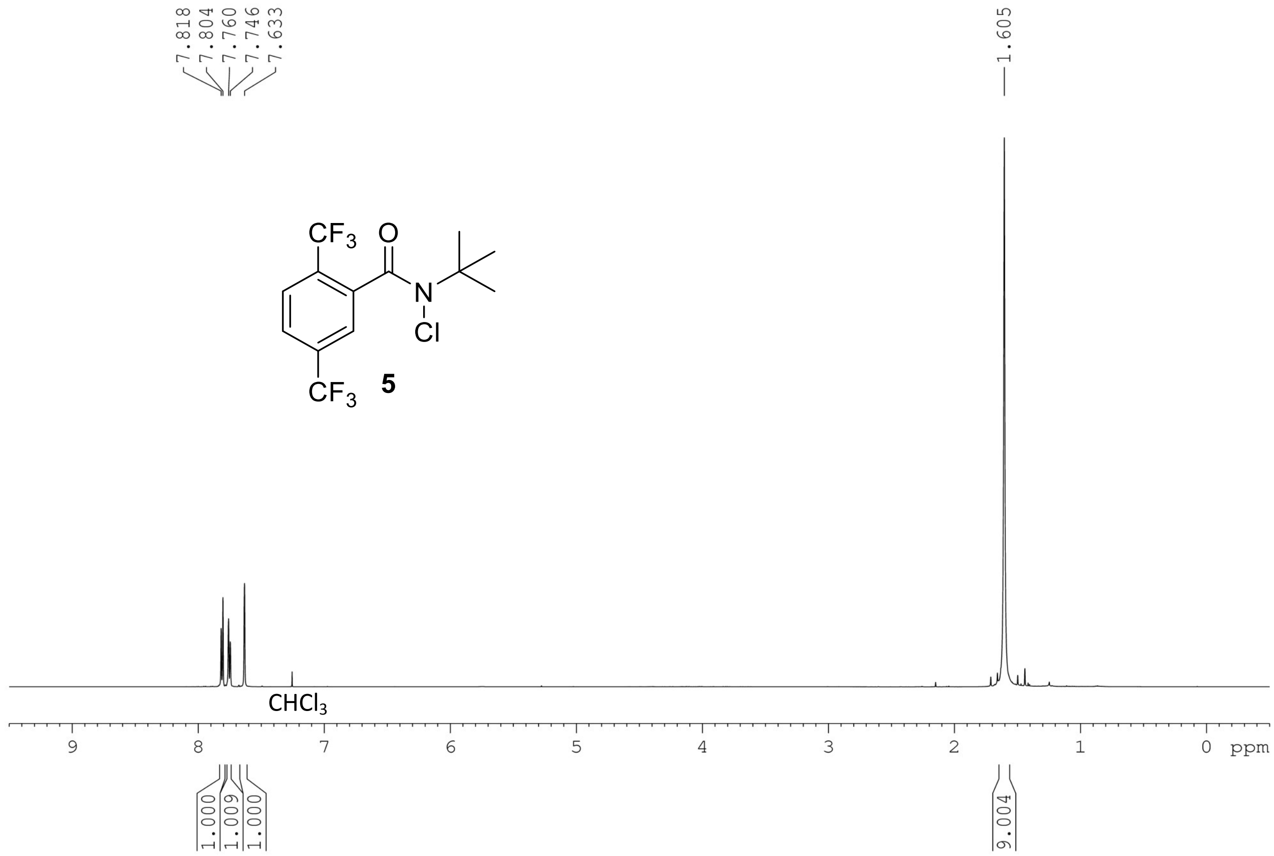
7.818
7.804
7.760
7.746
7.633
1.605
CF3
O
N
Cl
CF3
5
CHCl3
9 8 7 6 5 4 3 2 1 0 ppm
1.000
1.009
1.000
9.004

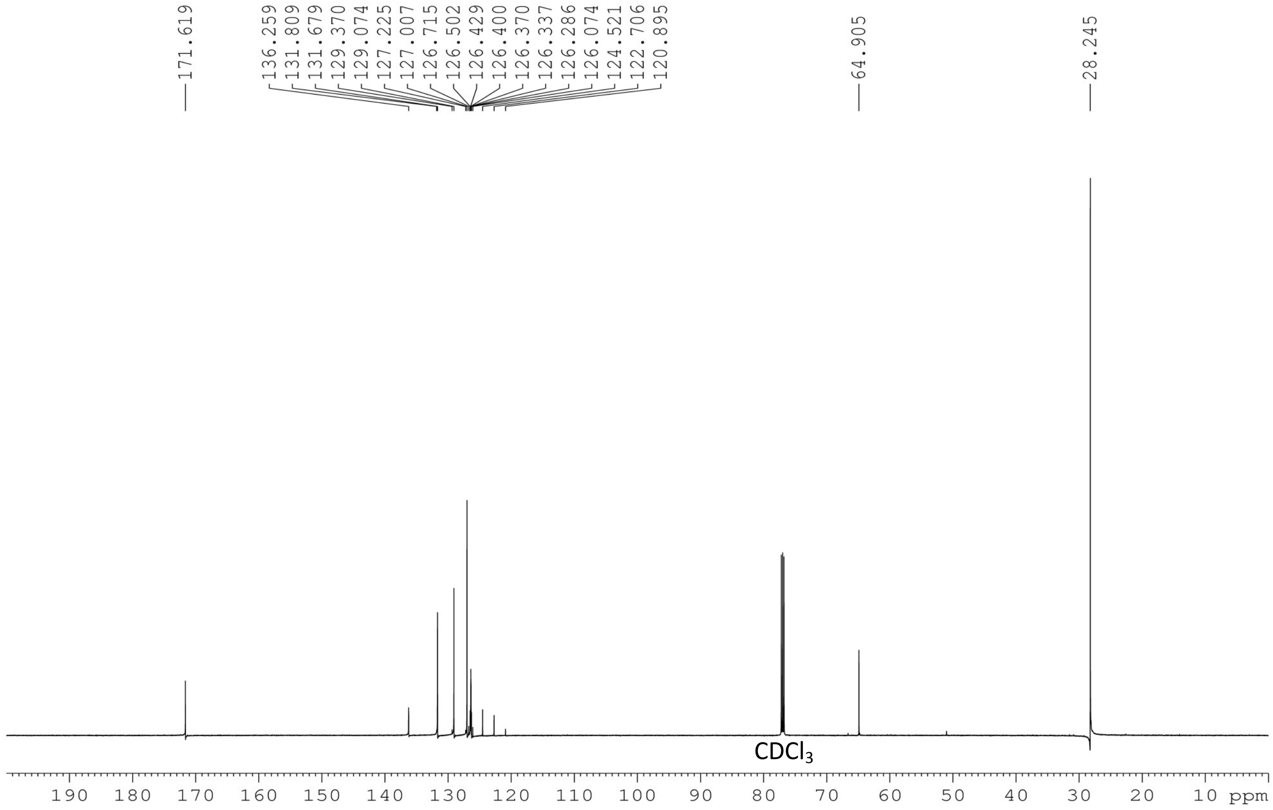
171.619
136.259
131.809
131.679
129.370
129.074
127.225
127.007
126.715
126.502
126.429
126.400
126.370
126.337
126.286
126.074
124.521
122.706
120.895
64.905
28.245
CDCl3
190 180 170 160 150 140 130 120 110 100 90 80 70 60 50 40 30 20 10 ppm

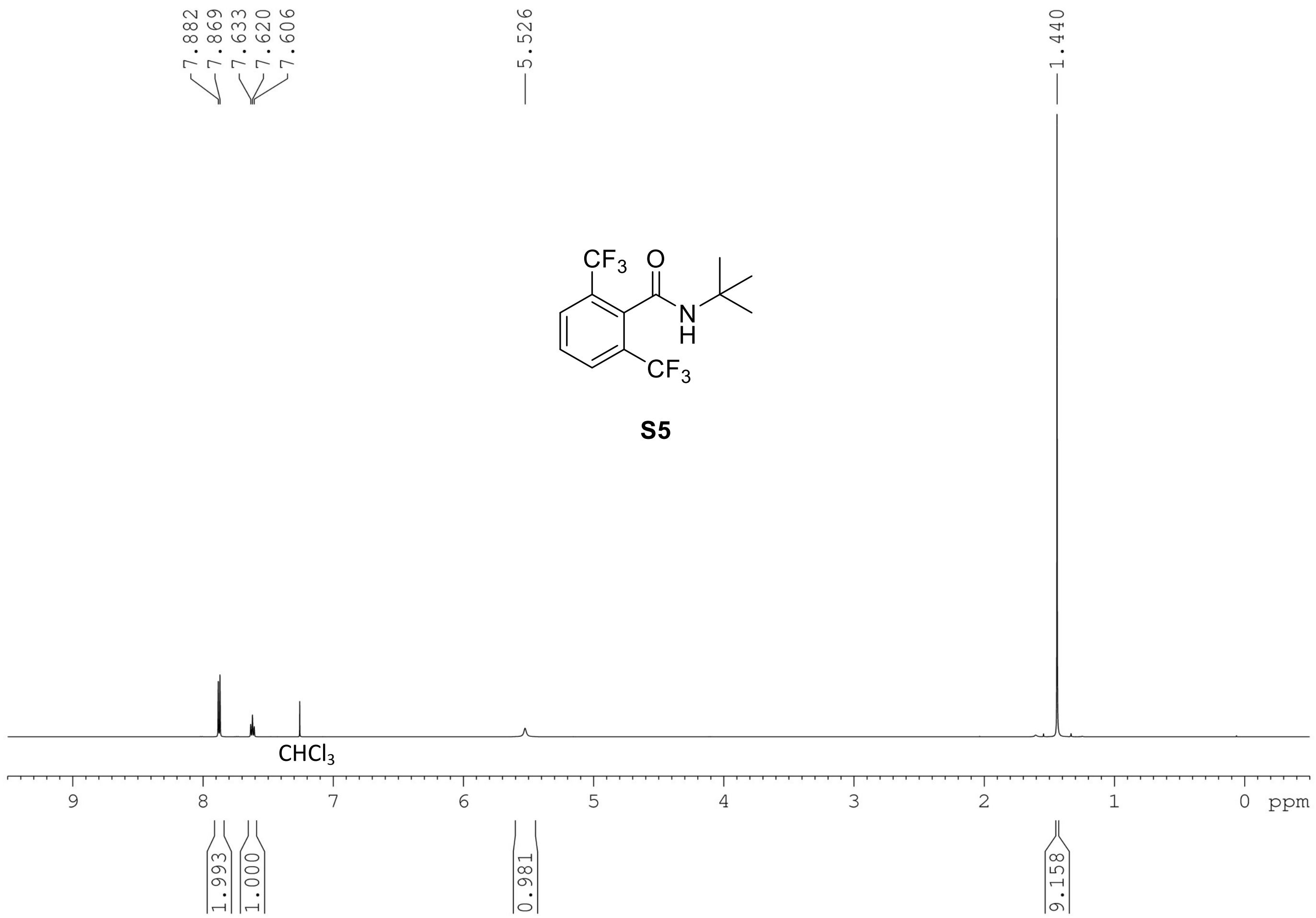
7.882
7.869
7.633
7.620
7.606
5.526
1.440
S5
CHCl3
9
8
7
6
5
4
3
2
1
0 ppm
1.993
1.000
0.981
9.158

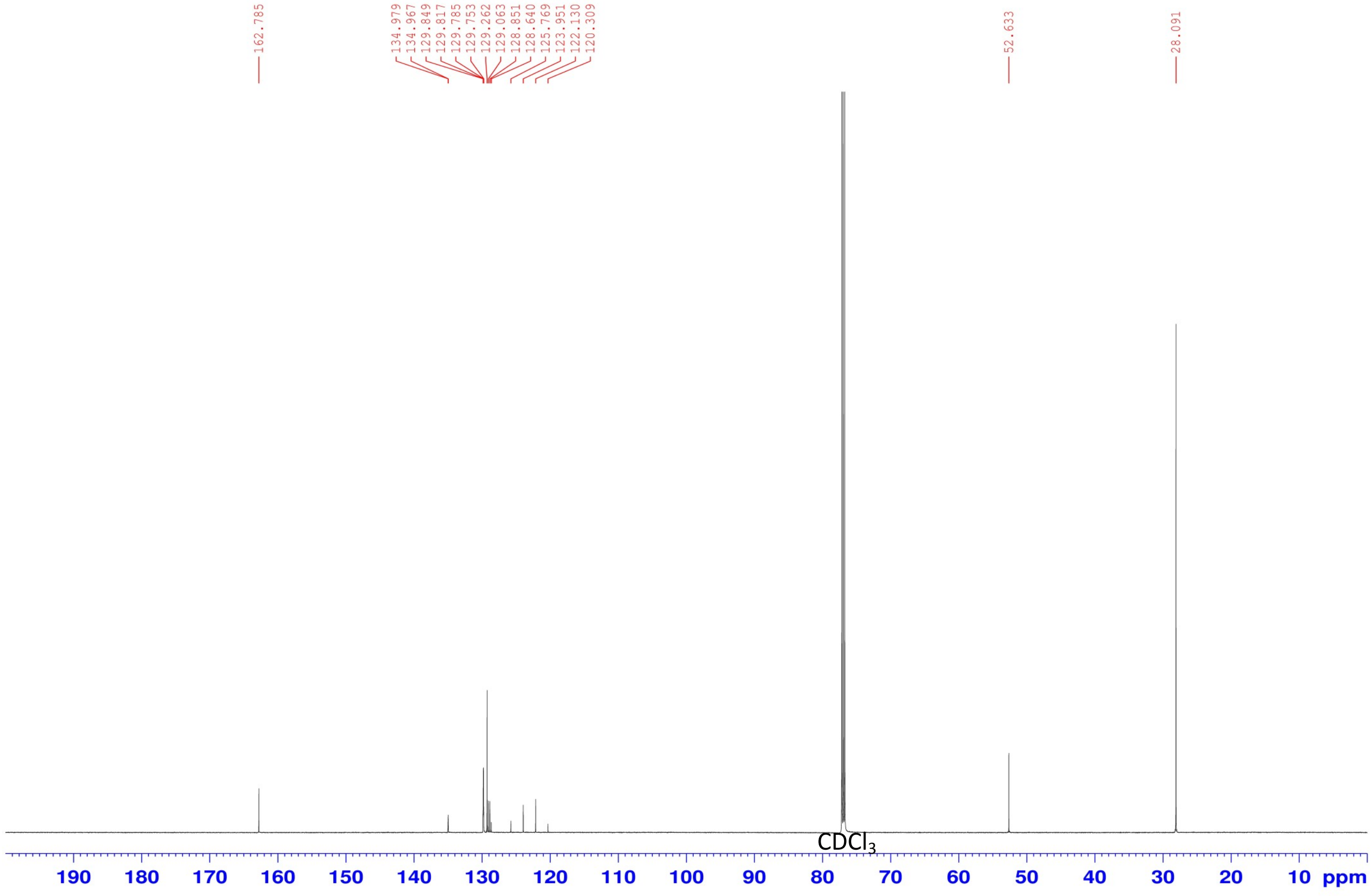
162.785
134.979
134.967
129.849
129.817
129.785
129.753
129.262
129.063
128.851
128.640
125.769
123.951
122.130
120.309
52.633
28.091
CDCl3
190
180
170
160
150
140
130
120
110
100
90
80
70
60
50
40
30
20
10 ppm

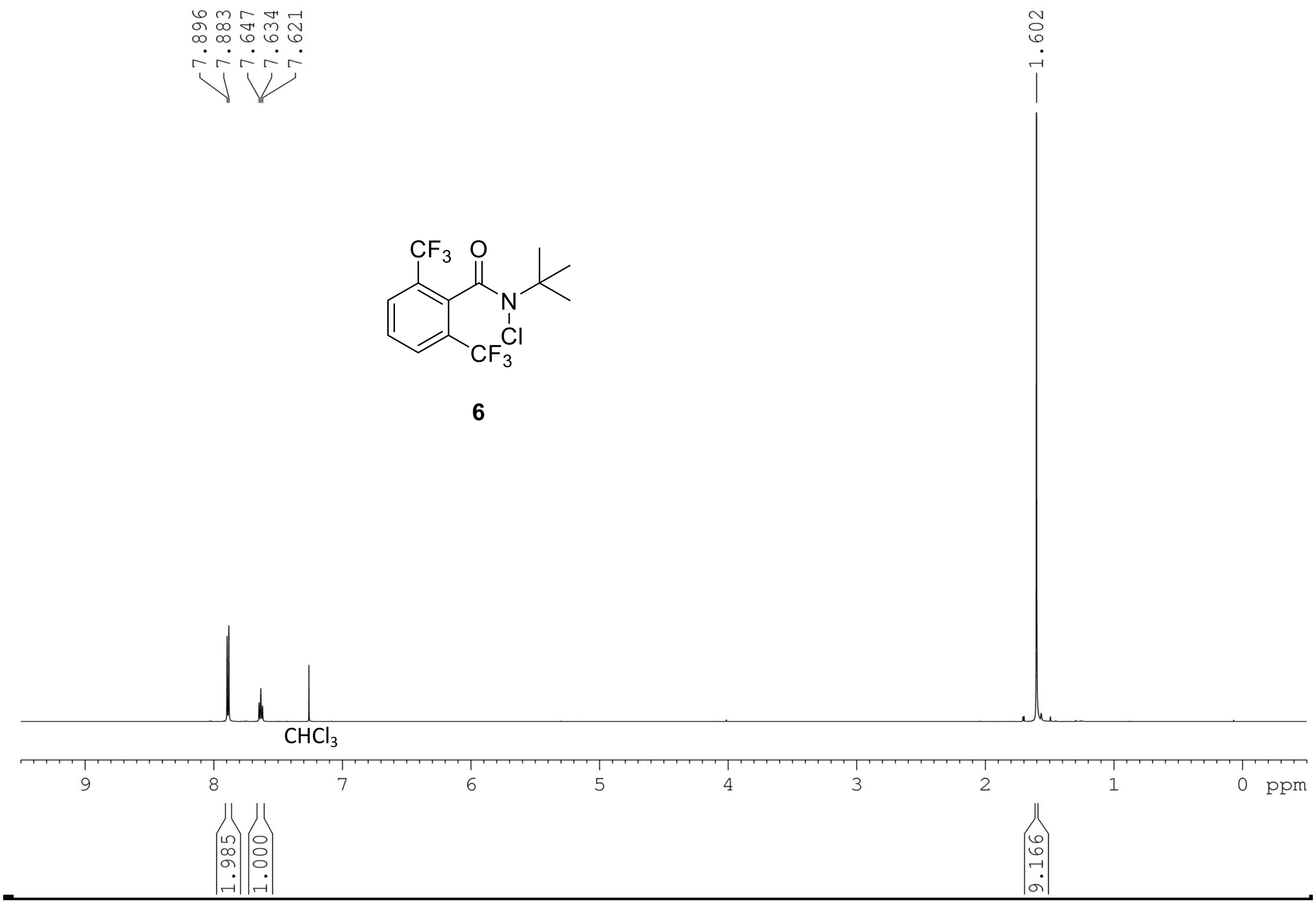

7.896
7.883
7.647
7.634
7.621
1.602
CF3
O
N
Cl
CF3
CHCl3
9 8 7 6 5 4 3 2 1 0 ppm
1.985
1.000
9.166

6

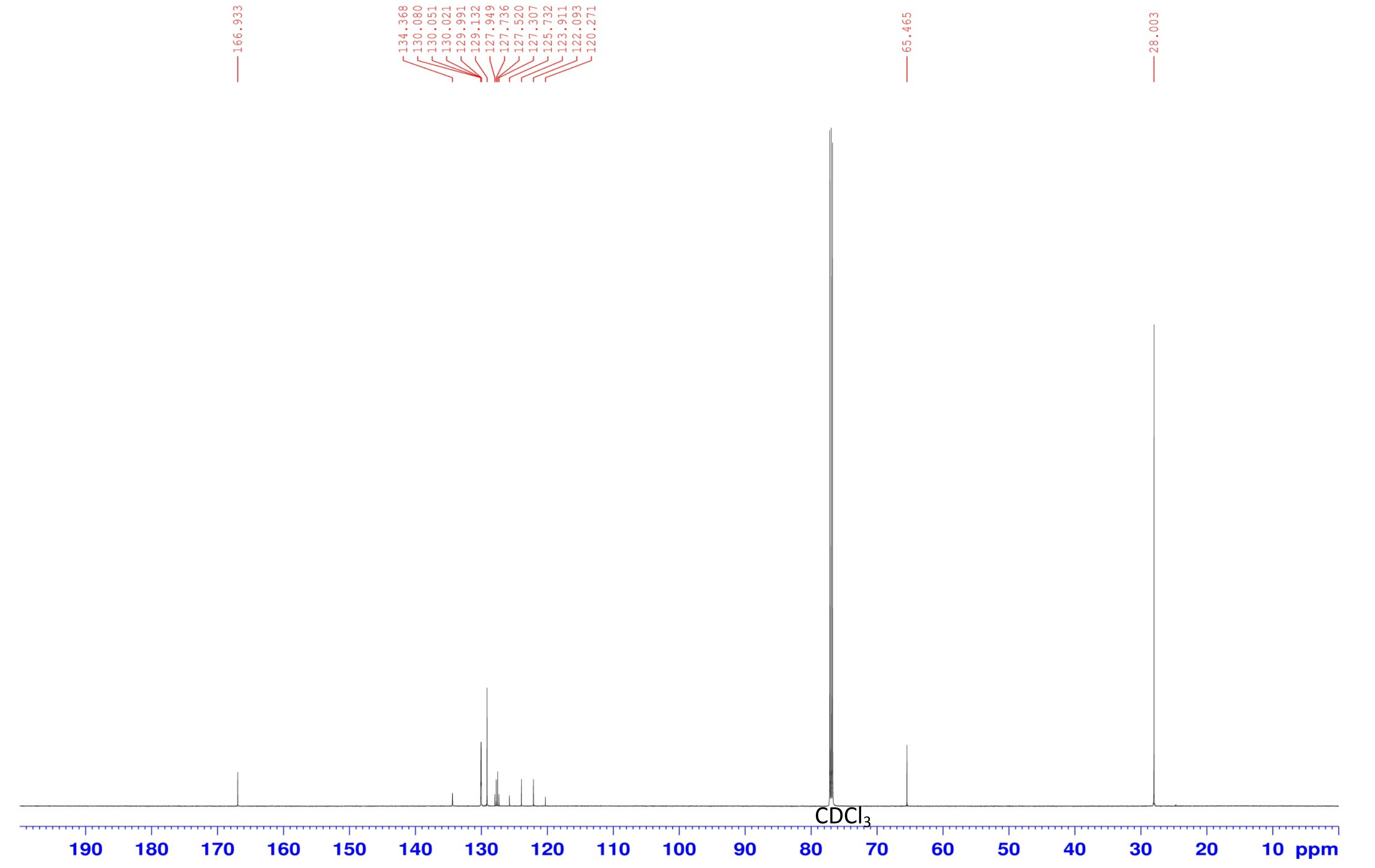

166.933
134.368
130.080
130.051
130.021
129.991
129.132
127.949
127.736
127.520
127.307
125.732
123.911
122.093
120.271
65.465
28.003
CDCl3
190 180 170 160 150 140 130 120 110 100 90 80 70 60 50 40 30 20 10 ppm

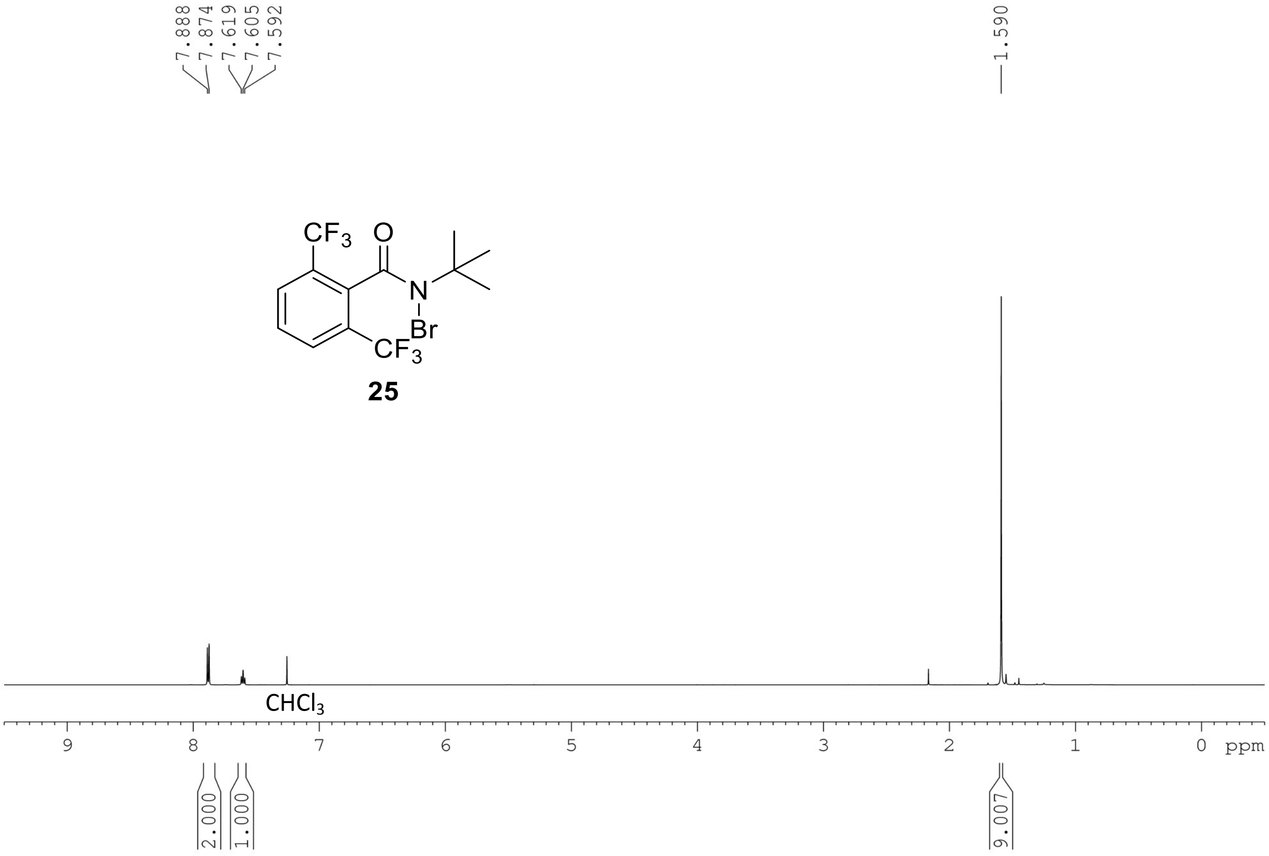

7.888
7.874
7.619
7.605
7.592
1.590
CF3
O
N
Br
CF3
25
CHCl3
9 8 7 6 5 4 3 2 1 0 ppm
2.000
1.000
9.007

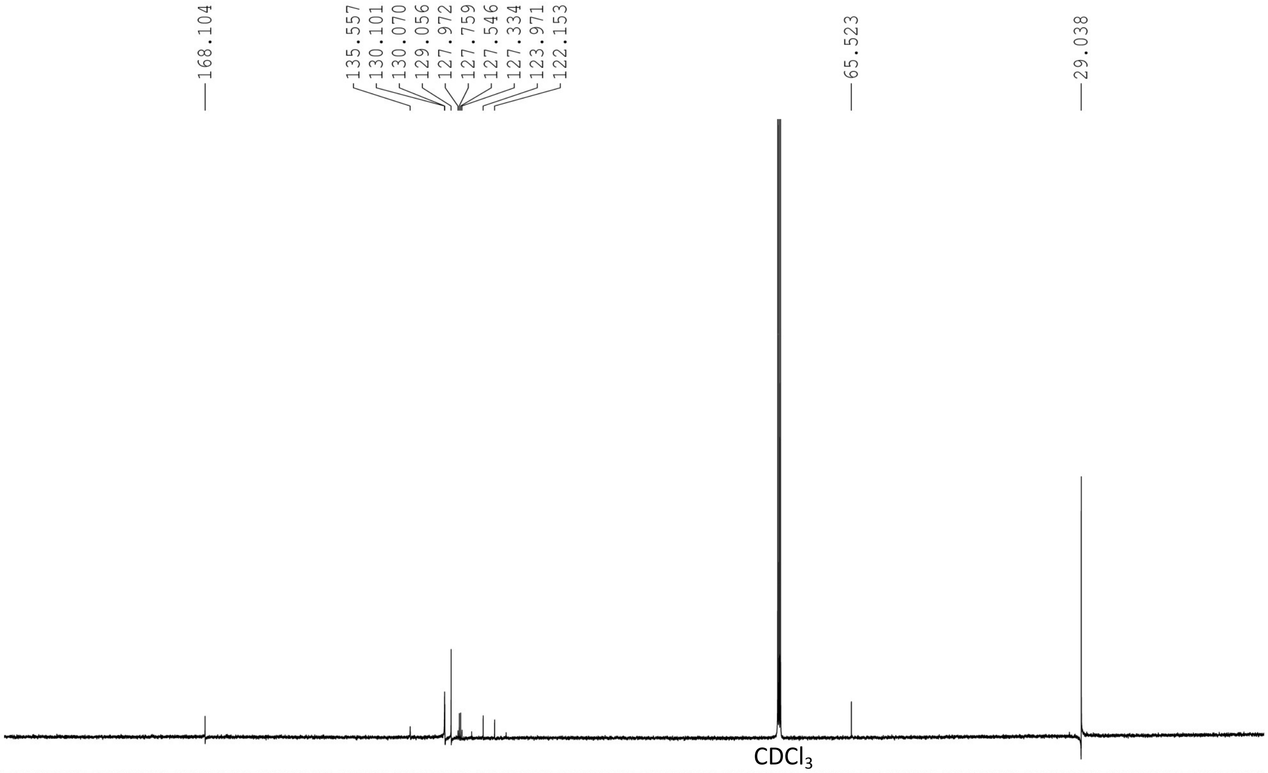

168.104
135.557
130.101
130.070
129.056
127.972
127.759
127.546
127.334
123.971
122.153
65.523
29.038
CDCl3
190 180 170 160 150 140 130 120 110 100 90 80 70 60 50 40 30 20 10 ppm

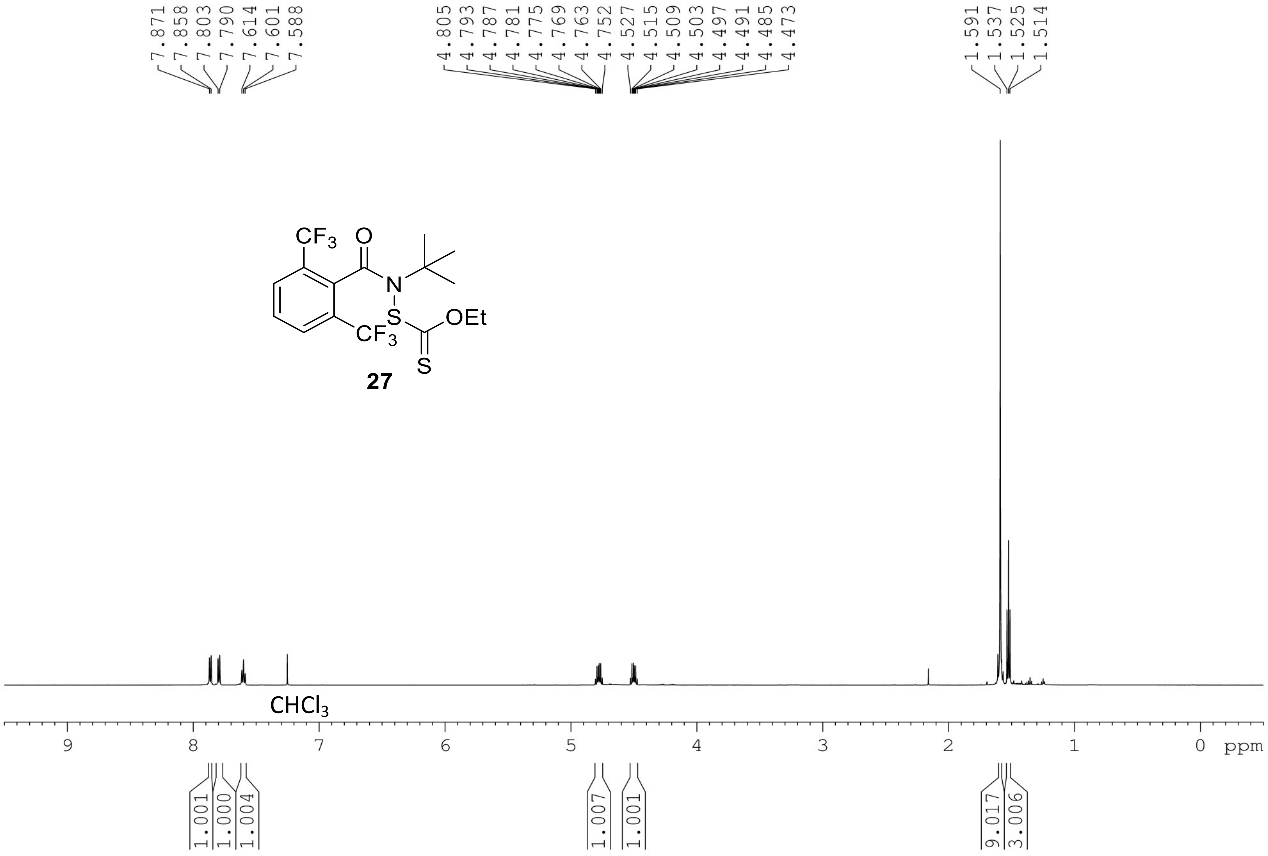

CF_3
O
N
S
OEt
CF_3
S
27
7.871
7.858
7.803
7.790
7.614
7.601
7.588
4.805
4.793
4.787
4.781
4.775
4.769
4.763
4.752
4.527
4.515
4.509
4.503
4.497
4.491
4.485
4.473
1.591
1.537
1.525
1.514
$CHCl_3$
9 8 7 6 5 4 3 2 1 0 ppm
1.001
1.000
1.004
1.007
1.001
9.017
3.006

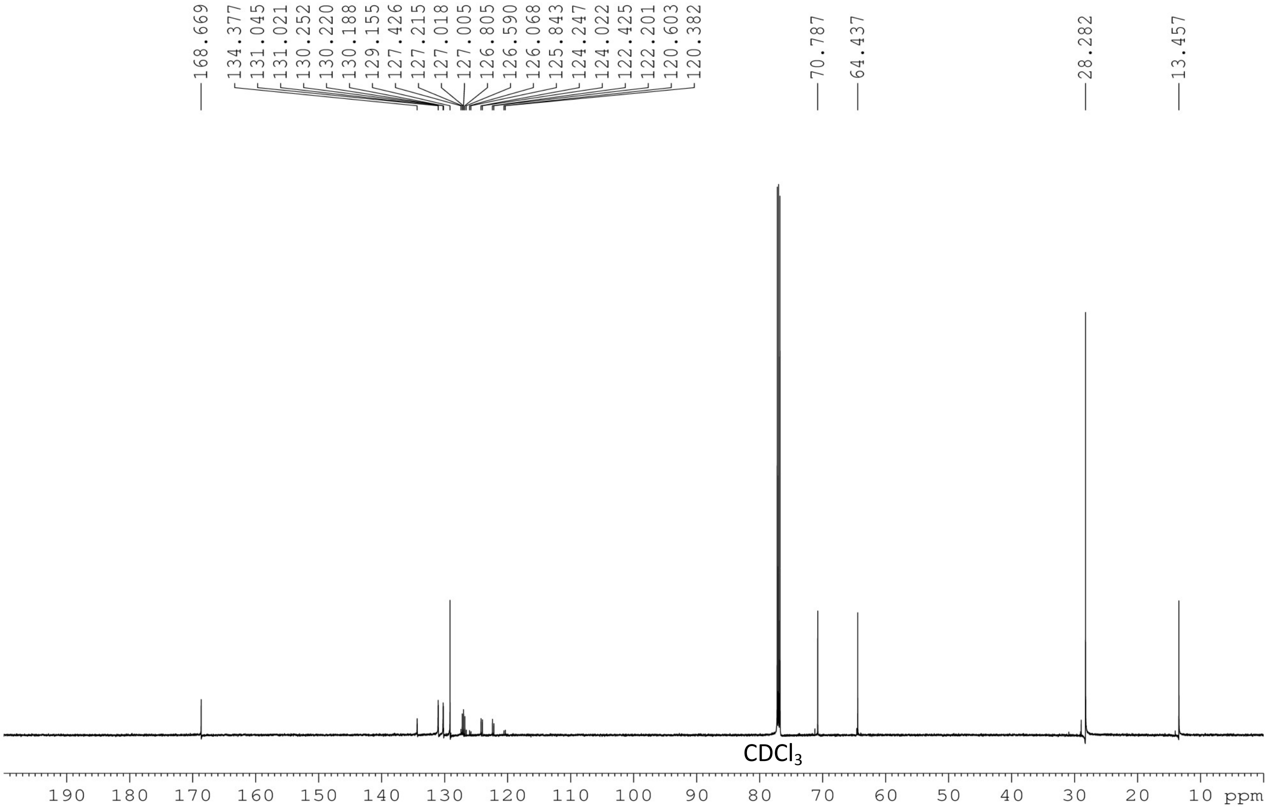

168.669
134.377
131.045
131.021
130.252
130.220
130.188
129.155
127.426
127.215
127.018
127.005
126.805
126.590
126.068
125.843
124.247
124.022
122.425
122.201
120.603
120.382
70.787
64.437
28.282
13.457
$CDCl_3$
190 180 170 160 150 140 130 120 110 100 90 80 70 60 50 40 30 20 10 ppm

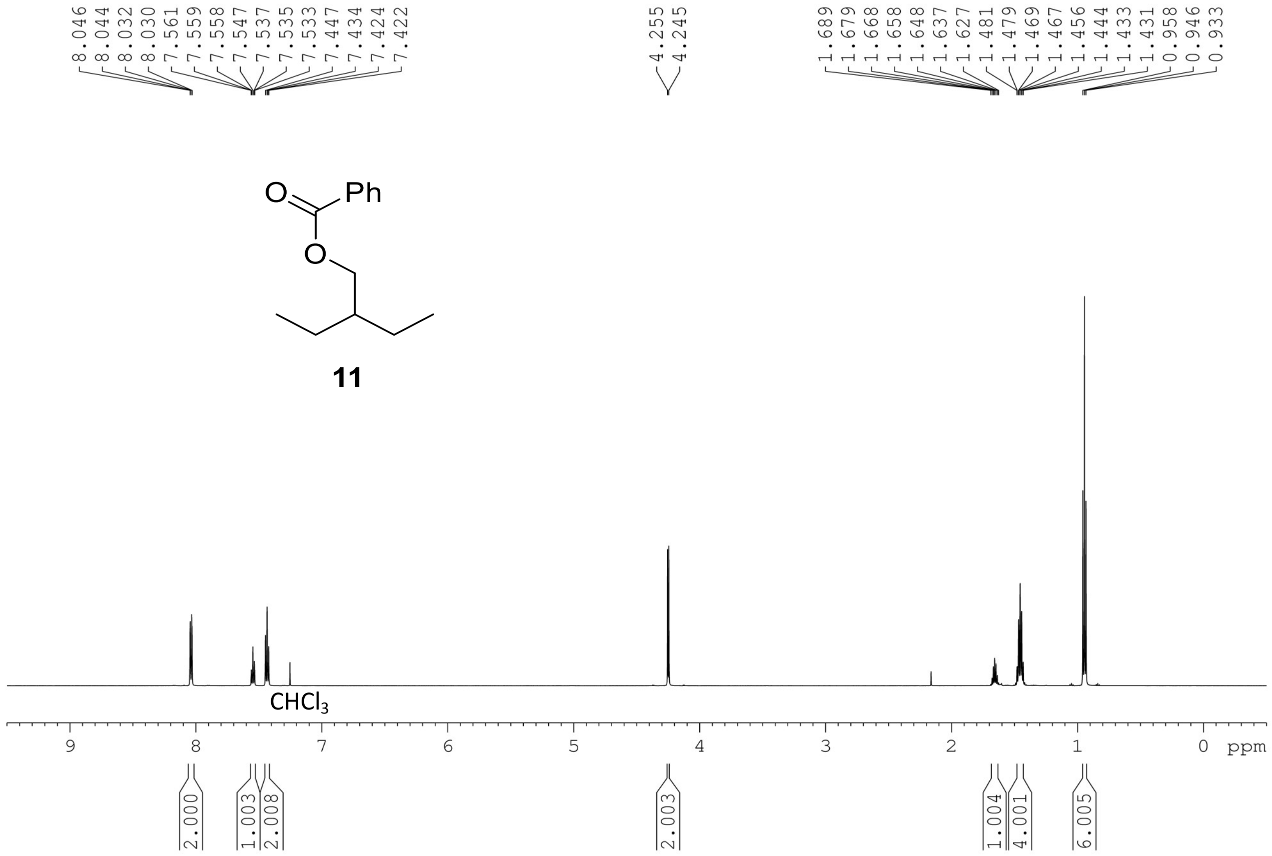
8.046
8.044
8.032
8.030
7.561
7.559
7.558
7.547
7.537
7.535
7.533
7.447
7.434
7.424
7.422
4.255
4.245
1.689
1.679
1.668
1.658
1.648
1.637
1.627
1.481
1.479
1.469
1.467
1.456
1.444
1.433
1.431
0.958
0.946
0.933
O
Ph
O
11
CHCl3
9 8 7 6 5 4 3 2 1 0 ppm
2.000
1.003
2.008
2.003
1.004
4.001
6.005

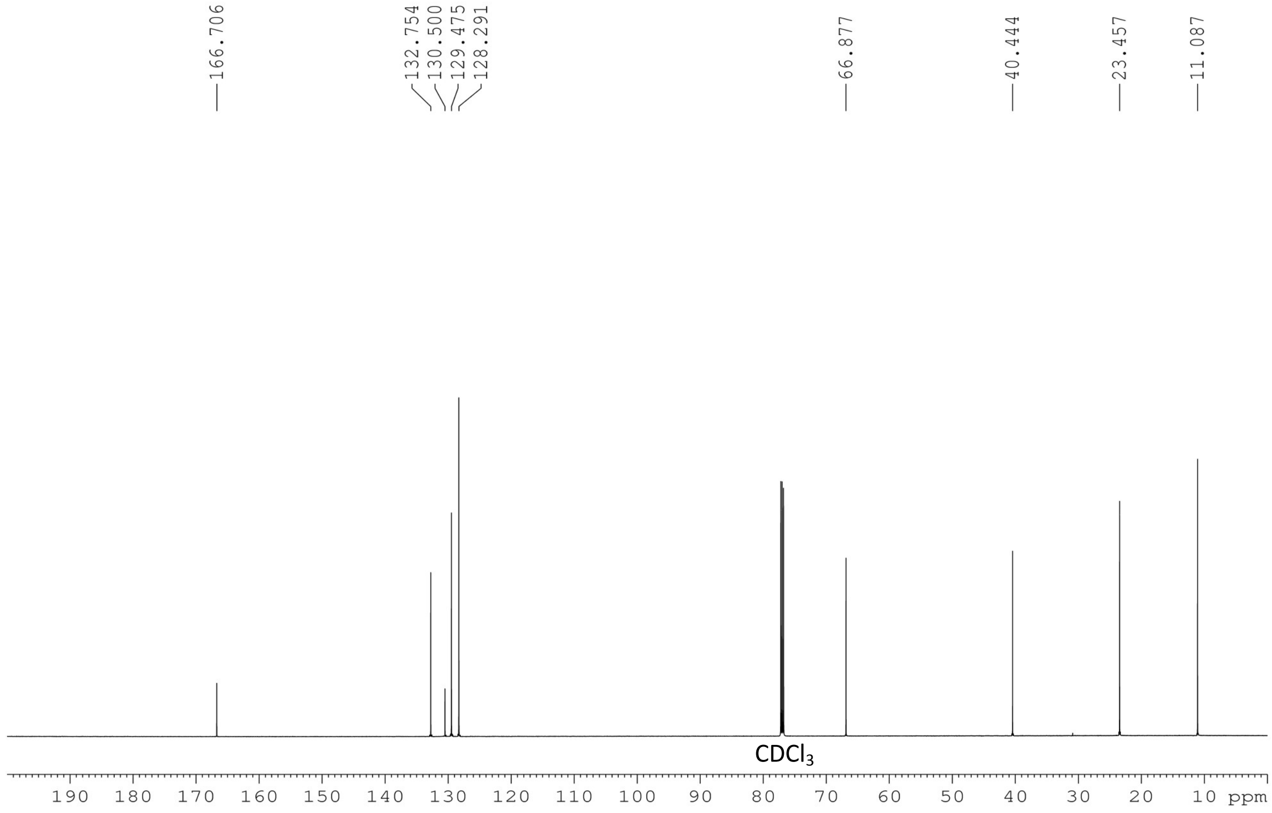
166.706
132.754
130.500
129.475
128.291
66.877
40.444
23.457
11.087
CDCl3
190 180 170 160 150 140 130 120 110 100 90 80 70 60 50 40 30 20 10 ppm

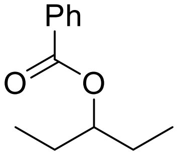

Ph
O
O

12

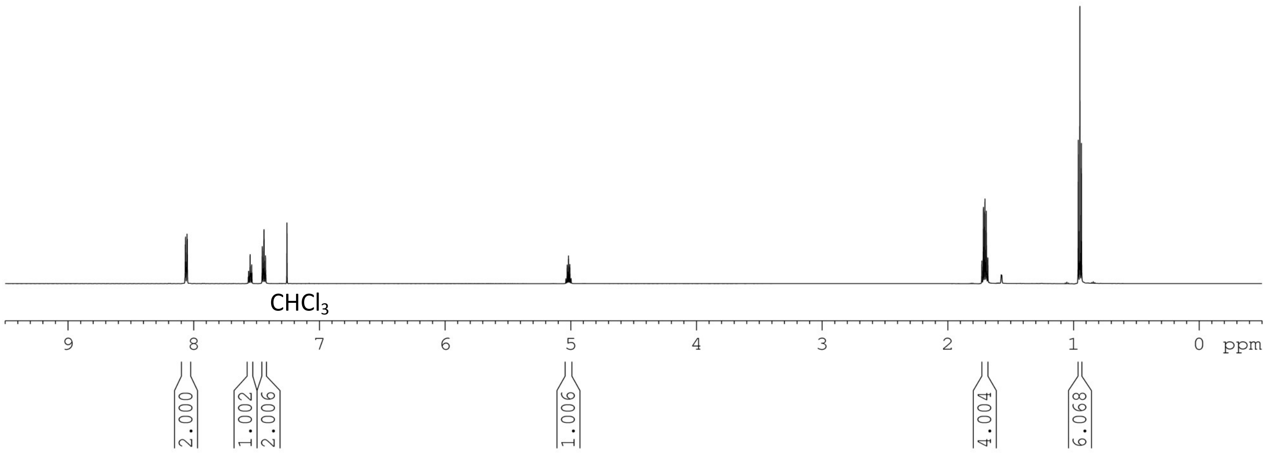

8.066
8.065
8.056
8.053
8.050
7.565
7.563
7.561
7.553
7.550
7.548
7.540
7.538
7.536
7.454
7.451
7.440
7.431
7.428
5.038
5.027
5.017
5.007
4.997
1.729
1.716
1.706
1.704
1.694
1.681
0.962
0.949
0.937
CHCl3
9 8 7 6 5 4 3 2 1 0 ppm
2.000
1.002
2.006
1.006
4.004
6.068

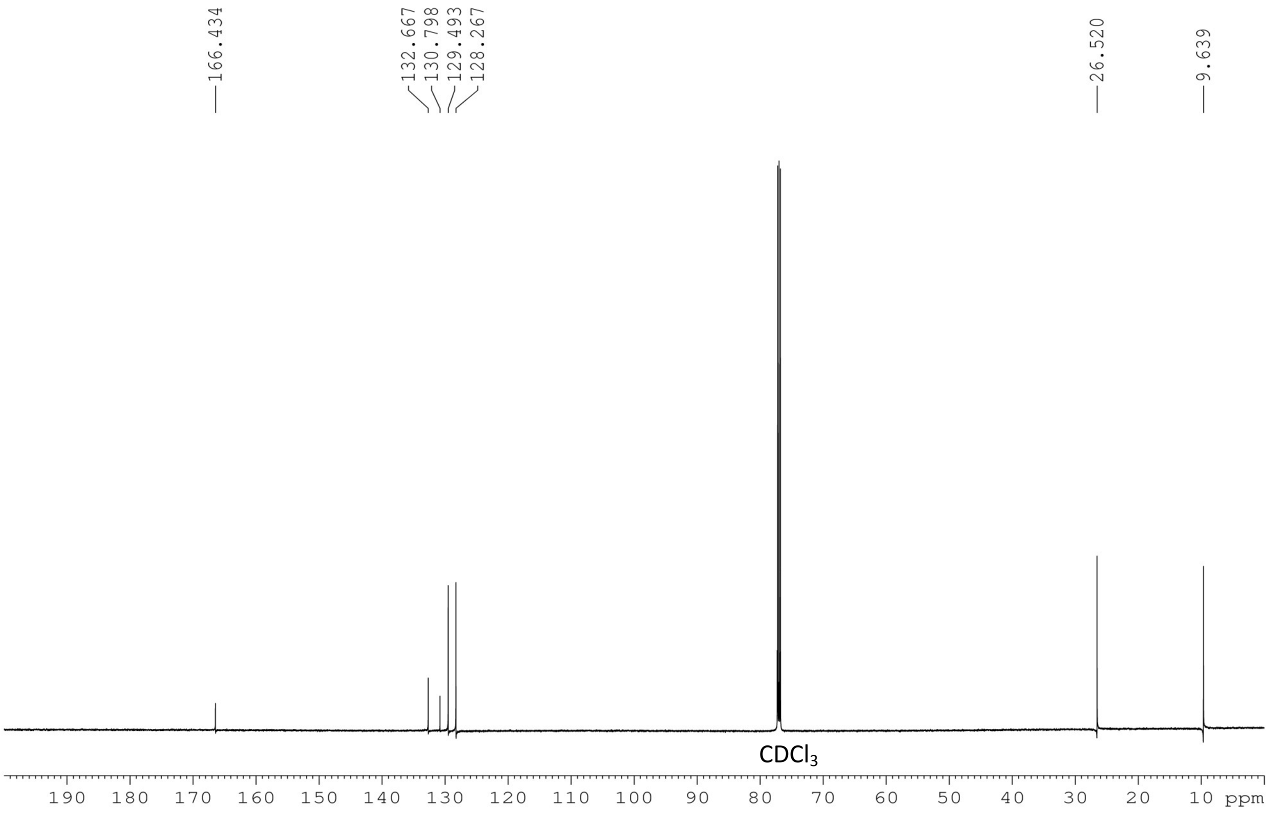

166.434
132.667
130.798
129.493
128.267
26.520
9.639
CDCl3
190 180 170 160 150 140 130 120 110 100 90 80 70 60 50 40 30 20 10 ppm

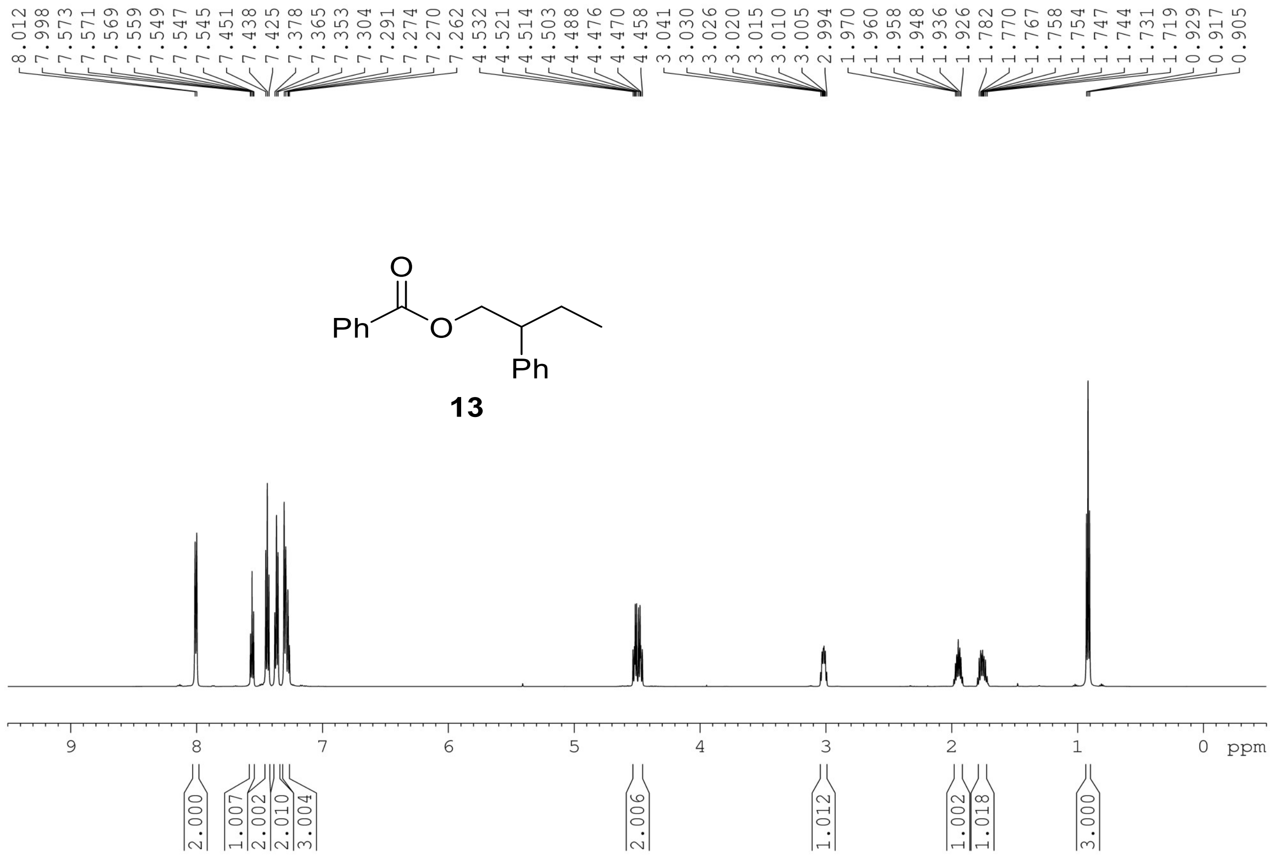
8.012
7.998
7.573
7.571
7.569
7.559
7.549
7.547
7.545
7.451
7.438
7.425
7.378
7.365
7.353
7.304
7.291
7.274
7.270
7.262
4.532
4.521
4.514
4.503
4.488
4.476
4.470
4.458
3.041
3.030
3.026
3.020
3.015
3.010
3.005
2.994
1.970
1.960
1.958
1.948
1.936
1.926
1.782
1.770
1.767
1.758
1.754
1.747
1.744
1.731
1.719
0.929
0.917
0.905
9 8 7 6 5 4 3 2 1 0 ppm
2.000
1.007
2.002
2.010
3.004
2.006
1.012
1.002
1.018
3.000
O
Ph
O
Ph

13

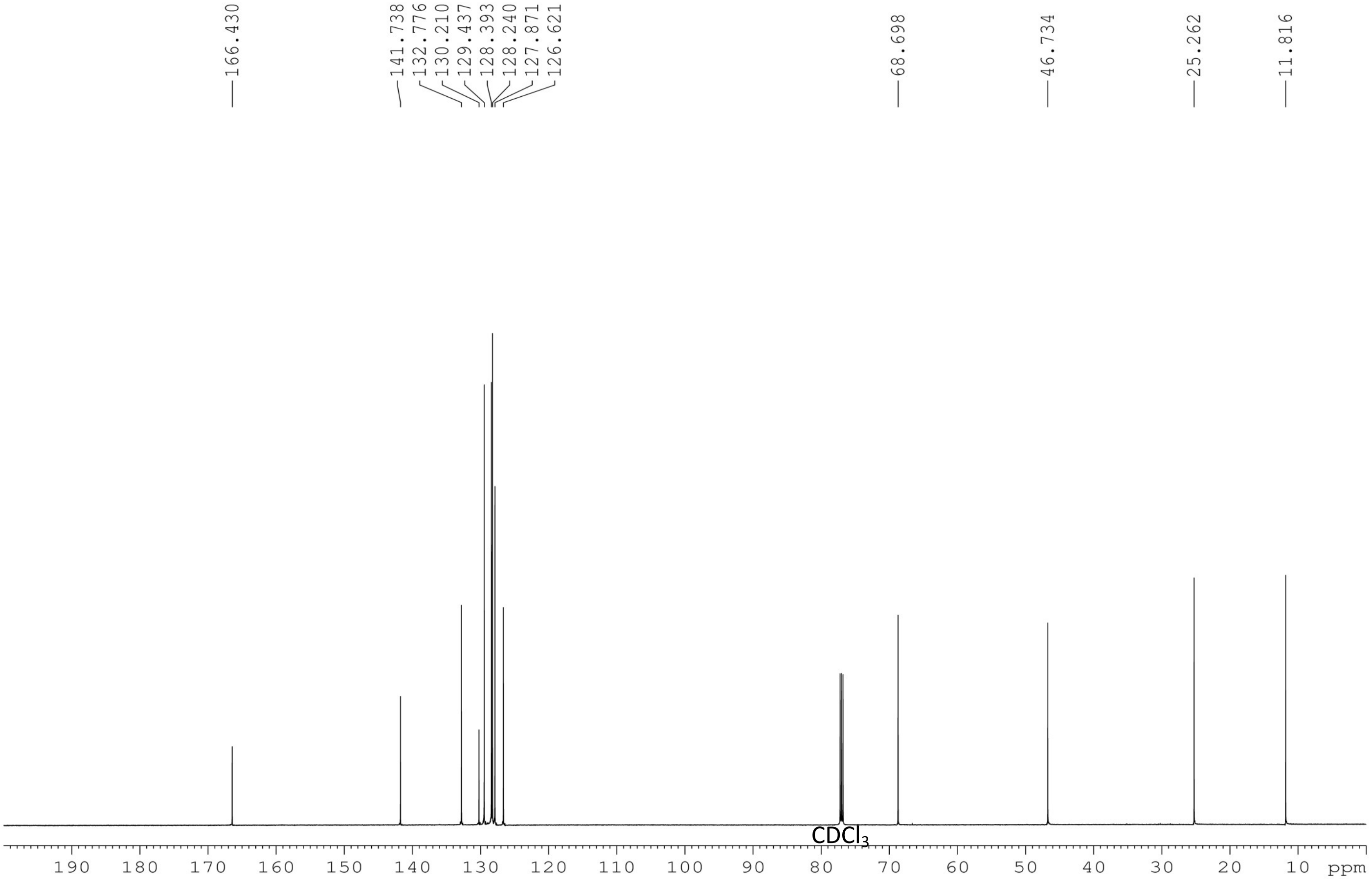
166.430
141.738
132.776
130.210
129.437
128.393
128.240
127.871
126.621
68.698
46.734
25.262
11.816
CDCl3
190 180 170 160 150 140 130 120 110 100 90 80 70 60 50 40 30 20 10 ppm

14

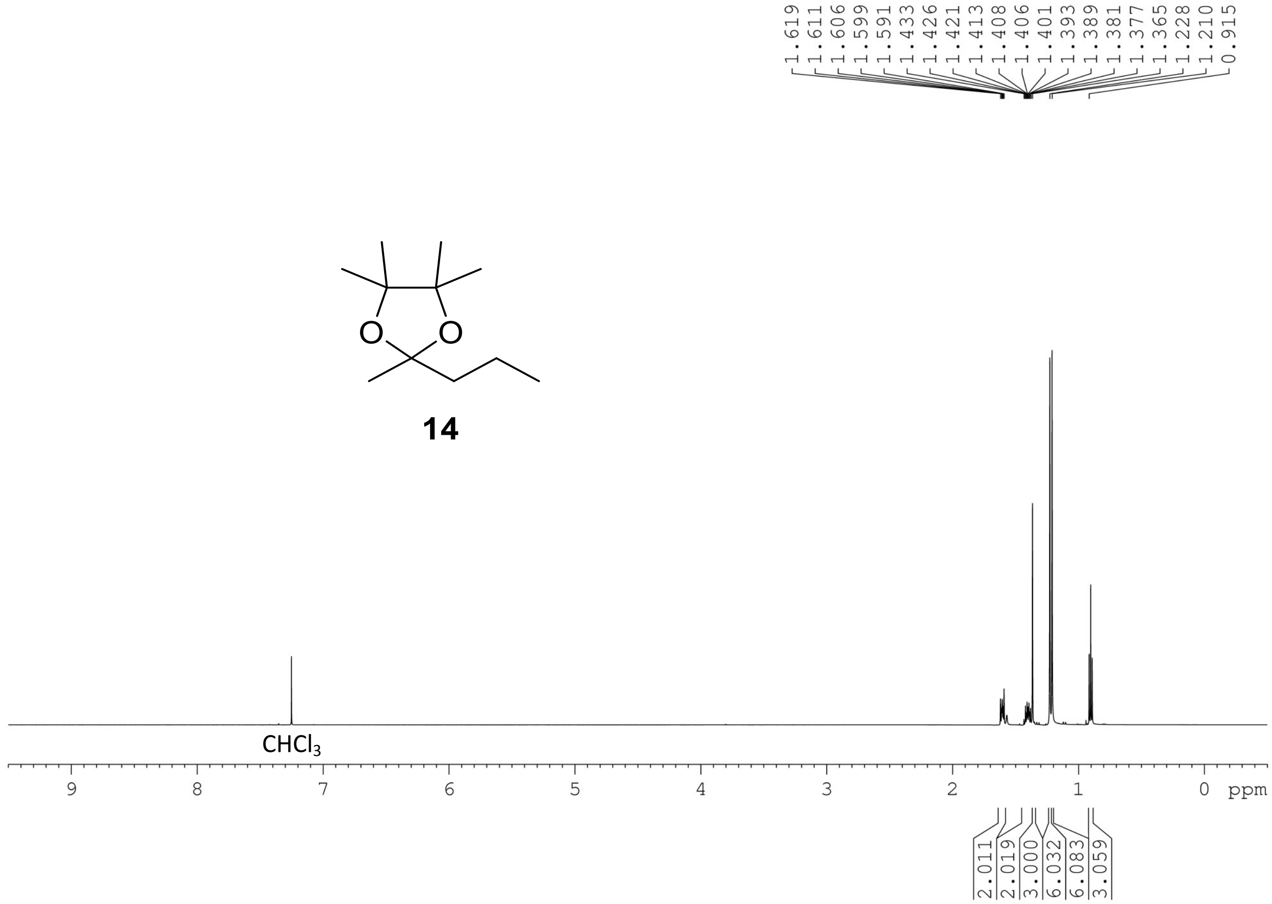
1.619
1.611
1.606
1.599
1.591
1.433
1.426
1.421
1.413
1.408
1.406
1.401
1.393
1.389
1.381
1.377
1.365
1.228
1.210
0.915
CHCl$_3$
9
8
7
6
5
4
3
2
1
0 ppm
2.011
2.019
3.000
6.032
6.083
3.059

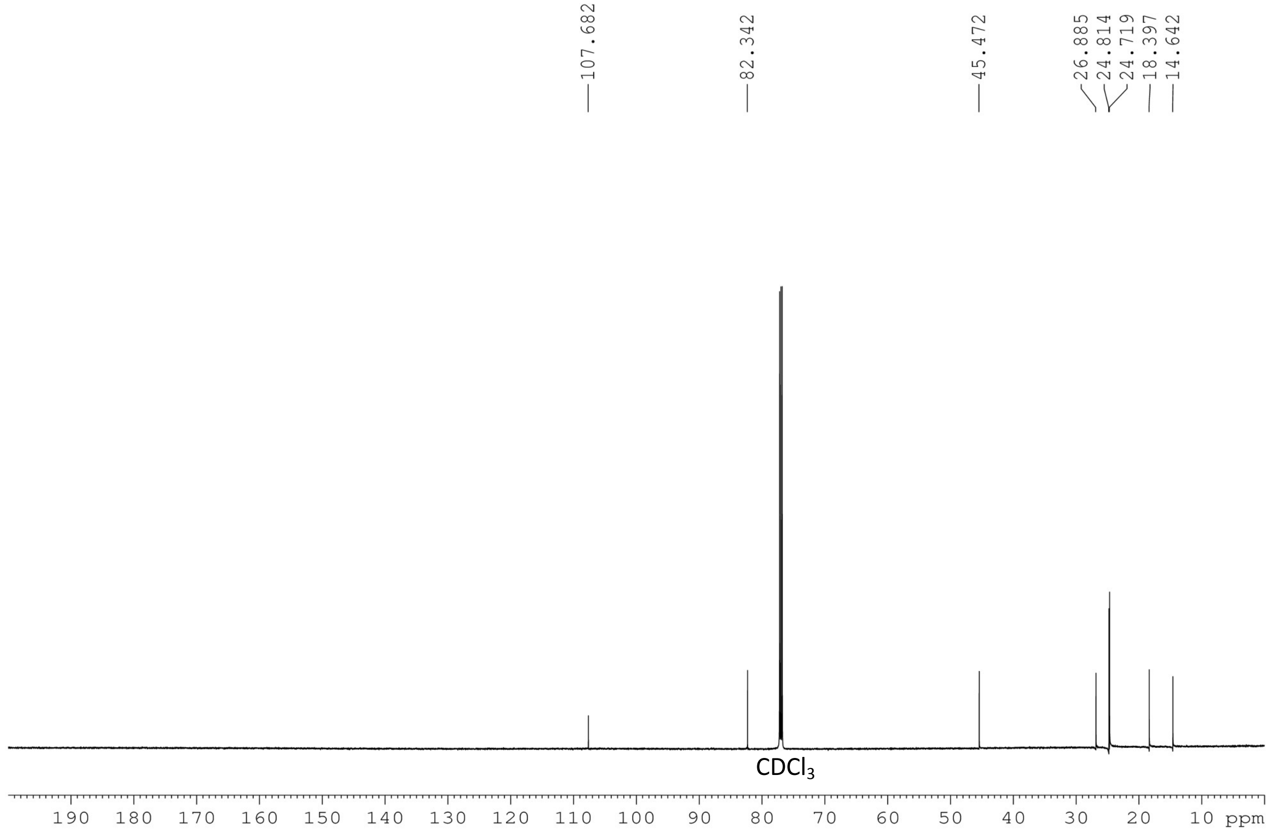
107.682
82.342
45.472
26.885
24.814
24.719
18.397
14.642
CDCl$_3$
190 180 170 160 150 140 130 120 110 100 90 80 70 60 50 40 30 20 10 ppm

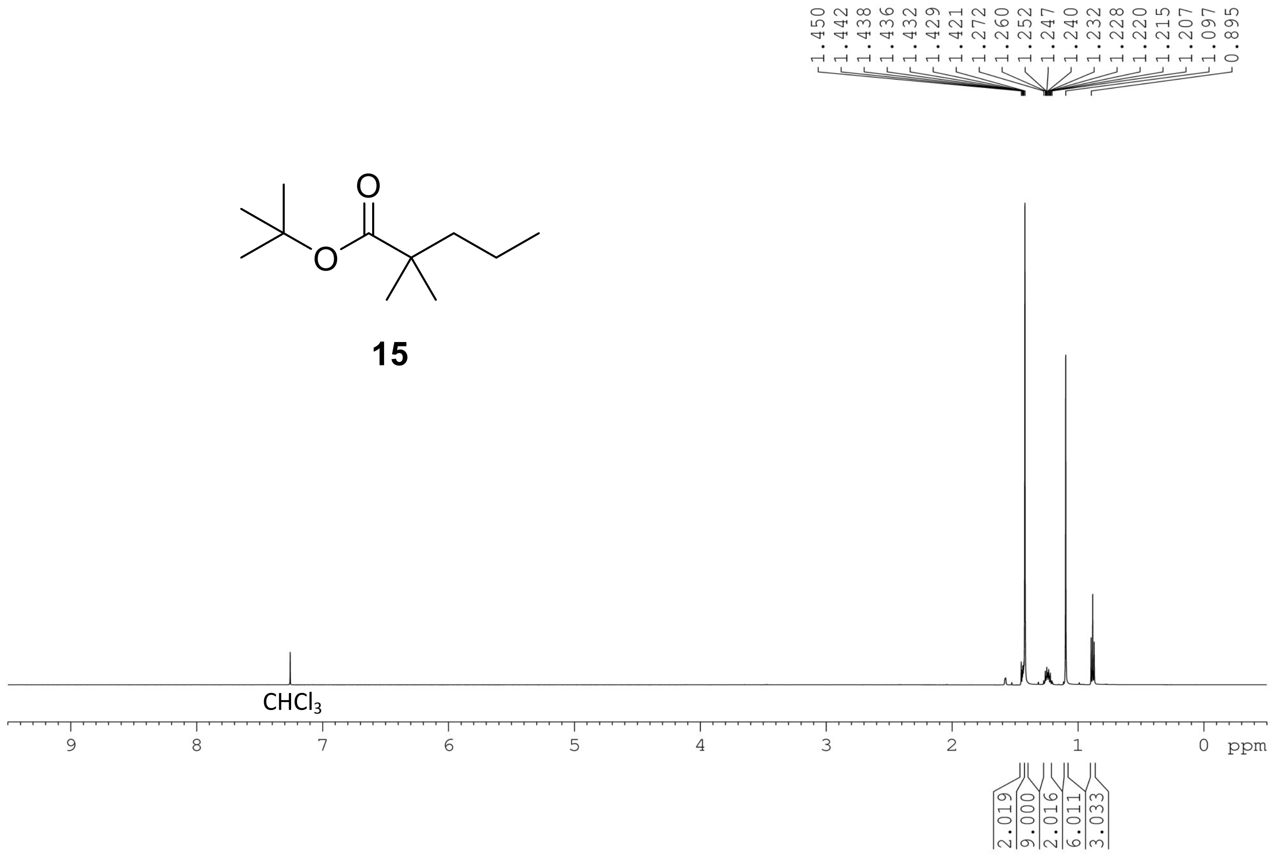

15

1.450
1.442
1.438
1.436
1.432
1.429
1.421
1.272
1.260
1.252
1.247
1.240
1.232
1.228
1.220
1.215
1.207
1.097
0.895
CHCl$_3$
9 8 7 6 5 4 3 2 1 0 ppm
2.019
9.000
2.016
6.011
3.033

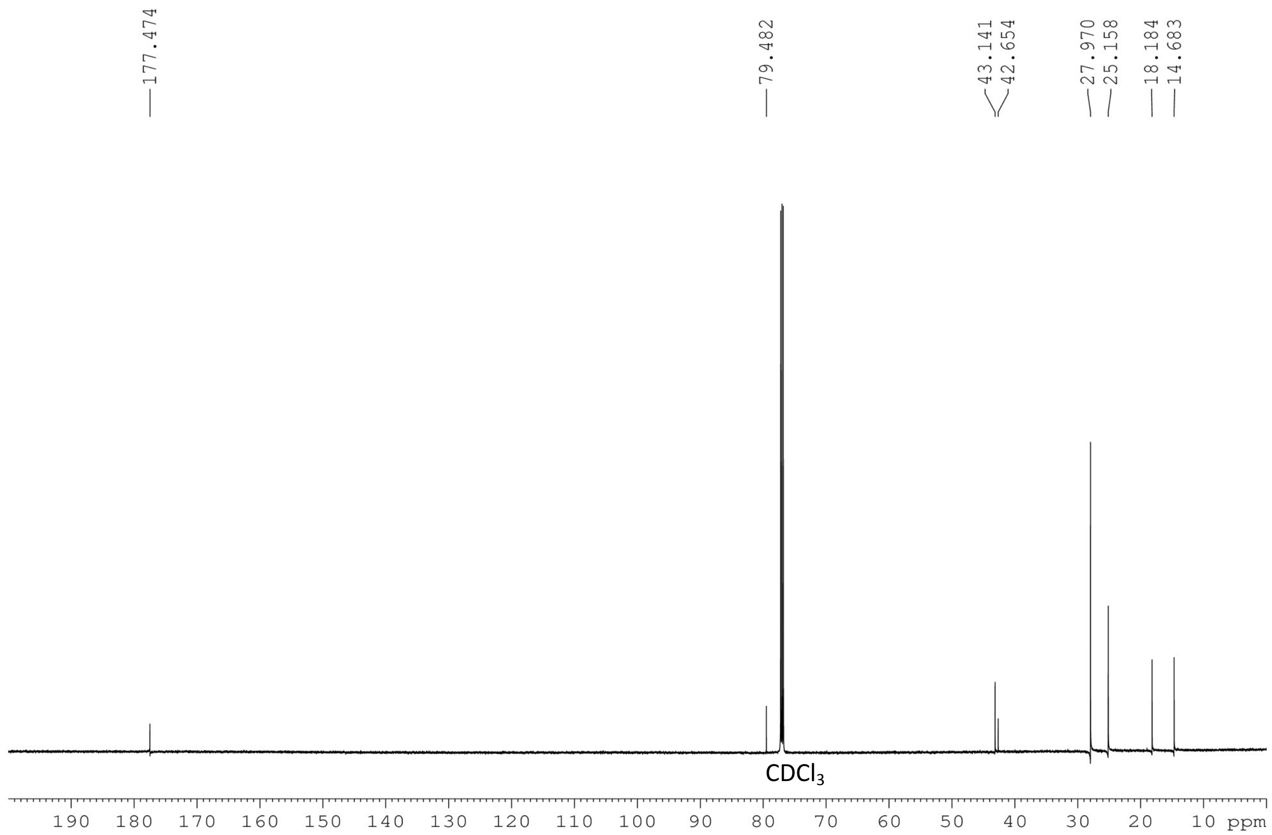
177.474
79.482
43.141
42.654
27.970
25.158
18.184
14.683
CDCl$_3$
190 180 170 160 150 140 130 120 110 100 90 80 70 60 50 40 30 20 10 ppm

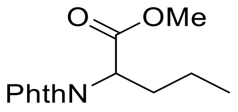

18

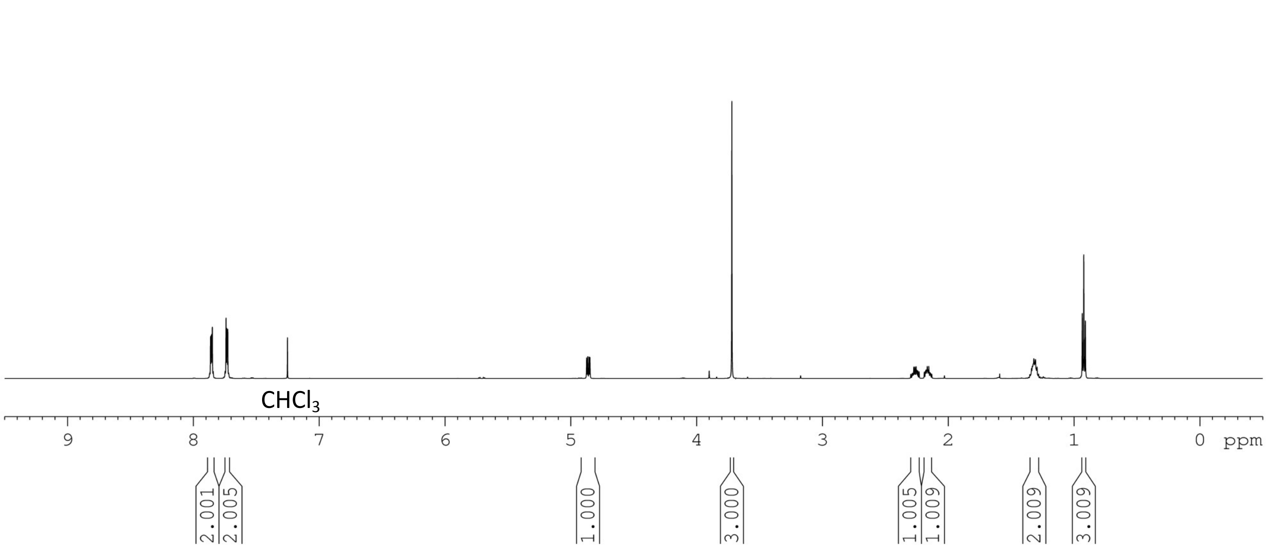
7.869
7.863
7.858
7.854
7.850
7.849
7.842
7.748
7.747
7.740
7.735
7.731
7.726
7.720
4.874
4.866
4.856
4.848
3.720
2.288
2.283
2.279
2.274
2.270
2.265
2.259
2.256
2.250
2.247
2.241
2.232
2.190
2.182
2.178
2.175
2.170
2.167
2.163
2.159
2.155
2.146
2.139
1.339
1.332
1.329
1.325
1.320
1.318
1.307
1.302
1.294
0.935
0.923
0.910
CHCl3
ppm
2.001
2.005
1.000
3.000
1.005
1.009
2.009
3.009

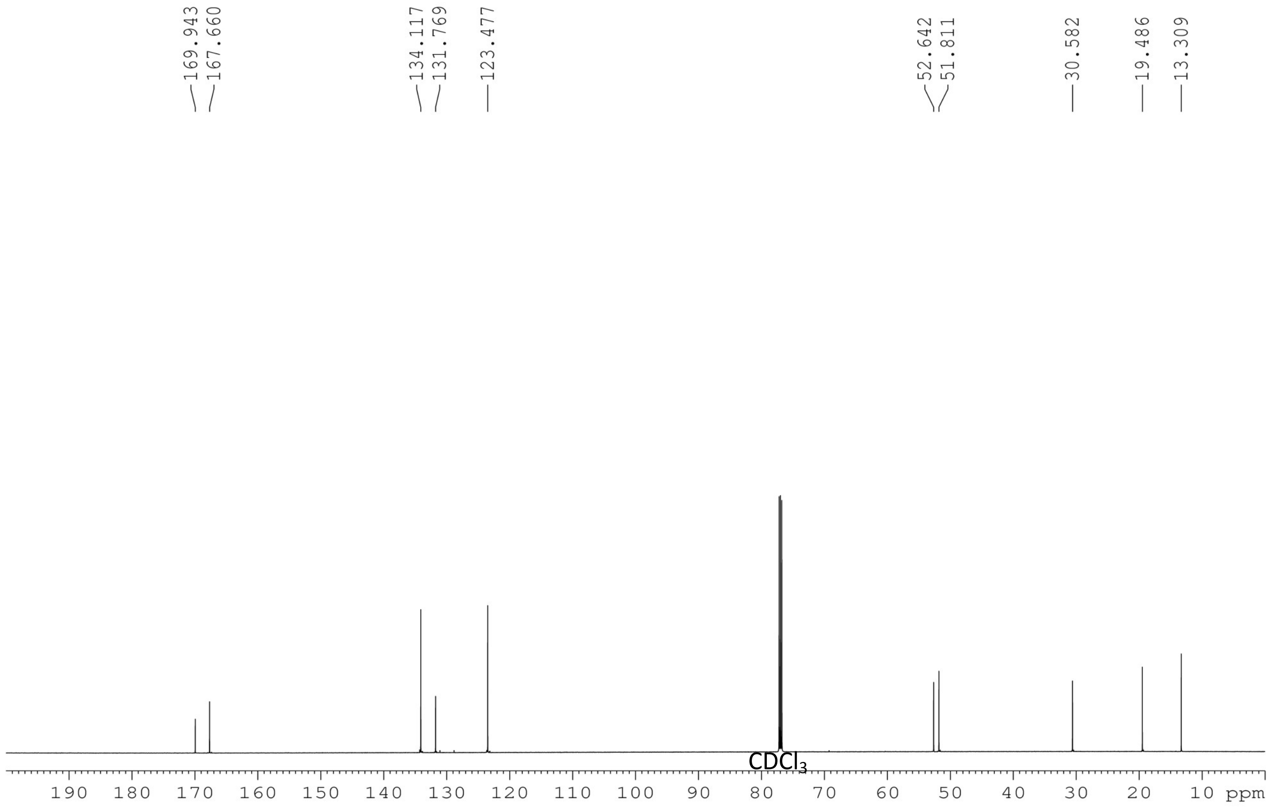
169.943
167.660
134.117
131.769
123.477
52.642
51.811
30.582
19.486
13.309
CDCl3
ppm

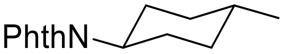
PhthN

20

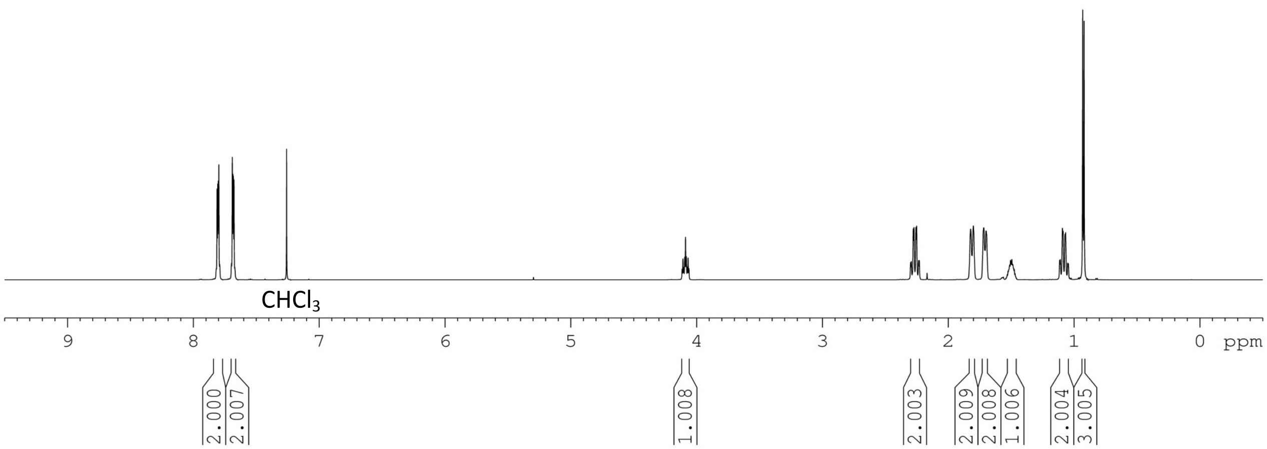
7.812
7.807
7.803
7.798
7.691
7.686
7.682
7.677
4.115
4.109
4.102
4.095
4.088
4.082
4.074
4.068
4.061
2.300
2.294
2.278
2.273
2.257
2.251
2.236
2.230
1.822
1.801
1.719
1.700
1.531
1.525
1.519
1.511
1.506
1.500
1.495
1.489
1.486
1.481
1.475
1.470
1.465
CHCl₃
9
8
7
6
5
4
3
2
1
0 ppm
2.000
2.007
1.008
2.003
2.009
2.008
1.006
2.004
3.005

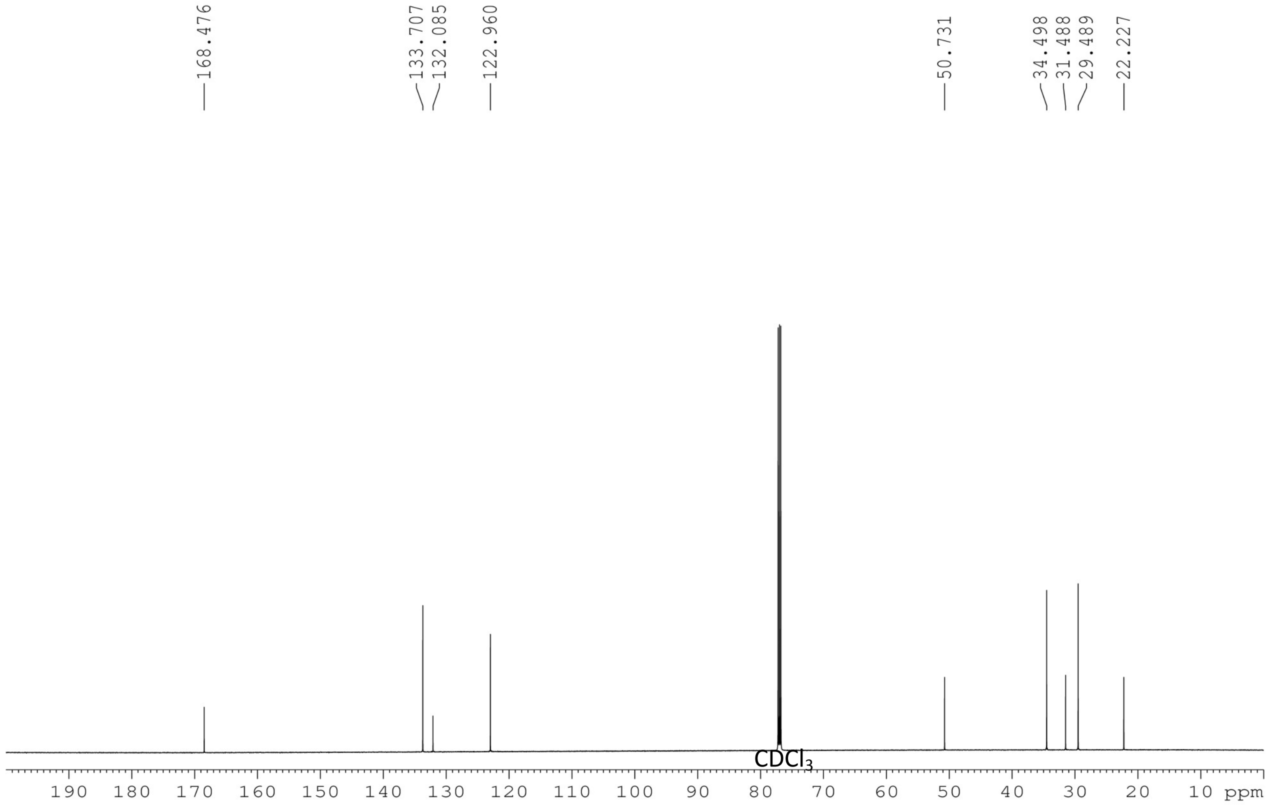
168.476
133.707
132.085
122.960
50.731
34.498
31.488
29.489
22.227
CDCl₃
190
180
170
160
150
140
130
120
110
100
90
80
70
60
50
40
30
20
10 ppm

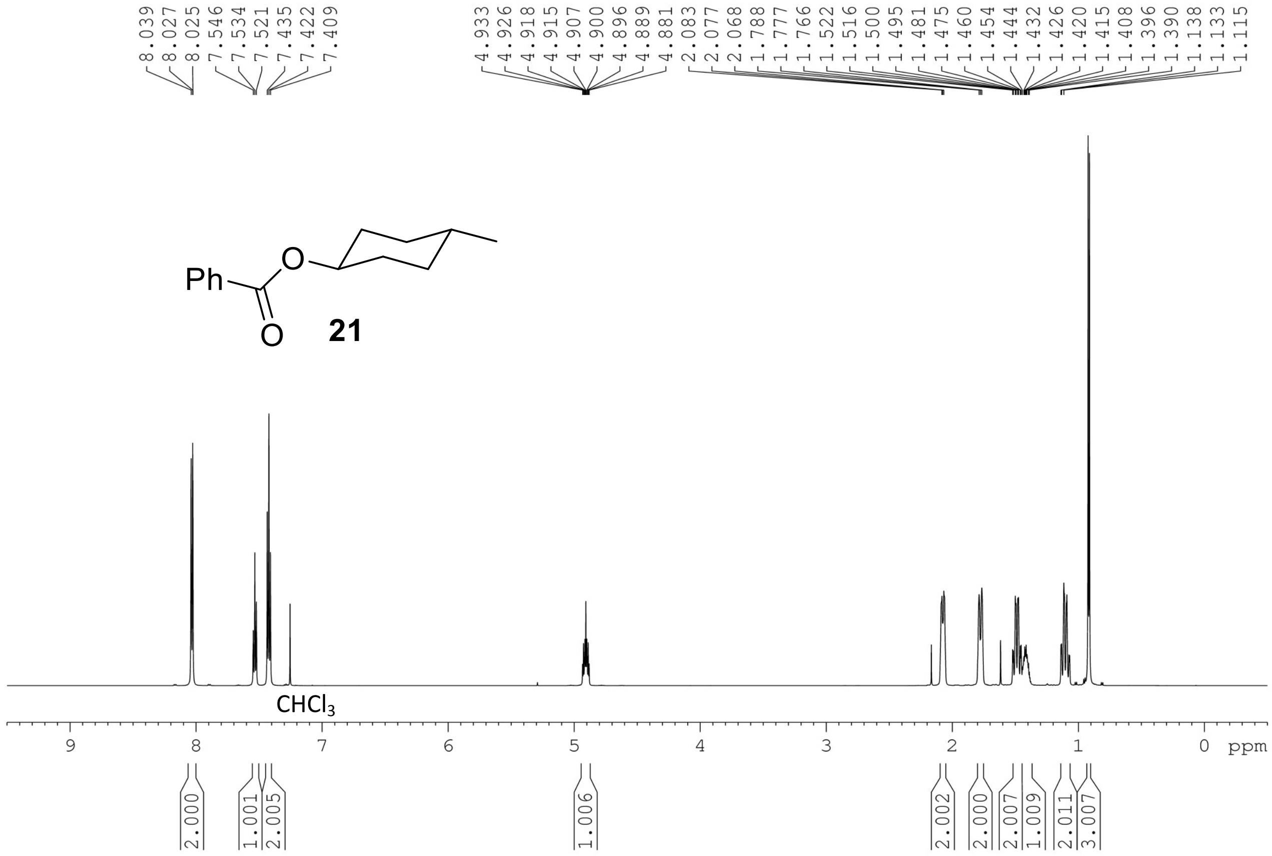

8.039
8.027
8.025
7.546
7.534
7.521
7.435
7.422
7.409
4.933
4.926
4.918
4.915
4.907
4.900
4.896
4.889
4.881
2.083
2.077
2.068
1.788
1.777
1.766
1.522
1.516
1.500
1.495
1.481
1.475
1.460
1.454
1.444
1.432
1.426
1.420
1.415
1.408
1.396
1.390
1.138
1.133
1.115
Ph
O
O
21
CHCl3
9 8 7 6 5 4 3 2 1 0 ppm
2.000
1.001
2.005
1.006
2.002
2.000
2.007
1.009
2.011
3.007

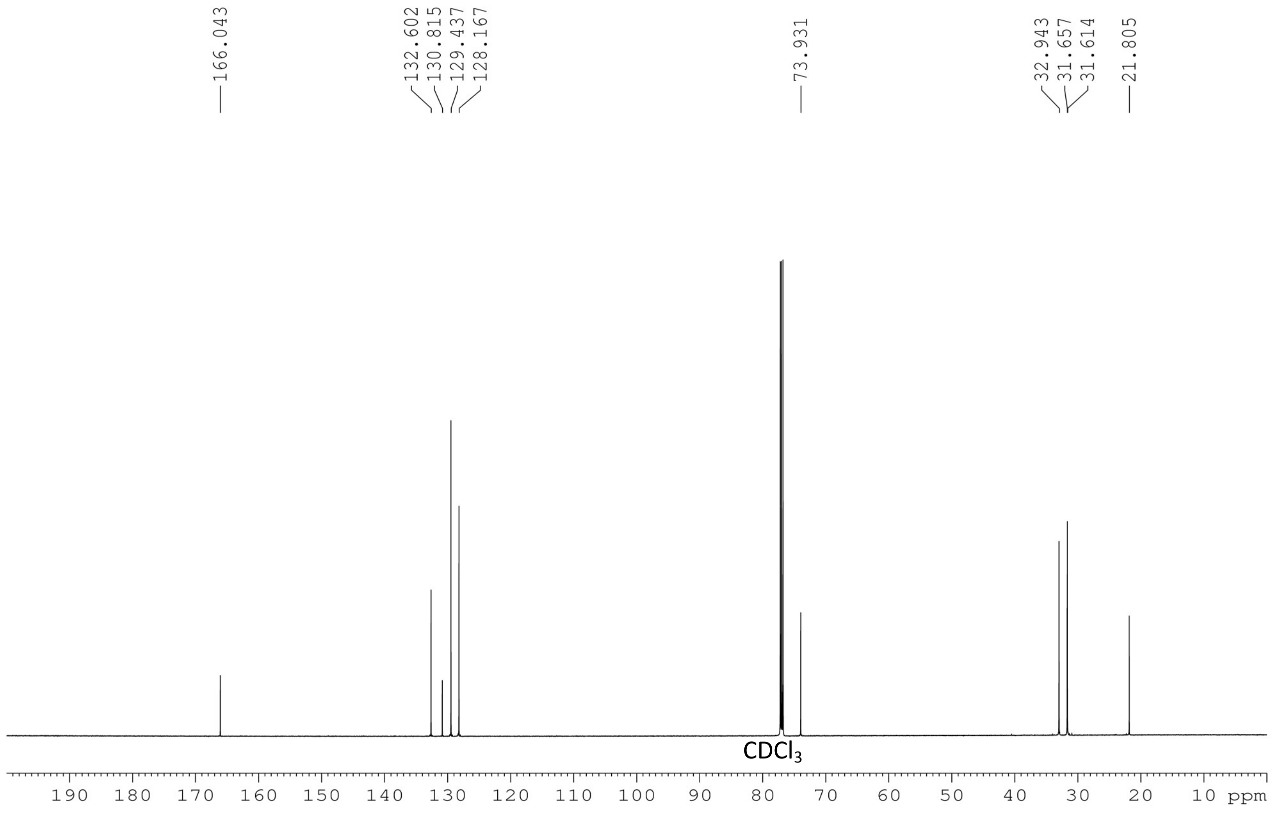

166.043
132.602
130.815
129.437
128.167
73.931
32.943
31.657
31.614
21.805
CDCl3
190 180 170 160 150 140 130 120 110 100 90 80 70 60 50 40 30 20 10 ppm

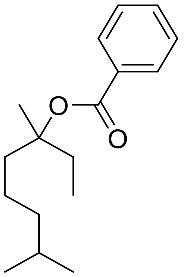

24

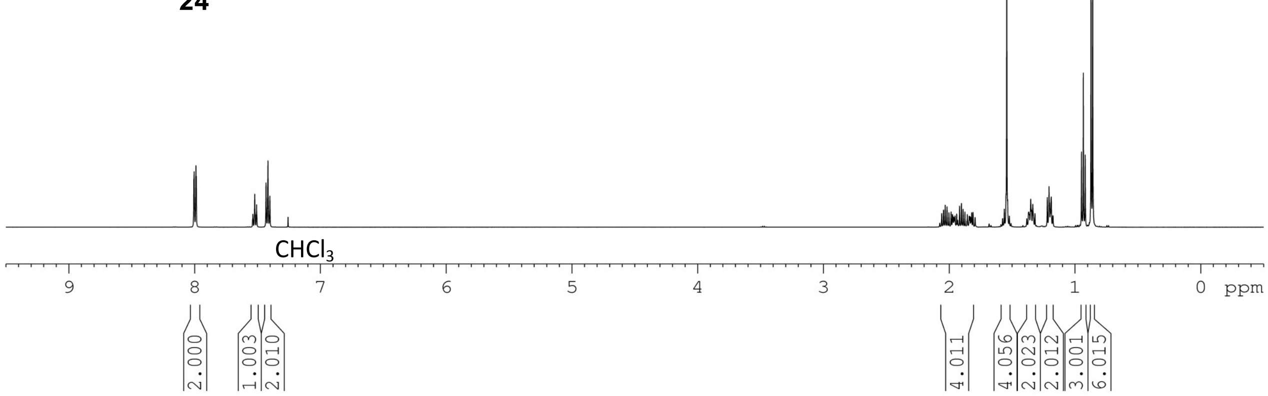

8.005 8.004 7.989 7.987 7.537 7.522 7.510 7.507 7.505 7.432 7.417 7.402 2.060 2.045 2.032 2.017 2.002 1.985 1.974 1.968 1.954 1.940 1.918 1.903 1.889 1.875 1.855 1.841 1.822 1.811 1.562 1.548 1.542 1.535 1.522 1.369 1.367 1.364 1.352 1.345 1.335 1.318 1.220 1.206 1.203 1.196 1.188 1.177 1.174 0.948 0.933 0.918 0.872 0.859
CHCl3
9 8 7 6 5 4 3 2 1 0 ppm
2.000 1.003 2.010 4.011 4.056 2.023 2.012 3.001 6.015

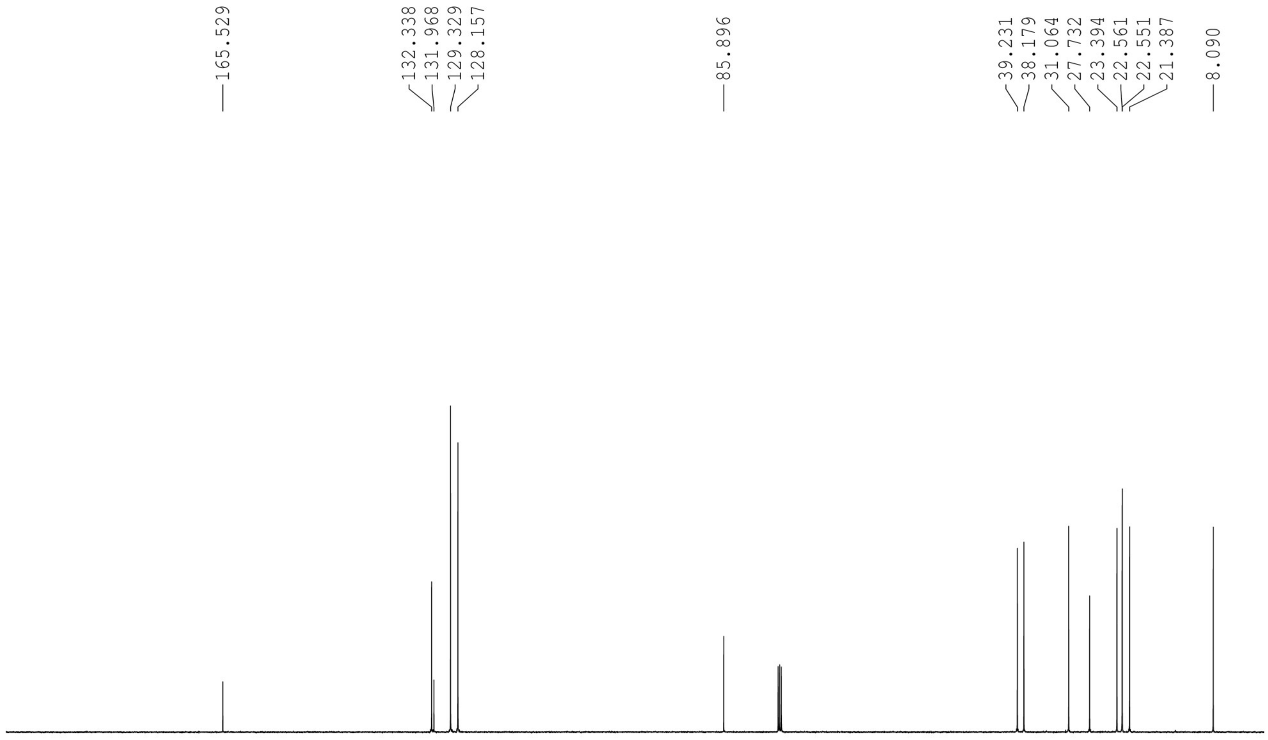

165.529
132.338 131.968 129.329 128.157
85.896
39.231 38.179 31.064 27.732 23.394 22.561 22.551 21.387
8.090
CDCl3
190 180 170 160 150 140 130 120 110 100 90 80 70 60 50 40 30 20 10 ppm

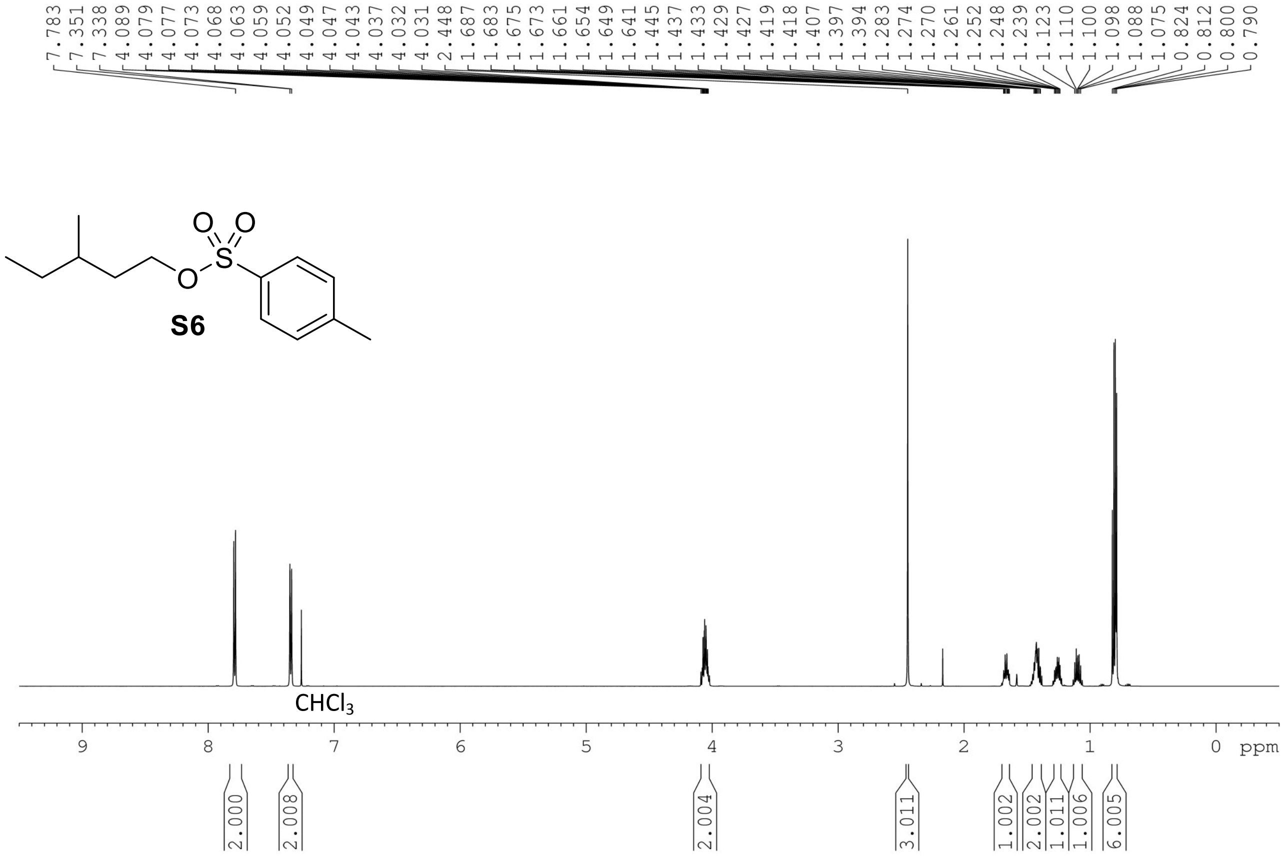
7.783
7.351
7.338
4.089
4.079
4.077
4.073
4.068
4.063
4.059
4.052
4.049
4.047
4.043
4.037
4.032
4.031
2.448
1.687
1.683
1.675
1.673
1.661
1.654
1.649
1.641
1.445
1.437
1.433
1.429
1.427
1.419
1.418
1.407
1.397
1.394
1.283
1.274
1.270
1.261
1.252
1.248
1.239
1.123
1.110
1.100
1.098
1.088
1.075
0.824
0.812
0.800
0.790
S6
CHCl3
9 8 7 6 5 4 3 2 1 0 ppm
2.000
2.008
2.004
3.011
1.002
2.002
1.011
1.006
6.005

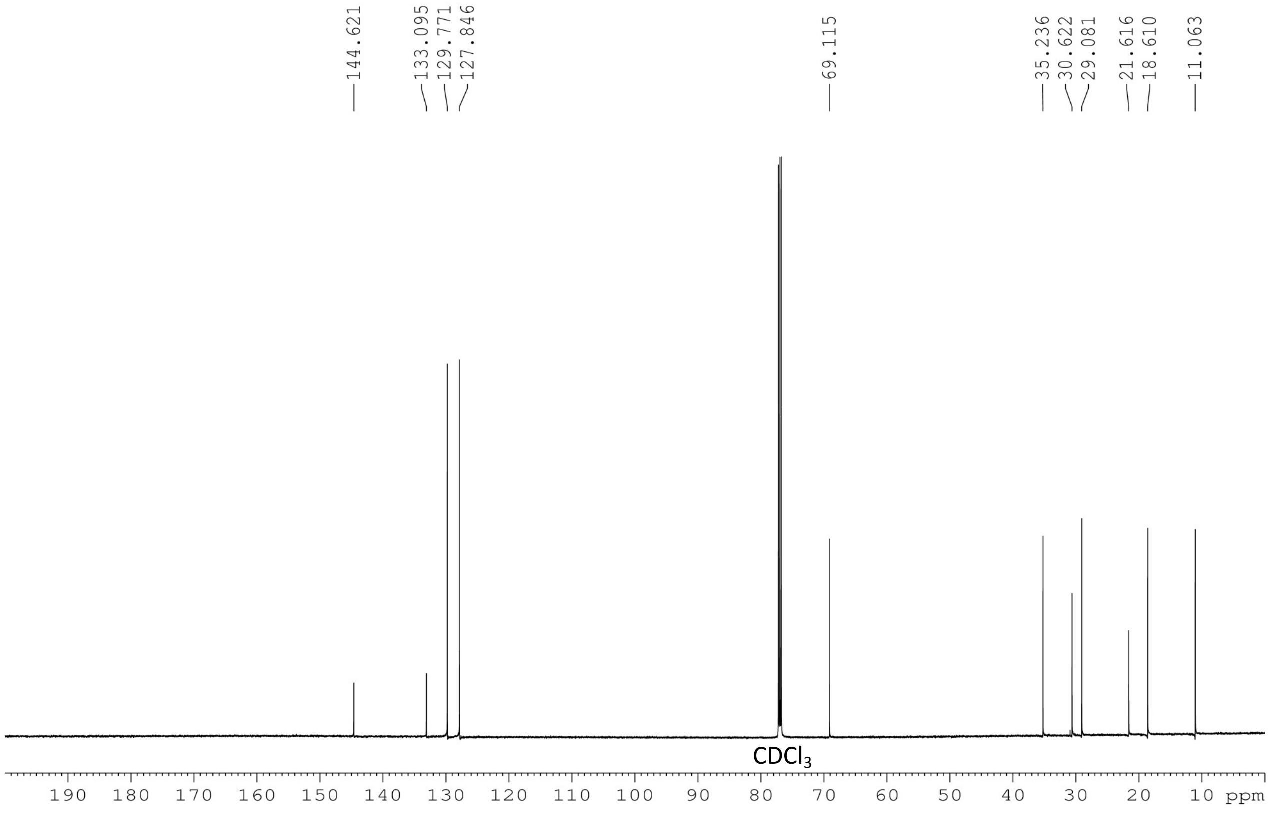
144.621
133.095
129.771
127.846
69.115
35.236
30.622
29.081
21.616
18.610
11.063
CDCl3
190 180 170 160 150 140 130 120 110 100 90 80 70 60 50 40 30 20 10 ppm

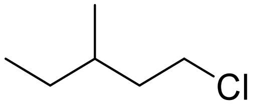

S7

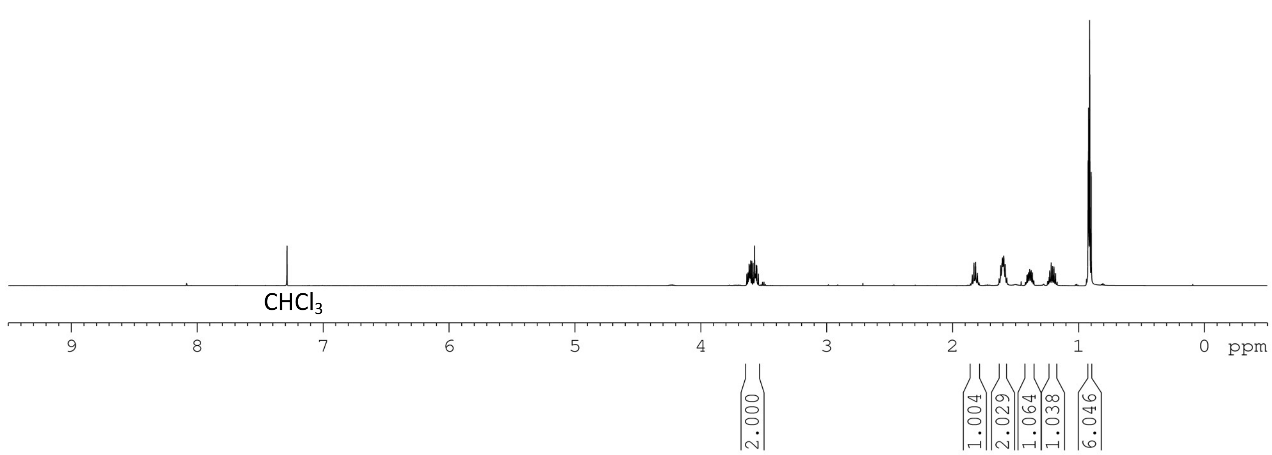
3.634 3.625 3.621 3.616 3.612 3.607 3.603 3.594 3.584 3.572 3.566 3.563 3.560 3.554 3.542 1.843 1.830 1.824 1.817 1.804 1.631 1.621 1.617 1.614 1.608 1.605 1.603 1.599 1.594 1.592 1.582 1.569 1.410 1.401 1.397 1.391 1.387 1.379 1.375 1.366 1.362 1.354 1.234 1.229 1.222 1.216 1.204 1.194 1.182 0.924 0.920 0.911 0.909 0.899
CHCl3
9 8 7 6 5 4 3 2 1 0 ppm
2.000 1.004 2.029 1.064 1.038 6.046

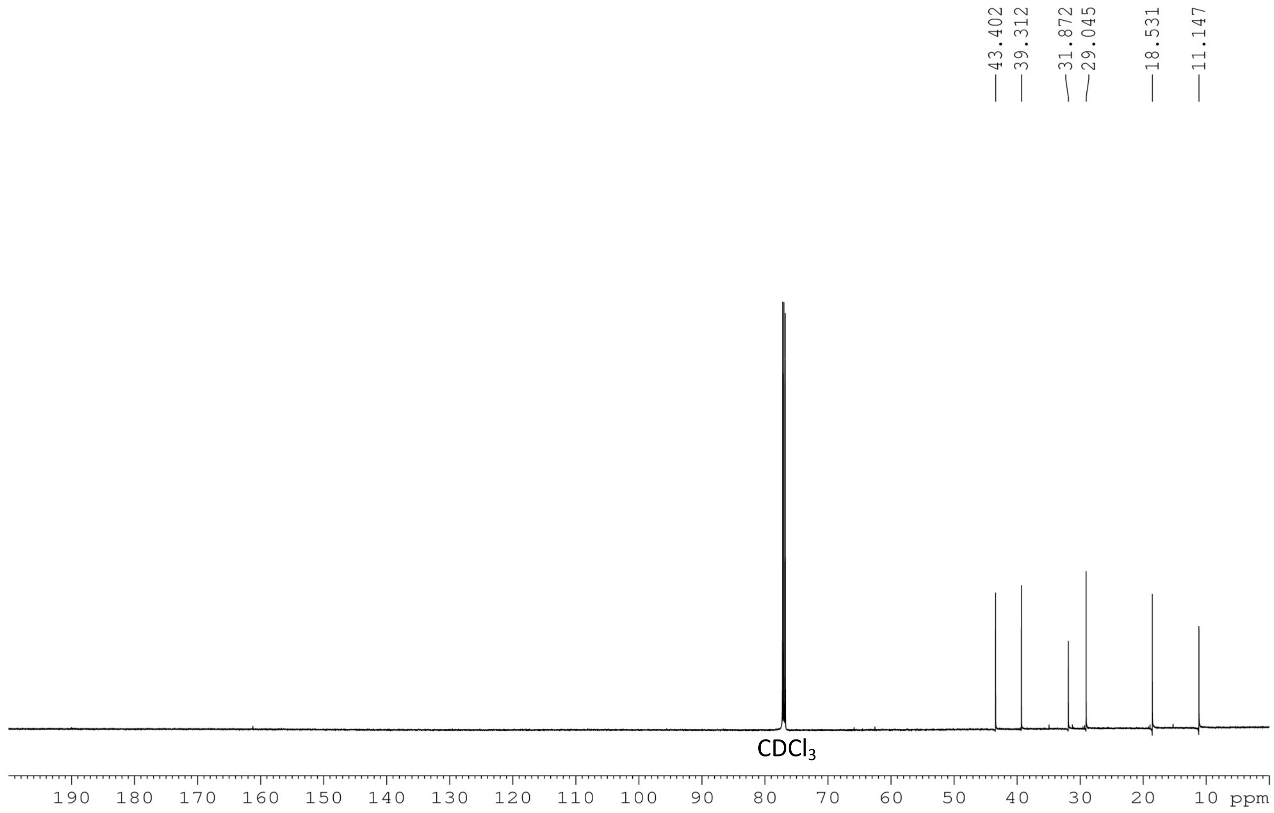
43.402 39.312 31.872 29.045 18.531 11.147
CDCl3
190 180 170 160 150 140 130 120 110 100 90 80 70 60 50 40 30 20 10 ppm

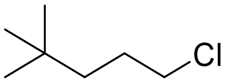
Cl

S8

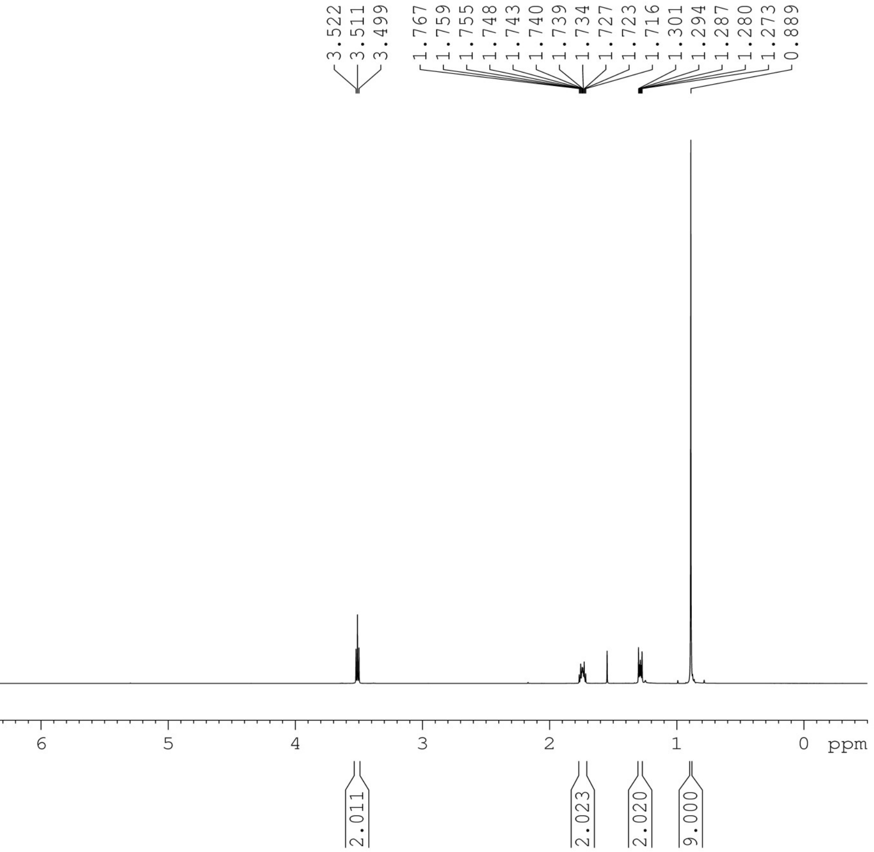
3.522
3.511
3.499
1.767
1.759
1.755
1.748
1.743
1.740
1.739
1.734
1.727
1.723
1.716
1.301
1.294
1.287
1.280
1.273
0.889
CHCl3
9
8
7
6
5
4
3
2
1
0 ppm
2.011
2.023
2.020
9.000

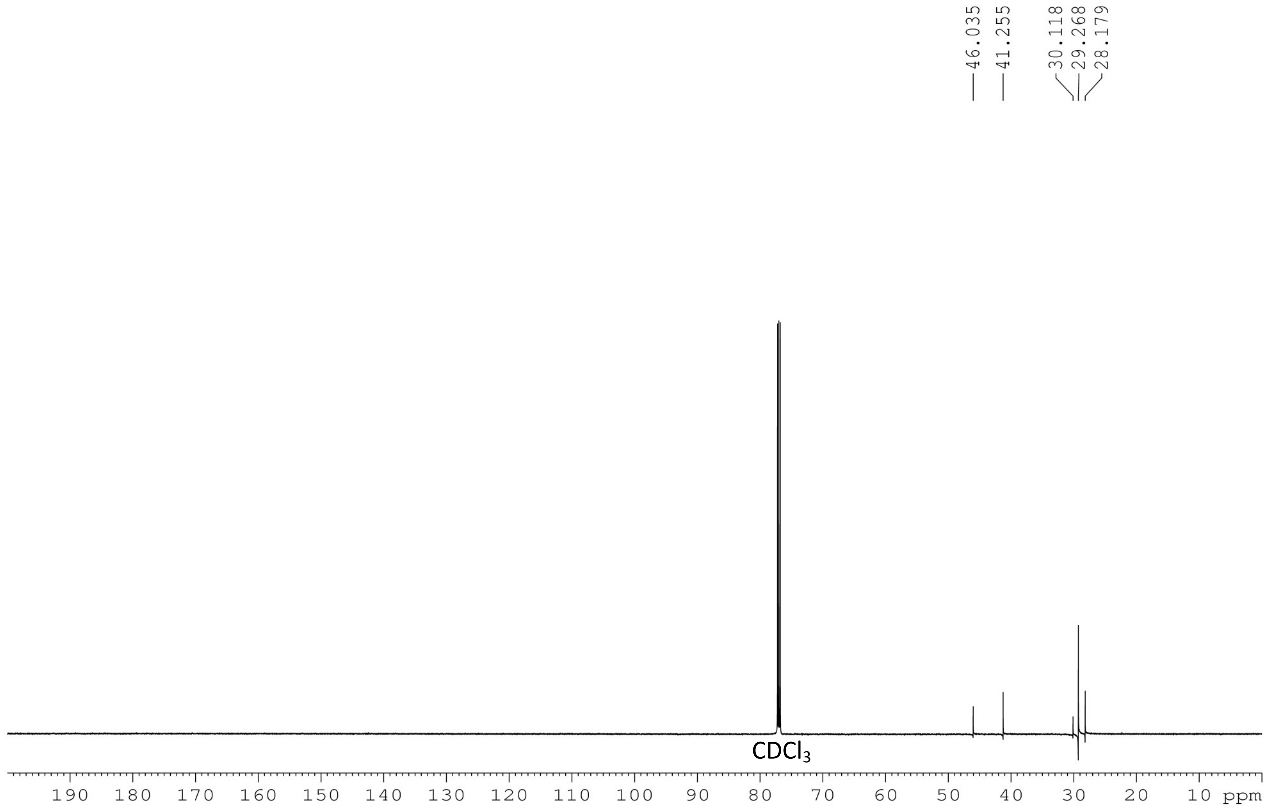
46.035
41.255
30.118
29.268
28.179
CDCl3
190
180
170
160
150
140
130
120
110
100
90
80
70
60
50
40
30
20
10 ppm

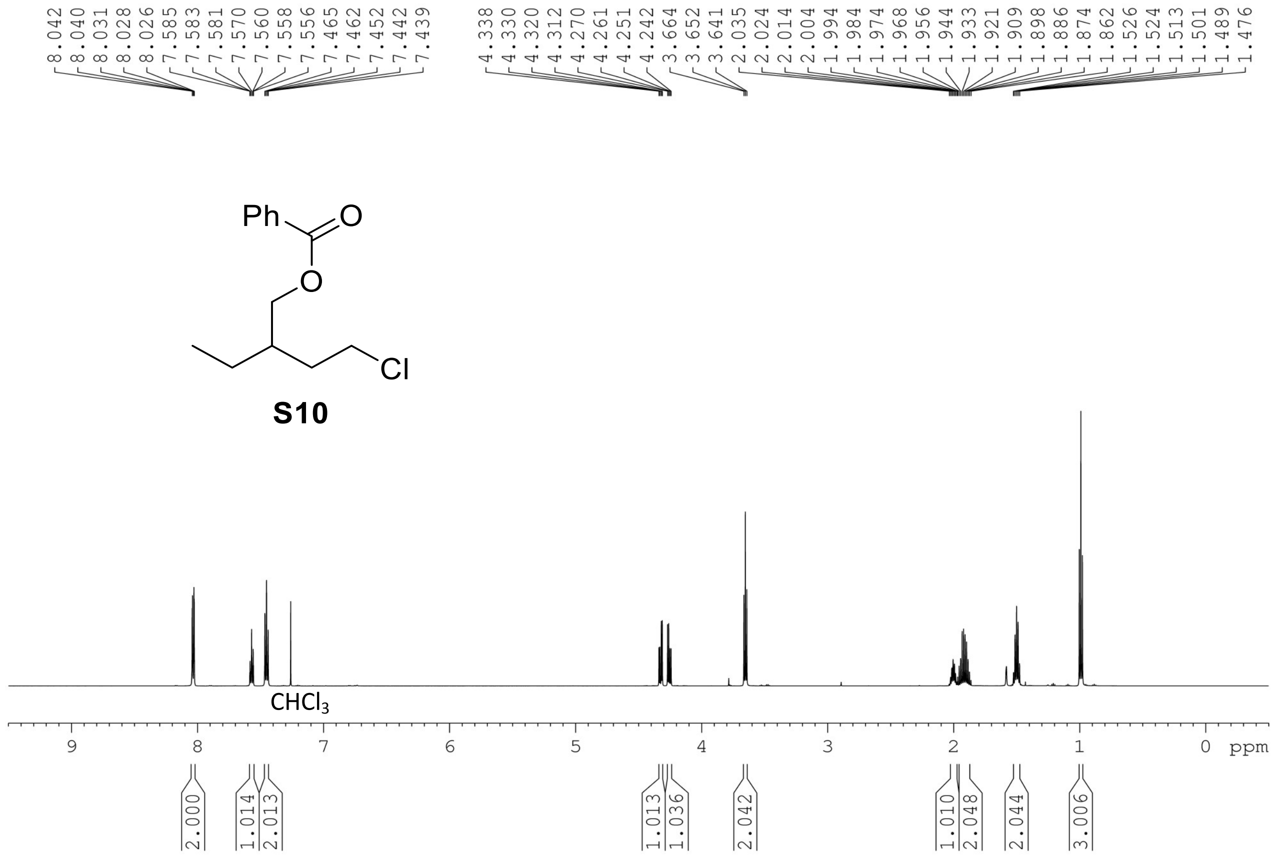
Ph
O
O
Cl
S10
8.042 8.040 8.031 8.028 8.026 7.585 7.583 7.581 7.570 7.560 7.558 7.556 7.465 7.462 7.452 7.442 7.439
4.338 4.330 4.320 4.312 4.270 4.261 4.251 4.242 3.664 3.652 3.641 2.035 2.024 2.014 2.004 1.994 1.984 1.974 1.968 1.956 1.944 1.933 1.921 1.909 1.898 1.886 1.874 1.862 1.526 1.524 1.513 1.501 1.489 1.476
CHCl$_3$
9 8 7 6 5 4 3 2 1 0 ppm
2.000 1.014 2.013 1.013 1.036 2.042 1.010 2.048 2.044 3.006

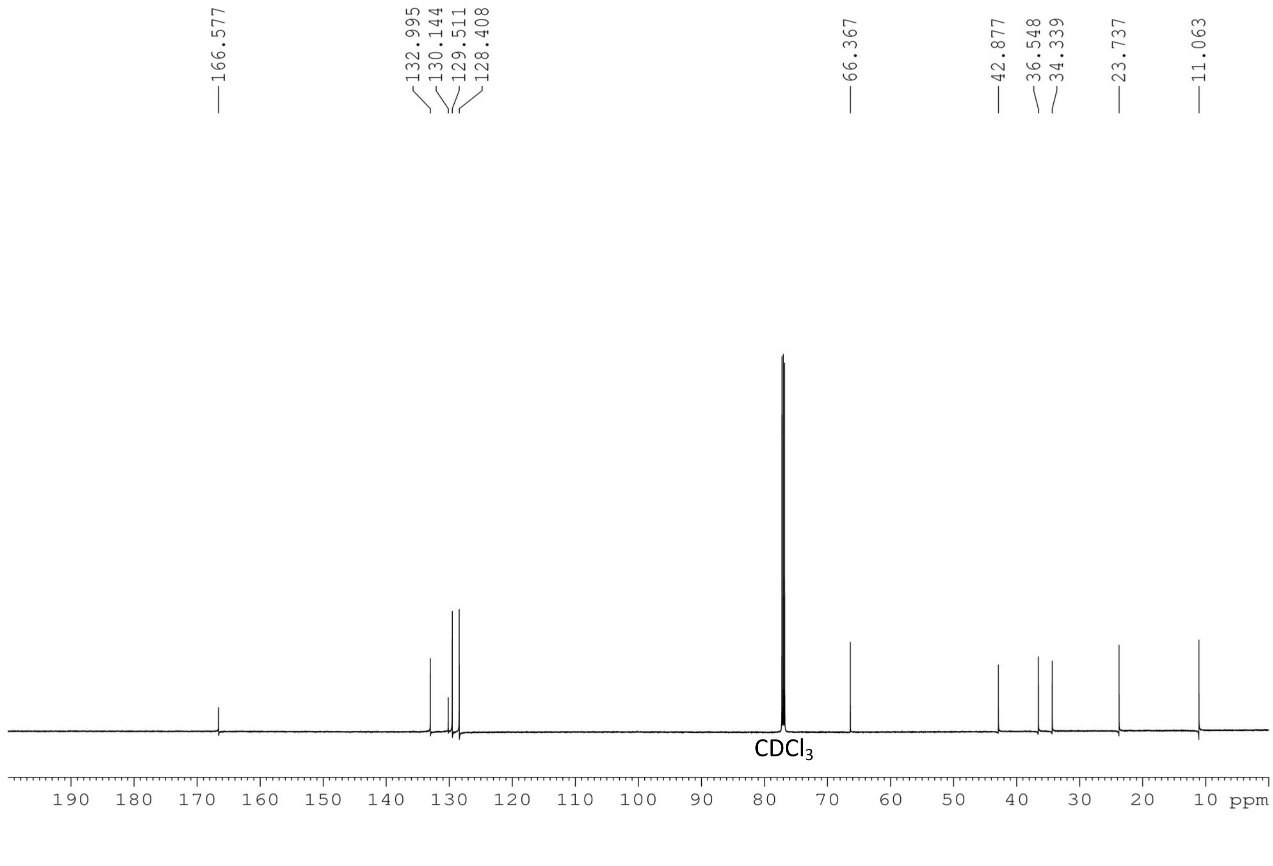
166.577
132.995 130.144 129.511 128.408
66.367
42.877 36.548 34.339 23.737 11.063
CDCl$_3$
190 180 170 160 150 140 130 120 110 100 90 80 70 60 50 40 30 20 10 ppm

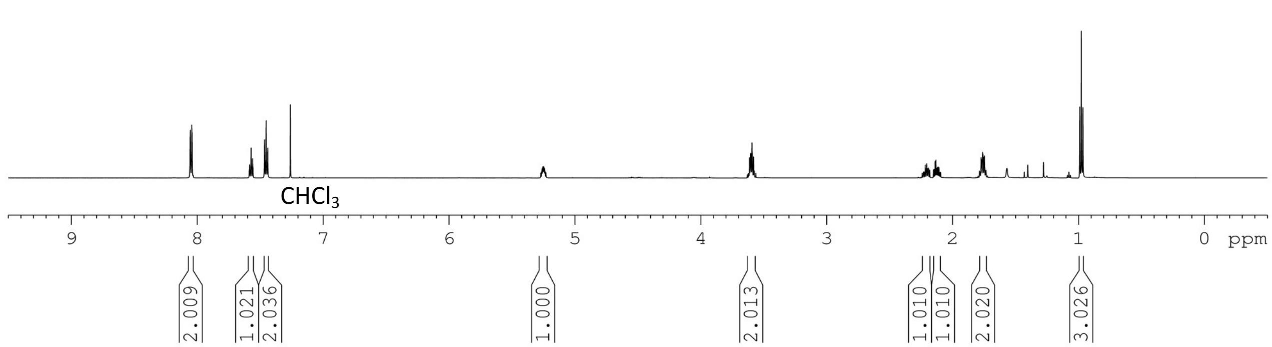

8.056
8.054
8.045
8.042
8.040
7.584
7.571
7.561
7.559
7.557
7.465
7.463
7.452
7.442
7.439
5.260
5.255
5.250
5.246
5.240
3.612
3.605
3.603
3.599
3.593
3.590
3.581
2.217
2.207
2.205
2.203
2.196
2.193
2.191
2.181
2.151
2.145
2.138
2.132
2.125
2.119
2.114
2.108
1.773
1.769
1.761
1.759
1.756
1.750
1.746
1.734
0.989
0.977
0.965
CHCl3
9 8 7 6 5 4 3 2 1 0 ppm
2.009
1.021
2.036
1.000
2.013
1.010
1.010
2.020
3.026

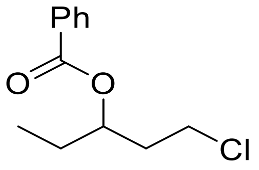

Ph
O
O
Cl

S12

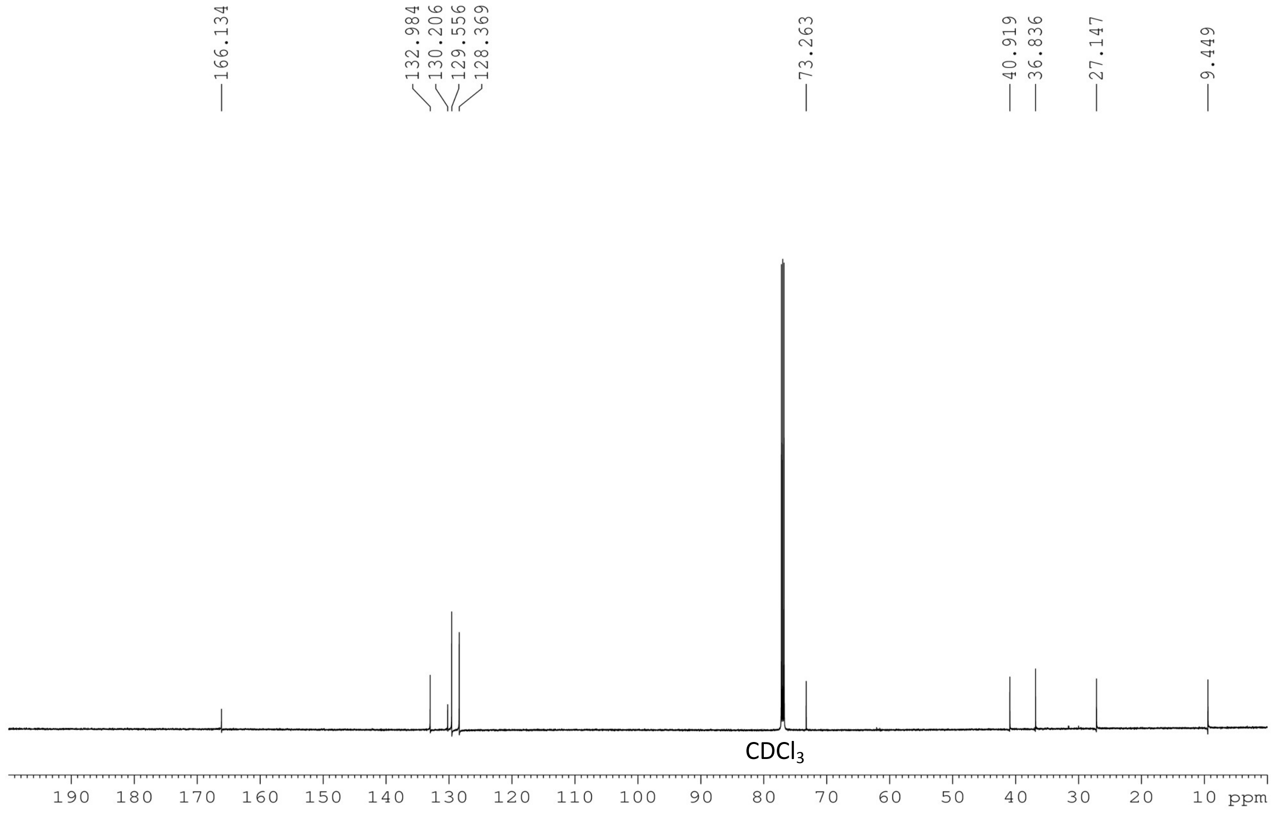

166.134
132.984
130.206
129.556
128.369
73.263
40.919
36.836
27.147
9.449
CDCl3
190 180 170 160 150 140 130 120 110 100 90 80 70 60 50 40 30 20 10 ppm

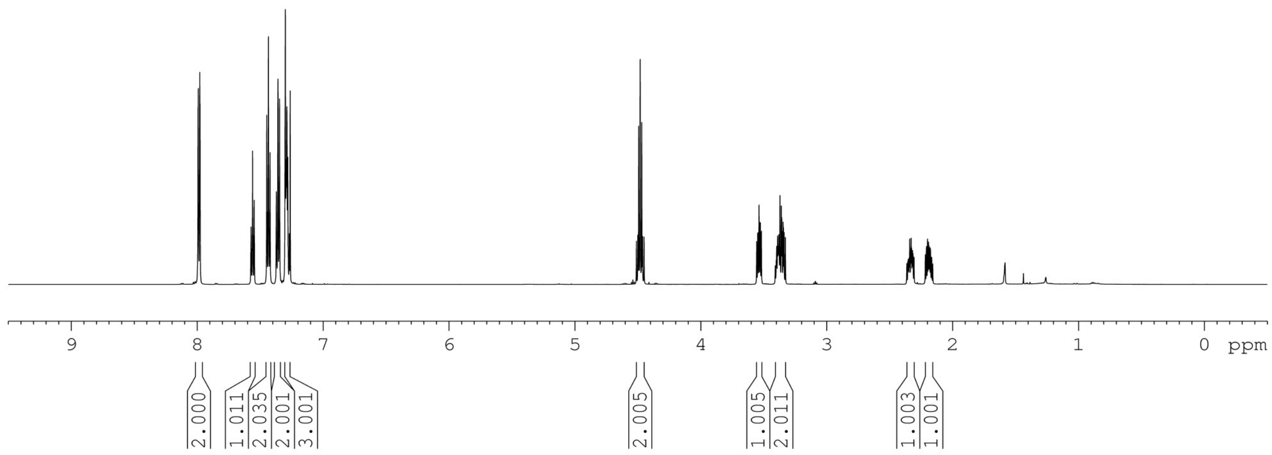
7.993
7.991
7.979
7.977
7.572
7.559
7.549
7.547
7.545
7.447
7.445
7.434
7.421
7.371
7.358
7.349
7.346
7.300
7.296
7.294
7.293
7.288
7.283
7.280
7.268
4.511
4.500
4.493
4.481
4.469
4.462
4.450
3.556
3.548
3.545
3.537
3.530
3.527
3.518
3.389
3.379
3.371
3.361
3.356
3.353
3.346
3.343
3.338
3.328
2.340
2.329
2.199
2.191
2.189
2.000
1.011
2.035
2.001
3.001
2.005
1.005
2.011
1.003
1.001
ppm

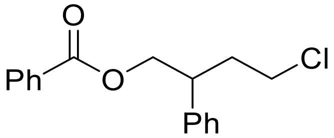
O
Ph
O
Cl
Ph

S13

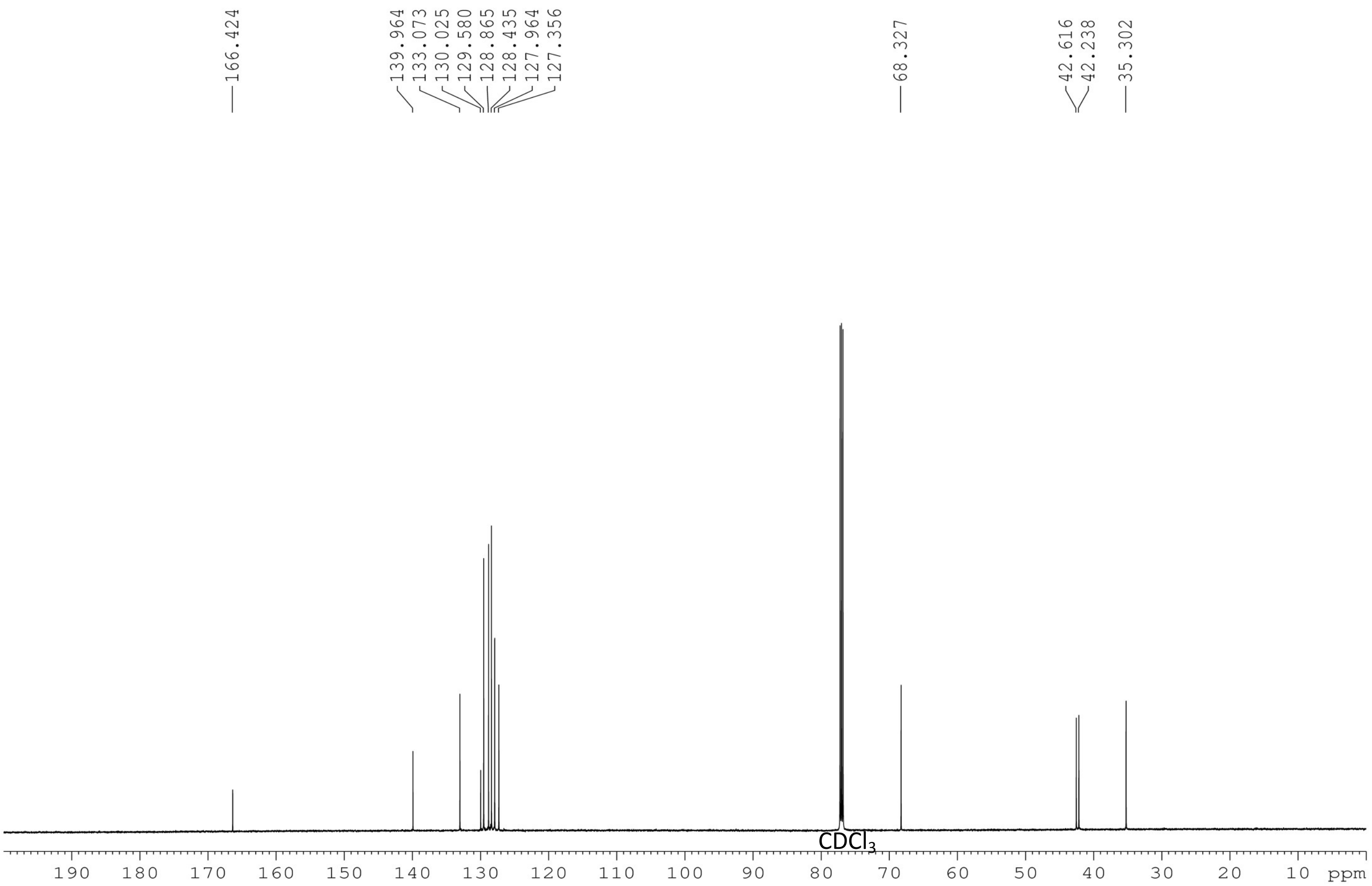
166.424
139.964
133.073
130.025
129.580
128.865
128.435
127.964
127.356
68.327
42.616
42.238
35.302
CDCl3
ppm

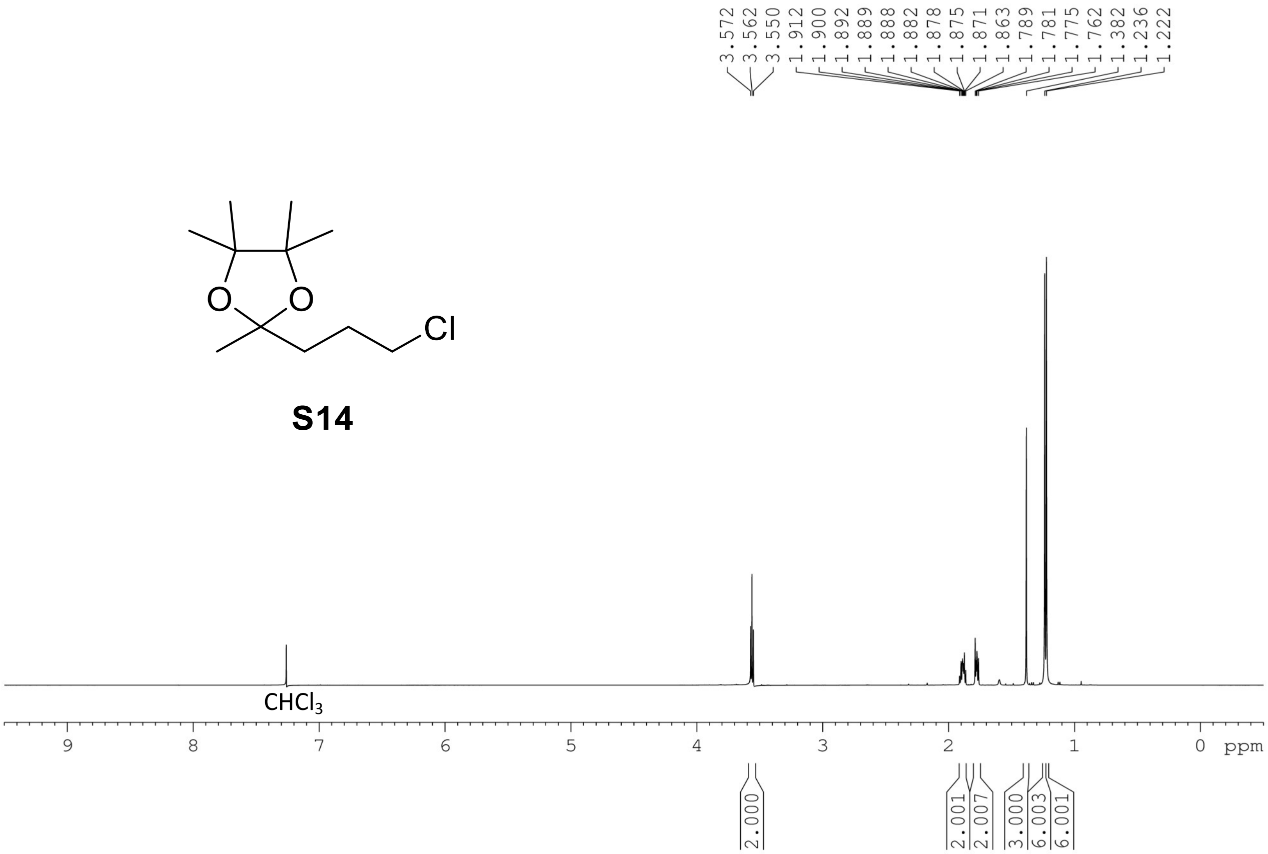

S14

3.572
3.562
3.550
1.912
1.900
1.892
1.889
1.888
1.882
1.878
1.875
1.871
1.863
1.789
1.781
1.775
1.762
1.382
1.236
1.222
$CHCl_3$
9 8 7 6 5 4 3 2 1 0 ppm
2.000
2.001
2.007
3.000
6.003
6.001

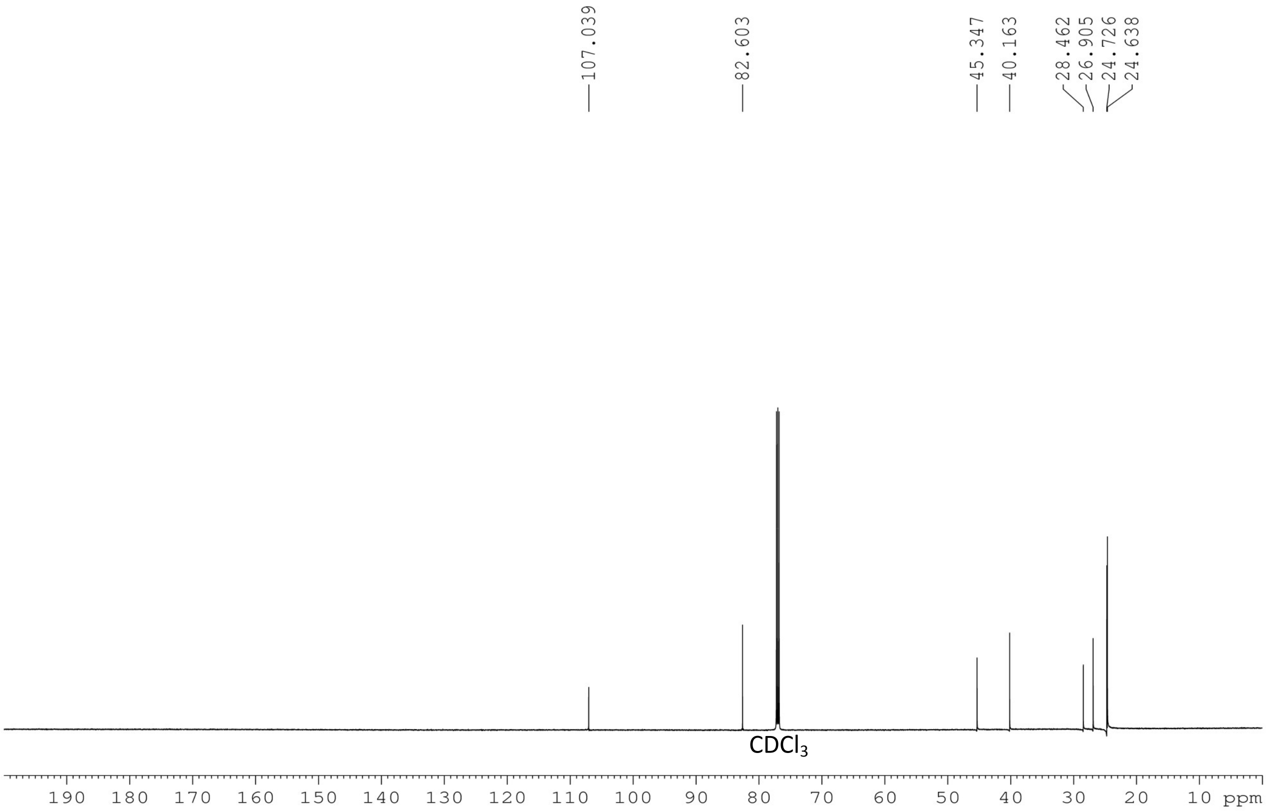
107.039
82.603
45.347
40.163
28.462
26.905
24.726
24.638
$CDCl_3$
190 180 170 160 150 140 130 120 110 100 90 80 70 60 50 40 30 20 10 ppm

S15

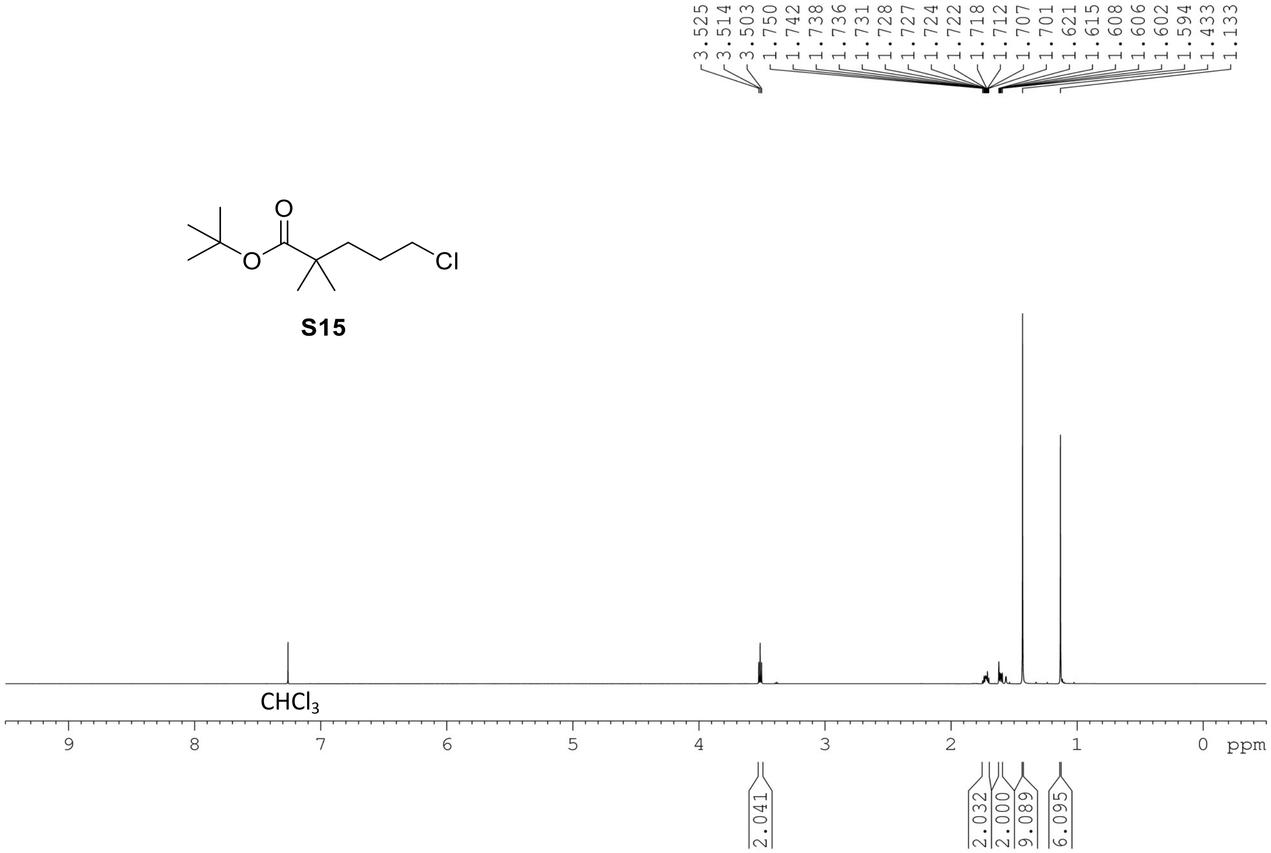
O
O
Cl
3.525
3.514
3.503
1.750
1.742
1.738
1.736
1.731
1.728
1.727
1.724
1.722
1.718
1.712
1.707
1.701
1.621
1.615
1.608
1.606
1.602
1.594
1.433
1.133
CHCl3
9 8 7 6 5 4 3 2 1 0 ppm
2.041
2.032
2.000
9.089
6.095

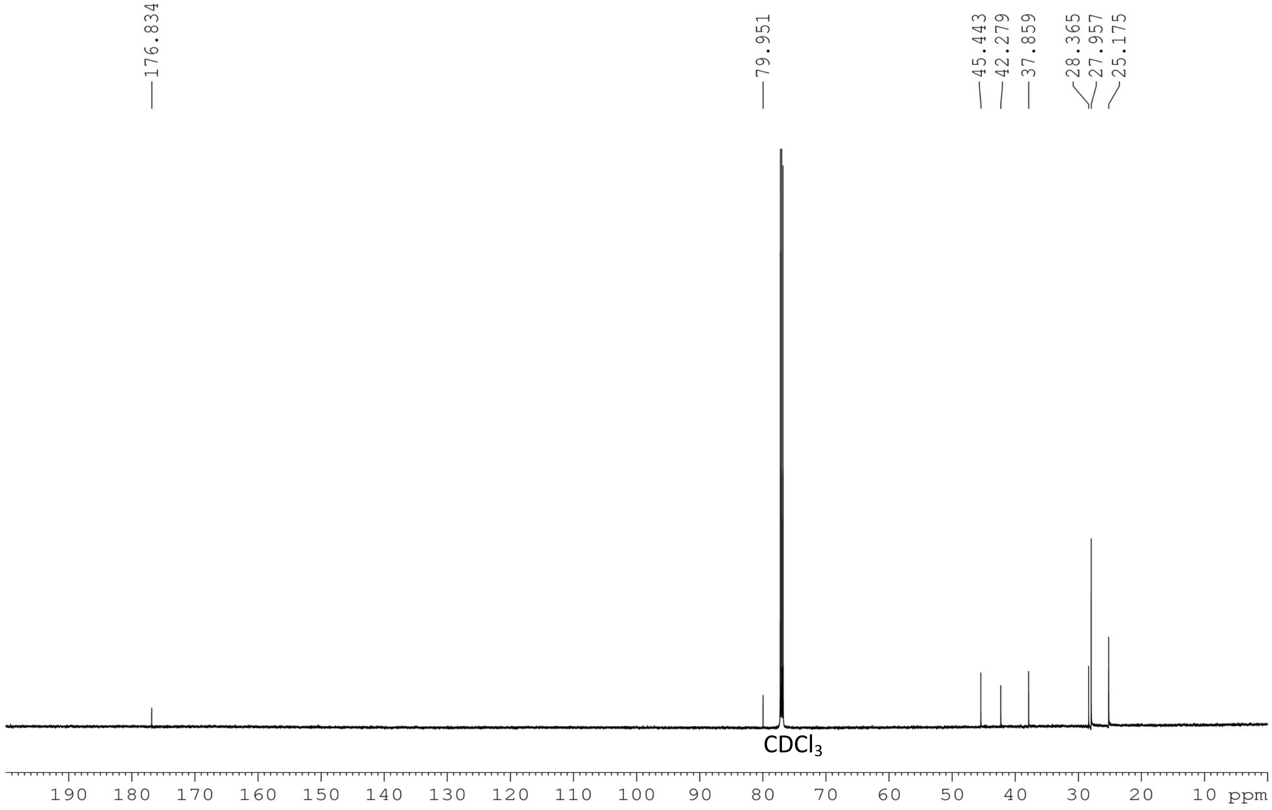
176.834
79.951
45.443
42.279
37.859
28.365
27.957
25.175
CDCl3
190 180 170 160 150 140 130 120 110 100 90 80 70 60 50 40 30 20 10 ppm

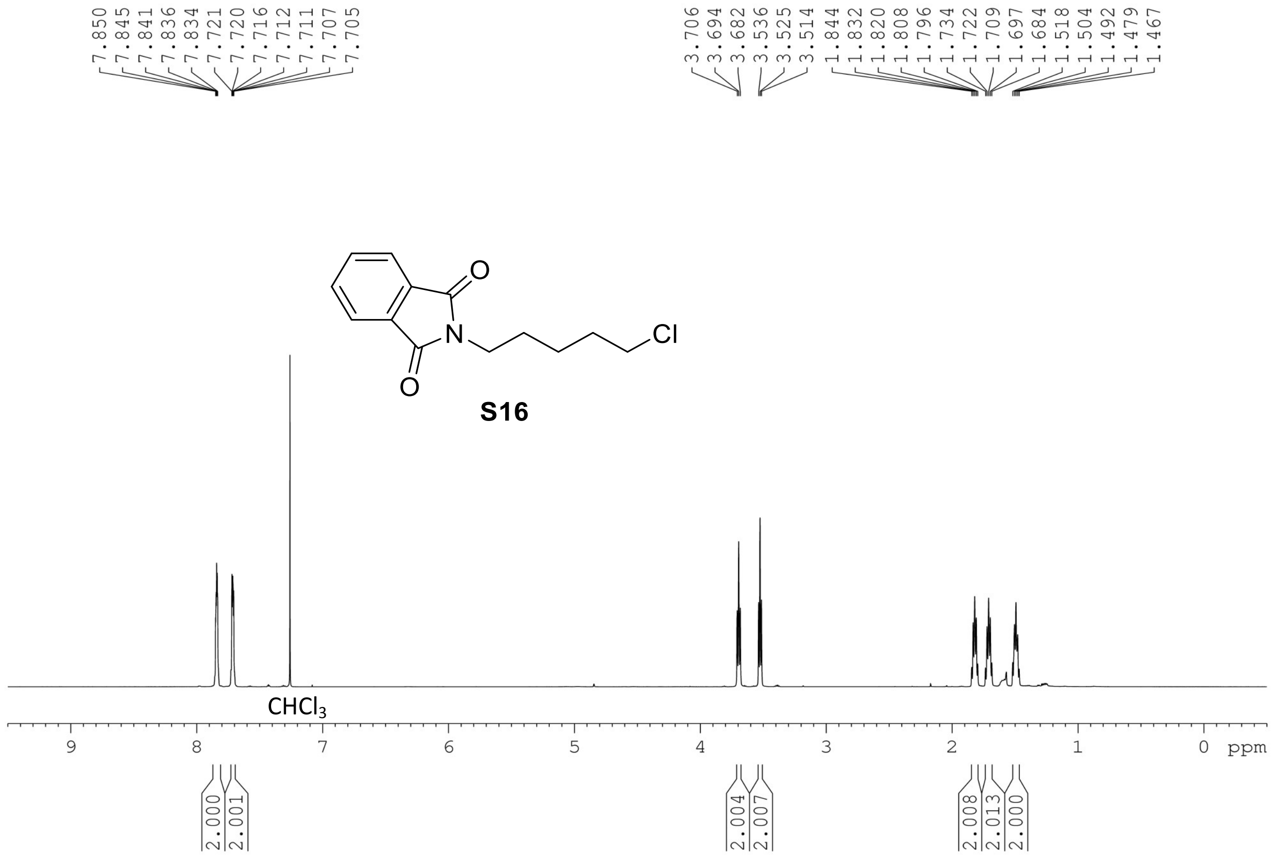
7.850
7.845
7.841
7.836
7.834
7.721
7.720
7.716
7.712
7.711
7.707
7.705
3.706
3.694
3.682
3.536
3.525
3.514
1.844
1.832
1.820
1.808
1.796
1.734
1.722
1.709
1.697
1.684
1.518
1.504
1.492
1.479
1.467
O
N
Cl
O
CHCl$_3$
9 8 7 6 5 4 3 2 1 0 ppm
2.000
2.001
2.004
2.007
2.008
2.013
2.000

S16

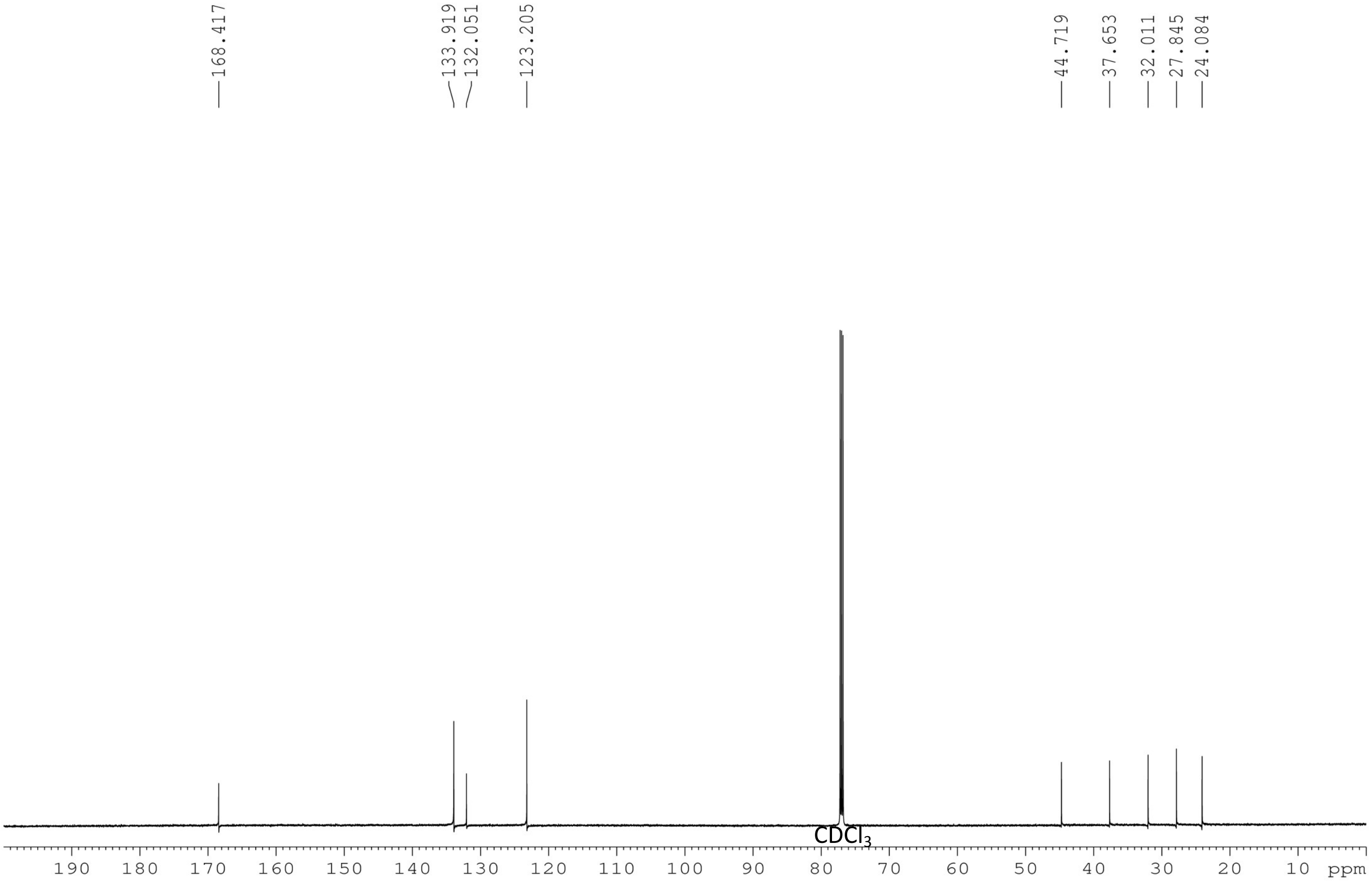
168.417
133.919
132.051
123.205
44.719
37.653
32.011
27.845
24.084
CDCl$_3$
190 180 170 160 150 140 130 120 110 100 90 80 70 60 50 40 30 20 10 ppm

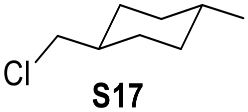

S17

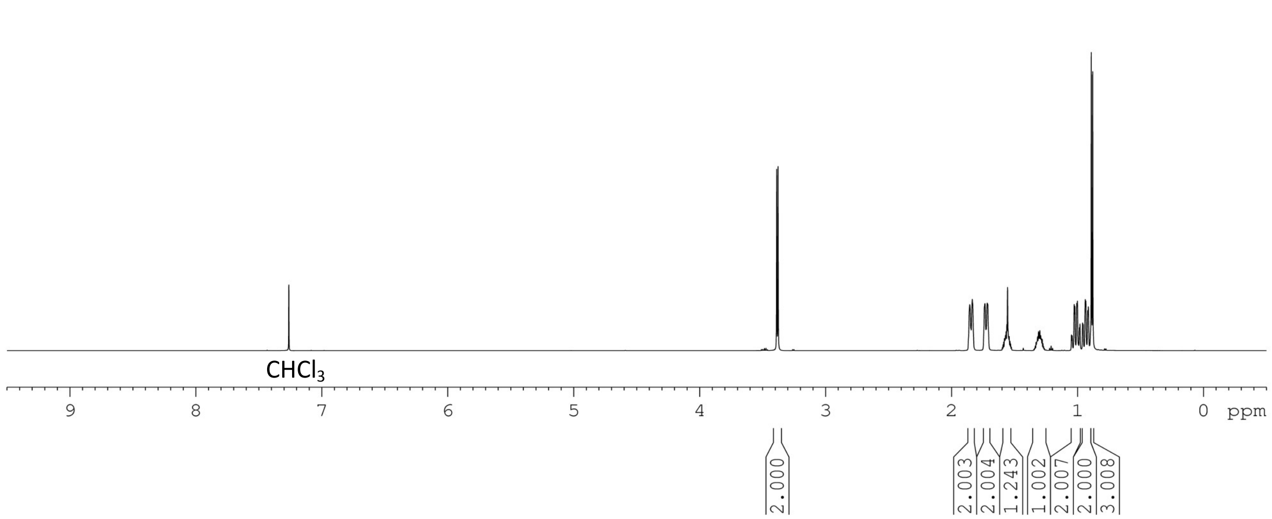
3.389
3.378
1.856
1.836
1.735
1.717
1.713
1.597
1.592
1.586
1.581
1.576
1.572
1.567
1.562
1.556
1.542
1.537
1.532
1.526
1.347
1.340
1.335
1.329
1.324
1.317
1.311
1.305
1.300
1.293
1.286
CHCl3
9 8 7 6 5 4 3 2 1 0 ppm
2.000
2.003
2.004
1.243
1.002
2.007
2.000
3.008

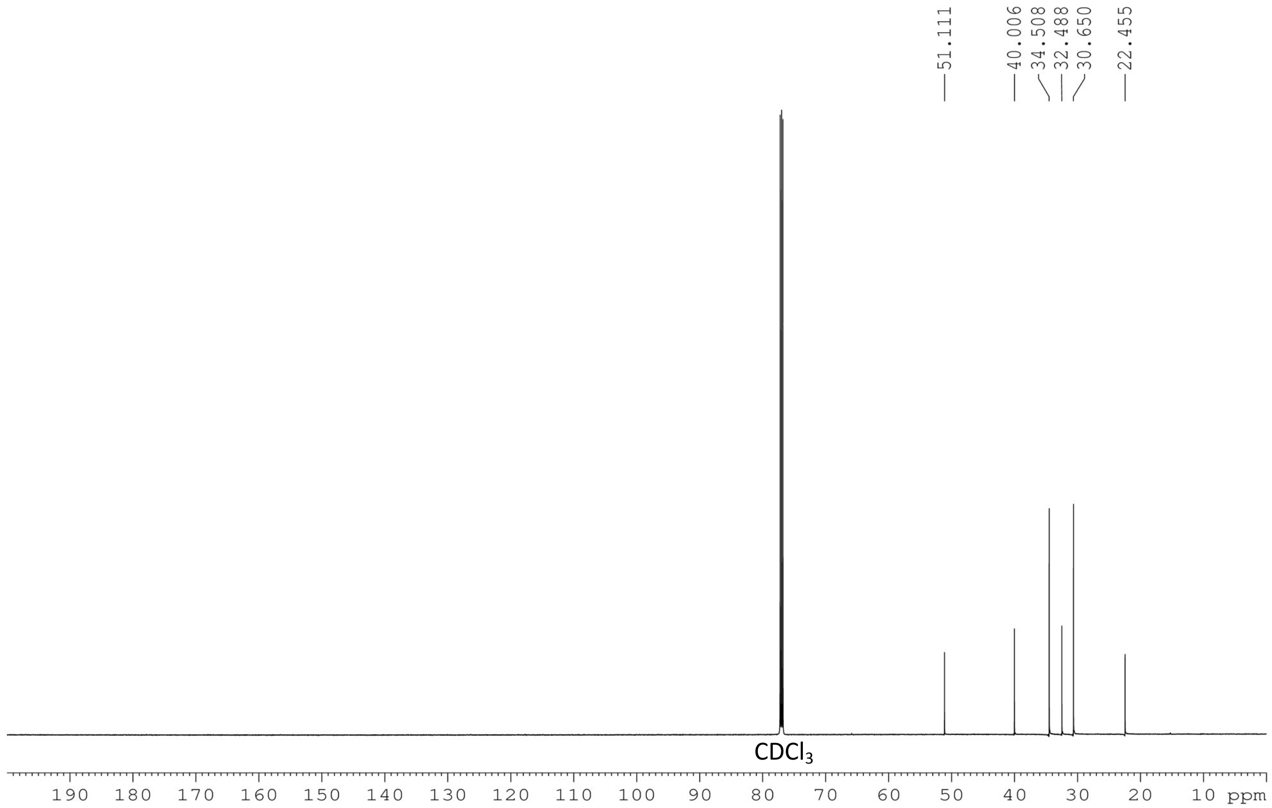
51.111
40.006
34.508
32.488
30.650
22.455
CDCl3
190 180 170 160 150 140 130 120 110 100 90 80 70 60 50 40 30 20 10 ppm

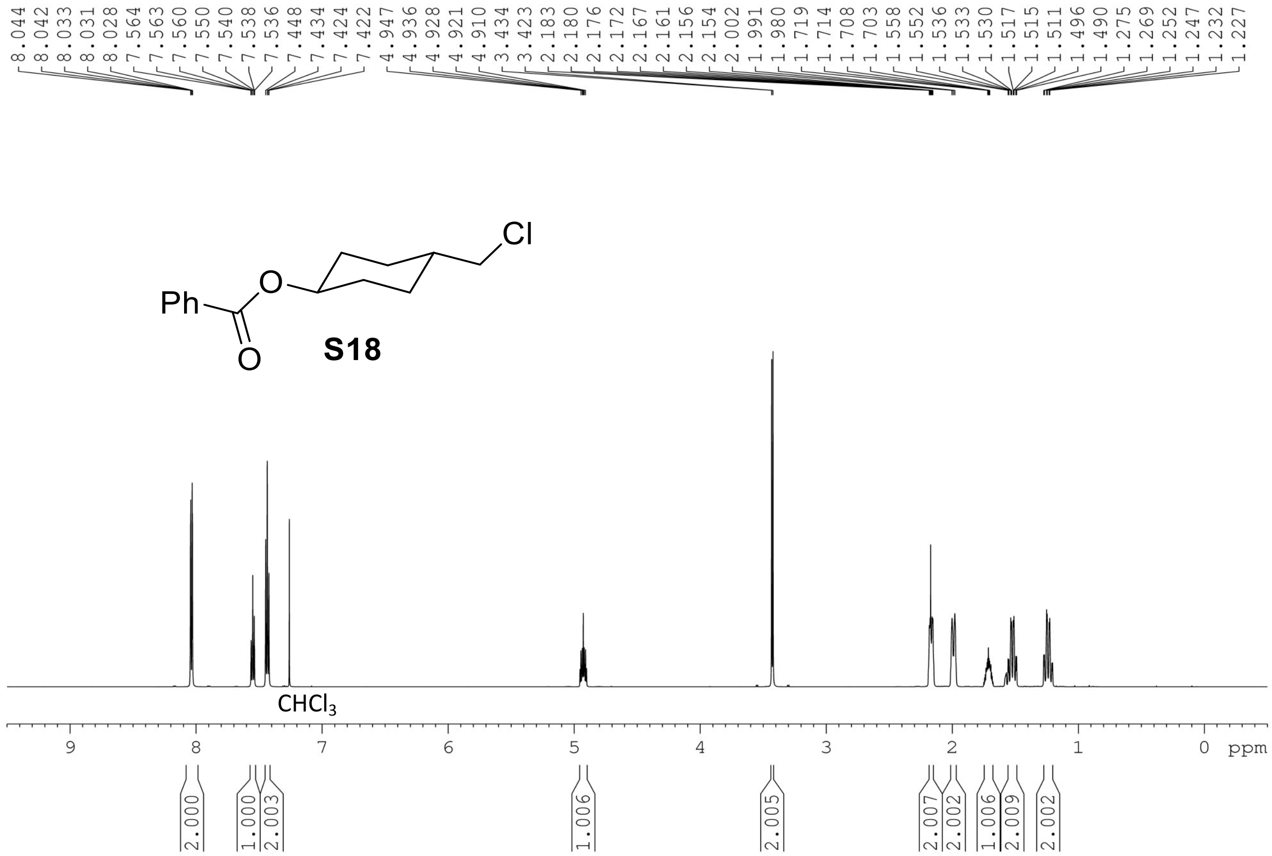
8.044 8.042 8.033 8.031 8.028 7.564 7.563 7.560 7.550 7.540 7.538 7.536 7.448 7.434 7.424 7.422 4.947 4.936 4.928 4.921 4.910 3.434 3.423 2.183 2.180 2.176 2.172 2.167 2.161 2.156 2.154 2.002 1.991 1.980 1.719 1.714 1.708 1.703 1.558 1.552 1.536 1.533 1.530 1.517 1.515 1.511 1.496 1.490 1.275 1.269 1.252 1.247 1.232 1.227
Cl
O
Ph
O
S18
CHCl$_3$
9 8 7 6 5 4 3 2 1 0 ppm
2.000 1.000 2.003 1.006 2.005 2.007 2.002 1.006 2.009 2.002

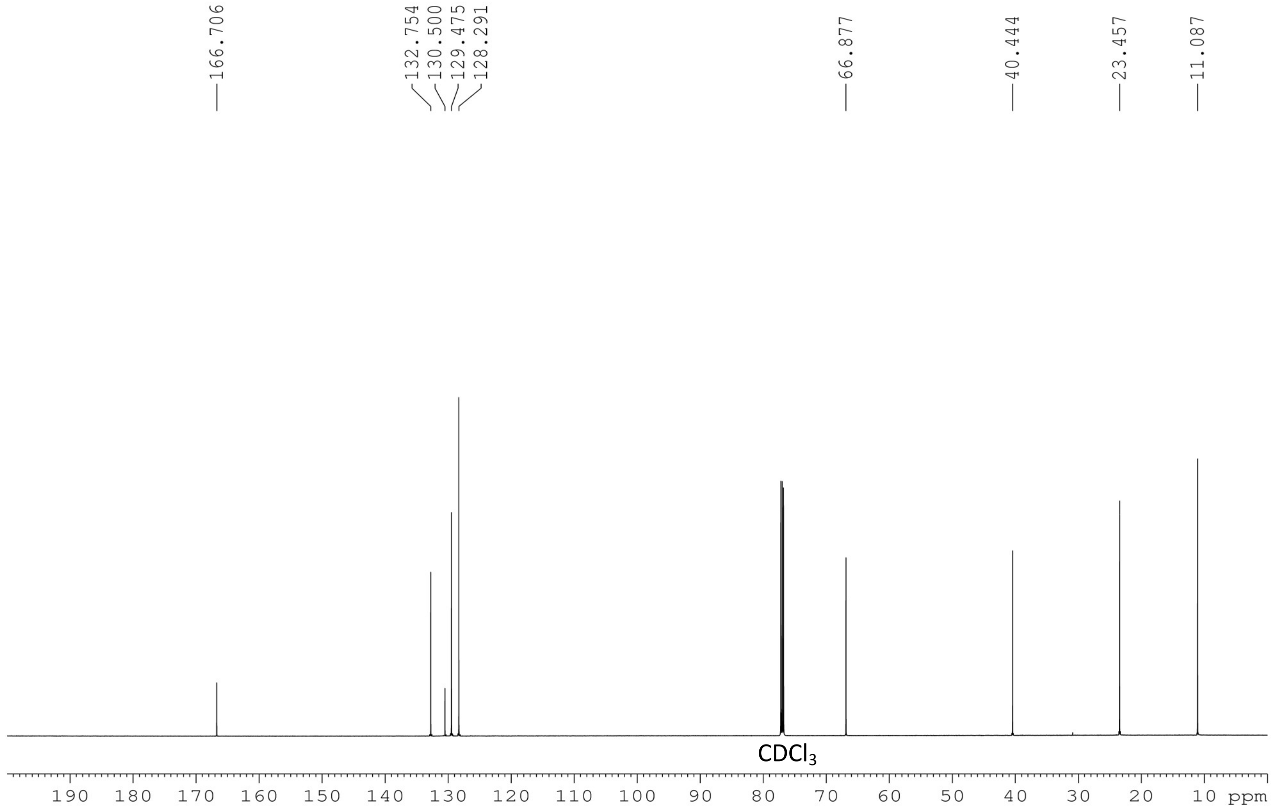
166.706
132.754 130.500 129.475 128.291
66.877
40.444
23.457
11.087
CDCl$_3$
190 180 170 160 150 140 130 120 110 100 90 80 70 60 50 40 30 20 10 ppm
